# Crosslinking pathways, dynamics, and kinetics between guanosine and lysine following one- versus two-electron oxidation of guanosine

**DOI:** 10.1093/nar/gkaf071

**Published:** 2025-03-04

**Authors:** May Myat Moe, Jonathan Benny, Varonica Lee, Midas Tsai, Jianbo Liu

**Affiliations:** Department of Chemistry and Biochemistry, Queens College of the City University of New York, 65-30 Kissena Blvd., Queens, NY 11367, United States; Ph.D. Program in Chemistry, the Graduate Center of the City University of New York, 365 5th Ave., NY, NY 10016, United States; Department of Chemistry and Biochemistry, Queens College of the City University of New York, 65-30 Kissena Blvd., Queens, NY 11367, United States; Ph.D. Program in Chemistry, the Graduate Center of the City University of New York, 365 5th Ave., NY, NY 10016, United States; Department of Chemistry and Biochemistry, Queens College of the City University of New York, 65-30 Kissena Blvd., Queens, NY 11367, United States; Department of Natural Sciences, LaGuardia Community College, 31-10 Thomson Ave., Long Island City, NY 11101, United States; Department of Chemistry and Biochemistry, Queens College of the City University of New York, 65-30 Kissena Blvd., Queens, NY 11367, United States; Ph.D. Program in Chemistry, the Graduate Center of the City University of New York, 365 5th Ave., NY, NY 10016, United States

## Abstract

DNA–protein crosslinks (DPCs) remain as a poorly understood DNA lesion. Herein, crosslinking between guanosine and lysine was explored using a model system comprising 9-methylguanine (9MG) and CH_3_NH_2_. Crosslinking was induced by one-electron oxidized 9MG^•+^ radical cations and doubly oxidized [9MG – H_N2_]^+^ cations, and analyzed as a function of reaction energy using an electrospray ionization tandem mass spectrometer. Experiment was augmented by dynamics simulations and kinetics modeling. Alongside the formation of X-NH_2_CH_3_[9MG]^•+^ (*X* = C2, C8) via direct addition, 8-CH_2_NH_2_[9MG + H_N7_]^+^ was discovered as a new crosslink between 9MG^•+^ and CH_3_NH_2_. This crosslink results from methyl–hydrogen abstraction of CH_3_NH_2_ by the N7 of 9MG^•+^, followed by adding ^•^CH_2_NH_2_ to [9MG + H_N7_]^+^. Notably, crosslinking is dramatically enhanced between [9MG – H_N2_]^+^ and CH_3_NH_2_, yielding major products X-^+^NH_2_CH_3_[9MG – H_N2_] (*X* = N2, N3, C5, and C8, along with their proton tautomers), which form from the direct CH_3_NH_2_ addition to [9MG – H_N2_]^+^, and minor products X-CH_2_NH_2_[9MG – H_N2_ + H_O6_]^+^ (*X* = N2, N3, C5, N7, and C8), which arise from the combination of methyl–hydrogen abstraction products. This work dissected and distinguished the roles of one- versus two-electron oxidized guanosine in DPC formation, offering novel insights into oxidative DNA damage.

## Graphical Abstract

**Graphical Abstract:**
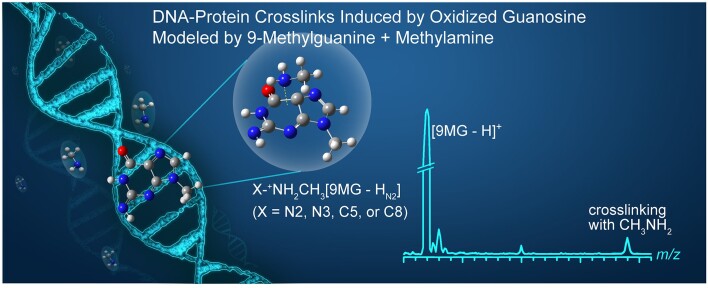

## Introduction

Proteins bind to DNA during cellular activities such as replication, transcription, and repair [1, 2]. These interactions are precisely regulated and primarily non-covalent [3]. By contrast, DNA–protein crosslinks (DPCs) [4, 5] keep proteins covalently trapped. DPCs disrupt genomic integrity and contribute to mutagenesis and carcinogenesis by blocking replication and transcription [6], and they are difficult to repair due to bulky size [3]. DPCs can be induced by endogenous and exogenous sources [3]. Because of numerous intermediates and mechanisms that could be involved, DPCs remain one of the ubiquitous yet least understood forms of DNA damage [7, 8].

Guanosine (G) has the lowest oxidation potential (*E*°) among natural DNA nucleosides, with *E*° versus NHE (normal hydrogen electrode) increasing in the order of 1.29 V for guanosine <1.42 V for adenosine <1.6 V for cytidine <1.7 V for thymidine [9, 10]. Complementary pairing with cytidine in double-stranded DNA further lowers the *E*° of guanosine by 0.28–0.34 V [11, 12]. As a result, guanosine is a primary DNA target for one-electron oxidation upon photoionization [13], ionizing radiation [14], electron transfer with transition metals [15], electrocatalytic oxidation [16], photooxidation [17], etc. Holes generated at other nucleosides also migrate to guanosine sites [18]. Collectively, these factors render the formation of guanosine radical cations (G^•+^) the ultimate trap for oxidatively generated damage to DNA [19]. Formation of G^•+^ is the initial step in a cascade of biological sequelae [20–25]. For instance, G^•+^ serves as a precursor [26] to 8-oxoguanosine (OG)—the most commonly observed DNA lesion [27].

Following the formation of G^•+^ and OG, two mechanisms have been proposed for the formation of DPCs. The first mechanism involves the nucleophilic addition of an amino acid side chain, such as the *ϵ*-NH_2_ of lysine (referred to as LysNH_2_), to the C5 and C8 positions of G^•+^ or its deprotonated counterpart [G – H]^•^, as demonstrated by the groups of Cadet and Ravanat [28–31], Burrows [32, 33], and Stemp [34–36]. The second mechanism is driven by an oxidized OG intermediate, 2-amino-7,9-dihydro-purine-6,8-dione (OG^OX^) [37, 38], which is highly susceptible to nucleophilic addition. Similar to the formation of guanidinohydantoin (Gh) and spiroiminodihydantoin (Sp) through the reaction of OG^OX^ with water [37, 38], OG^OX^-mediated DPCs yield LysNH-Gh and LysNH-Sp, as reported by the groups of Burrows [32, 33, 39–42], Liu [43], and Merion [8].

DPCs were examined in various structural contexts, including nucleobases, oligonucleotides, and single- and double-stranded DNA. LysNH_2_ was selected as the nucleophile due to its high abundance in DNA-coiling proteins (such as histones in eukaryotes [44]) and its proximity to guanosine residues [30, 40]. Depending on the specific DPC mechanisms under investigation, experiments incorporated type I [28–33] or II [8, 32, 33, 41] photooxidation, Ir^4+^/Fe^3+^/Ru^3+^-induced oxidation [32–36, 40–42], the Fenton reaction [8], photoionization [45], or ionizing radiation [44]. These investigations were complemented by calculations of reaction potential energy surfaces conducted by the groups of Schlegel [46–48], Mishra [49], Barone [50], etc., and molecular dynamics simulations performed by the Dumont group [51, 52].

**Scheme 1.**
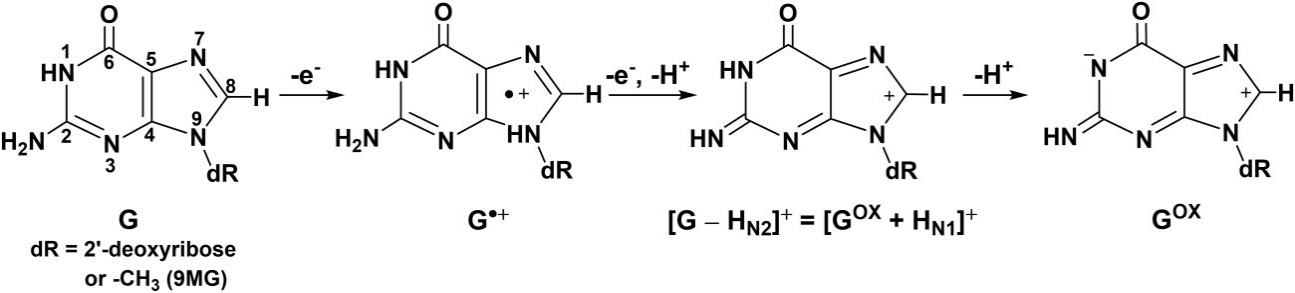
One- and two-electron oxidation of guanosine.

In the context of guanosine oxidation-induced DPCs, the potential for inducing DPCs via the doubly oxidized, non-radical cationic guanosine ([G – H]^+^, Scheme 1) remains largely unexplored [48]. As a quinonoid species, [G – H]^+^ (along with its neutral form G^OX^) is predicted to react with amines and water at its C5 and C8 with minimal or no activation barriers [48, 53]. However, the contribution of [G – H]^+^ to DPCs could not be directly assessed in aqueous experiments, as water effectively competes with LysNH_2_ for addition to [G – H]^+^ [48]. This limitation motivated us to investigate and compare DPCs induced by G^•+^ and [G – H]^+^ in the gas phase. In a rarefied gaseous environment, the complexities and interferences present in solution are largely avoided, and G^•+^ and [G – H]^+^ can persist for lifetimes several orders of magnitudes longer than in aqueous solution. This prolonged timeframe allows us to disentangle underlying processes and reveal intrinsic reactivities. In this sense, gas-phase experiments bridge gaps and resolve missing links in our understanding of DPC mechanisms.

In this work, 9-methylguanine (9MG) was used as a model compound for guanosine with the sugar moiety substituted by a methyl group, and methylamine, with *E*° similar to that of LysNH_2_ [54], was employed as a nucleophile [46, 48]. By generating and isolating 9MG^•+^ and [9MG – H]^+^ in the gas phase, we were able to detect their distinct DPCs with methylamine through mass spectrometry measurements of individual product ions and cross sections. Reaction mechanisms and energetics were then analyzed on the basis of dynamics simulations, density functional theory (DFT) and coupled-cluster theory electronic structure calculations, and kinetics modeling.

## Materials and Methods

### Experimental details

Formation of 9MG^•+^ and [9MG – H]^+^ and their reactions with methylamine were conducted using a home-built tandem mass spectrometer coupled with electrospray ionization [55, 56]. A fresh solution of [Cu^II^(9MG)_1–3_]^•2+^ was prepared by mixing 0.25 mM 9MG (Chemodex, >98%) and 0.25 mM Cu(NO_3_)_2_ (Alfa Aesar, 99.999%) in methanol/water (3:1 *v*/*v*). The solution was electrosprayed into the source chamber of the mass spectrometer via a desolvation capillary heated to 190°C and biased at 130 V relative to ground. A skimmer was positioned 3 mm away from the capillary exit and biased at 85 V. The electric field between the capillary exit and the skimmer facilitated the generation of 9MG^•+^ radical cations via a collision-induced electron-transfer reaction [Cu^II^(9MG)_3_]^•2+^ → [Cu^I^(9MG)_2_]^+^ + 9MG^•+^ [56–60]. [9MG – H]^+^ was produced for the first time via 9MG^•+^ → [9MG – H]^•^ + H^+^ followed by [9MG – H]^•^ + 9MG^•+^ → [9MG – H]^+^ + 9MG (see details in Supplementary Figure S1 and Supplementary Information).

Ions were thermalized to 310 K through collisional cooling and kinetic energy dumping within a radiofrequency hexapole ion guide. Ions of interest were mass selected using a quadrupole mass filter, followed by collimation into an octopole ion guide. The octopole ion guide trapped ions radially and guided them through a scattering cell that encircles the central 11 cm of the octopole, which contained deuterated CD_3_NH_2_ gas (Cambridge Isotope Laboratories, D_3_ 98%). A DC (direct current) bias on the octopole set kinetic energies for reactant ions in the laboratory frame (*E*_lab_), which determined ion-molecule collision energy (*E*_CM_) in the center-of-mass frame using *E*_CM_ = *E*_lab_ × *m*_neutral_ / (*m*_ion_ + *m*_neutral_), where *m*_ion_ and *m*_neutral_ represent the masses of ionic and neutral reactants, respectively. Product ions and unreacted reactant ions were collected by the octopole, passed into a second quadruple mass filter for analysis, and counted by a pulse-counting electron multiplier. The CD_3_NH_2_ gas pressure within the scattering cell was maintained at 0.01–0.015 mTorr to ensure that each reactant ion experienced, at most, a single collision with CD_3_NH_2_. Under these conditions, absolute product cross sections could be calculated using the Beer–Lambert law [55, 56, 61].

Primary ions were generated at intensities of 5 × 10^5^ counts/s for 9MG^•+^ and 2 × 10^3^ counts/s for [9MG – H]^+^. Both first and second quadrupole mass filters used ΔM as a control for mass resolution. The purity of mass-selected reactant ion beam was verified, with no leakage or background ions detected at the M ± 1 positions of the reactant ion. Random background ion was <1 count every 20 s. The full width at half maximum of *E*_lab_ was measured to be ≤0.65 eV using retarding potential analysis [55, 62], corresponding to a spread of ∼ 0.1 eV in nominal *E*_CM_. *E*_CM_ was scanned from 0.05 to 2.0 eV for each reaction to capture complex-mediated mechanism at low energies and direct mechanism at high energies. All measurements were repeated at least three times. The relative uncertainty in cross section measurements was estimated to be below 5%.

For endothermic reactions, threshold energy (*E*_0_) at 0 K was determined by fitting kinetic energy-dependent product ion cross section to a modified line-of-centers (LOC) model [63, 64]: $\sigma ( {{E_{{\mathrm{CM}}}}} ) = {\sigma _0}{({E_{{\mathrm{CM}}}} + {E_{{\mathrm{vib}}}} + {E_{{\mathrm{rot}}}} - {E_0})^n}/{E_{{\mathrm{CM}}}}$, where *σ*_0_ is a normalization factor, *E*_vib_ and *E*_rot_ are reactants vibrational and rotational energies, and *n* is a fitting parameter determining the efficiency of kinetic energy in driving the reaction. To account for the spread of nominal *E*_CM_ and Boltzmann distributions of *E*_vib_ and *E*_rot_, as well as Doppler broadening [65] and reaction kinetic shift [66] in the beam experiment, the LOC model was integrated into an in-house Monte Carlo ion-molecule collision simulation program [67, 68]. *E*_0_ was derived from a simulation that best matched experimental cross section.

### Computations

***(1) Molecular dynamics*:** Quasi-classical direct dynamics trajectory (QCT) simulations were performed using VENUS [69, 70] to set up initial conditions mimicking experiment and using Gaussian 16 [71] to propagate trajectory. Classical equations of motion were solved using a Hessian-based predictor-corrector algorithm [72] with a step size of 0.25 (amu)^1/2^·Bohr (equivalent to 0.4 fsec trajectory time). Hessian was updated every five steps. To balance computational accuracy and efficiency, the ωB97XD [73] functional paired with the 6-31G(d) basis set was chosen for calculations. This functional minimizes self-interaction errors and provides an accurate description of radicals [74, 75], as demonstrated in similar reactions [76, 77]; and the energy accuracy of ωB97XD/6-31G(d) is within 0.1 eV compared to that of ωB97XD/6-31+G(d,p).

Each trajectory began at an ion-neutral distance of 10 Å to ensure no initial interaction between the randomly oriented partners. Reactants were initiated at 300 K by sampling quasi-classical Boltzmann distributions of *E*_vib_ and *E*_rot_ [78]. Relative velocities, aligned with *E*_CM_ and impact parameter *b*, were added to the reactants. The purpose of the simulations was to identify important products, short-lived intermediates, and transition states (TSs). Therefore, trajectories were calculated at *b* = 0.1 Å (rather than randomly sampling *b* within the maximum collision radius) and *E*_CM_ = 0.05 or 0.1 eV to enhance the likelihood of capturing reactive trajectories. The trajectory terminated at 2500 (or 4000) fsec or when products had separated by 10 Å.

***(2) Reaction energetics*:** Structures of reactants, intermediates, TSs, and products were re-optimized at the ωB97XD/6-31+G(d,p) level of theory. Their Cartesian coordinates are provided in Supplementary Information. Vibrational frequencies were computed to confirm stationary structures (no imaginary frequency) and TSs (with only a single imaginary frequency). Intrinsic reaction coordinate was calculated to validate TSs connecting to correct products. For singlet diradicals, broken symmetry unrestricted SCF with “guess = mix” was used to mix HOMO and LUMO and break *α*–*β* and spatial symmetries in initial guesses [79]. Energies of diradicals were spin-purified using Yamaguchi’s approximate spin projection [80–83]: $E = \frac{{\langle{\hat{S}}^{2}\rangle}^{\text{HS}}}{{\langle{\hat{S}}^{2}\rangle}^{\text{HS}}-{\langle{\hat{S}}^{2}\rangle}^{\text{BS}}}E^{\text{BS}}-\frac{{\langle{\hat{S}}^{2}\rangle}^{\text{BS}}}{{\langle{\hat{S}}^{2}\rangle}^{\text{HS}}-{\langle{\hat{S}}^{2}\rangle}^{\text{BS}}}E^{\text{HS}}$, where ${E^{{\mathrm{BS}}}}$ and ${\langle{\hat{S}}^{2}\rangle}^{\text{BS}}$ denote the energy and the expectation value of total spin angular momentum operator for the broken-symmetry singlet, and ${E^{{\mathrm{HS}}}}$ and ${\langle{\hat{S}}^{2}\rangle}^{\text{HS}}$ denote corresponding values for the triplet. As shown in Supplementary Table S6, the degree of spin contamination before projection is minor. All calculated reaction enthalpies (ΔH, 298 K) include zero-point energy (ZPE, scaled by 0.975 [84]) and thermal correction.

Energies of the DFT-optimized structures were cross-checked using the domain-based local pair-natural orbital coupled cluster single-, double-, and perturbative triple excitation method DLPNO-CCSD(T) [85, 86] paired with the aug-cc-pVQZ basis set. The calculations were carried out using ORCA 4.2.1 [87, 88] and results are reported in Supplementary Tables S1–S6. In general, DLPNO-CCSD(T) predicted reaction energetics 0.2ȃ0.3 eV higher than ωB97XD.

***(3) Unimolecular kinetics*:** Rice–Ramsperger–Kessel–Marcus (RRKM) [89] rate constants (*k*) were calculated using the code by Zhu and Hase [90], with the density of states evaluated using a direct count algorithm [91]. Since no reverse barriers exist for crosslinking, orbit TSs [92] were assumed with the reaction orbital angular momentum (*L*) estimated from the collision cross section (*σ*_collision_), i.e. $L = \mu \cdot \nu\cdot\sqrt {{\sigma _{{\rm collision}}}/\pi }$, where *μ* is the reduced mass and *ν* is the relative velocity of collision partners.

## Experimental Results

### Products of 9MG^•+^ with CD_3_NH_2_

The CD_3_NH_2_ isotope combination was used to identify the sources of H abstraction in the products. Hydrogen atom scrambling (i.e. H/D exchange) between reactant ions and -CD_3_ can also provide supplementary evidence for complex mediation in the reaction, in addition to the observation of crosslinking products. The following product channels were detected for 9MG^•+^ (*m*/*z* 165) + CD_3_NH_2_:


\begin{eqnarray*} \begin{array}{l{@}{\quad}l{@}{\quad}l} m/z\,34\!: & {\mathrm{C}}{{\mathrm{D}}_3}{\mathrm{NH}}_2^{ \bullet + } + 9{\mathrm{MG}} & {\mathrm{charge\, transfer = CT}}\\ m/z\,{\mathrm{35\!:}}& {\rm {C}}{{\mathrm{D}}_{\mathrm{3}}}{\mathrm{NH}}_3^{+} + {\mathrm{ [9MG - H}}{{\mathrm{]}}^{\mathrm{ \bullet}}} & {\mathrm{ proton\, transfer}} = {\mathrm{PT}}\\ m/z\,{\mathrm{166\!:}}& {d_{\mathrm{1}}}\textrm{-}{\rm 9M}{{\mathrm{G}}^{ \bullet {\mathrm{ +}}}}+{\mathrm{ CH}}{{\mathrm{D}}_{\mathrm{2}}}{\mathrm{N}}{{\mathrm{H}}_{\mathrm{2}}} & {\mathrm{H}}/{\mathrm{D \,exchange}} = {\mathrm{H}}\_{\mathrm{Ex}}\\ {}&{{\mathrm{[9MG + H]}}^{\mathrm{ +}}}+{\mathrm{ C}}{{\mathrm{D}}_{\mathrm{3}}}{\mathrm{N}}{{\mathrm{H}}^ \bullet }& {\mathrm{ amine\textrm{-}H \,abstraction = HA\_NH_2}}\\ m/z\,167\!:& {\mathrm{[9MG + D}}{{\mathrm{]}}^{\mathrm{ +}}}+{{\mathrm{}}^ \bullet }{\mathrm{C}}{{\mathrm{D}}_{\mathrm{2}}}{\mathrm{N}}{{\mathrm{H}}_{\mathrm{2}}}& {\mathrm{ methyl\textrm{-}H \,abstraction}} = {\mathrm{HA}}\_{\mathrm{CD}}_3\\ m/z\,199\!:& {\mathrm{crosslinking\, adduct}}&{\mathrm{DPC}} \end{array}\end{eqnarray*}

**Figure 1.**
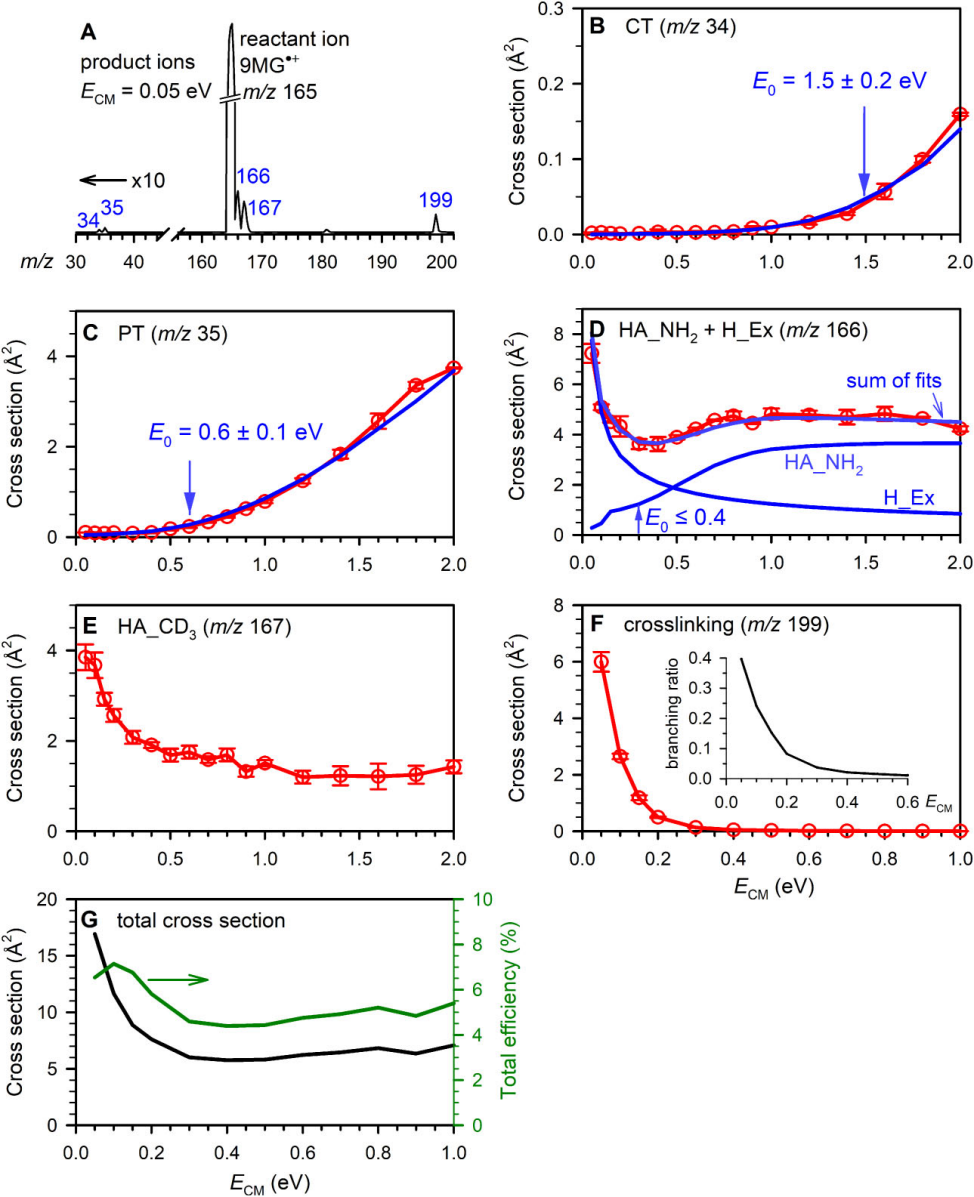
(**A**) Product ion mass spectrum for the reaction of 9MG^•+^ + CD_3_NH_2_, with the intensities of CT and PT product ions scaled by a factor of 10; (**B**–**F**) cross sections for individual product ions as a function of *E*_CM_, where red plots represent experimental data and blue lines represent LOC fits. The inset in panel (**F**) shows the branching ratio for crosslinking; (**G**) total product cross section (left axis) and reaction efficiency (right axis) as a function of *E*_CM_.

A representative product ion mass spectrum, recorded at *E*_CM_ = 0.05 eV, is shown in Fig. 1A. Individual product ion cross sections were measured across an *E*_CM_ range from 0.05 to 2.0 eV and are represented by red traces in Fig. 1B–F. Error bars represent standard deviations. The sum of all product ion cross sections (*σ*_total_) and reaction efficiency are presented in Fig. 1G. The *σ*_total_ reaches a maximum of 17 Å at *E*_CM_ = 0.05 eV, gradually decreases to 6 Å at 0.3 eV, and then nearly levels off at higher energies. Reaction efficiency was calculated as *σ*_total_/*σ*_collision_, where *σ*_collision_ was determined from either the ion-neutral capture cross section using a statistical adiabatic channel model [93] or the hard-sphere collision cross section based on orientation-averaged projected area [94, 95], whichever is greater. Reaction efficiency is maximized (7%) at *E*_CM_ = 0.1 eV, decreases to 5% at 0.3 eV, and remains nearly constant at higher energies. The dip in reaction efficiency at 0.05 eV is an artifact caused by back-scattering of some product ions at the lowest *E*_CM_, leading to their absence from product ion detection.

**(1)** ***CT and PT***: The cross sections for CT and PT present a threshold at low energies and increase monotonically with rising *E*_CM_ (Fig. 1B and C). By accounting for reaction energy broadening and kinetic shift, the blue-line LOC fits successfully reproduced experimental cross sections. From the LOC fits, the 0 K reaction threshold was determined to be 1.5 ± 0.2 eV for CT and 0.6 ± 0.1 eV for PT. The uncertainties in *E*_0_ were estimated from multiple LOC fits over an acceptable range of *n* and included uncertainty in *E*_CM_, with the best fits achieved at *n* = 2.4–2.5.

Using literature-reported adiabatic ionization potentials (AIPs), i.e. AIP(CH_3_NH_2_) = 9.04 eV [96] and AIP(9MG) = 7.63 eV [83], we calculated Δ*H* (0 K) of 1.41 eV for CT. This value agrees well with the experimental *E*_0_. The G4(MP2)-6X-calculated gas-phase acidity for 9MG^•+^ is 10.0 eV for N1H, 9.9 eV for N2H_a_, and 10.1 eV for N2H_b_ [97]. The NIST-listed basicity for CH_3_NH_2_ is 9.32 eV [98]. Combining these data leads to a PT threshold in the range of 0.58–0.78 eV, with the PT from N2H_a_ (with Δ*H* = 0.58 eV) best matching the experiment.

**(2) *H_Ex and HA_NH_2_***: The two reactions yielded the same product ion *m*/*z* 166; however, they could be distinguished through kinetic-energy dependence. The cross section in Fig. 1D consists of two components: the first component reaches a maximum at the lowest *E*_CM_ and decreases gradually with increasing *E*_CM_, which well matches the thermal-neutral H_Ex; while the second component grows at high energies and becomes energy independent at *E*_CM_ = 1.0–2.0 eV, which matches the endothermic HA_NH_2_. To distinguish individual contributions, we modeled the H_Ex cross section using the ion-dipole capture cross section [93] and the HA_NH_2_ cross section using the modified LOC model, matching their sum to the experimental data. The LOC-fitted *E*_CM_-dependence for H_Ex of 9MG^•+^ + CD_3_NH_2_ resembles that for 9MG^•+^ + D_2_O [76]. The LOC fitting for HA_NH_2_ yields a threshold <0.4 eV.

**(3) *HA_CD*_3_**: Opposite to amine-H abstraction, the cross section for hydrogen abstraction from CD_3_ increases with decreasing *E*_CM_ (Fig. 1E), indicating an exothermic pathway with no activation barrier above the reactants. The cross section reaches a maximum of 4 Å^2^ at *E*_CM_ = 0.05 eV, then declines to 2 Å^2^ at 1.2 eV before becoming energy independent at higher *E*_CM_. While the contribution of double H/D exchange to the product ions *m*/*z* 167 is possible, it is unlikely to be significant. This is supported by the fact that double H/D exchange was not observed in the reaction of 9MG^•+^ + D_2_O, which was measured over a similar *E*_CM_ range using the same mass spectrometer [76].

**(4) *Crosslinking***:DPCs represent the most important product channel. It shows sharp *E*_CM_ suppression with a maximum cross section of 6 Å^2^ at 0.05 eV, declining to 1 Å^2^ at 0.15 eV and becoming negligible starting at 0.3 eV (Fig. 1F). This pattern agrees with a reaction governed by a complex. The inset in Fig. 1F illustrates the product branching ratio for crosslinking. It decreases from 0.4 at *E*_CM_ = 0.05 eV to 0.1 at 0.2 eV, eventually approaching near zero at 0.4 eV and beyond. For comparison, the branching ratio for HA_NH_2_ + H_Ex is 0.44 at *E*_CM_ = 0.05–0.1 eV and increases to 0.60 at 0.4 eV. The branching ratio for HA_CD_3_ is 0.24 at *E*_CM_ = 0.05 eV and increases to 0.32–0.35 at 0.1–0.4 eV.

### Products of [9MG – H] ^+^ with CD_3_NH_2_

**Figure 2.**
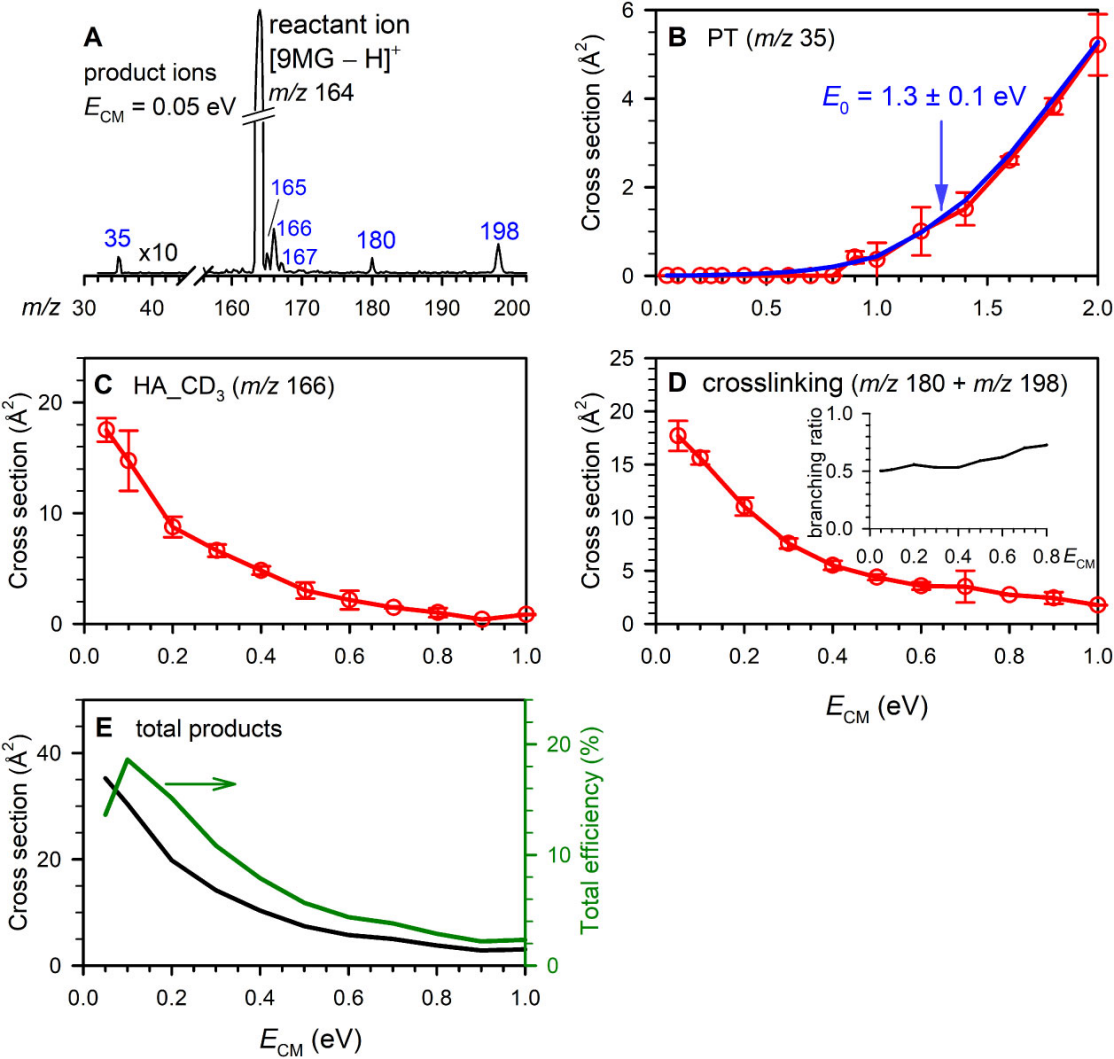
(**A**) A product ion mass spectrum for the reaction of [9MG – H]^+^ + CD_3_NH_2_, with the PT product ion intensities scaled by a factor of 10; (**B**–**D**) cross sections for individual product ions as a function of *E*_CM_, where red plots represent experimental data, and blue line represents a LOC fit. The insert in panel (D) shows the branching ratio for crosslinking; (**E**) total product cross section (left axis) and reaction efficiency (right axis) as a function of *E*_CM_.

This work generated, separated, and measured [9MG – H]^+^ for the first time. The reaction of [9MG – H]^+^ with CD_3_NH_2_ was assessed using a procedure similar to that for 9MG^•+^ + CD_3_NH_2_. The mass spectrum shown in Fig. 2A displays product ions measured at *E*_CM_ = 0.05 eV:


\begin{eqnarray*} \begin{array}{l{@}{\quad}l{@}{\quad}l} m/z\,{\mathrm{35\!:}}&{\mathrm{ C}}{{\mathrm{D}}_{\mathrm{3}}}{\mathrm{N}}{{\mathrm{H}}_{\mathrm{3}}}^{\mathrm{ +}} + {\mathrm{ 9M}}{{\mathrm{G}}^{{\mathrm{OX}}}}& {\mathrm{PT}}\\ m/z\,{\mathrm{165}}\!:& {d_{\mathrm{1}}}{\mathrm{ \textrm{-} [9MG - H}}{{\mathrm{]}}^{\mathrm{ +}}} + {\mathrm{ CH}}{{\mathrm{D}}_{\mathrm{2}}}{\mathrm{N}}{{\mathrm{H}}_{\mathrm{2}}}&{\mathrm{H}}\_{\mathrm{Ex}}\\ {}& {\mathrm{9M}}{{\mathrm{G}}^{{\mathrm{ \bullet +}}}}{\mathrm{ tautomers + C}}{{\mathrm{D}}_{\mathrm{3}}}{\mathrm{N}}{{\mathrm{H}}^{\mathrm{ \bullet}}}& {\mathrm{HA\_NH_{2}}}\\ m/z\,166\!: & {d_{\mathrm{1}}}{\mathrm{ \textrm{-} 9M}}{{\mathrm{G}}^{{\mathrm{ \bullet +}}}}{\mathrm{ tautomers}} + {{\mathrm{ }}^{\mathrm{ \bullet}}}{\mathrm{C}}{{\mathrm{D}}_{\mathrm{2}}}{\mathrm{N}}{{\mathrm{H}}_{\mathrm{2}}}& {\mathrm{HA}}\_{\mathrm{C}}{{\mathrm{D}}_3}\\ m/z\,167\!: & {d_1}{\mathrm{ \textrm{-} [9MG + H}}{{\mathrm{]}}^{\mathrm{ +}}} {\mathrm{ tautomers}} + {\mathrm{ C}}{{\mathrm{D}}_{\mathrm{2}}}{\mathrm{NH}}& {{\mathrm{H}}^\ominus }{\mathrm{A + PT}}\\ m/z\,198\!:&{\mathrm{crosslinking\, adduct}}&{\mathrm{ DPC}}\end{array}\end{eqnarray*}

Many product channels are similar to those observed in the reaction of 9MG^•+^+ CD_3_NH_2_, such as PT, H_Ex, HA_NH_2_, HA_CD_3_, and DPC. Products that are specific to [9MG – H]^+^ include *m*/*z* 167 that corresponds to *d*_1_-[9MG + H]^+^ tautomers formed via sequential hydride abstraction (H^⊝^A) and proton transfer, and *m*/*z* 180 that corresponds to CD_3_ elimination from an adduct (indicating that certain crosslinking pathways release enough energy to eliminate the methyl group). The intensities of *m*/*z* 180 are thus lumped into the crosslinking cross sections. Figure 2B–D presents individual product channels across the *E*_CM_ range of 0.05–2.0 eV, except for *m*/*z* 165 and 167 as their cross sections were too low to make meaningful measurements.

The two major product channels, HA_CD_3_ and crosslinking, are both exothermic, reaching a maximum at the lowest *E*_CM_ and gradually decreasing to a minimum at 1.0 eV. The LOC-fitted *E*_0_ for the PT reaction is 1.3 ± 0.1 eV with *n* = 2.4 (Figure 2B). At first glance, it may seem surprising that CT was absent for [9MG – H]^+^ + CD_3_NH_2_, despite it being only endothermic by 0.6 eV. However, the CT product pair, ^2^[9MG – H]^•^ + ^2^CD_3_NH_2_^•+^, once formed, would readily undergo proton transfer barrierlessly to yield *d*_1_-9MG^•+^ + ^•^CD_2_NH_2_.

Notably, the reaction efficiency for [9MG – H]^+^ with CD_3_NH_2_ reaches 19% at low energy (Fig. 2E), which is more than twice that for 9MG^•+^. Furthermore, the cross section for methyl-H abstraction by [9MG – H]^+^ is five times higher than that by 9MG^•+^. The crosslinking for [9MG – H]^+^ is three times greater than that for 9MG^•+^. The product branching ratio for crosslinking in the [9MG – H]^+^ reaction reaches 0.5 at *E*_CM_ = 0.05 eV and increases to 0.75 at 0.8 eV (while that for HA_CD_3_ decreases from 0.5 at 0.05 eV to 0.25 at 0.8 eV). In contrast, the crosslinking branching ratio in the 9MG^•+^ reaction is only 0.4 at 0.05 eV and rapidly decreases to near zero at 0.6 eV. It suggests that [9MG – H]^+^ is more susceptible to DPCs. It also suggests that the crosslinking of [9MG – H]^+^ involves complex-mediated pathways at low energies and direct addition at high energies.

## Molecular Dynamics Trajectory Results

To probe the origin of the DPC enhancement by [9MG – H]^+^, dynamics simulations were utilized to mimic the crosslinking of CH_3_NH_2_ with 9MG^•+^ versus [9MG – H]^+^. Direct dynamics simulations [99] do not require a predefined potential surface. Instead, trajectories calculate energies, force constants, and Hessians “on the fly.” This approach allows for the inclusion of all energetically accessible reacting structures, so that trajectories can reveal preferred pathways. A total of 800 trajectories were calculated for 9MG^•+^ + CH_3_NH_2_ and 400 trajectories for [9MG – H]^+^ + CH_3_NH_2_. All trajectories were simulated at 0.05 or 0.1 eV to exclude endothermic CT and PT, as they are not directly relevant to DPCs.

### 9MG^•+^ + CH_3_NH_2_

**Figure 3.**
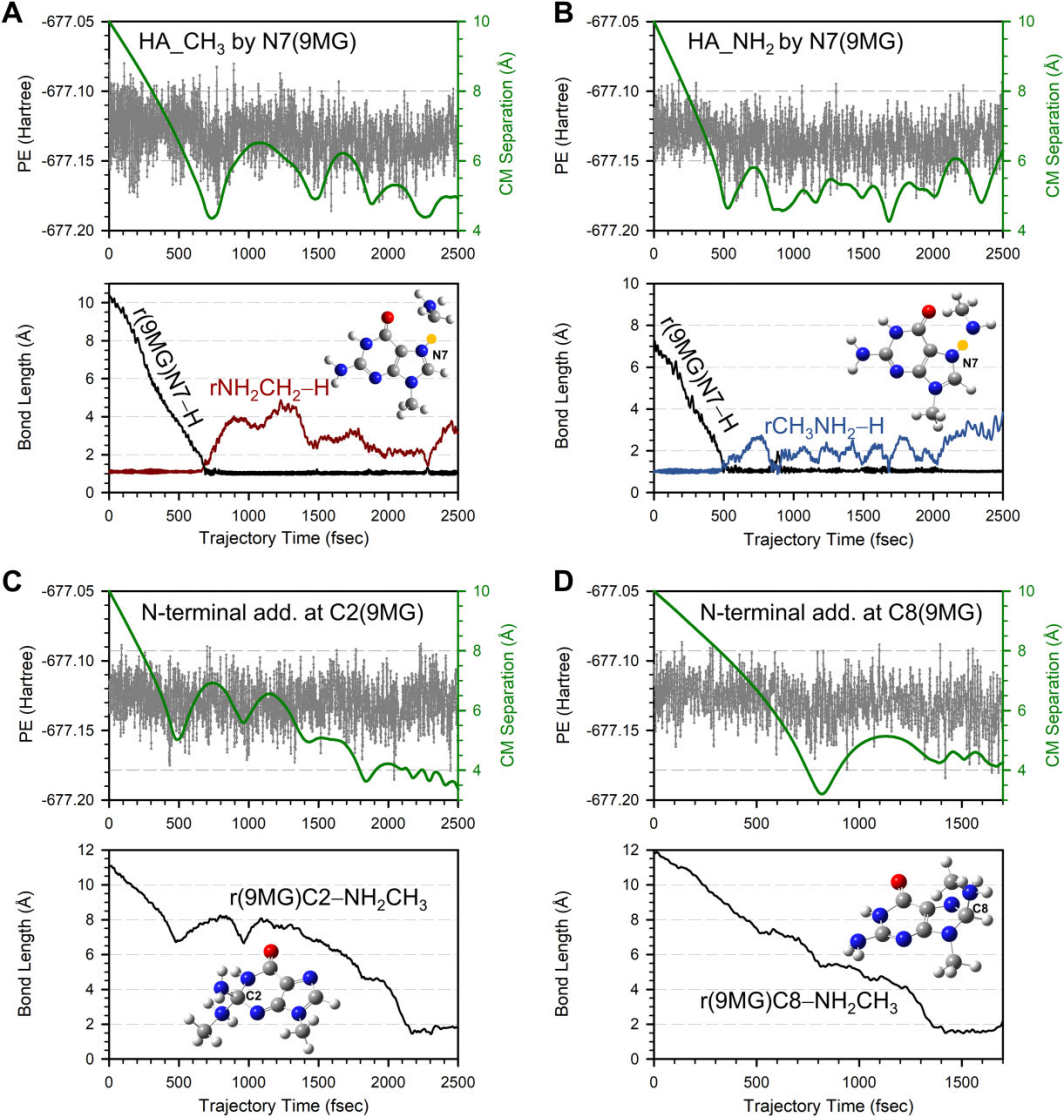
Trajectories for the reaction of 9MG^•+^ + CH_3_NH_2_ simulated at *E*_CM_ = 0.05 eV, showing (**A**) methyl-H abstraction, (**B**) amine-H abstraction, (**C**) N-terminal addition of CH_3_NH_2_ to C2 in 9MG^•+^, and (**D**) N-terminal addition of CH_3_NH_2_ to C8 in 9MG^•+^. Each top frame depicts changes of potential energy (left axis) and center-of-mass reactant/product separation (right axis), while the bottom frame shows reactive bond lengths throughout the trajectory. Inset snapshots illustrate product structures, with the abstracting H highlighted in yellow.

Four reactive trajectory pathways were identified for 9MG^•+^ + CH_3_NH_2_. These include HA_CH_3_, HA_NH_2_, N-terminal addition of CH_3_NH_2_ to the C2 of 9MG^•+^, and N-terminal addition of CH_3_NH_2_ to the C8 of 9MG^•+^. Fig. 3 illustrates individual pathways. For each trajectory, the top frame presents changes in reaction potential energy (PE, left axis) and center-of-mass reactant/product distance (CM separation, right axis) throughout the trajectory, and the bottom frame presents variations of reactive bond lengths. The high-frequency oscillations in PEs and bond lengths reflect vibrations and rotations of reactants and products. The first turning point in the CM separation marks the time at which the ion and molecule start to collide, which often introduces a change in PE.

**(1) *Hydrogen abstraction pathways***: HA_CH_3_ represents the most common trajectory outcome with a probability of 10 ± 1%. As shown in Fig. 3A, methyl-H abstraction [defined as point when the new r(9MG)N7-H bond forms] occurs immediately upon collision. Subsequently, a product-like complex forms, as indicated by the oscillation of the product CM distance around 5 Å. This complex does not maintain a well-defined geometry but undergoes significant intermolecular motion, as evidenced by multiple turning points in the relative motion of the product CM. Although the simulation lasted only 2500 fsec, the actual ion time-of-flight within the mass spectrometer was around 10^2^ μs. This extended time allowed the H-abstraction product to repeatedly encounter and find an optimal orientation for a subsequent addition reaction. A similar phenomenon occurred in the HA_NH_2_ trajectory (Fig. 3B), despite this being a minor channel with a trajectory probability of 1 ± 0.4%.

**(2) *Direct addition pathways***: The C2- and C8-addition of CH_3_NH_2_ account for 1 ± 0.4% and 2 ± 0.5% of all trajectories, respectively. The time required for addition varies depending on the initial collision orientation, e.g. the formation of the 2-NH_2_CH_3_[9MG]^•+^ adduct (Fig. 3C) occurs 1700 fsec after the collision, while the formation of 8-NH_2_CH_3_[9MG]^•+^ (Fig. 3D) occurs 600 fsec after the collision.

### [9MG – H]^+^ + CH_3_NH_2_

**Figure 4.**
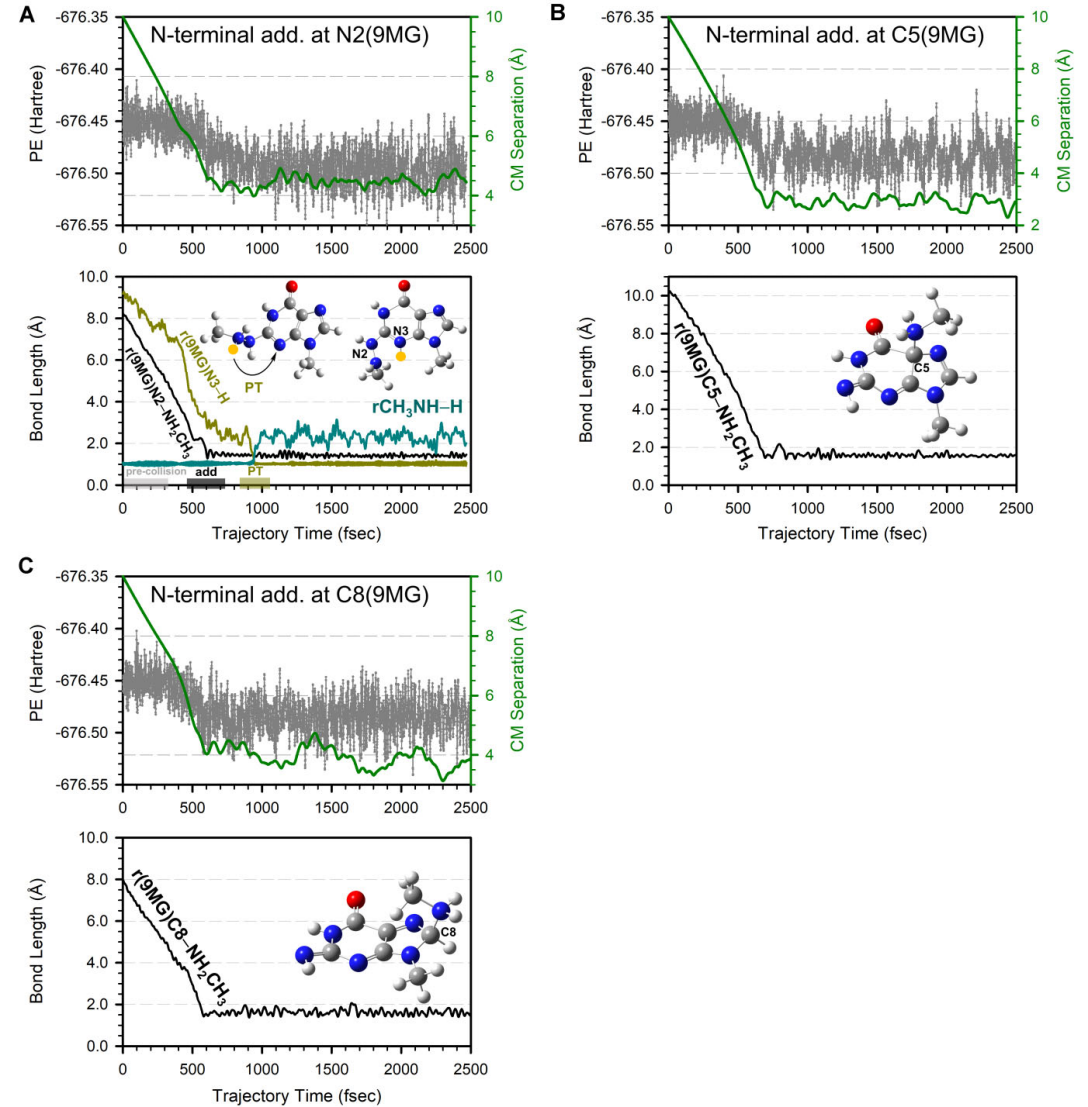
Trajectories for direct addition of [9MG – H]^+^ + CH_3_NH_2_ simulated at *E*_CM_ = 0.1 eV, leading to the formation of (**A**) 2-NHCH_3_[9MG – H_N2_+ H_N3_]^+^, (**B**) 5-^+^NH_2_CH_3_[9MG – H_N2_], and (**C**) 8-^+^NH_2_CH_3_[9MG – H_N2_]. Each top frame depicts the changes of potential energy (left axis) and center-of-mass reactant/product separation (right axis), while the bottom frame shows reactive bond lengths throughout the trajectory. Inset snapshots illustrate product structures, with PT highlighted in yellow.

**(1) *Direct addition pathways***: Trajectories for [9MG – H]^+^ + CH_3_NH_2_ are dominated by the formation of 2-^+^NH_2_CH_3_[9MG – H_N2_] (followed by proton tautomerization to 2-NHCH_3_[9MG – H_N2_ + H_N3_]^+^, Fig. 4A, yield = 3 ± 0.9%), 5-^+^NH_2_CH_3_[9MG – H_N2_] (Fig. 4B, 24 ± 2%), and 8-^+^NH_2_CH_3_[9MG – H_N2_] (Fig. 4C, 37 ± 2%). Unlike the addition of 9MG^•+^ + CH_3_NH_2_ which occurs significantly later after the collision, the addition of CH_3_NH_2_ to [9MG – H]^+^ takes place immediately upon collision, as seen from the CM separation and the rapid formation of new bonds. A significant decrease in PE upon addition indicates that the process is highly exothermic.

**Figure 5.**
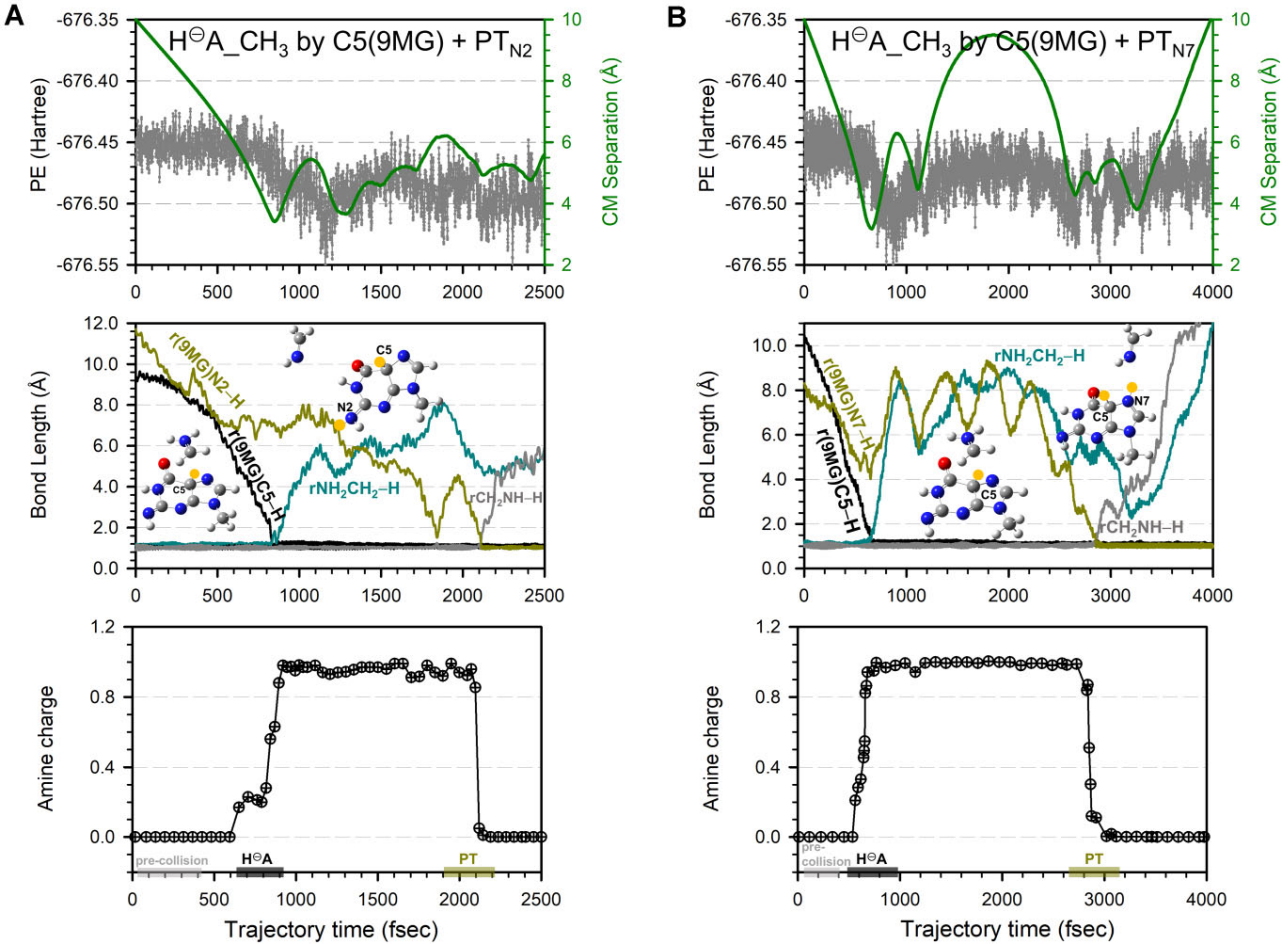
Trajectories for sequential hydride abstraction and PT of [9MG – H]^+^ + CH_3_NH_2_ simulated at *E*_CM_ = 0.05 eV, leading to the formation of (**A**) [9MG + H_C5_]^+^ + CH_2_NH and (**B**) [9MG – H_N2_ + H_C5_+ H_N7_]^+^ + CH_2_NH. Each top frame depicts the changes of potential energy (left axis) and center-of-mass reactant/product separation (right axis), the middle frame shows reactive bond lengths, and the bottom frame shows the charge of the amine moiety throughout the trajectory. Inset snapshots illustrate product structures, with H^⊝^A and PT highlighted in yellow.

**(2)** ***Sequential methyl hydride abstraction (H^⊝^A_CH_3_) and PT***: This pathway accounts for a small fraction (2 ± 0.7%) of the trajectories. As exemplified in Fig. 5A–B, the C5 of [9MG – H]^+^ abstracts a hydride anion from the methyl group of CH_3_NH_2_, followed by PT from CH_2_^+^NH_2_ to the N2 or N7. Hydride abstraction and PT are separated by 650–850 fsec in the trajectory and are distinguished by accompanying charge transfer. The final products contribute to the *m*/*z* 167 peak in Fig. 2A.

In summary, the trajectories successfully reproduced all experimental products and their relative importance, except for the open-shell H abstraction for [9MG – H]^+^ + CH_3_NH_2_, as the QCT trajectories remained in the initial closed-shell singlet state. Moreover, the trajectories correctly predicted a significantly higher overall reactivity and crosslinking yield of [9MG – H]^+^ compared to 9MG^•+^. This indicates that direct dynamics simulations effectively captured key reaction dynamics and outcomes and can therefore be used to extract mechanistic insights.

## Analysis of Crosslinking Pathways and Kinetics

Guided by trajectory results, reaction coordinates were constructed for all probable pathways at the ωB97XD/6-31+G(d,p) level of theory. To complete a comprehensive and reliable kinetics analysis, we included not only primary intermediates and products in the calculations but also downstream conversions.

### 9MG^•+^ mediates DPCs with CH_3_NH_2_ primarily by methyl-H abstraction and direct addition

**Scheme 2.**
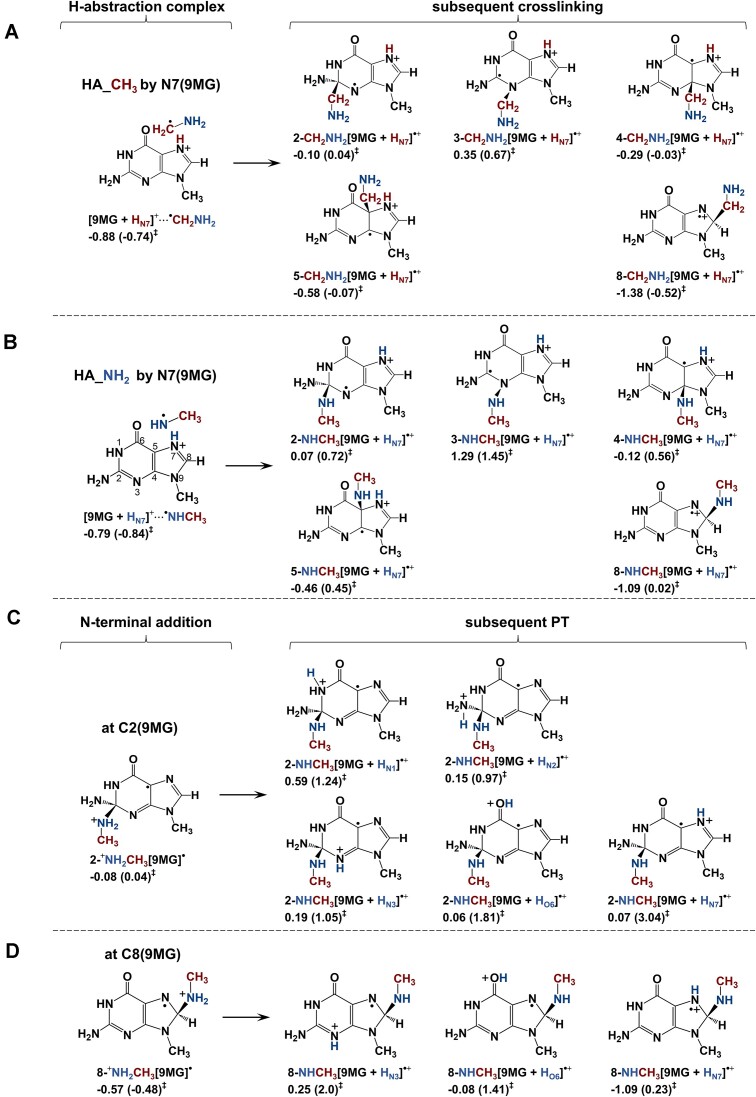
Most probable reaction pathways for 9MG^•+^ + CH_3_NH_2_ and subsequent crosslinking. Reaction enthalpies (eV) and activation barriers (in parentheses) were calculated at ωB97XD/6-31+G(d,p).

**(1) *Intermediacy of HA_CH_3_***: The dominance of HA_CH_3_ in both experiment and trajectories motivated us to investigate all possible methyl-H abstraction pathways. The results are summarized in Scheme 2A and Supplementary Scheme S1, and reactions (1.1–1.3). Given that methyl-H abstraction was measured to be exothermic, only reaction (1.1) is important.


\begin{eqnarray*} {\mathrm{9M}}{{\mathrm{G}}^{{\mathrm{ \bullet +}}}}+{\mathrm{C}}{{\mathrm{H}}_{\mathrm{3}}}{\mathrm{N}}{{\mathrm{H}}_{\mathrm{2}}} \to \end{eqnarray*}

(1.1) \begin{eqnarray*} [9{\mathrm{MG+H}}_{\mathrm{N7}}]^{+} {\cdots}^{\bullet}{\mathrm{CH}}_{2} {\mathrm{NH}}_{2}\quad \Delta {\mathrm{H = - 0.88\, eV, TS = - 0.74\, eV}} \to [9{\mathrm{MG+H}}_{\mathrm{N7}}]^{+} + {}^{\bullet} {\mathrm{CH}}_{2}{\mathrm{NH}}_{2}\quad\Delta {\mathrm{H = - 0.14\, eV}} \end{eqnarray*}

(1.2) \begin{eqnarray*} [9{\mathrm{MG + H}}_{\mathrm{O6}}]^{+} {\cdots}^{\bullet}{\mathrm{CH}}_{2} {\mathrm{NH}}_{2} \quad\Delta {\mathrm{H = - 0.69\, eV, TS = - 0.71\, eV}} \to [9{\mathrm{MG + H}}_{\mathrm{O6}}]^{+}+ {}^{\bullet}{\mathrm{CH}}_{2} {\mathrm{NH}}_{2}\quad \Delta {\mathrm{H = 0.18\, eV}} \end{eqnarray*}

(1.3) \begin{eqnarray*} [9{\mathrm{MG + H}}_{\mathrm{N3}}]^{+} {\cdots}^{\bullet}{\mathrm{CH}}_{2}{\mathrm{NH}}_{2}\quad\Delta {\mathrm{H = 0.02\, eV, TS = 0.22\, eV}} \to [9{\mathrm{MG + H}}_{\mathrm{N3}}]^{+}+ {}^{\bullet}{\mathrm{CH}}_{2} {\mathrm{NH}}_{2}\quad \Delta {\mathrm{H = 0.66\, eV}} \end{eqnarray*}

Since *d*_3_-methylamine was used in the experiment, the kinetic isotope effect (KIE) was evaluated. Using RRKM theory, the *k*_H_ and *k*_D_ for reaction (1.1) were calculated, yielding a *k*_H_/*k*_D_ ratio of 4 and 5 in the *E*_CM_ range of 0.05–0.3 eV. This indicates that methyl-H abstraction is significantly suppressed by deuteration. We also calculated tunneling effect for methyl-H abstraction [100]. TS imaginary frequency for reaction (1.1) is 169 cm^−1^ with CH_3_NH_2_ and 140 cm^−1^ with CD_3_NH_2_. Consequently, the Wigner tunneling factor [101], calculated as $[ {1 + \frac{1}{{24}}{{( {\frac{{{\mathrm{h}}\upsilon}}{{{k_{\mathrm{B}}}{\mathrm{T}}}}} )}^2}} ]$, is 1.03 for CH_3_NH_2_ and 1.02 for CD_3_NH_2_, indicating that tunneling can be neglected.

As implied by the trajectory results, the [9MG + H_N7_]^+^⋅⋅⋅^•^CH_2_NH_2_ complex may ultimately rearrange to form various DPCs. These possibilities are explored in Scheme 2A, of which the conversion to 8-CH_2_NH_2_[9MG + H_N7_]^•+^ is the most appreciable. That is, *k* is 8 × 10^8^ s^−1^ at *E*_CM_ = 0.05 eV and increases to 3 × 10^9^ s^−1^ at 0.3 eV. It indicates that crosslinking following methyl-H abstraction is highly effective.

**(2) *Intermediacy of HA_NH_2_***: Probable amine-H abstraction pathways are presented in Scheme 2B and Supplementary Scheme S2. According to DFT calculations, the N7, O6, and N3 of 9MG^•+^ all exhibit amine-H abstraction capability, leading to the formation of a product-like complex and/or separated products as listed in reactions (2.1–2.3). Note that the electronic energy for the H-abstraction TS in reactions (1.2) and (2.1–2.2) is only slightly higher than that of the corresponding product-like complex. Since the TS imaginary frequency is excluded in the thermal correction to the TS energy, the enthalpy of the TS falls below that of the complex.


\begin{eqnarray*} {\mathrm{9M}}{{\mathrm{G}}^{{\mathrm{ \bullet +}}}} + {\mathrm{ C}}{{\mathrm{H}}_{\mathrm{3}}}{\mathrm{N}}{{\mathrm{H}}_{\mathrm{2}}} \to \end{eqnarray*}

(2.1) \begin{eqnarray*} &&{{\mathrm{[9MG}} + {{\mathrm{H}}_{{\mathrm{N7}}}}{\mathrm{]}}^{\mathrm{ +}}}{ \cdots ^{\mathrm{ \bullet}}}{\mathrm{NHC}}{{\mathrm{H}}_{\mathrm{3}}} \quad\Delta {\mathrm{H = - 0}}{\mathrm{.79\, eV, TS = - 0}}{\mathrm{.84\, eV}} \to {{\mathrm{[9MG }} +{{\mathrm{H}}_{{\mathrm{N7}}}}{\mathrm{]}}^{\mathrm{ +}}}+ {}^{\bullet}{\mathrm{NHC}}{{\mathrm{H}}_{\mathrm{3}}} \quad\Delta {\mathrm{H = 0}}{\mathrm{.14\, eV}}\end{eqnarray*}

(2.2) \begin{eqnarray*} &&{{\mathrm{[9MG }} +{{\mathrm{H}}_{{\mathrm{O6}}}}{\mathrm{]}}^{\mathrm{ +}}}{ \cdots ^{\mathrm{ \bullet}}}{\mathrm{NHC}}{{\mathrm{H}}_{\mathrm{3}}} \quad\Delta {\mathrm{H = - 0}}{\mathrm{.21\, eV, TS = - 0}}{\mathrm{.27\, eV}} \to {{\mathrm{[9MG }} +{{\mathrm{H}}_{{\mathrm{O6}}}}{\mathrm{]}}^{\mathrm{ +}}}+ {}^{\bullet}{\mathrm{NHC}}{{\mathrm{H}}_{\mathrm{3}}} \quad\Delta {\mathrm{H = 0}}{\mathrm{.47\, eV}}\end{eqnarray*}

(2.3) \begin{eqnarray*} &&{{\mathrm{[9MG }} +{{\mathrm{H}}_{{\mathrm{N3}}}}{\mathrm{]}}^{\mathrm{ +}}}{ \cdots ^{\mathrm{ \bullet}}}{\mathrm{NHC}}{{\mathrm{H}}_{\mathrm{3}}} \quad\Delta {\mathrm{H = 0}}{\mathrm{.03\, eV, TS = 0}}{\mathrm{.31\, eV}} \to {{\mathrm{[9MG }} +{{\mathrm{H}}_{{\mathrm{N3}}}}{\mathrm{]}}^{\mathrm{ +}}}+ {}^{\bullet}{\mathrm{NHC}}{{\mathrm{H}}_{\mathrm{3}}} \quad\Delta {\mathrm{H = 0}}{\mathrm{.95\, eV}}\end{eqnarray*}

Consistent with the trajectory results, the DFT calculations reveal that only H abstraction by N7, i.e. reaction (2.1), matches the experimental HA_NH_2_ threshold (Fig. 1D), confirming its relevance. The TS has an imaginary frequency of 857 cm^−1^ and a Wigner tunneling factor of 1.7, indicating significant H tunneling.

Scheme 2B also illustrates the covalent addition of ^•^NHCH_3_ to [9MG + H_N7_]^+^ within the product-like complex [9MG + H_N7_]^+^⋅⋅⋅^•^NHCH_3_. Most addition pathways involve a high barrier and/or yield an endothermic product. Only the formation of 8-NHCH_3_[9MG + H_N7_]^•+^ exhibits exothermicity (−1.09 eV) and a near-thermal barrier (0.02 eV), making it a likely contributor to crosslinking.

Our results highlight the critical role of N7-mediated methyl- and amine-H abstraction in DPC formation. This is supported by the observed reduction of DPCs in N7-capped guanosine [102]. Since methyl-H abstraction dominates over amine-H abstraction at low energies, it is more pertinent to DPCs.

**(3) *C2- and C8-addition***: The addition of CH_3_NH_2_ to the guanine C2 and C8 was calculated in reactions (3.1–3.2) and illustrated in Scheme 2C and D. The C8-addition represents a previously proposed mechanism for G^•+^-induced DPCs [28–36]. While the resulting adducts may undergo proton transfer to form tautomers [48], all tautomerization processes require substantial barriers and can be disregarded at low energies.


\begin{eqnarray*} {\mathrm{9M}}{{\mathrm{G}}^{{\mathrm{ \bullet +}}}} + {\mathrm{ C}}{{\mathrm{H}}_{\mathrm{3}}}{\mathrm{N}}{{\mathrm{H}}_{\mathrm{2}}} \to \end{eqnarray*}

(3.1) \begin{eqnarray*} &&{\mathrm{2}}{{-}^{\mathrm{ +}}}{\mathrm{N}}{{\mathrm{H}}_{\mathrm{2}}}{\mathrm{C}}{{\mathrm{H}}_{\mathrm{3}}}{{\mathrm{[9MG]}}^{\mathrm{ \bullet}}}\quad \Delta {\mathrm{H = - 0}}{\mathrm{.08\, eV, TS = - 0}}{\mathrm{.04\, eV}}\end{eqnarray*}

(3.2) \begin{eqnarray*} &&{\mathrm{8}}{{-}^{\mathrm{ +}}}{\mathrm{N}}{{\mathrm{H}}_{\mathrm{2}}}{\mathrm{C}}{{\mathrm{H}}_{\mathrm{3}}}{{\mathrm{[9MG]}}^{\mathrm{ \bullet}}}\quad \Delta {\mathrm{H = - 0}}{\mathrm{.57\, eV, TS = - 0}}{\mathrm{.48\, eV}}\end{eqnarray*}

### [9MG – H]^+^ enhances DPCs via combination of direct addition, and abstraction of H and H^⊝^

**Scheme 3.**
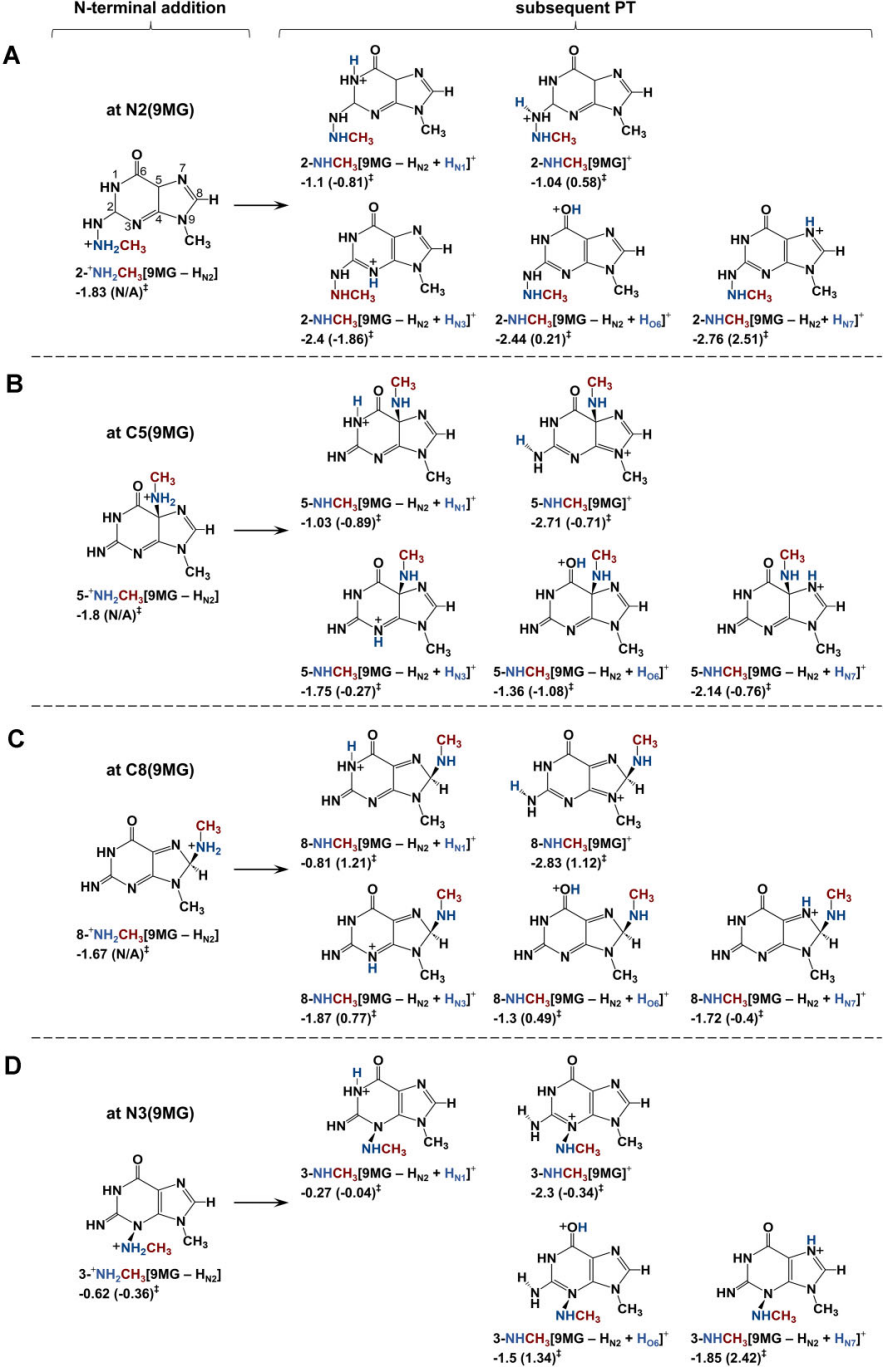
Barrierless direct addition pathways for [9MG – H]^+^ + CH_3_NH_2_ and subsequent proton tautomerization. Reaction enthalpies (eV) and activation barriers (in parentheses) were calculated at ωB97XD/6-31+G(d,p).

**(1) *N2-*, *C5-*, *C8-*, *and N3-addition***: Trajectory-predicted N2-, C5-, and C8-addition pathways were calculated in Scheme 3A–C and reactions (4.1–4.3), showcasing both primary adducts and downstream proton tautomerization products. The DFT calculations also reveal an exothermic N3 adduct, as presented in Scheme 3D and reaction (4.4). Additionally, probable O6- and N7-adducts for [9MG – H_N2_]^+^:NH_2_CH_3_ were explored in reactions (4.5–4.6) and Supplementary Scheme S3. However, both reactions involve activation barriers above reactants and are likely relevant only at high energies.


\begin{eqnarray*} &&{{\mathrm{[9MG}} - {{\mathrm{H}}_{{\mathrm{N2}}}}{\mathrm{]}}^{\mathrm{ +}}} + {\mathrm{ C}}{{\mathrm{H}}_{\mathrm{3}}}{\mathrm{N}}{{\mathrm{H}}_{\mathrm{2}}} \to \end{eqnarray*}

(4.1) \begin{eqnarray*} \begin{array}{l}{\mathrm{2}}{{-}^{\mathrm{ +}}}{\mathrm{N}}{{\mathrm{H}}_{\mathrm{2}}}{\mathrm{C}}{{\mathrm{H}}_{\mathrm{3}}}{\mathrm{[9MG }} - {{\mathrm{H}}_{{\mathrm{N2}}}}{\mathrm{]}}\quad\Delta {\mathrm{H = - 1}}{\mathrm{.83\, eV}} \to 2{\mathrm{-NHC}}{\mathrm{H}}_{3}[9{\mathrm{MG}} - {\mathrm{H}}_{\mathrm{N2}} + {\mathrm{H}}_{\mathrm{N3}}]^{+} \quad\Delta {\mathrm{H = - 2}}{\mathrm{.40\, eV, TS = - 1}}{\mathrm{.86\, eV}}\end{array} \end{eqnarray*}

(4.2) \begin{eqnarray*} \begin{array}{l}{\mathrm{5}}{{-}^{\mathrm{ +}}}{\mathrm{N}}{{\mathrm{H}}_{\mathrm{2}}}{\mathrm{C}}{{\mathrm{H}}_{\mathrm{3}}}{\mathrm{[9MG }} \,-\, {{\mathrm{H}}_{{\mathrm{N2}}}}{\mathrm{]}}\quad\Delta {\mathrm{H = - 1}}{\mathrm{.80\, eV}}\end{array}\end{eqnarray*}

(4.3) \begin{eqnarray*} \begin{array}{l}{\mathrm{8}}{{-}^{\mathrm{ +}}}{\mathrm{N}}{{\mathrm{H}}_{\mathrm{2}}}{\mathrm{C}}{{\mathrm{H}}_{\mathrm{3}}}{\mathrm{[9MG }} \,-\, {{\mathrm{H}}_{{\mathrm{N2}}}}{\mathrm{]}}\quad\Delta {\mathrm{H = - 1}}{\mathrm{.67\, eV}}\end{array}\end{eqnarray*}

(4.4) \begin{eqnarray*} \begin{array}{l}{\mathrm{3}}{{-}^{\mathrm{ +}}}{\mathrm{N}}{{\mathrm{H}}_{\mathrm{2}}}{\mathrm{C}}{{\mathrm{H}}_{\mathrm{3}}}{\mathrm{[9MG }} \,-\, {{\mathrm{H}}_{{\mathrm{N2}}}}{\mathrm{]}}\quad\Delta {\mathrm{H = - 0}}{\mathrm{.62\, eV, TS = - 0}}{\mathrm{.36\, eV}}\end{array}\end{eqnarray*}

(4.5) \begin{eqnarray*} \begin{array}{l}{\mathrm{6}}{{-}^{\mathrm{ +}}}{\mathrm{N}}{{\mathrm{H}}_{\mathrm{2}}}{\mathrm{C}}{{\mathrm{H}}_{\mathrm{3}}}{\mathrm{[9MG }} \,-\, {{\mathrm{H}}_{{\mathrm{N2}}}}{\mathrm{]}}\quad\Delta {\mathrm{H = 0}}{\mathrm{.10\, eV, TS = 0}}{\mathrm{.77\, eV}}\end{array}\end{eqnarray*}

(4.6) \begin{eqnarray*} \begin{array}{l}{\mathrm{7}{-NHC}}{{\mathrm{H}}_{\mathrm{3}}}{{\mathrm{[9MG}} \,-\, {{\mathrm{H}}_{{\mathrm{N2}}}} + {{\mathrm{H}}_{{\mathrm{O6}}}}{\mathrm{]}}^{\mathrm{ +}}} \quad\Delta {\mathrm{H = - 1}}{\mathrm{.48\, eV, TS = 0}}{\mathrm{.34\, eV}}\end{array}\end{eqnarray*}

As shown in the trajectories, primary adducts can undergo proton tautomerization. For example, 2-^+^NH_2_CH_3_[9MG – H_N2_] overwhelmingly converts to 2-NHCH_3_[9MG – H_N2_ + H_N3_]^+^ in reaction (4.1), with a rate constant of 10^12^ s^−1^ at *E*_CM_ = 0.05–0.3 eV. This tautomerization occurs within 350 fsec in the trajectory (Fig. 4A). As a result, 2-NHCH_3_[9MG – H_N2_ + H_N3_]^+^ emerges as the most stable C2-adduct.

**(2) *Methyl hydride abstraction enhances DPCs***:The sequential “methyl hydride abstraction + PT” occurs not only via reaction (5.1) as predicted by the trajectories, but also via reactions (5.2–5.3) as suggested by the DFT calculations, all of which are summarized in Supplementary Scheme S4.


\begin{eqnarray*}{{\mathrm{[9MG }} \,-\, {{\mathrm{H}}_{{\mathrm{N2}}}}{\mathrm{]}}^{\mathrm{ +}}} + {\mathrm{ C}}{{\mathrm{H}}_{\mathrm{3}}}{\mathrm{N}}{{\mathrm{H}}_{\mathrm{2}}} \to \end{eqnarray*}

(5.1) \begin{eqnarray*} {\mathrm{[9MG }} \,-\, {{\mathrm{H}}_{{\mathrm{N2}}}} + {{\mathrm{H}}_{{\mathrm{C5}}}}{\mathrm{]}} \cdots {\mathrm{C}}{{\mathrm{H}}_{\mathrm{2}}}^{\mathrm{ +}}{\mathrm{N}}{{\mathrm{H}}_{\mathrm{2}}} \quad\Delta {\mathrm{H = - 2}}{\mathrm{.39\, eV, TS = - 0}}{\mathrm{.47\, eV}} & \to & {{\mathrm{[9MG }} +{{\mathrm{H}}_{{\mathrm{C5}}}}{\mathrm{]}}^{\mathrm{ +}}}{\mathrm{\,or\,[9MG }} \,-\, {{\mathrm{H}}_{{\mathrm{N2}}}} + {{\mathrm{H}}_{{\mathrm{C5}}}} + {{\mathrm{H}}_{{\mathrm{N7}}}}{{\mathrm{]}}^{\mathrm{ +}}}\nonumber\\ &&+\, {\mathrm{C}}{{\mathrm{H}}_{\mathrm{2}}}{\mathrm{NH}} \quad\Delta {\mathrm{H = - 1}}{\mathrm{.83/ {-}1.18\, eV}}\end{eqnarray*}

(5.2) \begin{eqnarray*} {\mathrm{[9MG }} \,-\, {{\mathrm{H}}_{{\mathrm{N2}}}} + {{\mathrm{H}}_{{\mathrm{O6}}}}{\mathrm{]}} \cdots {\mathrm{C}}{{\mathrm{H}}_{\mathrm{2}}}^{\mathrm{ +}}{\mathrm{N}}{{\mathrm{H}}_{\mathrm{2}}} \quad\Delta {\mathrm{H = - 2}}{\mathrm{.30\, eV, TS = 0}}{\mathrm{.32\, eV}} & \to &{{\mathrm{[9MG }} \,-\, {{\mathrm{H}}_{{\mathrm{N2}}}} + {{\mathrm{H}}_{{\mathrm{O6}}}} + {{\mathrm{H}}_{{\mathrm{N7}}}}{\mathrm{]}}^{\mathrm{ +}}} + {\mathrm{C}}{{\mathrm{H}}_{\mathrm{2}}}{\mathrm{NH}}\nonumber\\ &&\quad\Delta {\mathrm{H = - 1}}{\mathrm{.19\, eV}}\end{eqnarray*}

(5.3) \begin{eqnarray*} {\mathrm{[9MG }} \,-\, {{\mathrm{H}}_{{\mathrm{N2}}}} + {{\mathrm{H}}_{{\mathrm{N7}}}}{\mathrm{]}} \cdots {\mathrm{C}}{{\mathrm{H}}_{\mathrm{2}}}^{\mathrm{ +}}{\mathrm{N}}{{\mathrm{H}}_{\mathrm{2}}} \quad\Delta {\mathrm{H = - 2}}{\mathrm{.30\, eV, TS = 0}}{\mathrm{.18\, eV}} &\to & {{\mathrm{[9MG }} \,-\, {{\mathrm{H}}_{{\mathrm{N2}}}} + {{\mathrm{H}}_{{\mathrm{O6}}}} + {{\mathrm{H}}_{{\mathrm{N7}}}}{\mathrm{]}}^{\mathrm{ +}}} + {\mathrm{ C}}{{\mathrm{H}}_{\mathrm{2}}}{\mathrm{NH}} \nonumber\\ &&\quad\Delta {\mathrm{H = - 1}}{\mathrm{.19\, eV}}\end{eqnarray*}

(5.4) \begin{eqnarray*} &&{\mathrm{9MG}}{ \cdots ^{\mathrm{ +}}}{\mathrm{N}}{{\mathrm{H}}_{\mathrm{2}}}{\mathrm{C}}{{\mathrm{H}}_{\mathrm{2}}} \quad\Delta {\mathrm{H = - 3}}{\mathrm{.96\, eV, TS = - 0}}{\mathrm{.40\, eV}}\end{eqnarray*}

(5.5) \begin{eqnarray*} &&{\mathrm{[9MG }} \,-\, {{\mathrm{H}}_{{\mathrm{N2}}}} + {{\mathrm{H}}_{{\mathrm{N3}}}}{\mathrm{]}}{ \cdots ^{\mathrm{ +}}}{\mathrm{N}}{{\mathrm{H}}_{\mathrm{2}}}{\mathrm{C}}{{\mathrm{H}}_{\mathrm{2}}} \quad\Delta {\mathrm{H = - 3}}{\mathrm{.20\, eV, TS = - 0}}{\mathrm{.38\, eV}}\end{eqnarray*}

**Scheme 4.**
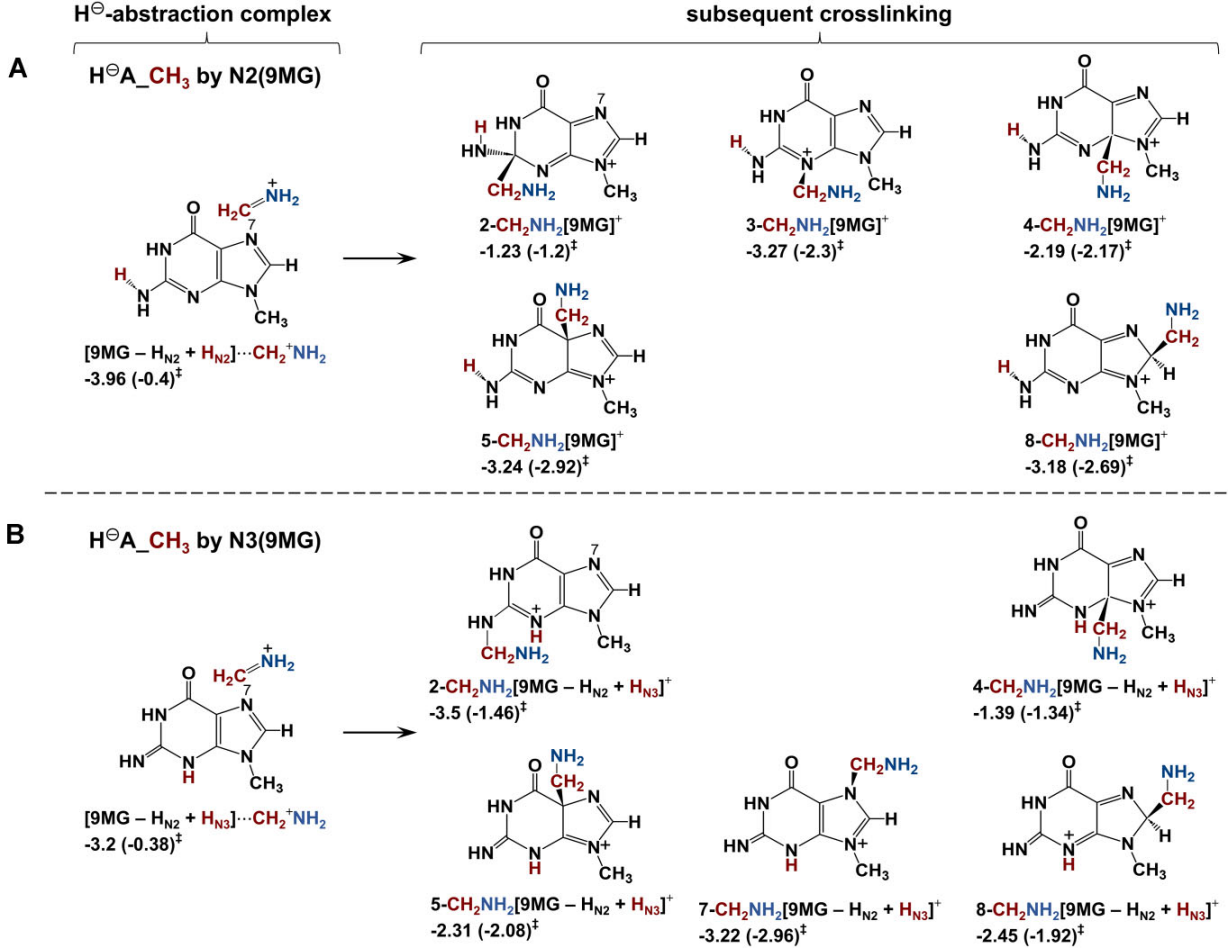
Methyl hydride abstraction for [9MG – H]^+^ + CH_3_NH_2_ and subsequent crosslinking. Reaction enthalpies (eV) and activation barriers (in parentheses) were calculated at ωB97XD/6-31+G(d,p).

We are more interested in exploring stand-alone H^⊝^A without subsequent PT, as these products may contribute to DPCs. Reactions (5.4–5.5) present the products formed from methyl hydride abstraction by the N2 and N3 of [9MG – H]^+^. Experimentally, no ^+^NH_2_CD_2_ product ions were detected, suggesting that these hydride abstraction products, once generated, undergo further reactions leading to DPC formation, as depicted in Scheme 4. Based on density of states calculations, the two dominant adducts are 5-CH_2_NH_2_[9MG]^+^ and 8-CH_2_NH_2_[9MG]^+^, each contributing ∼50% to the adduct population. A significant KIE is expected, with *k*_H_/*k*_D_ ratios of 4.5–5.0 for reaction (5.4) and 2.3–4.0 for reaction (5.5) at *E*_CM_ = 0.05–0.3 eV. The Wigner tunneling factors are 1.32 (for CH_3_NH_2_) and 1.48 (CD_3_NH_2_) for reaction (5.4), and 1.83 (CH_3_NH_2_) and 2.33 (CD_3_NH_2_) for reaction (5.5). It is noteworthy that H^⊝^A may initiate at the amine group; however, the resulting CH_3_NH^+^ product rapidly rearranges to CH_2_^+^NH_2_.

**Scheme 5.**
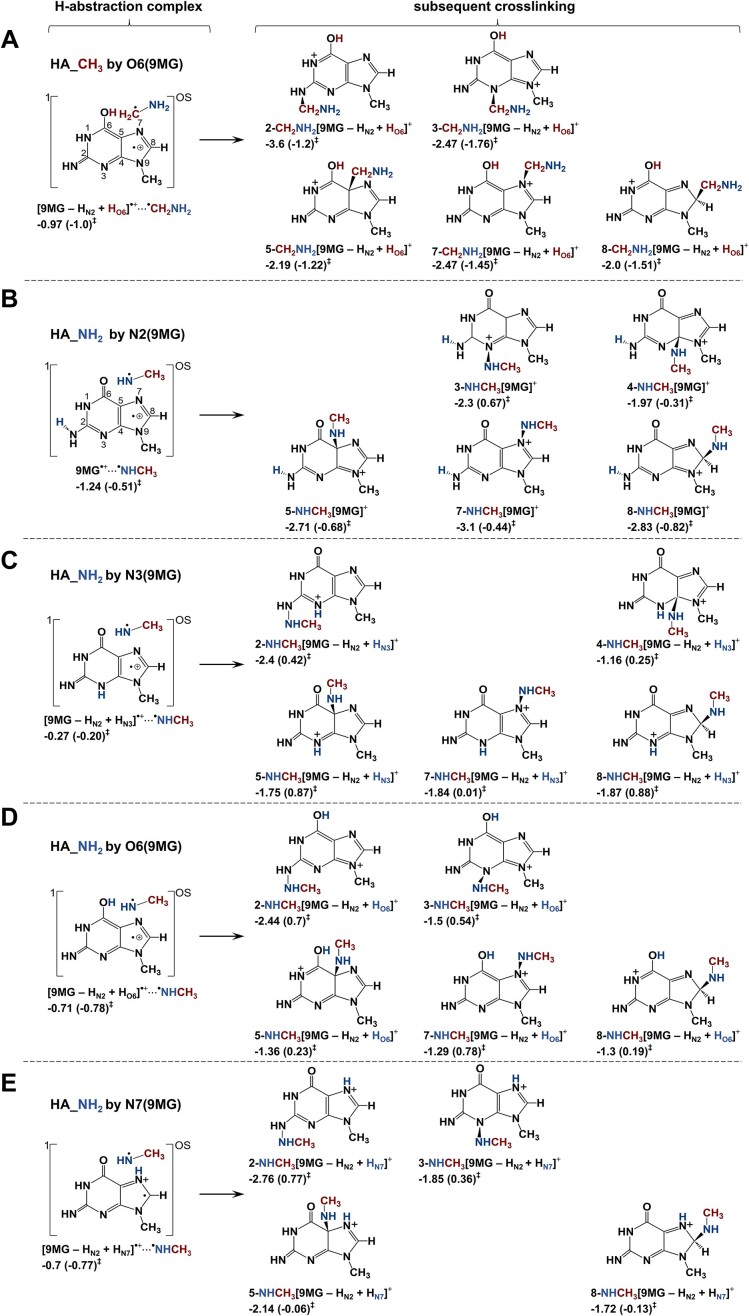
Amine and methyl hydrogen abstraction for [9MG – H]^+^ + CH_3_NH_2_ and subsequent crosslinking. Reaction enthalpies (eV) and activation barriers (in parentheses) were calculated using approximately spin-projected ωB97XD/6-31+G(d,p).

**(3) *H abstraction and combination of radical product pairs***: To facilitate a point-to-point comparison with the reaction pathways of 9MG^•+^ + CH_3_NH_2_, hydrogen abstraction for [9MG – H]^+^ + CH_3_NH_2_ was evaluated using open-shell DFT calculations. Only one methyl-H abstraction pathway was identified, as shown in reaction (6.1) and Scheme 5A. On the other hand, reactions (6.2–6.5) and Scheme 5B–E illustrate a total of four probable pathways for amine-H abstraction. The H-abstraction reactions yield radical product pairs, ^1,OS^[9MG^•+^(↑)⋅⋅⋅(↓)^•^CH_2_NH_2_] and ^1,OS^[9MG^•+^(↑)⋅⋅⋅(↓)^•^NHCH_3_], which introduce spin contamination in single-reference DFT calculations. To resolve this issue, approximate spin projection [80–83] was applied for the spin purification of reaction electronic energies.


\begin{eqnarray*}{{\mathrm{[9MG }} \,-\, {{\mathrm{H}}_{{\mathrm{N2}}}}{\mathrm{]}}^{\mathrm{ +}}} + {\mathrm{ C}}{{\mathrm{H}}_{\mathrm{3}}}{\mathrm{N}}{{\mathrm{H}}_{\mathrm{2}}} \to \end{eqnarray*}

(6.1) \begin{eqnarray*} [9{\mathrm{MG}} -{\mathrm{H}}_{\mathrm{N2}} + {\mathrm{H}}_{O6}]^{\bullet +}{\cdots}^{\bullet} {\mathrm{CH}}_{2} {\mathrm{NH}}_{2} \quad\Delta {\mathrm{H = - 0.97\, eV, TS = - 1.00\, eV}} \to [9{\mathrm{MG}} - {\mathrm{H}}_{\mathrm{N2}} + {\mathrm{H}}_{O6}]^{\bullet+} +\, {}^{\bullet}{\mathrm{CH}}_{2}{\mathrm{NH}}_{2} \quad\Delta {\mathrm{H = - 0.04\, eV}} \end{eqnarray*}

(6.2) \begin{eqnarray*} {\mathrm{9M}}{\mathrm{G}}^{\bullet + }{\cdots}^{\bullet} {\mathrm{NHC}}{\mathrm{H}}_{3} \quad\Delta {\mathrm{H = - 1.24\, eV, TS = - 0.51\, eV}} \to {\mathrm{9M}}{\mathrm{G}}^{\bullet+} + {}^{\bullet}{\mathrm{NHC}}{\mathrm{H}}_{3} \quad\Delta {\mathrm{H = - 0.37\, eV}} \end{eqnarray*}

(6.3) \begin{eqnarray*} {{\mathrm{[9MG }} \,-\, {{\mathrm{H}}_{{\mathrm{N2}}}} + {{\mathrm{H}}_{{\mathrm{N3}}}}{\mathrm{]}}^{{\mathrm{ \bullet +}}}}{ \cdots ^{\mathrm{ \bullet}}}{\mathrm{NHC}}{{\mathrm{H}}_{\mathrm{3}}} \quad\Delta {\mathrm{H = - 0}}{\mathrm{.27\, eV, TS = - 0}}{\mathrm{.20\, eV}} \to {{\mathrm{[9MG }} \,-\, {{\mathrm{H}}_{{\mathrm{N2}}}} + {{\mathrm{H}}_{{\mathrm{N3}}}}{\mathrm{]}}^{{\mathrm{ \bullet +}}}}+ {}^{\bullet}{\mathrm{NHC}}{{\mathrm{H}}_{\mathrm{3}}} \quad\Delta {\mathrm{H = 0}}{\mathrm{.74\, eV}} \end{eqnarray*}

(6.4) \begin{eqnarray*} {{\mathrm{[9MG }} \,-\, {{\mathrm{H}}_{{\mathrm{N2}}}} + {{\mathrm{H}}_{{\mathrm{O6}}}}{\mathrm{]}}^{{\mathrm{ \bullet +}}}}{ \cdots ^{\mathrm{ \bullet}}}{\mathrm{NHC}}{{\mathrm{H}}_{\mathrm{3}}} \quad\Delta {\mathrm{H = - 0}}{\mathrm{.71\, eV, TS = - 0}}{\mathrm{.78\, eV}} \to {{\mathrm{[9MG }} \,-\, {{\mathrm{H}}_{{\mathrm{N2}}}} + {{\mathrm{H}}_{{\mathrm{O6}}}}{\mathrm{]}}^{{\mathrm{ \bullet +}}}}+ {}^{\bullet}{\mathrm{NHC}}{{\mathrm{H}}_{\mathrm{3}}} \quad\Delta {\mathrm{H = 0}}{\mathrm{.25\, eV}} \end{eqnarray*}

(6.5) \begin{eqnarray*} {{\mathrm{[9MG }} \,-\, {{\mathrm{H}}_{{\mathrm{N2}}}} + {{\mathrm{H}}_{{\mathrm{N7}}}}{\mathrm{]}}^{{\mathrm{ \bullet +}}}}{ \cdots ^{\mathrm{ \bullet}}}{\mathrm{NHC}}{{\mathrm{H}}_{\mathrm{3}}} \quad\Delta {\mathrm{H = - 0}}{\mathrm{.70\, eV, TS = - 0}}{\mathrm{.77\, eV}} \to {{\mathrm{[9MG }} \,-\, {{\mathrm{H}}_{{\mathrm{N2}}}} + {{\mathrm{H}}_{{\mathrm{N7}}}}{\mathrm{]}}^{{\mathrm{ \bullet +}}}}+ {}^{\bullet}{\mathrm{NHC}}{{\mathrm{H}}_{\mathrm{3}}} \quad\Delta {\mathrm{H = 0}}{\mathrm{.16\, eV}} \end{eqnarray*}

The exothermic, barrierless methyl-H abstraction of CH_3_NH_2_ via reaction (6.1) results in the formation of the major experimental product ion, i.e. *d*_1_-[9MG – H_N2_ + H_O6_] ^•+^ at *m*/*z* 166 in Fig. 2A. The significance of methyl-H abstraction is further reflected by the potential combination of [9MG – H_N2_ + H_O6_]^•+^ with ^•^CH_2_NH_2_ to form a DPC, as illustrated in Scheme 5A.

The product-like complex 9MG^•+^⋅⋅⋅^•^NHCH_3_, formed in reaction (6.2), is the most favorable outcome of amine-H abstraction. The associated TS has an imaginary frequency of 446 cm^−1^, indicating negligible tunneling. While 9MG^•+^⋅⋅⋅^•^NHCH_3_ may separate into 9MG^•+^ + ^•^NHCH_3_, with an asymptote at −0.37 eV, the covalent association of the radical product pair is kinetically more favorable. This may explain the low intensity of product ions at *m*/*z* 165 in Fig. 2A. Kinetics modeling suggests that 8-NHCH_3_[9MG]^+^ is the dominating adduct (if any) resulting from the combination of amine-H abstraction products.

### Comparison of one- versus two-electron oxidation-mediated crosslinking

Table 1 highlights distinct features and differences in crosslinking mediated by 9MG^•+^ versus [9MG – H]^+^. Reactions were examined over a wide range of energy, and the lowest reaction energy was selected to match the thermal energy of reactants at room temperature, ensuring biological relevance. The crosslinking yield for 9MG^•+^ is <3% at thermal energy and becomes negligible at energies >0.3 eV. In contrast, the crosslinking yield for [9MG – H]^+^ exceeds 9% at thermal energy and remains significant (1.5%) even at high energies.

**Table 1.** The comparison of CH_3_NH_2_ with 9MG^•+^ versus [9MG – H]^+^

	Thermal energy			High energy (1–2 eV)		
	Yield%	Mechanism	Adducts	Yield%	Mechanism	Adducts
9MG^•+^	2.8	HA_CH_3_ (major)	8-CH_2_NH_2_[9MG + H_N7_]^•+^	0	N/A	None
		Direct addition	X-NH_2_CH_3_[9MG]^•+^(*X* = C2, C8)			
						
[9MG – H]^+^	9.3	Direct addition (major)	X-^+^NH_2_CH_3_[9MG – H_N2_] (major, *X* = N2, N3, C5, and C8)	1.5	Direct addition	X-^+^NH_2_CH_3_[9MG – H_N2_] (*X* = N2, N3, C5, and C8)
		HA_CH_3_	X-CH_2_NH_2_[9MG – H_N2_ + H_O6_]^+^ (minor, *X* = N2, N3, C5, N7, and C8)			

The large difference in DPC yields and their energy dependence can be attributed to distinct crosslinking mechanisms. For 9MG^•+^, crosslinking is mediated by direct addition and, to a greater extent, by covalent combination of the product pair [9MG + H]^+^⋅⋅⋅^•^CH_2_NH_2_ resulting from methyl-H abstraction. As the formation efficiency and lifetime of complexes are both suppressed by energy, it is rational that crosslinking diminishes as reaction energy increases. In contrast, crosslinking for [9MG – H]^+^ is predominant driven by direct addition between reactants. Since the addition pathways for [9MG – H]^+^ + CH_3_NH_2_ are exothermic and barrierless, reaction yield at high energies is mainly influenced by dynamics factors such as reaction orientations and thus becomes nearly constant. Methyl-H abstraction assists in crosslinking for [9MG – H]^+^ only at low energies. Following divergent reaction mechanisms, the crosslinking for 9MG^•+^ is primarily dominated by a 8-CH_2_NH_2_[9MG + H_N7_]^•+^ adduct, whereas the crosslinking for [9MG – H]^+^ leads to X-^+^NH_2_CH_3_[9MG – H_N2_] (*X* = N2, N3, C5, and C8) and their proton tautomers, with a minor contribution from X-CH_2_NH_2_[9MG – H_N2_ + H_O6_]^+^ (*X* = N2, N3, C5, N7, and C8) at low energies.

## Conclusions

This study presents a synergistic experimental and computational investigation into the reactions of methylamine with 9MG^•+^ and [9MG – H]^+^, designed to mimic and elucidate DNA–protein crosslinks induced by one- and two-electron oxidized guanosine nucleosides. While both singly and doubly oxidized guanosine species form under oxidative stress, their roles in the formation of DPCs remain elusive because of the difficulties in separating and characterizing these short-lived intermediates in solution-phase systems. This combined gas-phase experimental and theoretical work has, for the first time, uncovered new and distinct crosslinking mechanisms, products, yields, and reaction energy dependence for the two species. Crosslinking between 9MG^•+^ and methylamine primarily initiates with hydrogen abstraction from methylamine by 9MG^•+^, followed by the addition of the nascent ^•^CH_2_NH_2_ radical to protonated [9MG + H]^+^. Another pathway involves the addition of methylamine to the C2 and C8 of 9MG^•+^, as previously reported. Despite being exothermic and barrierless, crosslinking for 9MG^•+^ occurs only at low reaction energies and exhibits a low yield. In contrast, [9MG – H]^+^ predominantly undergoes direct addition with the N-terminal of methyl amine, followed by proton tautomerization of the product. The reaction operates efficiently across a wide range of energies and achieves three times the crosslinking yield of 9MG^•+^. Hydrogen abstraction from methylamine also contributes to the crosslinking of [9MG – H]^+^. This work has identified key intermediates and adduct structures involved in DPC formation and, particularly, underscored the critical role of two-electron nucleoside oxidation in driving these processes. The gas-phase experimental and computational results not only offer guidance for the experimental exploration of various structures in solution-phase DNA–protein coupling within biochemical systems but also serve as a foundation for understanding downstream oxidative DNA damage resulting from primary DPC products.

## Supplementary Material

gkaf071_Supplemental_File

## Data Availability

All data are incorporated into the article and its online supplementary material.
