# Supplementary material for: Crosslinking pathways, dynamics, and kinetics between guanosine and lysine following one- versus two-electron oxidation of guanosine
Source: Nucleic Acids Res. 2025 Mar 4;53(5):gkaf071. doi: 10.1093/nar/gkaf071 (PMC11879467; doi:10.1093/nar/gkaf071)
Supplement: gkaf071_Supplemental_File [file gkaf071_supplemental_file.pdf]

Supporting Information

Crosslinking Pathways, Dynamics, and Kinetics between Guanosine and Lysine following One- vs. Two-Electron Oxidation of Guanosine

May Myat Moe,[ab] Jonathan Benny,[ab] Varonica Lee,[a] Midas Tsai,[c] and Jianbo Liu*[ab]
[a] Department of Chemistry and Biochemistry, Queens College of the City University of New York, 65-30 Kissena Blvd., Queens, NY 11367, USA
[b] Ph.D. Program in Chemistry, the Graduate Center of the City University of New York, 365 5th Ave., New York, NY 10016, USA
[c] Department of Natural Sciences, LaGuardia Community College, 31-10 Thomson Ave., Long Island City, NY 11101, USA

Table of Contents

Formation and characterization of $[9MG - H]^+$ S2
Figures S1 CID of $[9MG - H]^+$, $9MG^{\bullet+}$ and $[9MG + H]^+$ S3
Scheme S1 Additional methyl-H abstraction pathways for $9MG^{\bullet+}$ and CH_3NH_2 .... S4
Scheme S2 Additional amine-H abstraction pathways for $9MG^{\bullet+}$ and CH_3NH_2 .... S5
Scheme S3 Additional direct addition pathways for $[9MG - H]^+$ and CH_3NH_2 .... S6
Scheme S4 Methyl-H⊖ abstraction pathways for $[9MG - H]^+$ and CH_3NH_2 .... S7
Table S1 Reaction energetics for pathways in Scheme 2A and Scheme S1 .... S8
Table S2 Reaction energetics for pathways in Scheme 2B and Scheme S2 .... S9
Table S3 Reaction energetics for pathways in Scheme 2C–D S10
Table S4 Reaction energetics for pathways in Scheme 3 and Scheme S3 .... S11
Table S5 Reaction energetics for pathways in Scheme 4 and Scheme S4 .... S12
Table S6 Reaction energetics for pathways in Scheme 5 .... S13
Cartesian coordinates for structures in Table S1 .... S14
Cartesian coordinates for structures in Table S2 .... S25
Cartesian coordinates for structures in Table S3 .... S36
Cartesian coordinates for structures in Table S4 .... S42
Cartesian coordinates for structures in Table S5 .... S56
Cartesian coordinates for structures in Table S6 .... S66

Formation and characterization of $[9MG - H]^+$

The doubly oxidized $[9MG - H]^+$ was generated from the gas-phase reactions of $9MG^{\bullet+}$. Note that $[9MG - H_{N1}]^+$ was proposed as the global minimum for $[9MG - H]^+$ in aqueous solution (1). However, in the gas phase, $[9MG - H_{N2}]^+$ is 0.15 eV lower in energy than $[9MG - H_{N1}]^+$, rendering $[9MG - H_{N2}]^+$ the dominant gaseous structure. Three pathways can be proposed for the formation of $[9MG - H]^+$:

$9MG^{\bullet+} \rightarrow [9MG - H]^+ + H$	$\Delta H = 4.6$ eV	(S1)
$9MG^{\bullet+} + 9MG^{\bullet+} \rightarrow [9MG - H]^+ + [9MG + H]^+$	$\Delta H = 0.51$ eV	(S2)
$9MG^{\bullet+} \rightarrow [9MG - H]^{\bullet} + H^+$	$\Delta H = 0.57$ eV	(S3a)
$[9MG - H]^{\bullet} + 9MG^{\bullet+} \rightarrow [9MG - H]^+ + 9MG$	$\Delta H = 0.71$ eV	(S3b)

The H elimination in reaction (S1), despite being the simplest, was not observed in the CID of $9MG^{\bullet+}$ with Xe (this work) or nitrogen gas (2) due to a very high dissociation energy. Therefore, it could be ruled out. $[9MG - H]^+$ may be generated via H transfer between two $9MG^{\bullet+}$ in reaction (S2); however, the reaction is less likely to occur due to significant Coulombic repulsion between two cationic reactants.

Reaction (S3a – b) represents the most probable formation mechanism. The deprotonation of $9MG^{\bullet+}$ is exothermic and occurs readily in aqueous solution but becomes moderately endothermic in the gas phase, with a threshold energy of 0.57 eV. The resulting deprotonated radicals, $[9MG - H]^{\bullet}$, can undergo charge transfer with $9MG^{\bullet+}$, with a reaction threshold of 0.71 eV. Given numerous collisions of $9MG^{\bullet+}$ radical cations within an electric filed between the exit of desolvation capillary (biased at 80 V relative to ground) and the skimmer (biased at 20 V) in the mass spectrometer source chamber, the deprotonation and subsequent charge transfer are expected to be feasible.

To confirm the structure of $[9MG - H]^+$, we compared the collision-induced dissociation (CID) tandem mass spectra of three individually mass-selected species: $[9MG - H]^+$, $9MG^{\bullet+}$, and $[9MG + H]^+$. The CID experiment was conducted at a center-of-mass collision energy of 3.0 eV, using the Xe atoms as collision gas. The dissociation product ions are presented in Figure S1, where black-colored portions of the ChemDraw structures represent observed fragment ions, and gray-colored portions indicate neutral losses. A common fragmentation feature for all three species is the elimination of neutral HCN and CHN_2. Notably, $9MG^{\bullet+}$ and $[9MG + H]^+$ exhibit nearly identical fragments, whereas $[9MG - H]^+$ exhibits the absence of NH_3, CH_2N_2, and HNCO losses. The unique fragmentation pattern of $[9MG - H]^+$ highlights its distinct conjugation structure, particularly the N2-imine group.

References

1 Thapa, B.; Hebert, S. P.; Munk, B. H.; Burrows, C. J.; Schlegel, H. B. (2019) Computational study of the formation of C8, C5, and C4 guanine:lysine adducts via oxidation of guanine by sulfate radical anion. *J. Phys. Chem. A,* **123**, 5150-5163.

2 Cheng, P.; Li, Y.; Li, S.; Zhang, M.; Zhou, Z. (2010) Collision-induced dissociation (CID) of guanine radical cation in the gas phase: An experimental and computational study. *Phys. Chem. Chem. Phys.*, **12,** 4667-4677.

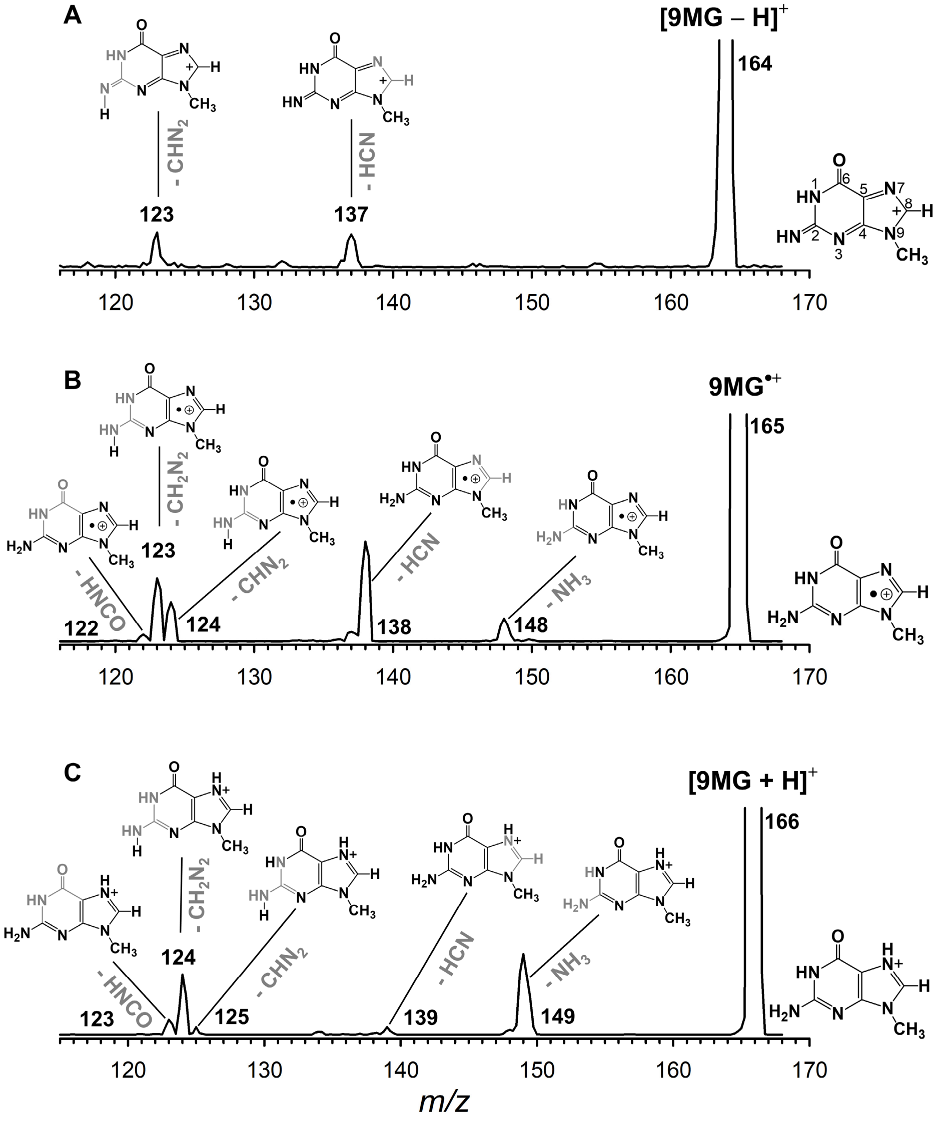

Figure S1. CID product ion mass spectra of $[9MG – H]^+$, $9MG^{•+}$, and $[9MG + H]^+$ recorded at E_{CM} = 3.0 eV. Gray structures represent possible neutral fragments.

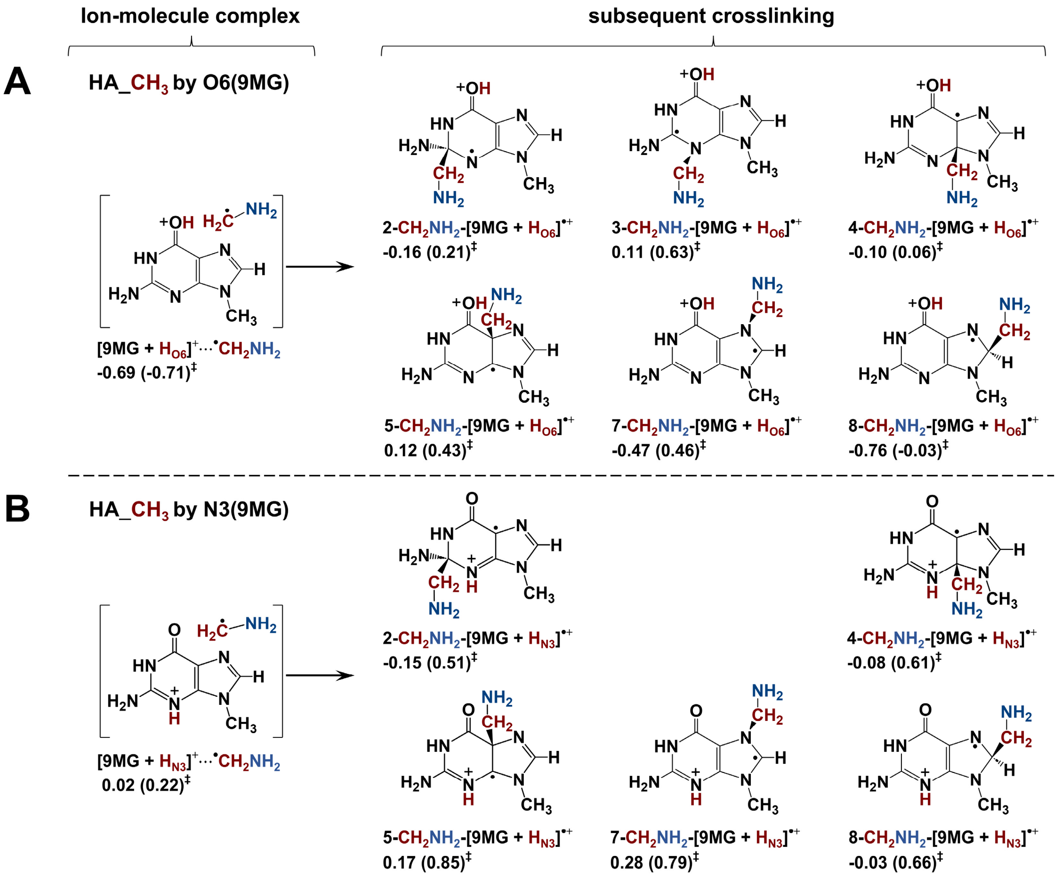

Scheme S1. Additional methyl-H abstraction pathways for $9MG^{\bullet+}$ + CH_3NH_2 and subsequent crosslinking. Reaction enthalpies (eV) and activation barriers (in parentheses) were calculated at ωB97XD/6-31+G(d,p).

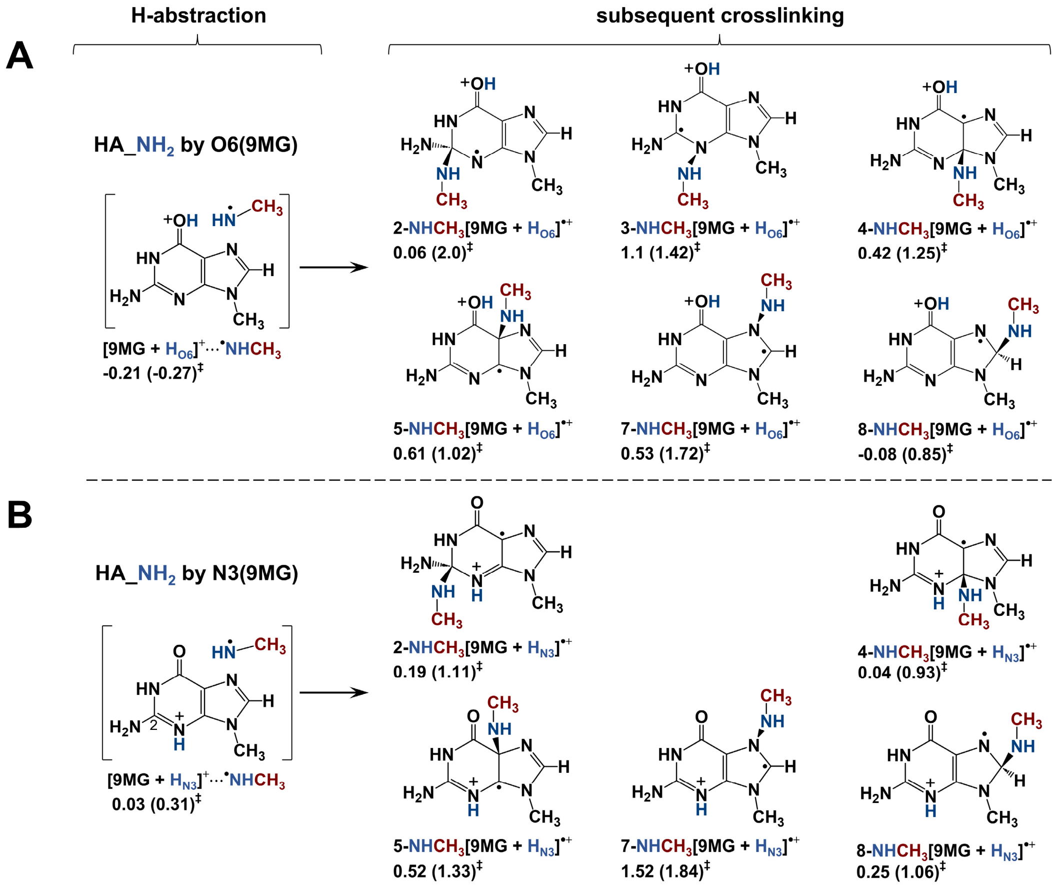

Scheme S2. Additional amine-H abstraction pathways for $9MG^{\bullet+}$ + CH_3NH_2 and subsequent crosslinking. Reaction enthalpies (eV) and activation barriers (in parentheses) were calculated at ωB97XD/ 6-31+G(d,p).

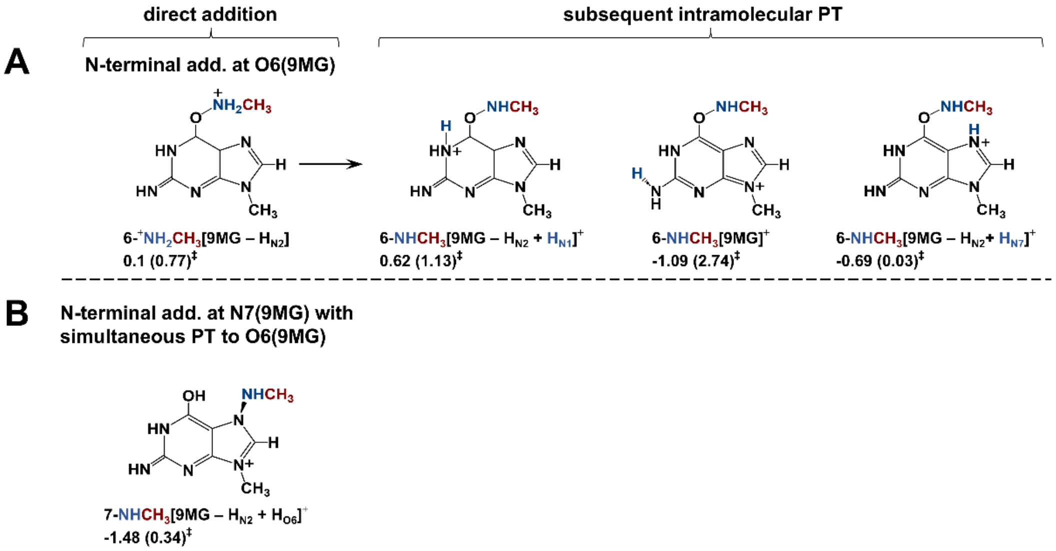

Scheme S3. Additional direct addition pathways for $[9MG - H]^+ + CH_3NH_2$ and subsequent PT. Reaction enthalpies (eV) and activation barriers (in parentheses) were calculated at ωB97XD/6-31+G(d,p).

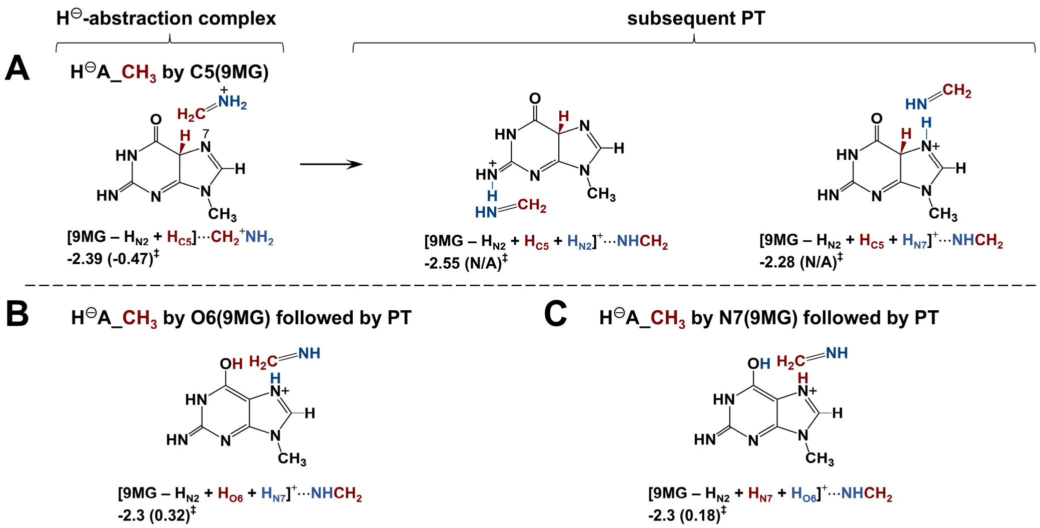

Scheme S4. Sequential methyl-H⊖ abstraction and PT for $[9MG - H]^{+} + CH_3NH_2$. Reaction enthalpies (eV) and activation barriers (in parentheses) were calculated at ωB97XD/6-31+G(d,p).

Table S1. Reaction energetics (eV) for HA_CH_3 pathways in Scheme 2A and Scheme S1

paths	products	ΔH (TS) [a]	ΔH (TS) [b]
HA_CH_3 by N7(9MG)	$[9MG + H_{N7}]^{+}\cdots{}^{\bullet}CH_2NH_2$ (complex)	-0.88 (-0.74)	-0.73 (-0.49)
	$\rightarrow [9MG + H_{N7}]^{+} + {}^{\bullet}CH_2NH_2$	-0.14	-0.12
subseq. crosslinking	$\rightarrow$ 2-$CH_2NH_2[9MG + H_{N7}]^{\bullet+}$	-0.10 (0.04)	-0.03 (0.18)
	$\rightarrow$ 3-$CH_2NH_2[9MG + H_{N7}]^{\bullet+}$	0.35 (0.67)	0.44 (0.79)
	$\rightarrow$ 4-$CH_2NH_2[9MG + H_{N7}]^{\bullet+}$	-0.29 (-0.03)	-0.18 (0.12)
	$\rightarrow$ 5-$CH_2NH_2[9MG + H_{N7}]^{\bullet+}$	-0.58 (-0.07)	-0.45 (0.05)
	$\rightarrow$ 8-$CH_2NH_2[9MG + H_{N7}]^{\bullet+}$	-1.38 (-0.52)	-1.20 (-0.34)
HA_CH_3 by O6(9MG)	$[9MG + H_{O6}]^{+}\cdots{}^{\bullet}CH_2NH_2$ (complex)	-0.69 (-0.71)	-0.54 (-0.47)
	$\rightarrow [9MG + H_{O6}]^{+} + {}^{\bullet}CH_2NH_2$	0.18	0.14
subseq. crosslinking	$\rightarrow$ 2-$CH_2NH_2[9MG + H_{O6}]^{\bullet+}$	-0.16 (0.21)	-0.14 (0.29)
	$\rightarrow$ 3-$CH_2NH_2[9MG + H_{O6}]^{\bullet+}$	0.11 (0.63)	0.20 (0.79)
	$\rightarrow$ 4-$CH_2NH_2[9MG + H_{O6}]^{\bullet+}$	-0.10 (0.06)	-0.02 (0.13)
	$\rightarrow$ 5-$CH_2NH_2[9MG + H_{O6}]^{\bullet+}$	0.12 (0.43)	0.25 (0.51)
	$\rightarrow$ 7-$CH_2NH_2[9MG + H_{O6}]^{\bullet+}$	-0.47 (0.46)	-0.40 (0.61)
	$\rightarrow$ 8-$CH_2NH_2[9MG + H_{O6}]^{\bullet+}$	-0.76 (-0.03)	-0.68 (0.06)
HA_CH_3 by N3(9MG)	$[9MG + H_{N3}]^{+}\cdots{}^{\bullet}CH_2NH_2$ (complex)	0.02 (0.22)	0.11 (0.44)
	$\rightarrow [9MG + H_{N3}]^{+} + {}^{\bullet}CH_2NH_2$	0.66	0.63
subseq. crosslinking	$\rightarrow$ 2-$CH_2NH_2[9MG + H_{N3}]^{\bullet+}$	-0.15 (0.51)	-0.09 (0.66)
	$\rightarrow$ 4-$CH_2NH_2[9MG + H_{N3}]^{\bullet+}$	-0.08 (0.61)	0.06 (0.71)
	$\rightarrow$ 5-$CH_2NH_2[9MG + H_{N3}]^{\bullet+}$	0.17 (0.85)	0.22 (0.93)
	$\rightarrow$ 7-$CH_2NH_2[9MG + H_{N3}]^{\bullet+}$	0.28 (0.79)	0.39 (0.84)
	$\rightarrow$ 8-$CH_2NH_2[9MG + H_{N3}]^{\bullet+}$	-0.03 (0.66)	0.08 (0.74)

[a] Calculated at ωB97XD/6-31+G(d,p), including 298 K thermal corrections.

[b] Calculated at DLPNO-CCSD(T)/aug-cc-pVQZ//ωB97XD/6-31+G(d,p), including 298 K thermal corrections.

Table S2. Reaction energetics (eV) for HA_NH$_2$ pathways in Scheme 2B and Scheme S2

paths	products	ΔH (TS) [a]	ΔH (TS) [b]
HA_NH$_2$ by N7(9MG)	[9MG + H$_{N7}$]$^+$···$^•$NHCH$_3$ (complex)	-0.79 (-0.84)	-0.66 (-0.66)
	→ [9MG + H$_{N7}$]$^+$ + $^•$NHCH$_3$	0.14	0.16
subseq. crosslinking	→ 2-NHCH$_3$[9MG + H$_{N7}$]$^{•+}$	0.07 (0.72)	0.11 (1.97)
	→ 3-NHCH$_3$[9MG + H$_{N7}$]$^{•+}$	1.29 (1.45)	1.49 (1.58)
	→ 4-NHCH$_3$[9MG + H$_{N7}$]$^{•+}$	-0.12 (0.56)	-0.003 (0.62)
	→ 5-NHCH$_3$[9MG + H$_{N7}$]$^{•+}$	-0.46 (0.45)	-0.33 (0.50)
	→ 8-NHCH$_3$[9MG + H$_{N7}$]$^{•+}$	-1.09 (0.02)	-0.94 (0.17)
HA_NH$_2$ by O6(9MG)	[9MG + H$_{O6}$]$^+$···$^•$NHCH$_3$ (complex)	-0.21 (-0.27)	-0.17 (-0.17)
	→ [9MG + H$_{O6}$]$^+$ + $^•$NHCH$_3$	0.47	0.42
subseq. crosslinking	→ 2-NHCH$_3$[9MG + H$_{O6}$]$^{•+}$	0.06 (2.0)	0.07 (1.93)
	→ 3-NHCH$_3$[9MG + H$_{O6}$]$^{•+}$	1.1 (1.42)	1.25 (1.43)
	→ 4-NHCH$_3$[9MG + H$_{O6}$]$^{•+}$	0.42 (1.25)	0.47(1.27)
	→ 5-NHCH$_3$[9MG + H$_{O6}$]$^{•+}$	0.61 (1.02)	0.69 (1.03)
	→ 7-NHCH$_3$[9MG + H$_{O6}$]$^{•+}$	0.53 (1.72)	0.69 (1.72)
	→ 8-NHCH$_3$[9MG + H$_{O6}$]$^{•+}$	-0.08 (0.85)	-0.06 (0.86)
HA_NH$_2$ by N3(9MG)	[9MG + H$_{N3}$]$^+$···$^•$NHCH$_3$ (complex)	0.03 (0.31)	0.12 (1.37)
	→ [9MG + H$_{N3}$]$^+$ + $^•$NHCH$_3$	0.95	0.91
subseq. crosslinking	→ 2-NHCH$_3$[9MG + H$_{N3}$]$^{•+}$	0.19 (1.11)	0.24 (1.22)
	→ 4-NHCH$_3$[9MG + H$_{N3}$]$^{•+}$	0.04 (0.93)	0.21 (0.98)
	→ 5-NHCH$_3$[9MG + H$_{N3}$]$^{•+}$	0.52 (1.33)	0.58 (1.32)
	→ 7-NHCH$_3$[9MG + H$_{N3}$]$^{•+}$	1.52 (1.84)	1.65 (1.87)
	→ 8-NHCH$_3$[9MG + H$_{N3}$]$^{•+}$	0.25 (1.06)	0.35 (1.1)

[a] Calculated at ωB97XD/6-31+G(d,p), including 298 K thermal corrections.

[b] Calculated at DLPNO-CCSD(T)/aug-cc-pVQZ//ωB97XD/6-31+G(d,p), including 298 K thermal corrections.

Table S3. Reaction energetics (eV) for direct addition pathways in Scheme 2C and D

paths	products	ΔH (TS) [a]	ΔH (TS) [b]
N-terminal add. at C2(9MG)	2-$^{+}NH_2CH_3$[9MG]$^{\bullet}$	-0.08 (-0.04)	-0.04 (0.05)
subseq. PT[c]	→ 2-$NHCH_3$[9MG + H_{N1}]$^{\bullet +}$	0.59 (1.24)	0.61 (1.32)
	→ 2-$NHCH_3$[9MG + H_{N2}]$^{\bullet +}$	0.15 (0.97)	0.19 (1.09)
	→ 2-$NHCH_3$[9MG + H_{N3}]$^{\bullet +}$	0.19 (1.05)	0.24 (1.18)
	→ 2-$NHCH_3$[9MG + H_{O6}]$^{\bullet +}$	0.06 (1.81)	0.06 (1.82)
	→ 2-$NHCH_3$[9MG + H_{N7}]$^{\bullet +}$	0.07 (3.04)	0.10 (2.96)
N-terminal add. at C8(9MG)	8-$^{+}NH_2CH_3$[9MG]$^{\bullet}$	-0.57 (-0.48)	-0.45 (-0.34)
subseq. PT	→ 8-$NHCH_3$[9MG + H_{N3}]$^{\bullet +}$	0.25 (2.0)	0.35 (2.0)
	→ 8-$NHCH_3$[9MG + H_{O6}]$^{\bullet +}$	-0.08 (1.41)	-0.06 (1.53)
	→ 8-$NHCH_3$[9MG + H_{N7}]$^{\bullet +}$	-1.09 (0.23)	-0.94 (0.42)

[a] Calculated at ωB97XD/6-31+G(d,p), including 298 K thermal corrections.

[b] Calculated at DLPNO-CCSD(T)/aug-cc-pVQZ//ωB97XD/6-31+G(d,p), including 298 K thermal corrections.

[c] PT barrier represents a single-step proton-transfer from -$^{+}NH_2$ to the target position; however, the barrier may decrease via multi-step consecutive proton transfer.

Table S4. Reaction energetics (eV) for direct addition pathways in Scheme 3 and Scheme S3

paths	products	ΔH (TS) [a]	ΔH (TS) [b]
N-terminal add. at N2(9MG)	2-$^{+}NH_2CH_3$[9MG – H_{N2}]	-1.83 (N/A)	-1.58 (N/A)
subseq. PT [c]	→ 2-$NHCH_3$[9MG – H_{N2} + H_{N1}]$^+$	-1.1 (-0.81)	-0.78 (-0.50)
	→ 2-$NHCH_3$[9MG]$^+$	-1.04 (0.58)	-0.83 (0.88)
	→ 2-$NHCH_3$[9MG – H_{N2} + H_{N3}]$^+$	-2.4 (-1.86)	-2.03 (-1.55)
	→ 2-$NHCH_3$[9MG – H_{N2} + H_{O6}]$^+$	-2.44 (0.21)	-2.12 (0.45)
	→ 2-$NHCH_3$[9MG – H_{N2} + H_{N7}]$^+$	-2.76 (2.51)	-2.37 (2.62)
N-terminal add. at N3(9MG)	3-$^{+}NH_2CH_3$[9MG – H_{N2}]	-0.62 (-0.36)	-0.45 (-0.17)
subseq. PT	→ 3-$NHCH_3$[9MG – H_{N2} + H_{N1}]$^+$	-0.27 (-0.04)	-0.14 (0.10)
	→ 3-$NHCH_3$[9MG]$^+$	-2.3 (-0.34)	-1.95 (-0.09)
	→ 3-$NHCH_3$[9MG – H_{N2} + H_{O6}]$^+$	-1.5 (1.34)	-1.22 (1.50)
	→ 3-$NHCH_3$[9MG – H_{N2} + H_{N7}]$^+$	-1.85 (2.42)	-1.50 (2.45)
N-terminal add. at C5(9MG)	5-$^{+}NH_2CH_3$[9MG – H_{N2}]	-1.8 (N/A)	-1.65 (N/A)
subseq. PT	→ 5-$NHCH_3$[9MG – H_{N2} + H_{N1}]$^+$	-1.03 (-0.89)	-1.06 (-0.77)
	→ 5-$NHCH_3$[9MG]$^+$	-2.71 (-0.71)	-2.48 (-0.52)
	→ 5-$NHCH_3$[9MG – H_{N2} + H_{N3}]$^+$	-1.75 (-0.27)	-1.61 (-0.13)
	→ 5-$NHCH_3$[9MG – H_{N2} + H_{O6}]$^+$	-1.36 (-1.08)	-1.24 (-0.91)
	→ 5-$NHCH_3$[9MG – H_{N2} + H_{N7}]$^+$	-2.14 (-0.76)	-1.92 (-0.54)
N-terminal add. at O6(9MG)	6-$^{+}NH_2CH_3$[9MG – H_{N2}]	0.1 (0.77)	0.28 (0.97)
subseq. PT	→ 6-$NHCH_3$[9MG – H_{N2} + H_{N1}]$^+$	0.62 (1.13)	0.8 (1.3)
	→ 6-$NHCH_3$[9MG]$^+$	-1.9 (2.74)	-1.61 (2.86)
	→ 6-$NHCH_3$[9MG – H_{N2} + H_{N7}]$^+$	-0.69 (0.03)	-0.41 (0.27)
N-terminal add. at N7(9MG) with simultaneous PT	7-$NHCH_3$[9MG – H_{N2} + H_{O6}]$^+$	-1.48 (0.34)	-1.17 (0.53)
N-terminal add. at C8(9MG)	8-$^{+}NH_2CH_3$[9MG – H_{N2}]	-1.67 (N/A)	-1.52 (N/A)
subseq. PT	→ 8-$NHCH_3$-[9MG – H_{N2} + H_{N1}]$^+$	-0.81 (1.21)	-0.69 (1.20)
	→ 8-$NHCH_3$[9MG]$^+$	-2.83 (1.12)	-2.55 (1.24)
	→ 8-$NHCH_3$[9MG – H_{N2} + H_{N3}]$^+$	-1.87 (0.77)	-1.73 (0.86)
	→ 8-$NHCH_3$[9MG – H_{N2} + H_{O6}]$^+$	-1.3 (0.49)	-1.20 (0.59)
	→ 8-$NHCH_3$[9MG – H_{N2} + H_{N7}]$^+$	-1.72 (-0.4)	-1.49 (-0.18)

[a] Calculated at ωB97XD/6-31+G(d,p), including 298 K thermal corrections.

[b] Calculated at DLPNO-CCSD(T)/aug-cc-pVQZ//ωB97XD/6-31+G(d,p), including 298 K thermal corrections.

[c] PT barrier represents a single-step proton-transfer from -$^{+}NH_2$ to the target position; however, the barrier may decrease via multi-step consecutive proton transfer.

Table S5. Reaction energetics (eV) for H⊖A_CH_3 pathways in Scheme 4 and Scheme S4

paths	products	ΔH (TS) [a]	ΔH (TS) [b]
H⊖A_CH_3 by N2(9MG)	9MG···$CH_2^+NH_2$ (complex)<br>→ 9MG + $CH_2^+NH_2$	-3.96 (-0.4)<br>-1.9	-3.69 (-0.08)<br>-1.7
subseq. crosslinking	→ 2-CH_2NH_2[9MG]$^+$	-1.23 (-1.2)	-1.03 (-0.87)
	→ 3-CH_2NH_2[9MG]$^+$	-3.27 (-2.29)	-3.0 (-1.99)
	→ 4-CH_2NH_2[9MG]$^+$	-2.19 (-2.17)	-1.98 (-1.87)
	→ 5-CH_2NH_2[9MG]$^+$	-3.24 (-2.92)	-2.97 (-2.63)
	→ 8-CH_2NH_2[9MG]$^+$	-3.18 (-2.69)	-2.89 (-2.35)
H⊖A_CH_3 by N3(9MG)	[9MG – H_{N2} + H_{N3}]···$CH_2^+NH_2$ (complex)<br>→ [9MG – H_{N2} + H_{N3}] + $CH_2^+NH_2$	-3.2 (-0.38)<br>-1.23	-3.0 (-0.15)<br>-1.08
subseq. crosslinking	→ 2-CH_2NH_2[9MG – H_{N2} + H_{N3}]$^+$	-3.5 (-1.46)	-3.17 (-1.16)
	→ 4-CH_2NH_2[9MG – H_{N2} + H_{N3}]$^+$	-1.39 (-1.34)	-1.16 (-1.05)
	→ 5-CH_2NH_2[9MG – H_{N2} + H_{N3}]$^+$	-2.31 (-2.08)	-2.13 (-1.84)
	→ 7-CH_2NH_2[9MG – H_{N2} + H_{N3}]$^+$	-3.22 (-2.96)	-2.95 (-2.71)
	→ 8-CH_2NH_2[9MG – H_{N2} + H_{N3}]$^+$	-2.45 (-1.92)	-2.28 (-1.67)
H⊖A_CH_3 by C5(9MG)	[9MG – H_{N2} + H_{C5}] ···$CH_2^+NH_2$ (complex)<br>→ [9MG – H_{N2} + H_{C5}] + $CH_2^+NH_2$	-2.39 (-0.47)<br>-0.78	-2.22 (-0.18)<br>-0.66
subseq. PT	→ [9MG + H_{C5}]$^+$···CH_2NH (complex)<br>→ [9MG + H_{C5}]$^+$ + CH_2NH<br>→ [9MG – H_{N2} + H_{C5}+ H_{N7}]$^+$···CH_2NH (complex)<br>→ [9MG – H_{N2} + H_{C5} + H_{N7}]$^+$ + CH_2NH	-2.55 (N/A)<br>-1.83<br>-2.28 (N/A)<br>-1.18	-2.36 (N/A)<br>-1.72<br>-2.14 (N/A)<br>-1.09
H⊖A_CH_3 by O6(9MG) + PT	[9MG – H_{N2} + H_{O6} + H_{N7}]$^+$···CH_2NH (complex)<br>→ [9MG – H_{N2} + H_{O6} + H_{N7}]$^+$ + CH_2NH	-2.3 (0.32)<br>-1.19	-2.1 (0.50)<br>-1.1
H⊖A_CH_3 by N7(9MG) + PT	[9MG – H_{N2} + H_{N7} + H_{O6}]$^+$···CH_2NH (complex)<br>→ [9MG – H_{N2} + H_{N7} + H_{O6}]$^+$ + CH_2NH	-2.3 (0.18)<br>-1.19	-2.1 (0.35)<br>-1.1

[a] Calculated at ωB97XD/6-31+G(d,p), including 298 K thermal corrections.

[b] Calculated at DLPNO-CCSD(T)/aug-cc-pVQZ//ωB97XD/6-31+G(d,p), including 298 K thermal corrections.

Table S6. Reaction energetics (eV) for HA_NH_2 and HA_CH_3 pathways in Scheme 5

paths	products	$\langle \hat{S}^2 \rangle_{complex}$ ($\langle \hat{S}^2 \rangle_{TS}$)	ΔH (TS) [a]	
			before projection	after projection
HA_CH_3 by O6(9MG)	[9MG – H_{N2} + H_{O6}]$^{•+}$···$^{•}CH_2NH_2$ (complex)<br>→ [9MG – H_{N2} + H_{O6}]$^{•+}$ + $^{•}CH_2NH_2$	**1.05** (**1.05**)	-0.97 (-1.00)<br>-0.04	**-0.97** (**-1.00**)<br>-0.04
subseq. crosslinking	→ 2-CH_2NH_2[9MG – H_{N2} + H_{O6}]$^+$	0.00 (0.00)	-3.60 (-1.20)	-3.60 (-1.20)
	→ 3-CH_2NH_2[9MG – H_{N2} + H_{O6}]$^+$	0.00 (0.00)	-2.47 (-1.76)	-2.47 (-1.76)
	→ 5-CH_2NH_2[9MG – H_{N2} + H_{O6}]$^+$	0.00 (0.00)	-2.19 (-1.22)	-2.19 (-1.22)
	→ 7-CH_2NH_2[9MG – H_{N2} + H_{O6}]$^+$	0.00 (0.00)	-2.47 (-1.45)	-2.47 (-1.45)
	→ 8-CH_2NH_2[9MG – H_{N2} + H_{O6}]$^+$	0.00 (0.00)	-2.00 (-1.51)	-2.00 (-1.51)
HA_NH_2 by N2(9MG)	9MG$^{•+}$···$^{•}NHCH_3$ (complex)<br>→ 9MG$^{•+}$ + $^{•}NHCH_3$	**1.02** (**0.87**)	-1.24 (-0.53)<br>-0.37	**-1.24** (**-0.51**)<br>-0.37
subseq. crosslinking	→ 3-$NHCH_3$[9MG]$^+$	0.00 (**0.65**)	-2.30 (0.58)	-2.30 (**0.67**)
	→ 4-$NHCH_3$[9MG]$^+$	0.00 (**0.78**)	-1.97 (-0.36)	-1.97 (**-0.31**)
	→ 5-$NHCH_3$[9MG]$^+$	0.00 (**0.72**)	-2.71 (-0.78)	-2.71 (**-0.68**)
	→ 7-$NHCH_3$[9MG]$^+$	0.00 (0.00)	-3.10 (-0.44)	-3.10 (-0.44)
	→ 8-$NHCH_3$[9MG]$^+$	0.00 (0.00)	-2.83 (-0.82)	-2.83 (-0.82)
HA_NH_2 by N3(9MG)	[9MG – H_{N2} + H_{N3}]$^{•+}$···$^{•}NHCH_3$ (complex)<br>→ [9MG – H_{N2} + H_{N3}]$^{•+}$ + $^{•}NHCH_3$	**1.03** (**1.03**)	-0.27 (-0.20)<br>0.74	**-0.27** (**-0.20**)<br>0.74
subseq. crosslinking	→ 2-$NHCH_3$[9MG – H_{N2} + H_{N3}]$^+$	0.00 (**0.85**)	-2.40 (0.40)	-2.40 (**0.42**)
	→ 4-$NHCH_3$[9MG – H_{N2} + H_{N3}]$^+$	0.00 (**0.85**)	-1.16 (0.22)	-1.16 (**0.25**)
	→ 5-$NHCH_3$[9MG – H_{N2} + H_{N3}]$^+$	0.00 (0.00)	-1.75 (0.87)	-1.75 (0.87)
	→ 7-$NHCH_3$[9MG – H_{N2} + H_{N3}]$^+$	0.00 (0.00)	-1.84 (0.01)	-1.84 (0.01)
	→ 8-$NHCH_3$[9MG – H_{N2} + H_{N3}]$^+$	0.00 (0.00)	-1.87 (0.88)	-1.87 (0.88)
HA_NH_2 by O6(9MG)	[9MG – H_{N2} + H_{O6}]$^{•+}$···$^{•}NHCH_3$ (complex)<br>→ [9MG – H_{N2} + H_{O6}]$^{•+}$ + $^{•}NHCH_3$	**1.05** (**1.05**)	-0.71 (-0.78)<br>0.25	**-0.71** (**-0.78**)
subseq. crosslinking	→ 2-$NHCH_3$[9MG – H_{N2} + H_{O6}]$^+$	0.00 (0.00)	-2.44 (0.70)	-2.44 (0.70)
	→ 3-$NHCH_3$[9MG – H_{N2} + H_{O6}]$^+$	0.00 (0.00)	-1.50 (0.54)	-1.50 (0.54)
	→ 5-$NHCH_3$[9MG – H_{N2} + H_{O6}]$^+$	0.00 (**0.72**)	-1.36 (0.13)	-1.36 (**0.23**)
	→ 7-$NHCH_3$[9MG – H_{N2} + H_{O6}]$^+$	0.00 (0.00)	-1.29 (0.78)	-1.29 (0.78)
	→ 8-$NHCH_3$[9MG – H_{N2} + H_{O6}]$^+$	0.00 (0.00)	-1.30 (0.19)	-1.30 (0.19)
HA_NH_2 by N7(9MG)	[9MG – H_{N2} + H_{N7}]$^{•+}$···$^{•}NHCH_3$ (complex)<br>→ [9MG – H_{N2} + H_{N7}]$^{•+}$ + $^{•}NHCH_3$	**1.05** (**1.05**)	-0.70 (-0.77)<br>0.16	**-0.70** (**-0.77**)<br>0.16
subseq. crosslinking	→ 2-$NHCH_3$[9MG – H_{N2} + H_{N7}]$^+$	0.00 (**0.78**)	-2.76 (0.71)	-2.76 (**0.77**)
	→ 3-$NHCH_3$[9MG – H_{N2} + H_{N7}]$^+$	0.00 (0.00)	-1.85 (0.36)	-1.85 (0.36)
	→ 5-$NHCH_3$[9MG – H_{N2} + H_{N7}]$^+$	0.00 (**0.85**)	-2.14 (-0.10)	-2.14 (**-0.06**)
	→ 8-$NHCH_3$[9MG – H_{N2} + H_{N7}]$^+$	0.00 (**0.93**)	-1.72 (-0.14)	-1.72 (**-0.13**)

[a] Singlet biradicals were calculated using unrestricted, broken-symmetry DFT vs. approximate spin projection (values in bold) at the ωB97XD/6-31+G(d,p) level of theory. All enthalpies include 298 K thermal corrections.

Coordinates for structures in Table S1, optimized at ωB97XD/6-31+G(d,p).

9MG•+
N1 1.951089 -0.354606 -0.005142
C2 0.216432 1.034959 -0.000539
C3 -1.205061 1.373599 0.001928
O4 -1.725500 2.455145 0.004341
N5 -1.999347 0.182774 0.001639
H6 -2.998658 0.360703 0.003298
C7 -1.529623 -1.090371 -0.000790
N8 -2.379565 -2.107275 -0.002563
H9 -3.382593 -1.994376 -0.003061
H10 -1.998213 -3.043644 -0.004027
N11 -0.209722 -1.400170 -0.001375
C12 0.580659 -0.354446 -0.001519
C13 2.813092 -1.534798 0.008441
H14 2.706697 -2.055012 0.961059
H15 2.530406 -2.196941 -0.810123
H16 3.844856 -1.211354 -0.122566
C17 2.319038 0.954047 -0.004590
N18 1.291151 1.814582 -0.002565
H19 3.359041 1.254389 -0.006842

Zero-point correction=	0.146470 (Hartree/Particle)
Thermal correction to Energy=	0.156362
Thermal correction to Enthalpy=	0.157306
Thermal correction to Gibbs Free Energy=	0.110575
Sum of electronic and zero-point Energies=	-581.292005
Sum of electronic and thermal Energies=	-581.282114
Sum of electronic and thermal Enthalpies=	-581.281170
Sum of electronic and thermal Free Energies=	-581.327900

CH_3NH_2
N1 0.746992 0.000020 -0.117560
C2 -0.705875 0.000029 0.017459
H3 -1.078835 -0.000100 1.053415
H4 -1.113571 0.880155 -0.487496
H5 -1.113680 -0.880149 -0.487353
H6 1.156244 0.817244 0.319994
H7 1.156151 -0.817466 0.319604

Zero-point correction=	0.064793 (Hartree/Particle)
Thermal correction to Energy=	0.068212
Thermal correction to Enthalpy=	0.069156
Thermal correction to Gibbs Free Energy=	0.041879
Sum of electronic and zero-point Energies=	-95.772913
Sum of electronic and thermal Energies=	-95.769494
Sum of electronic and thermal Enthalpies=	-95.768550
Sum of electronic and thermal Free Energies=	-95.795827

[9MG + H_{N7}]$^+$···•CH_2NH_2
C1 -0.224233 0.047287 -0.063949
C2 1.001205 0.676100 0.005418
C3 2.182519 -1.202042 0.013681
C4 -0.293299 -1.386037 -0.095287
C5 -0.591800 2.197097 -0.040404
N6 2.218343 0.116878 0.048514
N7 0.743788 2.032616 0.019806
N8 3.333345 -1.890875 0.046166
H9 3.378970 -2.895551 0.014787
H10 4.194321 -1.368468 0.090529
N11 -1.196927 1.023506 -0.088393
N12 -3.915668 -0.818842 0.535932
H13 -4.270817 -0.670612 1.466888
C14 -3.920835 0.201073 -0.381869
H15 -2.257368 0.850096 -0.181213
H16 -3.755711 -0.103728 -1.414132
H17 -4.654640 0.985027 -0.216774
C18 1.760804 3.078556 0.101429
H19 1.277269 4.048536 -0.006332
H20 2.265835 3.017956 1.065870
H21 2.485400 2.934583 -0.700024
H22 -1.089469 3.155059 -0.050055
N23 1.010109 -1.921585 -0.053261
H24 1.035818 -2.934631 -0.075756
O25 -1.270025 -2.110224 -0.148962
H26 -3.286505 -1.600562 0.402440

Zero-point correction=	0.212157 (Hartree/Particle)
Thermal correction to Energy=	0.226676
Thermal correction to Enthalpy=	0.227620
Thermal correction to Gibbs Free Energy=	0.170193
Sum of electronic and zero-point Energies=	-677.097638
Sum of electronic and thermal Energies=	-677.083118
Sum of electronic and thermal Enthalpies=	-677.082174
Sum of electronic and thermal Free Energies=	-677.139601

TS for [9MG + H_{N7}]$^+$···•CH_2NH_2
C1 -0.237291 0.128077 -0.002780
C2 1.051085 0.644345 0.011558
C3 2.066799 -1.328614 -0.020968
C4 -0.406801 -1.286146 -0.018434
C5 -0.431735 2.259854 0.032154
N6 2.218174 -0.023125 0.004617
O7 -1.442586 -1.957894 -0.021924
N8 0.909846 2.005412 0.035626
N9 3.151724 -2.124812 -0.030135
H10 3.102737 -3.127753 -0.084741
H11 4.057105 -1.682670 -0.037055
N12 -1.151674 1.163776 0.014008
N13 -3.854779 -0.751547 0.400919
H14 -3.098561 -1.451238 0.296043
C15 -3.808948 0.345662 -0.435481
H16 -4.627617 1.048821 -0.295973
H17 -2.751209 0.869452 -0.195603
H18 -3.635028 0.058838 -1.478739
C19 1.999767 2.972291 0.060704
H20 1.576691 3.976388 0.057560
H21 2.598244 2.829111 0.961273
H22 2.629898 2.837201 -0.819188
H23 -0.825534 3.266300 0.040855
N24 0.831797 -1.940689 -0.032534
H25 0.764017 -2.951462 -0.042393
H26 -4.382936 -0.725751 1.265318

Zero-point correction=	0.209719 (Hartree/Particle)
Thermal correction to Energy=	0.223520
Thermal correction to Enthalpy=	0.224465
Thermal correction to Gibbs Free Energy=	0.167897
Sum of electronic and zero-point Energies=	-677.091833
Sum of electronic and thermal Energies=	-677.078032
Sum of electronic and thermal Enthalpies=	-677.077087
Sum of electronic and thermal Free Energies=	-677.133655

•CH_2NH_2
N1 0.654332 0.000122 0.092835
H2 1.135106 0.835783 -0.207460
C3 -0.728236 0.000058 -0.081571
H4 -1.240146 -0.932443 0.125335
H5 -1.240653 0.931913 0.127076
H6 1.134780 -0.836455 -0.205372

Zero-point correction=	0.050735 (Hartree/Particle)
Thermal correction to Energy=	0.054180
Thermal correction to Enthalpy=	0.055124
Thermal correction to Gibbs Free Energy=	0.027563
Sum of electronic and zero-point Energies=	-95.128414
Sum of electronic and thermal Energies=	-95.124969

Sum of electronic and thermal Enthalpies= -95.124025
Sum of electronic and thermal Free Energies= -95.151587

2-$CH_2NH_2[9MG + H_{N7}]^{•+}$
C1 1.069568 0.885741 -0.130449
C2 0.759100 -0.440565 0.154759
C3 -1.520030 -0.004446 0.354959
C4 0.072251 1.955005 -0.013294
C5 2.930583 -0.278981 -0.244169
N6 -0.412831 -0.944484 0.443629
O7 0.331416 3.134390 -0.156750
N8 1.973207 -1.145137 0.071207
N9 -2.473726 -0.154921 1.411469
H10 -2.042411 -0.064968 2.325721
H11 -2.930090 -1.058833 1.352911
N12 2.409383 0.950556 -0.370057
H13 -2.963652 0.491371 -1.168137
H14 -2.347959 -2.393346 -0.990973
H15 -3.828273 -1.621255 -0.759842
C16 2.118976 -2.581388 0.306745
H17 3.156304 -2.864872 0.134720
H18 1.835484 -2.802530 1.335971
H19 1.464890 -3.120798 -0.378249
H20 3.973272 -0.524358 -0.383473
N21 -1.125515 1.413552 0.349861
H22 -1.883777 2.066551 0.512908
H23 2.920363 1.798410 -0.591763
C24 -2.236615 -0.313603 -1.034041
H25 -1.466806 -0.205090 -1.807410
N26 -2.899328 -1.563278 -1.148313

Zero-point correction= 0.215720 (Hartree/Particle)
Thermal correction to Energy= 0.228823
Thermal correction to Enthalpy= 0.229767
Thermal correction to Gibbs Free Energy= 0.176099
Sum of electronic and zero-point Energies= -677.067406
Sum of electronic and thermal Energies= -677.054303
Sum of electronic and thermal Enthalpies= -677.053359
Sum of electronic and thermal Free Energies= -677.107027

TS for 2-$CH_2NH_2[9MG + H_{N7}]^{•+}$
C1 1.047495 0.898704 -0.139751
C2 0.767074 -0.409116 0.212765
C3 -1.408385 -0.002781 0.585479
C4 0.058861 1.940557 0.008107
C5 2.914744 -0.248870 -0.319550
N6 -0.384381 -0.912475 0.634804
O7 0.212308 3.128306 -0.197864
N8 1.966827 -1.108041 0.081013
N9 -2.566350 -0.239367 1.353924
H10 -2.535993 0.244558 2.246271
H11 -2.674738 -1.232807 1.522564
N12 2.385641 0.961725 -0.458913
H13 -2.824077 0.485879 -1.288019
H14 -2.308947 -2.434221 -1.374880
H15 -3.767955 -1.640776 -0.942109
C16 2.138090 -2.525704 0.383215
H17 3.175151 -2.804739 0.201221
H18 1.882874 -2.701428 1.428671
H19 1.477983 -3.112831 -0.255541
H20 3.947227 -0.504355 -0.502339
N21 -1.129687 1.378498 0.472043
H22 -1.904748 2.024571 0.569067
H23 2.880757 1.798336 -0.741653
C24 -2.174840 -0.383663 -1.238558
H25 -1.254382 -0.325555 -1.816154
N26 -2.822029 -1.571460 -1.284933

Zero-point correction= 0.214320 (Hartree/Particle)
Thermal correction to Energy= 0.227519
Thermal correction to Enthalpy= 0.228464
Thermal correction to Gibbs Free Energy= 0.174534
Sum of electronic and zero-point Energies= -677.062313
Sum of electronic and thermal Energies= -677.049113
Sum of electronic and thermal Enthalpies= -677.048169
Sum of electronic and thermal Free Energies= -677.102099

3-$CH_2NH_2[9MG + H_{N7}]^{•+}$
C1 1.239013 0.696046 0.004466
C2 0.494692 -0.434530 -0.156374
C3 -1.306310 0.833027 -0.681944
C4 0.639031 2.026134 0.202810
C5 2.621129 -1.010495 -0.079200
N6 -0.883368 -0.471309 -0.248015
N7 1.377344 -1.498520 -0.207717
N8 -2.668745 0.955495 -0.942941
H9 -2.924937 1.847145 -1.351859
H10 -3.005770 0.202587 -1.531811
N11 2.554842 0.312304 0.044485
N12 -2.858164 -1.567985 0.825642
H13 -2.928351 -2.340483 0.178103
C14 -1.565132 -1.039486 1.007994
H15 3.340018 0.942457 0.162722
H16 -1.598483 -0.236849 1.751888
H17 -0.890330 -1.818688 1.374374
C18 1.015907 -2.903143 -0.403884
H19 1.824060 -3.410801 -0.929009
H20 0.836314 -3.384289 0.558666
H21 0.110971 -2.932048 -1.011441
H22 3.525124 -1.599696 -0.086801
N23 -0.715483 1.946718 -0.013184
H24 -1.241762 2.799530 0.139194
O25 1.257015 3.008109 0.562829
H26 -3.577930 -0.875977 0.672236

Zero-point correction= 0.217212 (Hartree/Particle)
Thermal correction to Energy= 0.230256
Thermal correction to Enthalpy= 0.231201
Thermal correction to Gibbs Free Energy= 0.177716
Sum of electronic and zero-point Energies= -677.050804
Sum of electronic and thermal Energies= -677.037760
Sum of electronic and thermal Enthalpies= -677.036815
Sum of electronic and thermal Free Energies= -677.090300

TS for 3-$CH_2NH_2[9MG + H_{N7}]^{•+}$
C1 -1.208327 -0.756702 0.083136
C2 -0.537778 0.387724 -0.255899
C3 1.334020 -0.717734 -0.726587
C4 -0.526226 -2.035961 0.225223
C5 -2.672957 0.881133 0.037434
N6 0.799051 0.517094 -0.489001
N7 -1.477162 1.406891 -0.273920
N8 2.657284 -0.803280 -1.132028
H9 2.885091 -1.623552 -1.682656
H10 2.961069 0.040019 -1.602376
N11 -2.528970 -0.423061 0.253335
N12 2.889631 1.488931 1.106670
H13 3.245503 2.267574 0.575152
C14 1.544318 1.308527 1.206267
H15 -3.265430 -1.072089 0.504149
H16 1.232923 0.521072 1.888379
H17 0.960547 2.223890 1.248071
C18 -1.206614 2.803850 -0.613309
H19 -2.017459 3.192278 -1.228884
H20 -1.106894 3.399186 0.295541
H21 -0.274738 2.827420 -1.178150
H22 -3.600142 1.430197 0.091972
N23 0.798923 -1.875747 -0.136847
H24 1.370460 -2.710129 -0.067539
O25 -1.027048 -3.072602 0.621490

H26 3.495523 0.684137 1.149374

Zero-point correction=	0.212958 (Hartree/Particle)
Thermal correction to Energy=	0.226701
Thermal correction to Enthalpy=	0.227645
Thermal correction to Gibbs Free Energy=	0.171906
Sum of electronic and zero-point Energies=	-677.039767
Sum of electronic and thermal Energies=	-677.026023
Sum of electronic and thermal Enthalpies=	-677.025079
Sum of electronic and thermal Free Energies=	-677.080819

4-CH₂NH₂[9MG + H$_{N7}$]$^{•+}$

C1 0.082932 1.085362 0.176875
C2 0.581726 -0.316534 0.153626
C3 -1.494554 -0.924829 -0.666081
C4 -1.318680 1.409260 0.051141
C5 2.170826 1.189480 -0.550248
N6 -0.234533 -1.208331 -0.603134
N7 1.921275 -0.091084 -0.474797
N8 -2.376487 -1.819421 -1.175477
H9 -3.253010 -1.515411 -1.569321
H10 -1.981579 -2.667861 -1.552324
N11 1.119337 1.926896 -0.153614
N12 -0.325186 -1.142740 2.386595
H13 -0.943082 -1.858833 2.031407
C14 0.839394 -0.908085 1.604872
H15 1.043692 2.933844 -0.233304
H16 1.514943 -0.227466 2.135260
H17 1.359241 -1.858665 1.448859
C18 2.815242 -1.180494 -0.847078
H19 3.572145 -0.809288 -1.537913
H20 3.296858 -1.597083 0.039927
H21 2.213017 -1.946600 -1.337171
H22 3.101320 1.617546 -0.900567
N23 -2.069692 0.263497 -0.198173
H24 -3.071741 0.398222 -0.248395
O25 -1.790769 2.528338 0.128200
H26 -0.829971 -0.321802 2.689505

Zero-point correction=	0.215780 (Hartree/Particle)
Thermal correction to Energy=	0.228992
Thermal correction to Enthalpy=	0.229936
Thermal correction to Gibbs Free Energy=	0.176566
Sum of electronic and zero-point Energies=	-677.074378
Sum of electronic and thermal Energies=	-677.061167
Sum of electronic and thermal Enthalpies=	-677.060223
Sum of electronic and thermal Free Energies=	-677.113593

TS for 4-CH₂NH₂[9MG + H$_{N7}$]$^{•+}$

C1 0.174340 1.094301 -0.029437
C2 0.538927 -0.268496 -0.140408
C3 -1.568043 -0.865855 -0.659363
C4 -1.185336 1.513874 -0.074438
C5 2.350197 1.024743 -0.384058
N6 -0.313940 -1.228355 -0.638342
N7 1.914933 -0.228012 -0.506733
N8 -2.522324 -1.770841 -0.997000
H9 -3.410647 -1.463275 -1.361243
H10 -2.181363 -2.654478 -1.345230
N11 1.338542 1.837407 -0.089436
N12 -0.502883 -0.978595 2.417148
H13 -1.114258 -1.763218 2.257202
C14 0.716612 -0.924290 1.821639
H15 1.395817 2.844119 -0.004785
H16 1.393326 -0.166652 2.212607
H17 1.157767 -1.892675 1.600706
C18 2.709178 -1.397360 -0.859739
H19 3.599874 -1.078288 -1.400209
H20 3.000706 -1.946727 0.038172
H21 2.099613 -2.036714 -1.498505
H22 3.376221 1.336984 -0.513096
N23 -2.018645 0.396040 -0.316155
H24 -3.007562 0.610431 -0.350143
O25 -1.634472 2.638888 0.051266
H26 -0.918754 -0.155618 2.822849

Zero-point correction=	0.213417 (Hartree/Particle)
Thermal correction to Energy=	0.226904
Thermal correction to Enthalpy=	0.227848
Thermal correction to Gibbs Free Energy=	0.173563
Sum of electronic and zero-point Energies=	-677.065176
Sum of electronic and thermal Energies=	-677.051689
Sum of electronic and thermal Enthalpies=	-677.050745
Sum of electronic and thermal Free Energies=	-677.105031

5-CH₂NH₂[9MG + H$_{N7}$]$^{•+}$

C1 -0.240850 -0.902069 0.032602
C2 -0.622833 0.536583 0.169874
C3 1.437565 1.324368 -0.188204
C4 0.973888 -0.994065 -0.878978
C5 -2.453322 -0.531714 -0.457056
N6 0.215200 1.573811 0.222670
N7 -1.986784 0.639692 -0.029668
N8 2.395493 2.261194 -0.079228
H9 3.355273 2.098697 -0.334376
H10 2.142577 3.156925 0.308191
N11 -1.469438 -1.435259 -0.542753
N12 1.155946 -1.116235 2.179023
H13 1.065459 -0.166174 2.509968
C14 0.065188 -1.621773 1.412439
H15 -1.591720 -2.394559 -0.832643
H16 0.237322 -2.679161 1.184275
H17 -0.860502 -1.550726 1.994201
C18 -2.737807 1.889132 0.067639
H19 -3.792458 1.683522 -0.110095
H20 -2.603547 2.308412 1.064648
H21 -2.361786 2.592536 -0.676342
H22 -3.486544 -0.715801 -0.711987
N23 1.795945 0.122958 -0.792696
H24 2.654127 0.063426 -1.328434
O25 1.235675 -1.950581 -1.568457
H26 2.080885 -1.318352 1.828920

Zero-point correction=	0.214696 (Hartree/Particle)
Thermal correction to Energy=	0.228425
Thermal correction to Enthalpy=	0.229369
Thermal correction to Gibbs Free Energy=	0.174833
Sum of electronic and zero-point Energies=	-677.085565
Sum of electronic and thermal Energies=	-677.071836
Sum of electronic and thermal Enthalpies=	-677.070892
Sum of electronic and thermal Free Energies=	-677.125427

TS for 5-CH₂NH₂[9MG + H$_{N7}$]$^{•+}$

C1 -0.251074 -0.780103 -0.322541
C2 -0.731474 0.516655 -0.043449
C3 1.263456 1.482092 -0.209619
C4 1.058406 -0.869311 -0.957148
C5 -2.484362 -0.786958 -0.362997
N6 0.005975 1.625744 0.140173
N7 -2.102209 0.443545 0.008147
N8 2.129880 2.498049 -0.024327
H9 3.064880 2.488144 -0.396331
H10 1.749578 3.381493 0.277577
N11 -1.393211 -1.516888 -0.654661
N12 1.496041 -1.098253 2.111894
H13 1.410424 -0.408282 2.839402
C14 0.385495 -1.675332 1.559624
H15 -1.398783 -2.478426 -0.962772
H16 0.521107 -2.676000 1.156649
H17 -0.555560 -1.485340 2.069533

C18 -2.982492 1.567589 0.317613
H19 -4.009770 1.209118 0.365861
H20 -2.691412 1.993951 1.277284
H21 -2.885798 2.325402 -0.460566
H22 -3.506447 -1.118046 -0.452773
N23 1.770047 0.322435 -0.772350
H24 2.723518 0.297020 -1.112867
O25 1.535589 -1.831406 -1.519777
H26 2.400160 -1.528003 2.006197

Zero-point correction=	0.211560 (Hartree/Particle)
Thermal correction to Energy=	0.225917
Thermal correction to Enthalpy=	0.226861
Thermal correction to Gibbs Free Energy=	0.169987
Sum of electronic and zero-point Energies=	-677.067762
Sum of electronic and thermal Energies=	-677.053405
Sum of electronic and thermal Enthalpies=	-677.052461
Sum of electronic and thermal Free Energies=	-677.109335

8-CH_2NH_2[9MG + H_{N7}]$^{•+}$

C1 0.175360 -0.837013 -0.351914
C2 0.087966 0.577920 -0.263434
C3 2.270146 0.792635 0.207534
C4 1.427752 -1.503572 -0.141045
C5 -2.019931 -0.249013 -0.715345
N6 1.098775 1.396366 0.011578
N7 -1.175907 0.929698 -0.522861
N8 3.331387 1.553225 0.484459
H9 4.254673 1.180954 0.638017
H10 3.198722 2.552625 0.524854
N11 -1.036228 -1.321654 -0.669210
N12 -2.637141 -0.413111 1.741635
H13 -2.225994 0.446353 2.077495
C14 -3.104558 -0.394061 0.390473
H15 -1.251209 -2.301748 -0.789926
H16 -3.657347 -1.315185 0.177428
H17 -3.817464 0.426443 0.256307
C18 -1.698243 2.283251 -0.561799
H19 -2.243874 2.439922 -1.495136
H20 -2.364176 2.468606 0.285803
H21 -0.858196 2.975225 -0.513019
H22 -2.503071 -0.207734 -1.701262
N23 2.446851 -0.562232 0.144705
H24 3.360060 -0.972487 0.302596
O25 1.645974 -2.696606 -0.185844
H26 -2.085016 -1.217060 2.004635

Zero-point correction=	0.215846 (Hartree/Particle)
Thermal correction to Energy=	0.229412
Thermal correction to Enthalpy=	0.230356
Thermal correction to Gibbs Free Energy=	0.175464
Sum of electronic and zero-point Energies=	-677.114774
Sum of electronic and thermal Energies=	-677.101208
Sum of electronic and thermal Enthalpies=	-677.100263
Sum of electronic and thermal Free Energies=	-677.155155

TS for 8-CH_2NH_2[9MG + H_{N7}]$^{•+}$

C1 -0.189270 0.838285 -0.517655
C2 -0.056556 -0.528010 -0.429890
C3 -2.148932 -0.860920 0.242223
C4 -1.421297 1.478002 -0.175998
C5 1.899825 0.328891 -1.036259
N6 -0.989271 -1.417740 -0.059297
N7 1.234327 -0.832110 -0.811493
N8 -3.168666 -1.645252 0.632135
H9 -4.099371 -1.296243 0.788816
H10 -3.024702 -2.642656 0.618935
N11 1.025768 1.344528 -0.937792
N12 2.100231 0.258629 2.013080
H13 2.055160 -0.616708 2.510898
C14 3.107171 0.485441 1.115223
H15 1.222856 2.316386 -1.128088
H16 3.428832 1.511356 0.974562
H17 3.843539 -0.300141 0.982181
C18 1.827710 -2.159746 -0.797266
H19 2.570299 -2.234657 -1.591730
H20 2.297701 -2.350520 0.170780
H21 1.036728 -2.889574 -0.964572
H22 2.850164 0.389130 -1.542704
N23 -2.370246 0.493790 0.195833
H24 -3.273839 0.873037 0.453968
O25 -1.688192 2.662378 -0.172764
H26 1.661252 1.036995 2.479528

Zero-point correction=	0.212061 (Hartree/Particle)
Thermal correction to Energy=	0.226313
Thermal correction to Enthalpy=	0.227257
Thermal correction to Gibbs Free Energy=	0.170516
Sum of electronic and zero-point Energies=	-677.084031
Sum of electronic and thermal Energies=	-677.069779
Sum of electronic and thermal Enthalpies=	-677.068835
Sum of electronic and thermal Free Energies=	-677.125576

[9MG + H_{O6}]$^{+}$···$^{•}CH_2NH_2$

C1 0.750094 0.936617 -0.010615
C2 1.519537 -0.243471 -0.005642
C3 -0.231334 -1.614269 -0.011646
C4 -0.623658 0.779296 -0.012724
C5 2.760485 1.558613 -0.004211
N6 1.076773 -1.508387 -0.006699
N7 2.807099 0.170575 -0.005212
N8 -0.811267 -2.828723 -0.004206
H9 -1.805047 -2.968414 -0.071286
H10 -0.206410 -3.634883 -0.024999
N11 1.558347 2.053017 -0.009726
N12 -4.131525 1.023359 0.021581
H13 -4.514622 1.474821 -0.803471
C14 -4.223495 -0.375272 0.020537
H15 -4.430438 -0.867935 0.963259
H16 -4.463548 -0.864273 -0.916209
C17 3.992840 -0.674486 0.039851
H18 4.818956 -0.152055 -0.442403
H19 4.254553 -0.904879 1.074429
H20 3.787523 -1.599953 -0.497882
H21 3.672786 2.140464 -0.003381
N22 -1.074153 -0.517494 -0.012830
H23 -2.090311 -0.670695 -0.003760
O24 -1.468718 1.766094 -0.013057
H25 -4.486441 1.471462 0.860969
H26 -2.430995 1.498996 -0.004474

Zero-point correction=	0.212438 (Hartree/Particle)
Thermal correction to Energy=	0.227016
Thermal correction to Enthalpy=	0.227961
Thermal correction to Gibbs Free Energy=	0.169877
Sum of electronic and zero-point Energies=	-677.080833
Sum of electronic and thermal Energies=	-677.066254
Sum of electronic and thermal Enthalpies=	-677.065310
Sum of electronic and thermal Free Energies=	-677.123394

TS for [9MG + H_{O6}]$^{+}$···$^{•}CH_2NH_2$

C1 -0.250371 0.162075 -0.021580
C2 1.065305 0.629766 0.008756
C3 2.010058 -1.378880 -0.022414
C4 -0.442403 -1.234816 -0.037905
C5 -0.369285 2.282527 0.010420
N6 2.204485 -0.080083 0.009721
N7 0.971498 1.988288 0.031957
N8 3.062600 -2.215556 -0.033056
H9 3.984338 -1.808135 -0.020542

N10 -1.137585 1.224792 -0.022587
C11 2.089520 2.921480 0.069294
H12 2.677183 2.758229 0.973659
H13 2.722211 2.774269 -0.806962
H14 1.695342 3.937277 0.068574
H15 -0.719022 3.305908 0.018623
N16 0.751012 -1.942410 -0.042774
H17 0.641644 -2.949770 -0.052304
O18 -1.502617 -1.913925 -0.049567
H19 -2.679118 -1.253718 0.218383
H20 -3.406289 -0.210914 1.491598
C21 -3.728899 -0.606954 0.524378
N22 -3.857775 0.364624 -0.435447
H23 -3.107585 1.061126 -0.472812
H24 -4.418060 0.205499 -1.262401
H25 -4.550313 -1.319974 0.567050
H26 2.977414 -3.217162 -0.066723

Zero-point correction=	0.208905 (Hartree/Particle)
Thermal correction to Energy=	0.222613
Thermal correction to Enthalpy=	0.223557
Thermal correction to Gibbs Free Energy=	0.167316
Sum of electronic and zero-point Energies=	-677.090668
Sum of electronic and thermal Energies=	-677.076960
Sum of electronic and thermal Enthalpies=	-677.076016
Sum of electronic and thermal Free Energies=	-677.132257

2-CH_2NH_2[9MG + H_{O6}]$^{•+}$

C1 1.127406 0.852438 -0.151979
C2 0.769946 -0.491672 0.138442
C3 -1.505020 -0.034142 0.342959
C4 0.119002 1.838604 -0.021372
C5 2.918268 -0.241484 -0.254421
N6 -0.402530 -0.969946 0.426963
O7 0.317963 3.125173 -0.123645
N8 1.976109 -1.168750 0.061121
N9 -2.413557 -0.107587 1.441818
H10 -1.932113 -0.166605 2.333440
H11 -3.011860 -0.921851 1.347769
N12 2.445981 0.992786 -0.387035
H13 -3.010190 0.463854 -1.136480
H14 -2.360729 -2.409504 -0.913832
H15 -3.854427 -1.652152 -0.706211
C16 2.164581 -2.596898 0.285234
H17 3.207427 -2.845295 0.091532
H18 1.914433 -2.842651 1.318237
H19 1.521798 -3.161116 -0.391477
H20 3.958868 -0.508371 -0.383451
N21 -1.085324 1.402428 0.275547
H22 -1.824304 2.073160 0.465948
C23 -2.270608 -0.332028 -1.010634
H24 -1.524524 -0.229825 -1.807809
N25 -2.923593 -1.590493 -1.088504
H26 1.250858 3.320991 -0.307261

Zero-point correction=	0.215760 (Hartree/Particle)
Thermal correction to Energy=	0.228853
Thermal correction to Enthalpy=	0.229797
Thermal correction to Gibbs Free Energy=	0.176250
Sum of electronic and zero-point Energies=	-677.069467
Sum of electronic and thermal Energies=	-677.056374
Sum of electronic and thermal Enthalpies=	-677.055430
Sum of electronic and thermal Free Energies=	-677.108977

TS for 2-CH_2NH_2[9MG + H_{O6}]$^{•+}$

C1 1.102281 0.868842 -0.156015
C2 0.790444 -0.447887 0.250377
C3 -1.358408 0.021584 0.683000
C4 0.118583 1.820407 0.006758
C5 2.884513 -0.224054 -0.384557
N6 -0.366190 -0.893229 0.722160
O7 0.209640 3.110853 -0.222639
N8 1.955451 -1.135461 0.093120
N9 -2.546263 -0.179729 1.388108
H10 -2.585364 0.325636 2.268039
H11 -2.690826 -1.167916 1.558156
N12 2.419180 0.981224 -0.546030
H13 -2.859818 0.425898 -1.360465
H14 -2.305329 -2.496318 -1.409040
H15 -3.780412 -1.720825 -0.987736
C16 2.168475 -2.543333 0.395601
H17 3.227408 -2.769837 0.274727
H18 1.869414 -2.746991 1.424743
H19 1.584623 -3.167151 -0.283508
H20 3.901857 -0.528647 -0.592111
N21 -1.073032 1.384662 0.474511
H22 -1.831315 2.053381 0.550341
C23 -2.206517 -0.439707 -1.320306
H24 -1.267062 -0.390315 -1.863854
N25 -2.823366 -1.640218 -1.292816
H26 1.103014 3.340404 -0.520714

Zero-point correction=	0.212714 (Hartree/Particle)
Thermal correction to Energy=	0.226589
Thermal correction to Enthalpy=	0.227533
Thermal correction to Gibbs Free Energy=	0.171057
Sum of electronic and zero-point Energies=	-677.056949
Sum of electronic and thermal Energies=	-677.043074
Sum of electronic and thermal Enthalpies=	-677.042130
Sum of electronic and thermal Free Energies=	-677.098607

3-CH_2NH_2[9MG + H_{O6}]$^{•+}$

C1 1.498632 -0.018085 0.209847
C2 0.261848 -0.609905 0.113754
C3 -0.814755 1.462014 -0.136231
C4 1.597809 1.397295 0.254833
C5 1.857123 -2.084950 -0.070229
N6 -0.948724 0.111312 0.062017
N7 0.491884 -1.946358 -0.102789
N8 -1.924898 2.181986 -0.301594
H9 -1.903342 3.188859 -0.335566
H10 -2.788601 1.666613 -0.485946
N11 2.492223 -0.951189 0.106187
N12 -3.314219 -0.228258 -0.147759
H13 -3.316247 -0.905945 -0.903537
C14 -2.173072 -0.380557 0.731363
H15 -2.352256 0.209230 1.636573
H16 -1.959379 -1.406061 1.040105
C17 -0.461654 -3.019233 -0.334004
H18 0.068791 -3.860447 -0.779782
H19 -0.924060 -3.353074 0.598368
H20 -1.227365 -2.691224 -1.040269
H21 2.322967 -3.054684 -0.177292
N22 0.388244 2.039125 -0.120020
H23 0.465726 3.016731 -0.375472
O24 2.679842 2.125473 -0.077794
H25 -4.185728 -0.318169 0.364465
H26 3.473602 1.578590 -0.007598

Zero-point correction=	0.216060 (Hartree/Particle)
Thermal correction to Energy=	0.229501
Thermal correction to Enthalpy=	0.230445
Thermal correction to Gibbs Free Energy=	0.176138
Sum of electronic and zero-point Energies=	-677.060044
Sum of electronic and thermal Energies=	-677.046603
Sum of electronic and thermal Enthalpies=	-677.045659
Sum of electronic and thermal Free Energies=	-677.099966

TS for 3-CH_2NH_2[9MG + H_{O6}]$^{•+}$

C1 1.447562 0.287376 0.175705

C2 0.411789 -0.549420 -0.234027
C3 -1.026023 1.136682 -0.597723
C4 1.191709 1.653924 0.202868
C5 2.274199 -1.654500 0.092768
N6 -0.894900 -0.201280 -0.478370
N7 0.958991 -1.801599 -0.285289
N8 -2.244231 1.619425 -0.961974
H9 -2.350149 2.586640 -1.229759
H10 -2.823954 0.965440 -1.467886
N11 2.607807 -0.420281 0.375518
N12 -3.374859 -0.754158 0.906351
H13 -3.843488 -1.524904 0.459017
C14 -2.048038 -0.783241 1.140381
H15 -1.662228 -0.037615 1.829227
H16 -1.587526 -1.763687 1.181858
C17 0.288411 -3.035097 -0.665709
H18 0.977949 -3.664070 -1.229339
H19 -0.056897 -3.579589 0.217110
H20 -0.562005 -2.786660 -1.301686
H21 2.943819 -2.502929 0.132556
N22 -0.063235 2.018940 -0.225379
H23 -0.229944 3.015305 -0.316014
O24 2.007335 2.665514 0.492078
H25 -3.915334 0.078427 1.074579
H26 2.886413 2.327861 0.712143

Zero-point correction=	0.211770 (Hartree/Particle)
Thermal correction to Energy=	0.225913
Thermal correction to Enthalpy=	0.226857
Thermal correction to Gibbs Free Energy=	0.170593
Sum of electronic and zero-point Energies=	-677.041633
Sum of electronic and thermal Energies=	-677.027491
Sum of electronic and thermal Enthalpies=	-677.026547
Sum of electronic and thermal Free Energies=	-677.082811

4-CH$_2$NH$_2$[9MG + H$_{O6}$]$^{\bullet+}$

C1 0.115012 1.094071 0.060894
C2 0.620374 -0.305872 0.080923
C3 -1.443394 -1.003807 -0.650141
C4 -1.255140 1.298973 0.016151
C5 2.148591 1.267870 -0.477418
N6 -0.181040 -1.238090 -0.647546
N7 1.953878 -0.048162 -0.473415
N8 -2.351551 -1.918979 -1.083938
H9 -3.197326 -1.610066 -1.540316
H10 -1.951343 -2.769886 -1.452927
N11 1.076303 2.008961 -0.171079
N12 -0.324738 -1.109165 2.329386
H13 -0.890551 -1.893705 2.043825
C14 0.844935 -0.851504 1.576311
H15 1.478114 -0.122351 2.092354
H16 1.405695 -1.783113 1.456447
C17 2.926071 -1.086370 -0.771643
H18 3.705760 -0.671857 -1.410811
H19 3.378242 -1.473310 0.145574
H20 2.415339 -1.893994 -1.298393
H21 3.104827 1.702913 -0.743393
N22 -2.024131 0.198849 -0.174836
H23 -3.030800 0.300673 -0.136260
O24 -1.885001 2.452344 0.066598
H25 -0.860014 -0.317654 2.651105
H26 -1.257667 3.179535 0.199548

Zero-point correction=	0.215706 (Hartree/Particle)
Thermal correction to Energy=	0.228852
Thermal correction to Enthalpy=	0.229796
Thermal correction to Gibbs Free Energy=	0.176823
Sum of electronic and zero-point Energies=	-677.067527
Sum of electronic and thermal Energies=	-677.054381
Sum of electronic and thermal Enthalpies=	-677.053437
Sum of electronic and thermal Free Energies=	-677.106410

TS for 4-CH$_2$NH$_2$[9MG + H$_{O6}$]$^{\bullet+}$

C1 0.185260 1.095141 -0.091496
C2 0.588778 -0.275819 -0.211969
C3 -1.506244 -0.971028 -0.646453
C4 -1.152123 1.360268 -0.125134
C5 2.278779 1.143053 -0.348738
N6 -0.238440 -1.271007 -0.662277
N7 1.942715 -0.176295 -0.514928
N8 -2.443059 -1.916259 -0.919244
H9 -3.334473 -1.650548 -1.309844
H10 -2.074407 -2.799807 -1.240356
N11 1.274410 1.940717 -0.091962
N12 -0.469976 -0.898718 2.424393
H13 -0.978547 -1.767864 2.435085
C14 0.753798 -0.809292 1.854951
H15 1.373728 0.028211 2.163398
H16 1.250000 -1.754161 1.657246
C17 2.831256 -1.284474 -0.818097
H18 3.722652 -0.897177 -1.311474
H19 3.126960 -1.814059 0.092047
H20 2.318781 -1.974109 -1.489886
H21 3.306765 1.469887 -0.444359
N22 -1.991556 0.290046 -0.328521
H23 -2.987128 0.470699 -0.314631
O24 -1.765007 2.529905 -0.031431
H25 -0.883480 -0.117043 2.905811
H26 -1.116482 3.240245 0.077816

Zero-point correction=	0.212904 (Hartree/Particle)
Thermal correction to Energy=	0.226599
Thermal correction to Enthalpy=	0.227544
Thermal correction to Gibbs Free Energy=	0.172846
Sum of electronic and zero-point Energies=	-677.061970
Sum of electronic and thermal Energies=	-677.048275
Sum of electronic and thermal Enthalpies=	-677.047331
Sum of electronic and thermal Free Energies=	-677.102029

5-CH$_2$NH$_2$[9MG + H$_{O6}$]$^{\bullet+}$

C1 -0.322104 -0.876737 0.163912
C2 -0.591852 0.597176 0.126189
C3 1.527300 1.163925 -0.278127
C4 0.776088 -1.037462 -0.819882
C5 -2.432234 -0.545588 -0.264287
N6 0.303280 1.550485 0.100542
N7 -1.935977 0.731415 0.009973
N8 2.574191 1.994045 -0.116832
H9 3.494256 1.788611 -0.471125
H10 2.378773 2.938159 0.179789
N11 -1.569833 -1.495482 -0.256350
N12 1.196743 -0.860833 2.247163
H13 1.167441 0.107553 2.527506
C14 0.038879 -1.402381 1.623684
H15 0.134368 -2.487813 1.530041
H16 -0.846743 -1.201177 2.235316
C17 -2.650462 1.991859 -0.147194
H18 -3.716153 1.809906 -0.010299
H19 -2.306916 2.693382 0.612999
H20 -2.470420 2.414712 -1.138587
H21 -3.486059 -0.673057 -0.486196
N22 1.723810 -0.059688 -0.864705
H23 2.534166 -0.220993 -1.453259
O24 0.849644 -1.973710 -1.753617
H25 2.095096 -1.155071 1.897409
H26 0.105840 -2.588873 -1.658958

Zero-point correction=	0.213892 (Hartree/Particle)
Thermal correction to Energy=	0.227751
Thermal correction to Enthalpy=	0.228696

Thermal correction to Gibbs Free Energy= 0.174049
Sum of electronic and zero-point Energies= -677.060076
Sum of electronic and thermal Energies= -677.046217
Sum of electronic and thermal Enthalpies= -677.045273
Sum of electronic and thermal Free Energies= -677.099920

TS for 5-$CH_2NH_2[9MG + H_{O6}]^{\bullet+}$
C1 -0.277451 -0.812847 -0.250277
C2 -0.663813 0.559918 -0.057476
C3 1.410104 1.368549 -0.186192
C4 0.994088 -0.900126 -0.849855
C5 -2.391902 -0.728060 -0.492856
N6 0.129500 1.603625 0.102718
N7 -2.017968 0.558609 -0.105330
N8 2.320350 2.336542 0.030485
H9 3.286862 2.254908 -0.237625
H10 1.975958 3.241642 0.310620
N11 -1.409979 -1.553179 -0.648036
N12 1.069436 -1.007720 2.257293
H13 1.010647 -0.261526 2.932577
C14 -0.101809 -1.498352 1.714994
H15 -0.117050 -2.560521 1.489001
H16 -1.031198 -1.101687 2.118513
C17 -2.879702 1.725209 0.029321
H18 -3.916328 1.407701 -0.079688
H19 -2.740422 2.176991 1.012314
H20 -2.638758 2.457589 -0.743360
H21 -3.436251 -0.964507 -0.655457
N22 1.821745 0.185174 -0.724055
H23 2.765502 0.081117 -1.077740
O24 1.529020 -1.949993 -1.464925
H25 1.826339 -1.652125 2.425776
H26 0.853857 -2.626733 -1.613024

Zero-point correction= 0.211609 (Hartree/Particle)
Thermal correction to Energy= 0.225781
Thermal correction to Enthalpy= 0.226725
Thermal correction to Gibbs Free Energy= 0.170740
Sum of electronic and zero-point Energies= -677.048003
Sum of electronic and thermal Energies= -677.033832
Sum of electronic and thermal Enthalpies= -677.032888
Sum of electronic and thermal Free Energies= -677.088872

7-$CH_2NH_2[9MG + H_{O6}]^{\bullet+}$
C1 -0.188310 -0.387959 -0.059362
C2 0.469778 0.834629 -0.031362
C3 2.513791 -0.060219 -0.014431
C4 0.568887 -1.565175 -0.181248
C5 -1.702774 1.215854 0.007097
N6 1.805674 1.049832 -0.041943
N7 -0.496518 1.808533 0.002778
N8 3.857250 0.021746 0.077608
H9 4.458509 -0.753984 -0.145288
H10 4.257677 0.946754 0.060005
N11 -1.539226 -0.114855 -0.029581
N12 -3.901851 -0.461956 0.030662
H13 -4.481539 -0.584506 -0.786695
C14 -2.640228 -1.120227 -0.035051
H15 -2.456586 -1.778807 0.816163
H16 -2.515605 -1.701024 -0.952909
C17 -0.231619 3.243041 0.048029
H18 -1.181847 3.774801 0.068767
H19 0.345139 3.474935 0.943740
H20 0.338071 3.531840 -0.835475
H21 -2.659916 1.708428 0.046869
N22 1.963280 -1.315873 -0.040481
H23 2.552161 -2.121581 0.116156
O24 0.129814 -2.785673 0.235142
H25 -4.427640 -0.651751 0.871404
H26 0.175666 -3.431386 -0.479204

Zero-point correction= 0.214530 (Hartree/Particle)
Thermal correction to Energy= 0.228975
Thermal correction to Enthalpy= 0.229919
Thermal correction to Gibbs Free Energy= 0.173003
Sum of electronic and zero-point Energies= -677.082293
Sum of electronic and thermal Energies= -677.067848
Sum of electronic and thermal Enthalpies= -677.066904
Sum of electronic and thermal Free Energies= -677.123819

TS for 7-$CH_2NH_2[9MG + H_{O6}]^{\bullet+}$
C1 -0.139149 -0.455220 -0.449801
C2 0.316084 0.854406 -0.141743
C3 2.397162 0.194171 0.350724
C4 0.790204 -1.448992 -0.393172
C5 -1.790352 0.867916 -0.806558
N6 1.547149 1.196304 0.275795
N7 -0.732333 1.660541 -0.360993
N8 3.656040 0.420953 0.764327
H9 4.358570 -0.295888 0.830504
H10 3.912658 1.371465 0.979743
N11 -1.483155 -0.442703 -0.816068
N12 -3.398363 -0.575930 1.299163
H13 -4.322928 -0.384857 0.949619
C14 -2.582939 -1.444304 0.676682
H15 -1.715518 -1.783381 1.233570
H16 -3.045534 -2.136100 -0.014153
C17 -0.742234 3.111437 -0.247084
H18 -1.557674 3.424219 0.407145
H19 0.209533 3.428860 0.177542
H20 -0.868079 3.560192 -1.234316
H21 -2.736870 1.297964 -1.091709
N22 2.061545 -1.100588 0.023618
H23 2.751766 -1.840017 0.048172
O24 0.653294 -2.739916 -0.681291
H25 -3.080012 -0.017750 2.073881
H26 -0.181101 -2.891914 -1.144850
Zero-point correction= 0.210792 (Hartree/Particle)
Thermal correction to Energy= 0.225438
Thermal correction to Enthalpy= 0.226382
Thermal correction to Gibbs Free Energy= 0.167986
Sum of electronic and zero-point Energies= -677.048282
Sum of electronic and thermal Energies= -677.033636
Sum of electronic and thermal Enthalpies= -677.032692
Sum of electronic and thermal Free Energies= -677.091088

8-$CH_2NH_2[9MG + H_{O6}]^{\bullet+}$
C1 -0.129585 0.817831 -0.353689
C2 -0.050386 -0.622455 -0.257809
C3 -2.235555 -0.843613 0.213329
C4 -1.378697 1.378762 -0.148837
C5 1.983526 0.301509 -0.720828
N6 -1.066860 -1.439654 0.019927
N7 1.203166 -0.941786 -0.511815
N8 -3.304180 -1.592787 0.499227
H9 -4.226476 -1.217855 0.651006
H10 -3.174774 -2.592334 0.549225
N11 1.032586 1.386586 -0.649930
N12 2.685806 0.474139 1.715331
H13 2.287531 -0.390782 2.054746
C14 3.106523 0.459159 0.344279
H15 3.621095 1.394509 0.109979
H16 3.831369 -0.347631 0.193352
C17 1.767745 -2.277708 -0.557410
H18 2.289959 -2.420998 -1.505987
H19 2.471147 -2.423635 0.266598
H20 0.959011 -3.003429 -0.474124
H21 2.445757 0.272585 -1.717480
N22 -2.413754 0.518213 0.137679
H23 -3.325626 0.933043 0.288040

O24 -1.710693 2.652615 -0.194253
H25 2.107620 1.264480 1.965720
H26 -0.939840 3.197234 -0.414200

Zero-point correction=	0.215891 (Hartree/Particle)
Thermal correction to Energy=	0.229164
Thermal correction to Enthalpy=	0.230109
Thermal correction to Gibbs Free Energy=	0.176345
Sum of electronic and zero-point Energies=	-677.091941
Sum of electronic and thermal Energies=	-677.078668
Sum of electronic and thermal Enthalpies=	-677.077724
Sum of electronic and thermal Free Energies=	-677.131487

TS for 8-CH_2NH_2[9MG + H_{O6}]$^{•+}$

C1 -0.117198 0.827224 -0.489086
C2 0.018037 -0.581775 -0.395405
C3 -2.098364 -0.918187 0.234812
C4 -1.357648 1.334827 -0.206696
C5 1.899419 0.406891 -0.982080
N6 -0.926120 -1.459410 -0.041780
N7 1.281418 -0.838627 -0.773065
N8 -3.117959 -1.705026 0.620992
H9 -4.059755 -1.371230 0.741481
H10 -2.959955 -2.700835 0.620130
N11 1.044877 1.418804 -0.902563
N12 2.019185 0.352561 1.972373
H13 1.997535 -0.496915 2.514233
C14 3.013936 0.545636 1.052791
H15 3.334716 1.563731 0.870074
H16 3.747574 -0.246500 0.942429
C17 1.931488 -2.135993 -0.788814
H18 2.680135 -2.152274 -1.581265
H19 2.411580 -2.336156 0.173071
H20 1.182015 -2.903066 -0.982651
H21 2.843684 0.454580 -1.507945
N22 -2.329163 0.435484 0.162686
H23 -3.242012 0.817139 0.380201
O24 -1.756398 2.594173 -0.234817
H25 1.607815 1.154361 2.423260
H26 -1.035837 3.165543 -0.538109

Zero-point correction=	0.211928 (Hartree/Particle)
Thermal correction to Energy=	0.225989
Thermal correction to Enthalpy=	0.226933
Thermal correction to Gibbs Free Energy=	0.171110
Sum of electronic and zero-point Energies=	-677.065761
Sum of electronic and thermal Energies=	-677.051700
Sum of electronic and thermal Enthalpies=	-677.050756
Sum of electronic and thermal Free Energies=	-677.106580

[9MG + H_{N3}]$^{+}$···•CH_2NH_2

C1 -1.668352 -0.592481 0.086076
C2 -0.302310 -0.577951 -0.092334
C3 -0.161703 1.748293 -0.163432
C4 -2.404760 0.645728 0.152191
C5 -1.049574 -2.612934 0.015301
N6 0.470488 0.565575 -0.208212
N7 0.107261 -1.867336 -0.140513
N8 0.542691 2.881256 -0.265648
H9 0.095277 3.783498 -0.224818
H10 1.529755 2.856835 -0.468329
N11 -2.119453 -1.882916 0.153762
N12 4.037770 -0.190022 0.547035
H13 4.632735 -0.748303 -0.047329
C14 3.504521 0.980698 0.053812
H15 3.386282 1.788566 0.772203
H16 3.761649 1.236429 -0.970505
C17 1.450226 -2.371855 -0.384751
H18 1.455011 -3.443545 -0.187806
H19 1.735858 -2.200109 -1.425675
H20 2.165055 -1.885584 0.282872
H21 -1.015261 -3.693689 0.017790
N22 -1.492385 1.777507 -0.011936
H23 -1.972642 2.670931 -0.002689
O24 -3.571090 0.878526 0.298944
H25 4.251159 -0.256582 1.531395
H26 1.510948 0.557923 -0.191263

Zero-point correction=	0.211373 (Hartree/Particle)
Thermal correction to Energy=	0.226548
Thermal correction to Enthalpy=	0.227492
Thermal correction to Gibbs Free Energy=	0.167620
Sum of electronic and zero-point Energies=	-677.065097
Sum of electronic and thermal Energies=	-677.049921
Sum of electronic and thermal Enthalpies=	-677.048977
Sum of electronic and thermal Free Energies=	-677.108850

TS for [9MG + H_{N3}]$^{+}$···•CH_2NH_2

C1 0.210768 1.367604 0.151399
C2 -0.423508 0.287390 -0.431512
C3 1.464498 -0.965255 -0.662417
C4 1.644723 1.327891 0.331204
C5 -1.814549 1.927747 -0.079430
N6 0.125556 -0.919768 -0.794226
N7 -1.729399 0.645106 -0.580815
N8 2.083954 -2.113133 -0.976218
H9 3.083013 -2.219092 -0.892071
H10 1.589798 -2.785371 -1.543458
N11 -0.673431 2.388791 0.361084
N12 -1.175943 -1.610395 2.031133
H13 -2.033877 -1.081506 2.081329
C14 -0.946785 -2.524754 1.030968
H15 -0.173951 -3.256492 1.270651
H16 -1.854241 -2.953546 0.603142
C17 -2.786648 -0.131322 -1.210695
H18 -3.577508 0.547349 -1.529174
H19 -2.386785 -0.644976 -2.086150
H20 -3.206915 -0.863855 -0.516532
H21 -2.752225 2.466880 -0.078329
N22 2.173033 0.073249 -0.179551
H23 3.186360 0.031952 -0.157080
O24 2.412905 2.128127 0.791509
H25 -0.461406 -1.369076 2.702234
H26 -0.432885 -1.880058 0.006420

Zero-point correction=	0.207807 (Hartree/Particle)
Thermal correction to Energy=	0.221846
Thermal correction to Enthalpy=	0.222791
Thermal correction to Gibbs Free Energy=	0.166140
Sum of electronic and zero-point Energies=	-677.056688
Sum of electronic and thermal Energies=	-677.042649
Sum of electronic and thermal Enthalpies=	-677.041704
Sum of electronic and thermal Free Energies=	-677.098355

2-CH_2NH_2[9MG + H_{N3}]$^{•+}$

C1 -1.232918 0.903451 0.056418
C2 -0.816305 -0.463582 -0.022039
C3 1.551985 0.163608 -0.206426
C4 -0.221497 1.982655 0.145391
C5 -2.979087 -0.283568 0.049892
N6 0.441172 -0.832329 -0.073117
O7 -0.479616 3.157969 0.236524
N8 -1.958993 -1.204200 -0.018738
N9 2.083736 0.195992 -1.531338
H10 1.431883 0.535584 -2.229486
H11 2.506396 -0.675984 -1.828550
N12 -2.562132 0.978172 0.096378
H13 3.497917 0.333613 0.688058
H14 3.097583 -2.243657 1.374978
H15 3.736574 -1.851931 -0.067817

C16 -2.069441 -2.658540 -0.074027
H17 -3.125784 -2.922605 -0.106113
H18 -1.584958 -3.036334 -0.976604
H19 -1.620001 -3.103852 0.815856
H20 -4.016514 -0.591715 0.063755
N21 1.070371 1.479750 0.164046
H22 1.782985 2.200702 0.133484
C23 2.629591 -0.326780 0.785385
H24 2.222595 -0.212752 1.792628
N25 2.917960 -1.732366 0.518889
H26 0.789478 -1.793418 -0.052782

Zero-point correction= 0.215841 (Hartree/Particle)
Thermal correction to Energy= 0.229001
Thermal correction to Enthalpy= 0.229945
Thermal correction to Gibbs Free Energy= 0.175606
Sum of electronic and zero-point Energies= -677.069385
Sum of electronic and thermal Energies= -677.056225
Sum of electronic and thermal Enthalpies= -677.055280
Sum of electronic and thermal Free Energies= -677.109620

TS for 2-CH_2NH_2[9MG + H_{N3}]$^{•+}$
C1 -1.081860 0.976384 0.128770
C2 -0.867759 -0.320306 -0.288712
C3 1.425885 0.041068 -0.694975
C4 -0.025453 1.965059 0.028421
C5 -2.906336 -0.059320 0.418533
N6 0.325530 -0.782279 -0.797292
O7 -0.025360 3.134834 0.306247
N8 -2.030336 -0.995886 -0.108300
N9 2.601312 -0.276621 -1.347643
H10 2.773971 0.215670 -2.216119
H11 2.843461 -1.256768 -1.382652
N12 -2.368560 1.116662 0.575545
H13 2.868640 0.268194 1.370593
H14 1.965523 -2.521626 1.750860
H15 3.531239 -1.989929 1.318381
C16 -2.311848 -2.385332 -0.432524
H17 -3.354253 -2.591218 -0.191553
H18 -2.163648 -2.568041 -1.499784
H19 -1.681486 -3.054508 0.158897
H20 -3.927456 -0.325352 0.654839
N21 1.174105 1.368828 -0.502301
H22 1.973211 1.993342 -0.505786
C23 2.109587 -0.505538 1.351479
H24 1.154203 -0.297728 1.825361
N25 2.543993 -1.787686 1.371527
H26 0.443869 -1.723925 -1.139722

Zero-point correction= 0.212476 (Hartree/Particle)
Thermal correction to Energy= 0.226387
Thermal correction to Enthalpy= 0.227331
Thermal correction to Gibbs Free Energy= 0.171713
Sum of electronic and zero-point Energies= -677.045806
Sum of electronic and thermal Energies= -677.031895
Sum of electronic and thermal Enthalpies= -677.030951
Sum of electronic and thermal Free Energies= -677.086569

4-CH_2NH_2[9MG + H_{N3}]$^{•+}$
C1 0.147527 1.143205 0.030065
C2 0.647458 -0.277306 0.122819
C3 -1.593688 -0.911487 -0.636044
C4 -1.253760 1.448664 0.048583
C5 2.147556 1.225193 -0.656439
N6 -0.281400 -1.154886 -0.612163
N7 1.931231 -0.113510 -0.540103
N8 -2.446779 -1.829445 -1.103073
H9 -3.411908 -1.598606 -1.283851
H10 -2.137071 -2.747695 -1.380963
N11 1.121696 1.992414 -0.353729
N12 -0.320879 -0.893929 2.401823
H13 -0.973268 -1.636776 2.206437
C14 0.845014 -0.804823 1.586071
H15 1.571670 -0.132775 2.055537
H16 1.311273 -1.795301 1.522429
C17 2.977595 -1.124364 -0.578082
H18 3.750807 -0.795838 -1.273518
H19 3.429778 -1.288755 0.404450
H20 2.576384 -2.068783 -0.953951
H21 3.098865 1.607630 -1.007276
N22 -2.060278 0.261588 -0.181125
H23 -3.058463 0.435777 -0.168048
O24 -1.820371 2.506153 0.176807
H25 -0.764904 -0.026115 2.662698
H26 0.058470 -2.032095 -0.981644

Zero-point correction= 0.214719 (Hartree/Particle)
Thermal correction to Energy= 0.228280
Thermal correction to Enthalpy= 0.229224
Thermal correction to Gibbs Free Energy= 0.175224
Sum of electronic and zero-point Energies= -677.067214
Sum of electronic and thermal Energies= -677.053653
Sum of electronic and thermal Enthalpies= -677.052709
Sum of electronic and thermal Free Energies= -677.106710

TS for 4-CH_2NH_2[9MG + H_{N3}]$^{•+}$
C1 0.256439 1.128530 -0.167525
C2 0.607451 -0.235046 -0.208373
C3 -1.633525 -0.870874 -0.640350
C4 -1.111342 1.539826 -0.157790
C5 2.360081 1.060783 -0.411181
N6 -0.330498 -1.200384 -0.625515
N7 1.949363 -0.250822 -0.536470
N8 -2.556514 -1.815690 -0.867638
H9 -3.532154 -1.573649 -0.946342
H10 -2.295518 -2.741734 -1.168487
N11 1.384790 1.901929 -0.201059
N12 -0.596754 -0.653947 2.411129
H13 -1.003726 -1.484786 2.813430
C14 0.697276 -0.714606 1.941347
H15 1.356586 0.116734 2.175680
H16 1.150396 -1.703159 1.938578
C17 2.796180 -1.417772 -0.687563
H18 3.796127 -1.087443 -0.968580
H19 2.865365 -1.981738 0.247779
H20 2.419069 -2.062487 -1.485998
H21 3.405190 1.325455 -0.503123
N22 -1.990779 0.397534 -0.437111
H23 -2.968852 0.658252 -0.489237
O24 -1.633669 2.614976 -0.011314
H25 -0.893082 0.199018 2.863156
H26 -0.082673 -2.179668 -0.601074

Zero-point correction= 0.211627 (Hartree/Particle)
Thermal correction to Energy= 0.225774
Thermal correction to Enthalpy= 0.226719
Thermal correction to Gibbs Free Energy= 0.170412
Sum of electronic and zero-point Energies= -677.042366
Sum of electronic and thermal Energies= -677.028218
Sum of electronic and thermal Enthalpies= -677.027274
Sum of electronic and thermal Free Energies= -677.083581

5-CH_2NH_2[9MG + H_{N3}]$^{•+}$
C1 -0.282208 -0.959152 0.072988
C2 -0.716364 0.437770 0.432130
C3 1.382400 1.379383 -0.197340
C4 0.882074 -0.932957 -0.920232
C5 -2.371052 -0.666291 -0.544031
N6 0.202197 1.507006 0.412939
N7 -2.002224 0.579098 -0.053988

N8 2.327091 2.315434 -0.080684
H9 3.260046 2.162715 -0.431501
H10 2.149486 3.191865 0.384957
N11 -1.462146 -1.567982 -0.533279
N12 1.371712 -1.174421 1.895272
H13 1.194245 -0.858651 2.840264
C14 0.192723 -1.792686 1.297065
H15 0.394656 -2.803726 0.933661
H16 -0.649811 -1.849652 1.992054
C17 -2.859048 1.737420 0.106878
H18 -3.798624 1.556655 -0.415386
H19 -3.072855 1.922093 1.163905
H20 -2.387971 2.620234 -0.335109
H21 -3.380565 -0.822106 -0.909119
N22 1.608512 0.292386 -0.961590
H23 2.438998 0.256100 -1.541486
O24 1.240531 -1.838037 -1.615806
H25 2.152905 -1.817396 1.934528
H26 0.088110 2.264589 1.074238

Zero-point correction=	0.215213 (Hartree/Particle)
Thermal correction to Energy=	0.229026
Thermal correction to Enthalpy=	0.229971
Thermal correction to Gibbs Free Energy=	0.174664
Sum of electronic and zero-point Energies=	-677.058105
Sum of electronic and thermal Energies=	-677.044292
Sum of electronic and thermal Enthalpies=	-677.043348
Sum of electronic and thermal Free Energies=	-677.098655

TS for 5-CH_2NH_2[9MG + H_{N3}]$^{•+}$

C1 -0.253226 -0.784972 -0.336818
C2 -0.799357 0.458936 0.045257
C3 1.237501 1.608742 -0.118075
C4 1.076876 -0.749483 -0.948533
C5 -2.388071 -0.925445 -0.502196
N6 -0.052265 1.604532 0.245262
N7 -2.162794 0.344215 -0.000588
N8 2.036982 2.652620 0.179465
H9 3.001342 2.648400 -0.114011
H10 1.645255 3.556424 0.395090
N11 -1.311755 -1.597139 -0.767430
N12 1.588875 -1.525946 1.855032
H13 1.607098 -1.096634 2.766750
C14 0.380246 -1.939375 1.338499
H15 0.376806 -2.867032 0.774382
H16 -0.498452 -1.745851 1.947319
C17 -3.149287 1.343160 0.363668
H18 -4.141749 0.940020 0.163611
H19 -3.083924 1.584460 1.428670
H20 -3.020383 2.249420 -0.235942
H21 -3.398639 -1.284253 -0.650497
N22 1.732447 0.514768 -0.729541
H23 2.687026 0.528346 -1.069505
O24 1.674926 -1.606300 -1.540925
H25 2.406068 -2.077959 1.642504
H26 -0.428380 2.394317 0.752817

Zero-point correction=	0.210956 (Hartree/Particle)
Thermal correction to Energy=	0.225469
Thermal correction to Enthalpy=	0.226414
Thermal correction to Gibbs Free Energy=	0.169143
Sum of electronic and zero-point Energies=	-677.034060
Sum of electronic and thermal Energies=	-677.019546
Sum of electronic and thermal Enthalpies=	-677.018602
Sum of electronic and thermal Free Energies=	-677.075872

7-CH_2NH_2[9MG + H_{N3}]$^{•+}$

C1 0.370680 -0.211153 -0.161966
C2 -0.521920 0.874962 -0.408492
C3 -2.353839 -0.612999 -0.007329
C4 -0.028899 -1.528237 0.040039
C5 1.443014 1.669362 0.262873
N6 -1.898033 0.651663 -0.089206
N7 0.173037 2.020649 0.052071
N8 -3.672796 -0.834617 0.074094
H9 -4.038702 -1.771844 0.134909
H10 -4.328423 -0.078762 0.193042
N11 1.613933 0.362577 0.138899
N12 3.259608 -1.326637 -0.569122
H13 3.478300 -0.931976 -1.472753
C14 2.877676 -0.411101 0.426731
H15 2.694353 -0.942317 1.362427
H16 3.643355 0.352177 0.587836
C17 -0.322186 3.385648 -0.040392
H18 0.441764 4.073039 0.322473
H19 -0.563766 3.624931 -1.079308
H20 -1.208790 3.497359 0.587551
H21 2.240411 2.372999 0.459151
N22 -1.498347 -1.636120 -0.047244
H23 -1.832898 -2.580524 0.101375
O24 0.594473 -2.560566 0.278372
H25 2.658326 -2.140334 -0.625277
H26 -2.568681 1.348277 -0.383624

Zero-point correction=	0.216005 (Hartree/Particle)
Thermal correction to Energy=	0.229634
Thermal correction to Enthalpy=	0.230578
Thermal correction to Gibbs Free Energy=	0.176053
Sum of electronic and zero-point Energies=	-677.053703
Sum of electronic and thermal Energies=	-677.040074
Sum of electronic and thermal Enthalpies=	-677.039130
Sum of electronic and thermal Free Energies=	-677.093655

TS for 7-CH_2NH_2[9MG + H_{N3}]$^{•+}$

C1 -0.367439 0.065758 0.141534
C2 0.852723 0.771183 0.349184
C3 2.080415 -1.236721 -0.024063
C4 -0.409882 -1.315589 0.013975
C5 -0.769039 2.155184 -0.208763
N6 2.071578 0.103807 0.008518
N7 0.579214 2.086937 -0.059989
N8 3.245618 -1.897000 -0.128146
H9 3.265282 -2.900963 -0.207971
H10 4.112258 -1.408568 -0.285224
N11 -1.379021 1.002144 -0.103941
N12 -3.548511 -0.876464 0.463585
H13 -3.997939 -0.700097 1.352283
C14 -3.476039 0.038081 -0.439166
H15 -3.009300 -0.212827 -1.381662
H16 -4.031327 0.962824 -0.338016
C17 1.466506 3.221865 0.116245
H18 0.984371 4.115583 -0.281127
H19 1.697451 3.375345 1.174980
H20 2.391834 3.066184 -0.444347
H21 -1.267705 3.102320 -0.371786
N22 0.932279 -1.905687 0.085621
H23 0.923735 -2.912560 -0.016782
O24 -1.339649 -2.147776 -0.162962
H25 -2.871589 -1.666963 0.350937
H26 2.948567 0.567206 0.199199

Zero-point correction=	0.212499 (Hartree/Particle)
Thermal correction to Energy=	0.226385
Thermal correction to Enthalpy=	0.227329
Thermal correction to Gibbs Free Energy=	0.171571
Sum of electronic and zero-point Energies=	-677.035526
Sum of electronic and thermal Energies=	-677.021640
Sum of electronic and thermal Enthalpies=	-677.020695
Sum of electronic and thermal Free Energies=	-677.076453

8-CH_2NH_2[9MG + H_{N3}]$^{\bullet+}$

C1 -0.077052 0.867508 -0.381904
C2 -0.033692 -0.530331 -0.269411
C3 -2.337897 -0.712816 0.217817
C4 -1.332385 1.559253 -0.185506
C5 2.030382 0.268992 -0.722355
N6 -1.136191 -1.305201 0.028718
N7 1.202225 -0.957076 -0.505687
N8 -3.411890 -1.454540 0.508581
H9 -4.314910 -1.028516 0.651836
H10 -3.368561 -2.460111 0.565944
N11 1.114921 1.377771 -0.677904
N12 2.698210 0.520169 1.715409
H13 2.242230 -0.302567 2.085915
C14 3.141658 0.405999 0.354458
H15 3.714121 1.297464 0.084580
H16 3.820983 -0.448821 0.262617
C17 1.697640 -2.317276 -0.516990
H18 2.655857 -2.332074 -1.037582
H19 1.849675 -2.705397 0.496072
H20 1.017769 -2.973269 -1.070337
H21 2.504305 0.198962 -1.713121
N22 -2.410692 0.614569 0.110905
H23 -3.304092 1.076777 0.245736
O24 -1.605557 2.727211 -0.228177
H25 2.148435 1.350446 1.894566
H26 -1.049364 -2.308403 0.122389

Zero-point correction= 0.214052 (Hartree/Particle)
Thermal correction to Energy= 0.227971
Thermal correction to Enthalpy= 0.228915
Thermal correction to Gibbs Free Energy= 0.173273
Sum of electronic and zero-point Energies= -677.065615
Sum of electronic and thermal Energies= -677.051697
Sum of electronic and thermal Free Energies= -677.106395

TS for 8-CH_2NH_2[9MG + H_{N3}]$^{\bullet+}$

C1 -0.073430 0.866632 -0.518062
C2 0.013229 -0.507397 -0.413625
C3 -2.227906 -0.778717 0.235251
C4 -1.317796 1.529405 -0.230006
C5 1.949585 0.345572 -0.957760
N6 -1.032823 -1.334615 -0.038496
N7 1.263463 -0.870351 -0.767284
N8 -3.261676 -1.545770 0.608021
H9 -4.166266 -1.141525 0.794328
H10 -3.200498 -2.551563 0.620941
N11 1.116724 1.387427 -0.919993
N12 2.100933 0.350714 1.939049
H13 2.276561 -0.369516 2.624033
C14 3.046959 0.537224 0.943610
H15 3.357740 1.555222 0.734306
H16 3.812719 -0.230590 0.867941
C17 1.869291 -2.182268 -0.672453
H18 2.817544 -2.164988 -1.209782
H19 2.058800 -2.453024 0.371450
H20 1.233983 -2.933925 -1.148612
H21 2.869380 0.339827 -1.530335
N22 -2.346551 0.547522 0.133899
H23 -3.242008 0.982091 0.329805
O24 -1.631764 2.688462 -0.232786
H25 1.698037 1.184695 2.340498
H26 -0.901960 -2.331599 0.059631

Zero-point correction= 0.211383 (Hartree/Particle)
Thermal correction to Energy= 0.225605
Thermal correction to Enthalpy= 0.226549
Thermal correction to Gibbs Free Energy= 0.170168
Sum of electronic and zero-point Energies= -677.040616
Sum of electronic and thermal Energies= -677.026394
Sum of electronic and thermal Enthalpies= -677.025450
Sum of electronic and thermal Free Energies= -677.081831

Coordinates for structures in Table S2, optimized at ωB97XD/6-31+G(d,p)

[9MG + H_{N7}]$^{+}$···$^{•}$NHCH$_3$
C1 -0.130241 -0.035671 -0.002442
C2 1.026028 0.712257 -0.001418
C3 2.384634 -1.041750 0.001679
C4 -0.064312 -1.469341 -0.001919
C5 -0.718758 2.058986 -0.007958
N6 2.294156 0.274682 0.001393
O7 -0.973471 -2.276850 -0.003352
N8 0.630118 2.034730 -0.006110
N9 3.598629 -1.615276 0.003288
H10 3.740708 -2.611223 0.003447
H11 4.404834 -1.010459 0.004533
N12 -1.201263 0.829353 -0.006495
N13 -3.545487 -0.455232 -0.003053
H14 -2.228361 0.483532 -0.007326
C15 -4.964956 -0.281551 0.010315
H16 -5.403772 -0.773968 0.891239
H17 -5.231375 0.775999 0.015647
H18 -5.419545 -0.769173 -0.865273
C19 1.533886 3.181686 0.008806
H20 0.953344 4.091501 -0.138570
H21 2.054429 3.223519 0.966173
H22 2.260202 3.072794 -0.796536
H23 -1.308365 2.963132 -0.011120
N24 1.287606 -1.875194 0.000692
H25 1.413749 -2.880772 0.001319
H26 -3.292084 -1.449230 -0.007112

Zero-point correction=	0.210597 (Hartree/Particle)
Thermal correction to Energy=	0.225444
Thermal correction to Enthalpy=	0.226388
Thermal correction to Gibbs Free Energy=	0.167105
Sum of electronic and zero-point Energies=	-677.094550
Sum of electronic and thermal Energies=	-677.079702
Sum of electronic and thermal Enthalpies=	-677.078758
Sum of electronic and thermal Free Energies=	-677.138042

TS for [9MG + H_{N7}]$^{+}$···$^{•}$NHCH$_3$
C1 -0.160184 -0.013502 -0.001306
C2 1.009344 0.717015 -0.001408
C3 2.342726 -1.056942 0.000805
C4 -0.107400 -1.443526 -0.000657
C5 -0.728743 2.064918 -0.006942
N6 2.272555 0.258464 0.000428
O7 -1.030742 -2.245242 -0.001207
N8 0.628062 2.038724 -0.006358
N9 3.548210 -1.652378 0.001372
H10 3.673272 -2.650331 0.001812
H11 4.364027 -1.060975 0.002059
N12 -1.236330 0.849011 -0.004837
N13 -3.393173 -0.483320 -0.002012
H14 -2.398772 0.342389 -0.004769
C15 -4.800582 -0.269426 0.007137
H16 -5.247106 -0.755510 0.887268
H17 -5.036111 0.794436 0.009755
H18 -5.258114 -0.753694 -0.868332
C19 1.536151 3.179986 0.008771
H20 0.961556 4.090991 -0.155203
H21 2.046485 3.232861 0.971373
H22 2.272324 3.062738 -0.786649
H23 -1.298296 2.982555 -0.010229
N24 1.232385 -1.874718 0.000905
H25 1.342733 -2.881907 0.001590
H26 -3.085891 -1.463237 -0.003920

Zero-point correction=	0.207012 (Hartree/Particle)
Thermal correction to Energy=	0.221467
Thermal correction to Enthalpy=	0.222411
Thermal correction to Gibbs Free Energy=	0.164336
Sum of electronic and zero-point Energies=	-677.096076
Sum of electronic and thermal Energies=	-677.081621
Sum of electronic and thermal Enthalpies=	-677.080677
Sum of electronic and thermal Free Energies=	-677.138752

[9MG + H_{N7}]$^{+}$
C1 0.218019 0.965160 0.000572
C2 0.528992 -0.374276 -0.000745
C3 -1.583630 -1.053973 -0.000667
C4 -1.154521 1.397567 0.001770
C5 2.407182 0.787604 -0.004023
N6 -0.310838 -1.415562 -0.000753
O7 -1.589032 2.526576 0.002977
N8 1.912295 -0.458103 -0.004401
N9 -2.529293 -2.002807 -0.002349
H10 -3.515601 -1.802197 -0.003220
H11 -2.231590 -2.966117 -0.003704
N12 1.406773 1.662158 -0.001405
H13 1.499291 2.670510 -0.002893
C14 2.672270 -1.708065 0.006560
H15 3.730043 -1.480310 -0.117077
H16 2.506558 -2.220408 0.954515
H17 2.328509 -2.336189 -0.814947
H18 3.457508 1.036287 -0.006137
N19 -1.994493 0.259818 0.001302
H20 -2.983433 0.483188 0.002097

Zero-point correction=	0.160152 (Hartree/Particle)
Thermal correction to Energy=	0.170351
Thermal correction to Enthalpy=	0.171295
Thermal correction to Gibbs Free Energy=	0.124624
Sum of electronic and zero-point Energies=	-581.942158
Sum of electronic and thermal Energies=	-581.931959
Sum of electronic and thermal Enthalpies=	-581.931015
Sum of electronic and thermal Free Energies=	-581.977685

$^{•}$NHCH$_3$
N1 -0.801380 0.153228 0.000051
C2 0.627388 -0.012835 0.000061
H3 0.965291 -0.581461 -0.881652
H4 1.125389 0.959072 0.001636
H5 0.965569 -0.584890 0.879405
H6 -1.210917 -0.788305 -0.000113

Zero-point correction=	0.049364 (Hartree/Particle)
Thermal correction to Energy=	0.052833
Thermal correction to Enthalpy=	0.053777
Thermal correction to Gibbs Free Energy=	0.025915
Sum of electronic and zero-point Energies=	-95.117915
Sum of electronic and thermal Energies=	-95.114446
Sum of electronic and thermal Enthalpies=	-95.113502
Sum of electronic and thermal Free Energies=	-95.141364

2-NHCH$_3$[9MG + H_{N7}]$^{•+}$
C1 -1.030234 0.900654 0.135359
C2 -0.759103 -0.429631 -0.170534
C3 1.549337 -0.035691 -0.323146
C4 -0.004704 1.945947 0.019526
C5 -2.920615 -0.215158 0.251251
N6 0.396773 -0.967805 -0.450483
O7 -0.241867 3.128531 0.168413
N8 -1.992615 -1.101318 -0.086918
N9 2.483991 -0.211324 -1.394736
H10 2.066010 -0.034028 -2.302577
H11 2.890520 -1.139893 -1.393167
N12 -2.363740 0.999824 0.387770
N13 2.074909 -0.325983 0.988462

H14 2.641416 0.454682 1.301782
C15 2.775041 -1.598433 1.166982
H16 2.136585 -2.417914 0.831450
H17 3.737121 -1.649641 0.640208
H18 2.963590 -1.737488 2.232404
C19 -2.176177 -2.532749 -0.326292
H20 -3.217249 -2.792129 -0.139534
H21 -1.913709 -2.755408 -1.360700
H22 -1.523253 -3.088727 0.346479
H23 -3.968152 -0.432831 0.399904
N24 1.176381 1.388194 -0.370879
H25 1.940482 2.026436 -0.560775
H26 -2.849590 1.857945 0.625832

Zero-point correction=	0.215834 (Hartree/Particle)
Thermal correction to Energy=	0.228811
Thermal correction to Enthalpy=	0.229755
Thermal correction to Gibbs Free Energy=	0.176276
Sum of electronic and zero-point Energies=	-677.060975
Sum of electronic and thermal Energies=	-677.047998
Sum of electronic and thermal Enthalpies=	-677.047054
Sum of electronic and thermal Free Energies=	-677.100533

TS for 2-NHCH$_3$[9MG + H$_{N7}$]$^{•+}$

C1 -0.932081 0.949167 0.192440
C2 -0.738255 -0.360546 -0.217161
C3 1.460736 -0.093414 -0.533858
C4 0.102852 1.930730 0.016863
C5 -2.883736 -0.063234 0.286936
N6 0.370656 -0.934100 -0.652825
O7 0.030722 3.129211 0.209803
N8 -1.986813 -0.972350 -0.134782
N9 2.570930 -0.461507 -1.321174
H10 2.970546 0.253095 -1.914250
H11 2.428421 -1.343116 -1.797496
N12 -2.275130 1.096370 0.482738
N13 2.190266 -0.455439 0.956984
H14 2.945243 0.169872 1.248342
C15 2.169622 -1.752141 1.558567
H16 1.362984 -2.332813 1.108570
H17 3.132196 -2.260270 1.421607
H18 2.007302 -1.639997 2.638155
C19 -2.248731 -2.360910 -0.493608
H20 -3.302497 -2.579968 -0.325308
H21 -1.999748 -2.514142 -1.544271
H22 -1.630981 -3.014324 0.123312
H23 -3.932497 -0.257295 0.449370
N24 1.254391 1.311895 -0.489484
H25 2.036868 1.939629 -0.627267
H26 -2.716155 1.953644 0.789545

Zero-point correction=	0.211735 (Hartree/Particle)
Thermal correction to Energy=	0.225307
Thermal correction to Enthalpy=	0.226251
Thermal correction to Gibbs Free Energy=	0.171311
Sum of electronic and zero-point Energies=	-677.037678
Sum of electronic and thermal Energies=	-677.024107
Sum of electronic and thermal Enthalpies=	-677.023163
Sum of electronic and thermal Free Energies=	-677.078102

3-NHCH$_3$[9MG + H$_{N7}$]$^{•+}$

C1 1.328205 0.337176 0.395784
C2 0.225294 -0.560330 0.259538
C3 -1.124076 1.339808 -0.334600
C4 1.285326 1.715022 0.178053
C5 2.131364 -1.664720 -0.138006
N6 -0.989373 -0.015016 -0.262095
N7 0.813023 -1.756485 -0.262983
N8 -2.322967 1.839940 -0.617817
H9 -2.491368 2.832630 -0.644274
H10 -3.084214 1.181794 -0.725404
N11 2.478377 -0.452022 0.275384
N12 -2.215001 -0.700551 -0.126362
H13 -2.224145 -1.476652 -0.776638
C14 -2.516293 -1.104611 1.250234
H15 -2.664605 -0.210365 1.859672
H16 -3.451163 -1.666799 1.227549
H17 -1.727213 -1.712426 1.707262
C18 0.104494 -2.977157 -0.619974
H19 0.823815 -3.784330 -0.757621
H20 -0.586667 -3.250540 0.180422
H21 -0.434634 -2.833334 -1.558865
H22 2.819861 -2.483403 -0.291151
N23 -0.084460 2.144936 -0.095486
H24 -0.178270 3.143485 -0.235219
O25 2.176597 2.553233 0.166295
H26 3.422755 -0.102671 0.363236

Zero-point correction=	0.215920 (Hartree/Particle)
Thermal correction to Energy=	0.229054
Thermal correction to Enthalpy=	0.229998
Thermal correction to Gibbs Free Energy=	0.176513
Sum of electronic and zero-point Energies=	-677.016142
Sum of electronic and thermal Energies=	-677.003008
Sum of electronic and thermal Enthalpies=	-677.002064
Sum of electronic and thermal Free Energies=	-677.055549

TS for 3-NHCH$_3$-[9MG + H$_{N7}$]$^{•+}$

C1 0.256699 1.247274 0.058621
C2 0.548127 -0.047779 -0.295060
C3 -1.610208 -0.575576 -0.620374
C4 -1.096458 1.722250 0.161476
C5 2.440055 1.079254 -0.183306
N6 -0.340585 -1.081605 -0.447248
N7 1.925633 -0.126754 -0.443584
N8 -2.544692 -1.405986 -1.134543
H9 -3.523221 -1.167372 -1.087247
H10 -2.331229 -2.392115 -1.081826
N11 1.454529 1.924347 0.114392
N12 -0.374307 -2.185054 0.806563
H13 0.324401 -2.858984 0.497850
C14 0.012445 -1.576374 2.069760
H15 -0.737768 -0.838532 2.365912
H16 0.017796 -2.363555 2.829845
H17 1.004592 -1.097609 2.079007
C18 2.668728 -1.334237 -0.796551
H19 3.673175 -1.056465 -1.113231
H20 2.725208 -1.997169 0.069073
H21 2.149979 -1.832062 -1.615899
H22 3.490822 1.324130 -0.211696
N23 -1.964938 0.676172 -0.184263
H24 -2.940002 0.954420 -0.220549
O25 -1.477175 2.840222 0.475469
H26 1.557843 2.906819 0.338396

Zero-point correction=	0.212112 (Hartree/Particle)
Thermal correction to Energy=	0.225663
Thermal correction to Enthalpy=	0.226607
Thermal correction to Gibbs Free Energy=	0.171811
Sum of electronic and zero-point Energies=	-677.010978
Sum of electronic and thermal Energies=	-676.997427
Sum of electronic and thermal Enthalpies=	-676.996483
Sum of electronic and thermal Free Energies=	-677.051279

4-NHCH$_3$[9MG + H$_{N7}$]$^{•+}$

C1 0.301655 -1.130219 0.312018
C2 -0.534170 0.113776 0.197541
C3 1.359061 1.174976 -0.567929
C4 1.745029 -1.040430 0.369647
C5 -1.595213 -1.800591 -0.626889

N6 0.067120 1.085696 -0.654378
O7 2.495046 -1.970961 0.593843
N8 -1.737633 -0.511082 -0.546832
N9 2.009397 2.213977 -1.133447
H10 2.987449 2.161523 -1.368894
H11 1.447668 2.887487 -1.631042
N12 -0.413596 -2.215749 -0.118996
N13 -0.863210 0.640706 1.471221
H14 -1.204435 -0.052071 2.125109
C15 -1.469192 1.960843 1.616810
H16 -0.970293 2.659638 0.945775
H17 -2.546389 1.981042 1.409894
H18 -1.308575 2.298214 2.641825
C19 -2.868344 0.276096 -1.014722
H20 -3.450084 -0.303715 -1.731816
H21 -3.501646 0.561212 -0.172475
H22 -2.471031 1.169591 -1.498324
H23 -2.322871 -2.482821 -1.048737
N24 2.171512 0.257632 0.101774
H25 3.165822 0.423392 0.192857
H26 -0.054059 -3.160774 -0.169166

Zero-point correction= 0.214786 (Hartree/Particle)
Thermal correction to Energy= 0.228367
Thermal correction to Enthalpy= 0.229311
Thermal correction to Gibbs Free Energy= 0.174426
Sum of electronic and zero-point Energies= -677.068578
Sum of electronic and thermal Energies= -677.054997
Sum of electronic and thermal Enthalpies= -677.054053
Sum of electronic and thermal Free Energies= -677.108937

TS for 4-$NHCH_3$[9MG + H_{N7}]$^{\bullet+}$

C1 -0.019101 0.963396 -0.270270
C2 0.318584 -0.402142 -0.150668
C3 -1.840845 -1.014780 -0.049069
C4 -1.372046 1.414546 -0.108808
C5 2.013398 0.715720 -1.078818
N6 -0.606100 -1.407782 -0.224994
O7 -1.780637 2.556698 -0.161975
N8 1.583668 -0.505646 -0.786959
N9 -2.828367 -1.928790 0.010822
H10 -3.799100 -1.682916 -0.093400
H11 -2.568390 -2.894292 -0.120191
N12 1.078947 1.621080 -0.781894
N13 0.892144 -0.511807 1.668079
H14 0.029262 -0.294927 2.175584
C15 1.947481 0.370162 2.120129
H16 2.879873 0.142917 1.595632
H17 1.719825 1.440846 2.022265
H18 2.118509 0.178396 3.186947
C19 2.311864 -1.762095 -0.890768
H20 3.275507 -1.584294 -1.366648
H21 2.445453 -2.164231 0.116374
H22 1.720664 -2.460416 -1.483394
H23 2.983233 0.944683 -1.496150
N24 -2.215909 0.310810 0.117166
H25 -3.189296 0.549794 0.263765
H26 1.142870 2.616951 -0.950900

Zero-point correction= 0.212670 (Hartree/Particle)
Thermal correction to Energy= 0.225932
Thermal correction to Enthalpy= 0.226877
Thermal correction to Gibbs Free Energy= 0.173148
Sum of electronic and zero-point Energies= -677.043269
Sum of electronic and thermal Energies= -677.030007
Sum of electronic and thermal Enthalpies= -677.029063
Sum of electronic and thermal Free Energies= -677.082791

5-$NHCH_3$[9MG + H_{N7}]$^{\bullet+}$

C1 -0.049512 -0.785497 0.079369
C2 -0.690184 0.562011 0.245417
C3 1.238973 1.670991 0.098708
C4 1.155558 -0.593088 -0.847514
C5 -2.281787 -0.766783 -0.524758
N6 -0.036396 1.704458 0.427660
O7 1.532808 -1.391939 -1.671098
N8 -2.036487 0.451087 -0.038564
N9 2.033244 2.720746 0.362381
H10 3.034597 2.677302 0.266716
H11 1.633401 3.499355 0.863216
N12 -1.166710 -1.500635 -0.564563
N13 0.428381 -1.316867 1.324923
H14 -0.255821 -1.182133 2.061683
C15 0.969487 -2.676704 1.313063
H16 1.803813 -2.742028 0.611773
H17 1.351367 -2.899836 2.309737
H18 0.227483 -3.441804 1.046981
H19 -3.258805 -1.102766 -0.840963
C20 -2.986156 1.557644 0.055498
H21 -2.932982 1.986329 1.056028
H22 -2.725857 2.319224 -0.680641
H23 -3.989491 1.178419 -0.133664
N24 1.799530 0.614323 -0.604458
H25 2.666316 0.750870 -1.110591
H26 -1.105687 -2.430641 -0.951847

Zero-point correction= 0.214314 (Hartree/Particle)
Thermal correction to Energy= 0.228033
Thermal correction to Enthalpy= 0.228977
Thermal correction to Gibbs Free Energy= 0.174247
Sum of electronic and zero-point Energies= -677.081102
Sum of electronic and thermal Energies= -677.067382
Sum of electronic and thermal Enthalpies= -677.066438
Sum of electronic and thermal Free Energies= -677.121169

TS for 5-$NHCH_3$[9MG + H_{N7}]$^{\bullet+}$

C1 0.251497 0.588823 -0.302565
C2 0.488003 -0.770441 -0.010122
C3 -1.672178 -1.273319 0.117454
C4 -1.076742 0.922358 -0.832393
C5 2.411823 0.137693 -0.594779
N6 -0.429403 -1.675722 0.332450
O7 -1.377611 1.916972 -1.448027
N8 1.844081 -0.982662 -0.123306
N9 -2.686334 -2.074832 0.466135
H10 -3.649968 -1.785694 0.429390
H11 -2.462712 -2.950097 0.914320
N12 1.466415 1.066232 -0.784493
N13 -0.162419 1.399954 1.476740
H14 0.489553 0.870321 2.064010
C15 0.268526 2.786082 1.442402
H16 -0.304813 3.334693 0.689696
H17 0.037557 3.233040 2.418083
H18 1.344872 2.935922 1.271748
H19 3.464840 0.263748 -0.792050
C20 2.513059 -2.252187 0.157808
H21 2.314127 -2.538064 1.190575
H22 2.124405 -3.016707 -0.515399
H23 3.583881 -2.127725 0.004004
N24 -1.989231 -0.089524 -0.515586
H25 -2.947952 0.108660 -0.775192
H26 1.621402 1.997962 -1.145376
Zero-point correction= 0.212555 (Hartree/Particle)
Thermal correction to Energy= 0.226027
Thermal correction to Enthalpy= 0.226971
Thermal correction to Gibbs Free Energy= 0.172616
Sum of electronic and zero-point Energies= -677.047448
Sum of electronic and thermal Energies= -677.033976
Sum of electronic and thermal Enthalpies= -677.033032
Sum of electronic and thermal Free Energies= -677.087388

8-NHCH$_3$[9MG + H$_{N7}$]$^{•+}$
C1 0.167710 -0.781735 -0.226485
C2 0.272830 0.634800 -0.142715
C3 2.489349 0.560526 0.192220
C4 1.336778 -1.604579 -0.095398
C5 -1.949201 0.096984 -0.451161
N6 2.485065 -0.804339 0.126483
N7 1.394416 1.311917 0.062301
O8 1.398111 -2.814546 -0.156128
N9 -0.944475 1.153315 -0.334003
N10 3.651456 1.181141 0.399233
H11 4.527383 0.696625 0.512786
H12 3.644637 2.188968 0.450435
N13 -1.108881 -1.103741 -0.471089
N14 -2.834430 0.094255 0.682892
H15 -3.207855 1.027847 0.821956
C16 -3.928895 -0.873082 0.571099
H17 -3.544187 -1.888858 0.697018
H18 -4.632156 -0.696999 1.384986
H19 -4.472804 -0.810732 -0.383027
H20 -2.469358 0.185448 -1.422042
C21 -1.280304 2.565893 -0.316451
H22 -0.375738 3.135704 -0.528114
H23 -2.024445 2.770437 -1.089167
H24 -1.665062 2.865250 0.662914
H25 -1.462055 -2.047818 -0.538316
H26 3.345112 -1.330189 0.232221

Zero-point correction=	0.215662 (Hartree/Particle)
Thermal correction to Energy=	0.229180
Thermal correction to Enthalpy=	0.230124
Thermal correction to Gibbs Free Energy=	0.175125
Sum of electronic and zero-point Energies=	-677.104119
Sum of electronic and thermal Energies=	-677.090601
Sum of electronic and thermal Enthalpies=	-677.089656
Sum of electronic and thermal Free Energies=	-677.144655

TS for 8-NHCH$_3$[9MG + H$_{N7}$]$^{•+}$
C1 -0.154679 0.831897 -0.483567
C2 -0.037369 -0.544532 -0.410271
C3 -2.155449 -0.852098 0.199627
C4 -1.394780 1.481377 -0.176807
C5 1.959604 0.309309 -0.903947
N6 -2.364892 0.504863 0.153072
N7 -0.990160 -1.419478 -0.071020
O8 -1.645652 2.667545 -0.177815
N9 1.260926 -0.855166 -0.738517
N10 -3.189836 -1.630748 0.546682
H11 -4.111352 -1.273037 0.736124
H12 -3.045887 -2.628534 0.560037
N13 1.071592 1.330573 -0.856042
N14 2.977989 0.414112 0.864495
H15 3.384762 1.355645 0.882525
C16 2.079761 0.278260 1.982867
H17 1.634376 -0.719520 1.997555
H18 2.644532 0.417849 2.914593
H19 1.277845 1.034269 1.989209
H20 2.880180 0.359584 -1.465472
C21 1.879791 -2.171953 -0.678271
H22 1.106969 -2.895117 -0.422355
H23 2.313933 -2.424164 -1.646616
H24 2.659642 -2.159725 0.087920
H25 1.275030 2.299983 -1.056540
H26 -3.275427 0.889748 0.377059

Zero-point correction=	0.212087 (Hartree/Particle)
Thermal correction to Energy=	0.225699
Thermal correction to Enthalpy=	0.226643
Thermal correction to Gibbs Free Energy=	0.171762
Sum of electronic and zero-point Energies=	-677.063576
Sum of electronic and thermal Energies=	-677.049964
Sum of electronic and thermal Enthalpies=	-677.049020
Sum of electronic and thermal Free Energies=	-677.103901

[9MG + H$_{O6}$]$^{+}$···$^{•}$NHCH$_3$
C1 0.663097 -0.888543 0.090739
C2 1.616547 0.139690 -0.019752
C3 0.132030 1.794484 0.047584
C4 -0.665915 -0.504780 0.176925
C5 2.533692 -1.846172 -0.023982
N6 1.396469 1.463955 -0.038382
N7 2.812629 -0.487647 -0.089003
N8 -0.235700 3.091902 0.005034
H9 -1.147291 3.409743 0.289603
H10 0.509891 3.771437 -0.008963
N11 1.268615 -2.125614 0.085451
N12 -4.091387 -0.336612 0.383623
H13 -4.592725 -0.141173 1.254062
C14 -5.018629 -0.567482 -0.682198
H15 -4.500369 -0.677759 -1.635161
H16 -5.577060 -1.496356 -0.476903
H17 -5.772600 0.229080 -0.743765
C18 4.117245 0.141443 -0.245012
H19 4.880985 -0.538294 0.132253
H20 4.308167 0.362990 -1.296739
H21 4.138309 1.065967 0.332043
H22 3.330854 -2.576693 -0.060030
N23 -0.882570 0.856483 0.148248
H24 -1.844948 1.172455 0.164139
O25 -1.661336 -1.318118 0.273449
H26 -2.587325 -0.885553 0.331250

Zero-point correction=	0.210681 (Hartree/Particle)
Thermal correction to Energy=	0.225519
Thermal correction to Enthalpy=	0.226463
Thermal correction to Gibbs Free Energy=	0.166733
Sum of electronic and zero-point Energies=	-677.073222
Sum of electronic and thermal Energies=	-677.058384
Sum of electronic and thermal Enthalpies=	-677.057439
Sum of electronic and thermal Free Energies=	-677.117170

TS for [9MG + H$_{O6}$]$^{+}$···$^{•}$NHCH$_3$
C1 -0.432329 -0.772292 0.029565
C2 -1.629307 -0.043588 -0.001775
C3 -0.652360 1.950973 0.005201
C4 0.762889 -0.050535 0.060307
C5 -1.979302 -2.202064 0.001873
N6 -1.782036 1.293478 -0.026470
N7 -2.615983 -0.971124 -0.018511
N8 -0.651384 3.304365 0.042189
H9 0.151877 3.844046 -0.235837
H10 -1.552449 3.746685 -0.061348
N11 -0.680224 -2.126631 0.031098
N12 4.266496 0.100246 -0.087002
H13 4.779976 0.970636 -0.230469
C14 5.101119 -1.056525 -0.036676
H15 4.505821 -1.944733 0.171502
H16 5.623387 -1.174673 -0.999373
H17 5.886842 -0.929841 0.721752
C18 -4.047049 -0.709773 -0.070487
H19 -4.575052 -1.656652 0.038225
H20 -4.329982 -0.041214 0.743530
H21 -4.310389 -0.252099 -1.025530
H22 -2.549927 -3.121178 -0.005201
N23 0.582588 1.324763 0.048532
H24 1.409267 1.897465 0.151570
O25 1.925250 -0.555464 0.107755
H26 2.980459 -0.067594 0.012232

Zero-point correction=	0.206454 (Hartree/Particle)

Thermal correction to Energy= 0.220955
Thermal correction to Enthalpy= 0.221899
Thermal correction to Gibbs Free Energy= 0.162372
Sum of electronic and zero-point Energies= -677.075224
Sum of electronic and thermal Energies= -677.060723
Sum of electronic and thermal Enthalpies= -677.059779
Sum of electronic and thermal Free Energies= -677.119306

[9MG + H$_{O6}$]$^+$
C1 0.217423 0.984707 0.000630
C2 0.604994 -0.374920 -0.001345
C3 -1.476691 -1.167360 -0.001030
C4 -1.134307 1.237435 0.001512
C5 2.319953 0.984556 -0.003910
N6 -0.194679 -1.446730 -0.001880
N7 1.954328 -0.357982 -0.006351
N8 -2.384224 -2.156081 -0.001080
H9 -3.380099 -2.011699 -0.007003
H10 -2.038203 -3.103798 -0.002712
N11 1.319129 1.812406 -0.001102
C12 2.847541 -1.510956 0.008387
H13 3.619440 -1.381477 -0.750885
H14 3.305930 -1.618793 0.992853
H15 2.262208 -2.401151 -0.219612
H16 3.363487 1.271204 -0.007014
N17 -1.956100 0.136819 0.001512
H18 -2.958596 0.278003 0.005706
O19 -1.628225 2.451646 0.003012
H20 -2.591020 2.514742 0.001411

Zero-point correction= 0.158451 (Hartree/Particle)
Thermal correction to Energy= 0.169207
Thermal correction to Enthalpy= 0.170151
Thermal correction to Gibbs Free Energy= 0.122313
Sum of electronic and zero-point Energies= -581.916314
Sum of electronic and thermal Energies= -581.905558
Sum of electronic and thermal Enthalpies= -581.904614
Sum of electronic and thermal Free Energies= -581.952451

2-NHCH$_3$[9MG + H$_{O6}$]$^{•+}$
C1 -1.045539 0.890640 0.181154
C2 -0.748513 -0.458343 -0.160989
C3 1.541624 -0.091627 -0.333616
C4 -0.003582 1.846095 0.044064
C5 -2.881577 -0.130042 0.255545
N6 0.391770 -0.981517 -0.474793
O7 -0.163238 3.134555 0.166444
N8 -1.987667 -1.082491 -0.103526
N9 2.452242 -0.177101 -1.440238
H10 1.975926 -0.142842 -2.336990
H11 2.988232 -1.037156 -1.398258
N12 -2.351421 1.080873 0.426939
N13 2.140616 -0.369574 0.945907
H14 2.943206 0.241112 1.068836
C15 2.514013 -1.760820 1.222168
H16 1.623044 -2.387688 1.241336
H17 3.228656 -2.186318 0.504187
H18 2.971441 -1.787021 2.211650
C19 -2.236684 -2.494139 -0.370321
H20 -3.287801 -2.705821 -0.177936
H21 -2.000996 -2.716856 -1.411823
H22 -1.612531 -3.103298 0.284497
H23 -3.932101 -0.352723 0.387921
N24 1.172201 1.375215 -0.298268
H25 1.931695 2.018568 -0.500399
H26 -1.085508 3.355178 0.375246

Zero-point correction= 0.215654 (Hartree/Particle)
Thermal correction to Energy= 0.228745
Thermal correction to Enthalpy= 0.229690
Thermal correction to Gibbs Free Energy= 0.175696
Sum of electronic and zero-point Energies= -677.061364
Sum of electronic and thermal Energies= -677.048272
Sum of electronic and thermal Enthalpies= -677.047328
Sum of electronic and thermal Free Energies= -677.101321

TS for 2-NHCH$_3$[9MG + H$_{O6}$]$^{•+}$
C1 -1.109958 0.866653 0.116322
C2 -0.761031 -0.434259 -0.283915
C3 1.388333 0.088932 -0.686797
C4 -0.133623 1.839353 0.008125
C5 -2.854503 -0.282248 0.344384
N6 0.430166 -0.855707 -0.715547
O7 -0.254245 3.117656 0.268794
N8 -1.894597 -1.161183 -0.129329
N9 2.527129 -0.115059 -1.404960
H10 3.362406 0.389705 -1.144242
H11 2.688808 -1.079121 -1.661207
N12 -2.425953 0.939622 0.504327
N13 1.748116 -0.672405 1.241584
H14 1.450841 0.048015 1.884839
C15 2.892186 -1.509267 1.288519
H16 2.680700 -2.482932 0.832642
H17 3.794605 -1.084190 0.820730
H18 3.112420 -1.712142 2.345649
C19 -2.051071 -2.583298 -0.409987
H20 -2.965932 -2.934965 0.065639
H21 -2.108717 -2.750100 -1.486800
H22 -1.197262 -3.122231 0.001315
H23 -3.861193 -0.619236 0.552419
N24 1.076644 1.438190 -0.436803
H25 1.784489 2.148447 -0.582049
H26 -1.159743 3.328100 0.545908

Zero-point correction= 0.219042 (Hartree/Particle)
Thermal correction to Energy= 0.232522
Thermal correction to Enthalpy= 0.233466
Thermal correction to Gibbs Free Energy= 0.178231
Sum of electronic and zero-point Energies= -676.990527
Sum of electronic and thermal Energies= -676.977048
Sum of electronic and thermal Enthalpies= -676.976104
Sum of electronic and thermal Free Energies= -677.031339

3-NHCH$_3$[9MG + H$_{O6}$]$^{•+}$
C1 -0.488479 -1.325770 -0.082269
C2 0.478804 -0.369311 0.128354
C3 -1.132983 1.341139 0.365880
C4 -1.852165 -0.974314 0.027099
C5 1.366237 -2.350285 -0.007333
N6 0.193234 0.995355 0.363689
N7 1.676453 -1.031063 0.220742
N8 -1.439226 2.628207 0.514242
H9 -2.383948 2.967903 0.432828
H10 -0.666359 3.280963 0.550563
N11 0.086567 -2.565872 -0.185221
N12 1.074262 2.036891 0.024721
H13 1.859055 2.018686 0.664934
C14 1.468971 2.043553 -1.387542
H15 0.597775 2.283023 -2.001566
H16 2.208937 2.834825 -1.516707
H17 1.891339 1.091615 -1.731083
C18 2.993861 -0.513480 0.555361
H19 3.692104 -1.349603 0.582245
H20 3.341902 0.197570 -0.197519
H21 2.980878 -0.046489 1.543295
H22 2.138668 -3.106261 -0.034909
N23 -2.077371 0.408844 0.235638
H24 -3.046400 0.679170 0.356156
O25 -2.929272 -1.590300 -0.494210
H26 -2.782679 -2.544729 -0.508532

Zero-point correction=	0.214681 (Hartree/Particle)
Thermal correction to Energy=	0.228382
Thermal correction to Enthalpy=	0.229326
Thermal correction to Gibbs Free Energy=	0.174692
Sum of electronic and zero-point Energies=	-677.023744
Sum of electronic and thermal Energies=	-677.010044
Sum of electronic and thermal Enthalpies=	-677.009100
Sum of electronic and thermal Free Energies=	-677.063734

TS for 3-NHCH$_3$[9MG + H$_{O6}$]$^{•+}$
C1 0.093079 1.314497 0.103230
C2 0.579409 0.070953 -0.301120
C3 -1.490803 -0.815161 -0.574323
C4 -1.282319 1.484468 0.138173
C5 2.184497 1.536858 -0.060402
N6 -0.148062 -1.064222 -0.535916
N7 1.930295 0.228222 -0.410933
N8 -2.302610 -1.819196 -0.938281
H9 -3.299125 -1.771214 -0.791713
H10 -1.884987 -2.740523 -0.903036
N11 1.114734 2.222124 0.246063
N12 0.060738 -2.361486 0.604731
H13 0.886052 -2.800013 0.196835
C14 0.377274 -1.807330 1.910472
H15 -0.497613 -1.288767 2.312456
H16 0.596749 -2.648197 2.577001
H17 1.239586 -1.125860 1.934239
C18 2.897924 -0.784628 -0.805027
H19 3.834231 -0.292112 -1.065556
H20 3.080179 -1.483555 0.015765
H21 2.524622 -1.322692 -1.677552
H22 3.193243 1.927656 -0.057723
N23 -2.019792 0.386119 -0.207381
H24 -3.024398 0.520997 -0.261106
O25 -1.982360 2.571486 0.417053
H26 -1.391154 3.313528 0.609962

Zero-point correction=	0.212360 (Hartree/Particle)
Thermal correction to Energy=	0.225800
Thermal correction to Enthalpy=	0.226744
Thermal correction to Gibbs Free Energy=	0.172336
Sum of electronic and zero-point Energies=	-677.011739
Sum of electronic and thermal Energies=	-676.998298
Sum of electronic and thermal Enthalpies=	-676.997354
Sum of electronic and thermal Free Energies=	-677.051762

4-NHCH$_3$[9MG + H$_{O6}$]$^{•+}$
C1 0.297160 -1.166494 0.151789
C2 -0.560960 0.079591 0.134757
C3 1.267047 1.248048 -0.576479
C4 1.667007 -0.961015 0.299948
C5 -1.564968 -1.843315 -0.592570
N6 -0.002812 1.107098 -0.700700
N7 -1.762461 -0.542089 -0.519527
N8 1.933720 2.330008 -1.049966
H9 2.872254 2.228685 -1.406911
H10 1.361328 3.019696 -1.515496
N11 -0.343676 -2.278384 -0.212540
N12 -0.783989 0.566721 1.464244
H13 -1.064938 -0.176712 2.094233
C14 -1.553429 1.794823 1.662379
H15 -1.209159 2.553415 0.958826
H16 -2.635980 1.668250 1.542241
H17 -1.361205 2.157150 2.673518
C18 -2.941070 0.197701 -0.937682
H19 -3.499944 -0.395256 -1.662228
H20 -3.581521 0.421590 -0.081924
H21 -2.611805 1.127508 -1.404584
H22 -2.324368 -2.521587 -0.965183
N23 2.102504 0.312183 0.088490
H24 2.991371 0.628648 0.455040
O25 2.496598 -1.942734 0.571742
H26 3.423465 -1.734308 0.395676

Zero-point correction=	0.215015 (Hartree/Particle)
Thermal correction to Energy=	0.228324
Thermal correction to Enthalpy=	0.229269
Thermal correction to Gibbs Free Energy=	0.175675
Sum of electronic and zero-point Energies=	-677.048443
Sum of electronic and thermal Energies=	-677.035134
Sum of electronic and thermal Enthalpies=	-677.034190
Sum of electronic and thermal Free Energies=	-677.087783

TS for 4-NHCH$_3$[9MG + H$_{O6}$]$^{•+}$
C1 -0.269856 1.186466 -0.010095
C2 0.459196 -0.048962 -0.203994
C3 -1.387605 -1.263100 -0.486399
C4 -1.620045 1.057634 0.194440
C5 1.702252 1.768246 -0.476889
N6 -0.115091 -1.182480 -0.725128
N7 1.711779 0.402215 -0.609549
N8 -2.086776 -2.395143 -0.736547
H9 -3.057446 -2.353775 -1.007373
H10 -1.552427 -3.157096 -1.127985
N11 0.549092 2.276385 -0.120905
N12 0.748654 -0.571865 1.607135
H13 1.042585 0.277476 2.100364
C14 1.764315 -1.589340 1.753324
H15 1.596245 -2.386421 1.024962
H16 2.797491 -1.225786 1.687476
H17 1.638124 -2.021526 2.756144
C18 2.792346 -0.419578 -1.132526
H19 3.248696 0.087702 -1.983301
H20 3.550963 -0.603789 -0.368145
H21 2.368081 -1.366688 -1.465996
H22 2.594377 2.351163 -0.667478
N23 -2.126199 -0.212042 0.066867
H24 -3.053411 -0.414593 0.418741
O25 -2.405387 2.067135 0.510114
H26 -3.344002 1.908569 0.351410

Zero-point correction=	0.211148 (Hartree/Particle)
Thermal correction to Energy=	0.224748
Thermal correction to Enthalpy=	0.225692
Thermal correction to Gibbs Free Energy=	0.170952
Sum of electronic and zero-point Energies=	-677.018404
Sum of electronic and thermal Energies=	-677.004804
Sum of electronic and thermal Enthalpies=	-677.003860
Sum of electronic and thermal Free Energies=	-677.058600

5-NHCH$_3$[9MG + H$_{O6}$]$^{•+}$
C1 -0.112349 -0.803276 0.133972
C2 -0.705374 0.594568 0.232208
C3 1.277624 1.585078 0.094974
C4 0.951457 -0.571862 -0.890627
C5 -2.256103 -0.879204 -0.301964
N6 -0.020598 1.697978 0.387825
N7 -2.028322 0.471147 0.039946
N8 2.133195 2.545121 0.466978
H9 3.130156 2.472115 0.346947
H10 1.771307 3.307140 1.020375
N11 -1.228971 -1.634662 -0.318038
N12 0.488770 -1.196621 1.389020
H13 -0.203584 -1.130840 2.130029
C14 1.101935 -2.529641 1.371102
H15 1.919001 -2.543350 0.644168
H16 1.530935 -2.719240 2.355769
H17 0.396479 -3.327945 1.117645
H18 -3.266581 -1.197405 -0.535813
C19 -2.986090 1.564097 -0.059755

H20 -2.774845 2.294600 0.721239
H21 -2.916220 2.047239 -1.037427
H22 -3.989631 1.163496 0.083493
N23 1.728039 0.547917 -0.679320
H24 2.663446 0.582550 -1.066886
O25 1.141182 -1.354271 -1.943016
H26 1.478679 -0.888895 -2.719744

Zero-point correction= 0.213351 (Hartree/Particle)
Thermal correction to Energy= 0.227364
Thermal correction to Enthalpy= 0.228308
Thermal correction to Gibbs Free Energy= 0.172905
Sum of electronic and zero-point Energies= -677.042358
Sum of electronic and thermal Energies= -677.028345
Sum of electronic and thermal Enthalpies= -677.027401
Sum of electronic and thermal Free Energies= -677.082804

TS for 5-NHCH$_3$[9MG + H$_{O6}$]$^{•+}$

C1 -0.130711 -0.680750 -0.210948
C2 -0.731175 0.610191 0.027260
C3 1.222868 1.659865 0.109608
C4 1.124482 -0.536214 -0.840223
C5 -2.226764 -0.896608 -0.531022
N6 -0.086933 1.728125 0.302009
N7 -2.066114 0.414636 -0.099398
N8 2.013775 2.684845 0.462936
H9 3.016237 2.602297 0.514939
H10 1.578770 3.454617 0.949091
N11 -1.123993 -1.565899 -0.663989
N12 0.379653 -1.187782 1.576453
H13 -0.553211 -1.202568 2.001168
C14 0.856807 -2.563913 1.531522
H15 1.818502 -2.608924 1.011196
H16 1.036069 -2.888625 2.563715
H17 0.156987 -3.274183 1.073424
H18 -3.214826 -1.285978 -0.742664
C19 -3.100377 1.429083 0.065865
H20 -2.995299 1.898253 1.044733
H21 -3.009368 2.187332 -0.713971
H22 -4.075016 0.947267 -0.003408
N23 1.807061 0.599914 -0.557881
H24 2.743909 0.691269 -0.935228
O25 1.723751 -1.399807 -1.629677
H26 1.152306 -2.169109 -1.778866

Zero-point correction= 0.211988 (Hartree/Particle)
Thermal correction to Energy= 0.225591
Thermal correction to Enthalpy= 0.226535
Thermal correction to Gibbs Free Energy= 0.171556
Sum of electronic and zero-point Energies= -677.026920
Sum of electronic and thermal Energies= -677.013318
Sum of electronic and thermal Enthalpies= -677.012374
Sum of electronic and thermal Free Energies= -677.067352

7-NHCH$_3$[9MG + H$_{O6}$]$^{•+}$

C1 -0.265514 -0.304432 0.293575
C2 0.471369 0.879680 0.095798
C3 2.405247 -0.236166 -0.138652
C4 0.379034 -1.497300 0.220176
C5 -1.706948 1.417216 0.237868
N6 1.748431 -1.433668 0.007390
N7 1.795438 0.936947 -0.109897
O8 -0.135766 -2.715358 0.321992
N9 -0.405020 1.897863 0.108384
N10 3.732514 -0.274816 -0.331187
H11 4.270789 -1.124945 -0.329829
H12 4.215690 0.604340 -0.430959
N13 -1.589308 0.041722 0.487302
N14 -2.565089 -0.945106 0.269502
H15 -3.257789 -0.868089 1.008014
C16 -3.187449 -0.875429 -1.061203
H17 -2.422442 -1.049166 -1.821287
H18 -3.926620 -1.675950 -1.121231
H19 -3.673728 0.086548 -1.254711
H20 -2.487335 2.023733 0.679441
C21 -0.069947 3.307659 -0.049500
H22 1.009531 3.390832 -0.167114
H23 -0.567946 3.705449 -0.935013
H24 -0.386132 3.860993 0.836721
H25 2.242633 -2.314091 -0.059103
H26 -1.104046 -2.624757 0.410314

Zero-point correction= 0.214596 (Hartree/Particle)
Thermal correction to Energy= 0.228339
Thermal correction to Enthalpy= 0.229284
Thermal correction to Gibbs Free Energy= 0.174424
Sum of electronic and zero-point Energies= -677.044912
Sum of electronic and thermal Energies= -677.031168
Sum of electronic and thermal Enthalpies= -677.030224
Sum of electronic and thermal Free Energies= -677.085083

TS for 7-NHCH$_3$[9MG + H$_{O6}$]$^{•+}$

C1 -0.311728 -0.325821 -0.477410
C2 0.262965 0.921649 -0.114889
C3 2.303601 0.074531 0.211972
C4 0.517089 -1.407070 -0.497219
C5 -1.863876 1.151534 -0.642019
N6 1.834569 -1.181363 -0.149338
N7 1.538794 1.138985 0.236259
O8 0.099521 -2.622530 -0.800704
N9 -0.724187 1.823283 -0.212171
N10 3.597494 0.166285 0.561630
H11 4.260920 -0.583044 0.456094
H12 3.948844 1.083044 0.793586
N13 -1.668331 -0.172232 -0.768224
N14 -2.815123 -1.043714 0.355235
H15 -2.885037 -1.901459 -0.193368
C16 -2.141814 -1.308085 1.609270
H17 -1.910350 -0.362296 2.110092
H18 -2.853184 -1.845305 2.248900
H19 -1.230252 -1.921999 1.548868
H20 -2.813743 1.644580 -0.772494
C21 -0.622739 3.256500 0.033218
H22 0.399799 3.481056 0.333767
H23 -0.864063 3.802444 -0.880197
H24 -1.311212 3.541667 0.830117
H25 2.466290 -1.968653 -0.086891
H26 0.792332 -3.287939 -0.884114

Zero-point correction= 0.210108 (Hartree/Particle)
Thermal correction to Energy= 0.224546
Thermal correction to Enthalpy= 0.225490
Thermal correction to Gibbs Free Energy= 0.168114
Sum of electronic and zero-point Energies= -677.001866
Sum of electronic and thermal Energies= -676.987429
Sum of electronic and thermal Enthalpies= -676.986485
Sum of electronic and thermal Free Energies= -677.043860

8-NHCH$_3$[9MG + H$_{O6}$]$^{•+}$

C1 -0.097236 0.754487 -0.239302
C2 -0.232162 -0.688612 -0.143970
C3 -2.450417 -0.609156 0.182116
C4 -1.267541 1.488431 -0.098719
C5 1.936001 -0.079180 -0.472656
N6 -2.435335 0.767428 0.102870
N7 -1.366805 -1.356798 0.068341
N8 0.969059 -1.195114 -0.305388
N9 -3.630790 -1.205394 0.379442
H10 -4.496277 -0.711500 0.523921
H11 -3.636902 -2.212989 0.438483

N12 1.153414 1.141474 -0.431205
N13 2.903081 -0.107157 0.595789
H14 3.427488 -0.975719 0.541952
C15 3.842068 1.020792 0.563744
H16 3.307326 1.945331 0.780893
H17 4.588937 0.861285 1.342365
H18 4.354299 1.132015 -0.402777
H19 2.380174 -0.164193 -1.483223
C20 1.338207 -2.596539 -0.244497
H21 0.436666 -3.202016 -0.335723
H22 2.016380 -2.828731 -1.068588
H23 1.823452 -2.815666 0.710396
H24 -3.315770 1.264608 0.129591
O25 -1.292648 2.804421 -0.181696
H26 -2.106473 3.219797 0.127045

Zero-point correction= 0.214058 (Hartree/Particle)
Thermal correction to Energy= 0.228106
Thermal correction to Enthalpy= 0.229050
Thermal correction to Gibbs Free Energy= 0.173040
Sum of electronic and zero-point Energies= -677.067551
Sum of electronic and thermal Energies= -677.053504
Sum of electronic and thermal Enthalpies= -677.052559
Sum of electronic and thermal Free Energies= -677.108570

TS for 8-NHCH$_3$[9MG + H$_{O6}$]$^{•+}$
C1 -0.092279 0.803223 -0.499554
C2 0.014225 -0.613230 -0.380159
C3 -2.114820 -0.903737 0.222116
C4 -1.323612 1.346241 -0.227216
C5 1.933319 0.323132 -0.927380
N6 -2.319976 0.461133 0.130795
N7 -0.951730 -1.465448 -0.024987
N8 1.280037 -0.908176 -0.702278
N9 -3.157977 -1.662223 0.596528
H10 -4.095738 -1.313121 0.704488
H11 -3.014134 -2.660188 0.631663
N12 1.092158 1.356000 -0.903562
N13 2.963483 0.509234 0.796959
H14 3.326306 1.461467 0.687904
C15 2.101630 0.480477 1.955157
H16 1.624523 -0.499627 2.049539
H17 2.723290 0.632962 2.848100
H18 1.337057 1.269875 1.961496
H19 2.854694 0.331452 -1.492959
C20 1.944121 -2.197630 -0.615134
H21 1.204652 -2.952608 -0.350923
H22 2.394484 -2.446636 -1.577225
H23 2.721252 -2.141396 0.151298
H24 -3.228648 0.828346 0.382604
O25 -1.560114 2.640844 -0.279475
H26 -2.484294 2.898220 -0.181356

Zero-point correction= 0.211021 (Hartree/Particle)
Thermal correction to Energy= 0.225077
Thermal correction to Enthalpy= 0.226021
Thermal correction to Gibbs Free Energy= 0.170292
Sum of electronic and zero-point Energies= -677.033413
Sum of electronic and thermal Energies= -677.019358
Sum of electronic and thermal Enthalpies= -677.018413
Sum of electronic and thermal Free Energies= -677.074142

[9MG + H$_{N3}$]$^{+}$···$^{•}$NHCH$_3$
C1 1.743925 0.436760 0.057490
C2 0.381123 0.584406 -0.071386
C3 -0.044835 -1.701076 -0.115514
C4 2.325579 -0.882152 0.112128
C5 1.365661 2.517914 0.029725
N6 -0.528185 -0.452090 -0.159055
O7 3.460038 -1.253961 0.220855
N8 0.123612 1.913755 -0.089574
N9 -0.892023 -2.733740 -0.195850
H10 -0.566740 -3.687180 -0.177713
H11 -1.882820 -2.570575 -0.294375
N12 2.345900 1.665477 0.119571
N13 -3.341328 -0.322348 -0.395221
H14 -3.863746 -0.300316 -1.275943
C15 -4.239422 -0.074455 0.694048
H16 -3.712844 -0.090141 1.649393
H17 -4.742511 0.896977 0.567159
H18 -5.047236 -0.822321 0.709972
C19 -1.168482 2.567958 -0.216504
H20 -1.615291 2.350347 -1.189561
H21 -1.837241 2.246293 0.585612
H22 -1.018803 3.643842 -0.131911
H23 1.460380 3.594974 0.042557
N24 1.275725 -1.895120 0.009876
H25 1.640111 -2.840938 0.044590
H26 -1.560772 -0.316938 -0.264770

Zero-point correction= 0.210824 (Hartree/Particle)
Thermal correction to Energy= 0.225793
Thermal correction to Enthalpy= 0.226737
Thermal correction to Gibbs Free Energy= 0.166891
Sum of electronic and zero-point Energies= -677.064587
Sum of electronic and thermal Energies= -677.049618
Sum of electronic and thermal Enthalpies= -677.048674
Sum of electronic and thermal Free Energies= -677.108520

TS for [9MG + H$_{N3}$]$^{+}$···$^{•}$NHCH$_3$
C1 -1.714346 0.028844 0.170907
C2 -0.483696 0.553815 -0.176794
C3 0.536034 -1.455652 -0.419007
C4 -1.877227 -1.401638 0.250285
C5 -1.974815 2.124945 0.096084
N6 0.682738 -0.134613 -0.427652
O7 -2.824358 -2.086908 0.531286
N8 -0.649032 1.905023 -0.227416
N9 1.631255 -2.228282 -0.676258
H10 1.513638 -3.229557 -0.731340
H11 2.284403 -1.854563 -1.349096
N12 -2.635939 1.026846 0.337870
N13 2.840778 0.670492 0.737867
H14 2.710801 1.173238 1.615984
C15 4.103463 0.044867 0.542232
H16 4.289056 -0.104841 -0.523357
H17 4.913422 0.604411 1.015158
H18 4.064417 -0.953451 1.018410
C19 0.341307 2.905526 -0.583592
H20 0.929835 3.207838 0.287358
H21 0.998677 2.508243 -1.359681
H22 -0.167702 3.783598 -0.981295
H23 -2.384252 3.125454 0.127946
N24 -0.632690 -2.052992 -0.116819
H25 -0.714684 -3.062644 -0.158901
H26 1.913164 0.387979 0.114688

Zero-point correction= 0.207028 (Hartree/Particle)
Thermal correction to Energy= 0.221250
Thermal correction to Enthalpy= 0.222195
Thermal correction to Gibbs Free Energy= 0.164478
Sum of electronic and zero-point Energies= -677.053678
Sum of electronic and thermal Energies= -677.039456
Sum of electronic and thermal Enthalpies= -677.038512
Sum of electronic and thermal Free Energies= -677.096228

[9MG + H$_{N3}$]$^{+}$
C1 0.293031 1.026668 -0.000015
C2 0.596496 -0.315046 -0.000005
C3 -1.647826 -1.005098 0.000005

C4 -1.083331 1.459417 -0.000013
C5 2.403517 0.882842 0.000028
N6 -0.342150 -1.337955 -0.000009
O7 -1.585229 2.544972 -0.000018
N8 1.945048 -0.425456 0.000033
N9 -2.584109 -1.957145 0.000008
H10 -3.565426 -1.723348 0.000010
H11 -2.349670 -2.937846 0.000062
N12 1.444958 1.764622 0.000006
C13 2.745040 -1.642464 -0.000035
H14 2.546262 -2.231005 0.899056
H15 2.547145 -2.230343 -0.899750
H16 3.797573 -1.361332 0.000622
H17 3.463260 1.097521 0.000051
N18 -1.975317 0.290777 0.000010
H19 -2.955440 0.553818 0.000034
H20 -0.062440 -2.309049 -0.000070

Zero-point correction=	0.158445 (Hartree/Particle)
Thermal correction to Energy=	0.168331
Thermal correction to Enthalpy=	0.169275
Thermal correction to Gibbs Free Energy=	0.123400
Sum of electronic and zero-point Energies=	-581.912412
Sum of electronic and thermal Energies=	-581.902526
Sum of electronic and thermal Enthalpies=	-581.901582
Sum of electronic and thermal Free Energies=	-581.947457

2-NHCH$_3$[9MG + H$_{N3}$]$^{\bullet+}$

C1 0.936061 1.021885 -0.164672
C2 0.852221 -0.355193 0.243523
C3 -1.571505 -0.165917 0.355841
C4 -0.227858 1.927533 0.022218
C5 2.867334 0.190602 -0.357493
N6 -0.265997 -0.911000 0.657023
O7 -0.201243 3.119328 -0.157570
N8 2.108404 -0.855036 0.124394
N9 -2.598118 -0.554352 1.243948
H10 -2.398077 -0.455158 2.230605
H11 -3.088576 -1.406471 1.015321
N12 2.181652 1.316632 -0.527004
N13 -1.836102 -0.411683 -1.037862
H14 -2.493301 0.284270 -1.374441
C15 -2.223693 -1.758881 -1.444694
H16 -1.430461 -2.470519 -1.196466
H17 -3.168922 -2.115805 -1.012565
H18 -2.332415 -1.766545 -2.529749
C19 2.545781 -2.218300 0.407737
H20 3.604952 -2.300243 0.166990
H21 2.408047 -2.442944 1.467310
H22 1.988614 -2.924285 -0.211933
H23 3.922019 0.069059 -0.567738
N24 -1.307139 1.242855 0.563799
H25 -2.131721 1.801932 0.750915
H26 -0.319157 -1.870196 0.977459

Zero-point correction=	0.214520 (Hartree/Particle)
Thermal correction to Energy=	0.227750
Thermal correction to Enthalpy=	0.228694
Thermal correction to Gibbs Free Energy=	0.174774
Sum of electronic and zero-point Energies=	-677.056827
Sum of electronic and thermal Energies=	-677.043597
Sum of electronic and thermal Enthalpies=	-677.042653
Sum of electronic and thermal Free Energies=	-677.096572

TS for 2-NHCH$_3$[9MG + H$_{N3}$]$^{\bullet+}$

C1 -0.657724 1.145289 0.074931
C2 -0.785028 -0.136701 -0.410571
C3 1.519880 -0.384707 -0.589465
C4 0.612912 1.850286 -0.020158
C5 -2.688585 0.606420 0.331541
N6 0.256719 -0.936100 -0.883781
O7 0.881462 2.991111 0.250430
N8 -2.082895 -0.496038 -0.242456
N9 2.561152 -1.065893 -1.147725
H10 3.484192 -0.658966 -1.140303
H11 2.527966 -2.074576 -1.132900
N12 -1.862757 1.594256 0.544305
N13 1.532135 -0.800251 1.238514
H14 2.124294 -0.222511 1.842808
C15 0.988772 -1.961315 1.858248
H16 0.429217 -2.543943 1.123863
H17 1.779109 -2.571259 2.317002
H18 0.315775 -1.652173 2.672386
C19 -2.712152 -1.746528 -0.637674
H20 -3.666052 -1.837633 -0.118977
H21 -2.890354 -1.769562 -1.715258
H22 -2.074425 -2.585626 -0.351129
H23 -3.745541 0.606235 0.560188
N24 1.632402 0.988543 -0.546898
H25 2.548253 1.418940 -0.594187
H26 0.190117 -1.265897 -1.841757

Zero-point correction=	0.210381 (Hartree/Particle)
Thermal correction to Energy=	0.224333
Thermal correction to Enthalpy=	0.225277
Thermal correction to Gibbs Free Energy=	0.168711
Sum of electronic and zero-point Energies=	-677.023709
Sum of electronic and thermal Energies=	-677.009757
Sum of electronic and thermal Enthalpies=	-677.008813
Sum of electronic and thermal Free Energies=	-677.065379

4-NHCH$_3$[9MG + H$_{N3}$]$^{\bullet+}$

C1 -0.180292 1.238171 0.165319
C2 0.565916 -0.080235 0.124631
C3 -1.544687 -1.068005 -0.544777
C4 -1.599688 1.220564 0.392119
C5 1.695012 1.725132 -0.677098
N6 -0.217572 -1.017763 -0.709406
O7 -2.353707 2.111742 0.693112
N8 1.763481 0.370447 -0.597437
N9 -2.255774 -2.056440 -1.093532
H10 -3.260924 -2.002431 -1.160176
H11 -1.810801 -2.892332 -1.439754
N12 0.562210 2.267782 -0.267675
N13 0.709273 -0.648560 1.424861
H14 0.950429 0.053530 2.113810
C15 1.387039 -1.925647 1.621545
H16 1.012293 -2.655147 0.898360
H17 2.478451 -1.878886 1.538653
H18 1.134031 -2.297160 2.615710
C19 2.994065 -0.392566 -0.752513
H20 3.578587 0.046667 -1.562401
H21 3.592358 -0.389907 0.162861
H22 2.764755 -1.425448 -1.022571
H23 2.523922 2.302100 -1.070260
N24 -2.149907 -0.108126 0.168897
H25 -3.132036 -0.196549 0.402662
H26 0.212422 -1.514243 -1.477099

Zero-point correction=	0.214727 (Hartree/Particle)
Thermal correction to Energy=	0.228098
Thermal correction to Enthalpy=	0.229042
Thermal correction to Gibbs Free Energy=	0.175286
Sum of electronic and zero-point Energies=	-677.062426
Sum of electronic and thermal Energies=	-677.049055
Sum of electronic and thermal Enthalpies=	-677.048111
Sum of electronic and thermal Free Energies=	-677.101867

TS for 4-NHCH$_3$[9MG + H$_{N3}$]$^{\bullet+}$

C1 0.000789 1.072507 -0.264282

C2 0.370612 -0.287856 -0.241887
C3 -1.883493 -0.991597 -0.126601
C4 -1.361985 1.466773 -0.027934
C5 1.986139 0.986413 -0.984735
N6 -0.595230 -1.304497 -0.361218
O7 -1.873959 2.547159 0.073379
N8 1.609273 -0.326739 -0.843039
N9 -2.797926 -1.960886 -0.056266
H10 -3.769573 -1.753319 0.117570
H11 -2.565675 -2.918703 -0.270862
N12 1.051017 1.844551 -0.658510
N13 0.861228 -0.677057 1.676871
H14 0.063834 -0.264426 2.173034
C15 2.042199 0.048651 2.097626
H16 2.932266 -0.352086 1.607267
H17 1.979350 1.130304 1.908478
H18 2.169993 -0.081101 3.180248
C19 2.429160 -1.512623 -1.015333
H20 3.412173 -1.210437 -1.375717
H21 2.539259 -2.028718 -0.056771
H22 1.980537 -2.181914 -1.753712
H23 2.974036 1.247853 -1.340264
N24 -2.231120 0.289725 0.040976
H25 -3.210578 0.531796 0.146989
H26 -0.295181 -2.245822 -0.136131

Zero-point correction=	0.211811 (Hartree/Particle)
Thermal correction to Energy=	0.225360
Thermal correction to Enthalpy=	0.226304
Thermal correction to Gibbs Free Energy=	0.171971
Sum of electronic and zero-point Energies=	-677.029937
Sum of electronic and thermal Energies=	-677.016388
Sum of electronic and thermal Enthalpies=	-677.015444
Sum of electronic and thermal Free Energies=	-677.069777

5-NHCH$_3$[9MG + H$_{N3}$]$^{•+}$

C1 -0.110679 -0.847796 0.156973
C2 -0.756637 0.476689 0.466658
C3 1.262915 1.660135 0.055449
C4 0.984342 -0.608251 -0.903858
C5 -2.207087 -0.864887 -0.518320
N6 0.024972 1.639062 0.564834
O7 1.332752 -1.340799 -1.781462
N8 -2.010737 0.450464 -0.093772
N9 2.098372 2.670273 0.307628
H10 3.067604 2.626886 0.031963
H11 1.801255 3.491495 0.811845
N12 -1.196988 -1.642434 -0.420254
N13 0.558600 -1.389946 1.316755
H14 -0.090109 -1.363603 2.097885
C15 1.109973 -2.743406 1.157428
H16 1.924724 -2.732529 0.430494
H17 1.523665 -3.050103 2.118895
H18 0.362382 -3.472346 0.829008
H19 -3.179668 -1.160440 -0.897929
C20 -3.015348 1.494178 -0.017794
H21 -3.355862 1.640559 1.011695
H22 -2.610845 2.430553 -0.411914
H23 -3.866942 1.205929 -0.634245
N24 1.653967 0.645142 -0.739020
H25 2.491349 0.746606 -1.301112
H26 -0.231733 2.355485 1.232702

Zero-point correction=	0.214545 (Hartree/Particle)
Thermal correction to Energy=	0.228292
Thermal correction to Enthalpy=	0.229236
Thermal correction to Gibbs Free Energy=	0.174162
Sum of electronic and zero-point Energies=	-677.045153
Sum of electronic and thermal Energies=	-677.031406
Sum of electronic and thermal Enthalpies=	-677.030462
Sum of electronic and thermal Free Energies=	-677.085537

TS for 5-NHCH$_3$[9MG + H$_{N3}$]$^{•+}$

C1 0.207463 0.693300 -0.278378
C2 0.716797 -0.598262 -0.021478
C3 -1.411756 -1.562007 0.066661
C4 -1.141421 0.735676 -0.872839
C5 2.335007 0.755893 -0.529714
N6 -0.092910 -1.669087 0.311380
O7 -1.683215 1.599124 -1.498922
N8 2.070408 -0.542555 -0.122306
N9 -2.253667 -2.528895 0.443885
H10 -3.251908 -2.402917 0.376791
H11 -1.927492 -3.392201 0.849349
N12 1.276213 1.490937 -0.688408
N13 -0.416103 1.301201 1.455162
H14 0.466457 1.154662 1.955182
C15 -0.634960 2.743057 1.390323
H16 -1.528927 2.960787 0.802629
H17 -0.811390 3.093565 2.415242
H18 0.211109 3.303030 0.976806
H19 3.353531 1.078945 -0.701447
C20 3.030782 -1.607588 0.117058
H21 3.083760 -1.853187 1.181126
H22 2.764142 -2.493913 -0.464554
H23 4.012787 -1.266376 -0.209478
N24 -1.860936 -0.485831 -0.598871
H25 -2.828802 -0.459024 -0.899170
H26 0.249950 -2.437170 0.873216

Zero-point correction=	0.211067 (Hartree/Particle)
Thermal correction to Energy=	0.225211
Thermal correction to Enthalpy=	0.226155
Thermal correction to Gibbs Free Energy=	0.169638
Sum of electronic and zero-point Energies=	-677.015946
Sum of electronic and thermal Energies=	-677.001802
Sum of electronic and thermal Enthalpies=	-677.000858
Sum of electronic and thermal Free Energies=	-677.057375

7-NHCH$_3$[9MG + H$_{N3}$]$^{•+}$

C1 -0.321460 -0.307199 0.004356
C2 0.471934 0.811139 -0.047447
C3 2.465213 -0.454001 0.012907
C4 0.227686 -1.615596 -0.043721
C5 -1.635004 1.534331 0.021876
N6 1.701786 -1.541668 0.021024
N7 1.858967 0.753088 -0.025864
O8 -0.270453 -2.711002 -0.112026
N9 -0.281699 1.920189 -0.102061
N10 3.800055 -0.528754 0.044599
H11 4.268970 -1.420793 0.082127
H12 4.382249 0.293971 0.039795
N13 -1.649992 0.128089 -0.058868
N14 -2.640408 -0.620120 0.606388
H15 -2.978616 -0.065303 1.388969
C16 -3.718867 -1.036013 -0.285114
H17 -3.316174 -1.725763 -1.028818
H18 -4.451294 -1.575287 0.318593
H19 -4.218012 -0.200849 -0.794479
H20 -2.390337 2.127256 -0.485522
C21 0.173325 3.296164 -0.003546
H22 1.029614 3.461618 -0.662601
H23 -0.631013 3.949307 -0.341310
H24 0.426179 3.555673 1.029025
H25 2.133534 -2.459684 0.028706
H26 2.410607 1.599150 -0.030667

Zero-point correction=	0.213105 (Hartree/Particle)
Thermal correction to Energy=	0.227446
Thermal correction to Enthalpy=	0.228391

Thermal correction to Gibbs Free Energy=	0.171519
Sum of electronic and zero-point Energies=	-677.009146
Sum of electronic and thermal Energies=	-676.994805
Sum of electronic and thermal Enthalpies=	-676.993860
Sum of electronic and thermal Free Energies=	-677.050732

TS for 7-$NHCH_3$[9MG + H_{N3}]$^{\bullet+}$

C1 -0.360225 -0.203980 -0.320052
C2 0.522144 0.827040 -0.096049
C3 2.370160 -0.612036 0.135892
C4 0.075488 -1.559677 -0.310851
C5 -1.474487 1.650627 -0.410559
N6 1.528916 -1.623386 -0.076440
N7 1.881048 0.644841 0.121231
O8 -0.506654 -2.602200 -0.452369
N9 -0.147059 1.992173 -0.137205
N10 3.672050 -0.820486 0.353251
H11 4.054569 -1.753662 0.362900
H12 4.315781 -0.061843 0.515468
N13 -1.652893 0.311845 -0.444239
N14 -2.592414 -0.325007 0.883624
H15 -3.094205 0.525232 1.154939
C16 -3.552296 -1.256153 0.303102
H17 -3.016928 -2.109295 -0.115882
H18 -4.185998 -1.617328 1.121129
H19 -4.193537 -0.814609 -0.468351
H20 -2.246220 2.395065 -0.521417
C21 0.375436 3.334360 0.057731
H22 1.260608 3.495738 -0.563368
H23 -0.384734 4.047726 -0.258939
H24 0.607641 3.514387 1.110894
H25 1.877843 -2.575807 -0.062194
H26 2.493553 1.431051 0.286947

Zero-point correction=	0.210740 (Hartree/Particle)
Thermal correction to Energy=	0.224934
Thermal correction to Enthalpy=	0.225879
Thermal correction to Gibbs Free Energy=	0.169523
Sum of electronic and zero-point Energies=	-676.997190
Sum of electronic and thermal Energies=	-676.982995
Sum of electronic and thermal Enthalpies=	-676.982051
Sum of electronic and thermal Free Energies=	-677.038406

8-$NHCH_3$[9MG + H_{N3}]$^{\bullet+}$

C1 -0.041800 0.777772 -0.308090
C2 -0.187439 -0.616670 -0.205254
C3 -2.508866 -0.481904 0.221123
C4 -1.200469 1.631336 -0.164210
C5 1.982730 -0.089391 -0.559547
N6 -2.404404 0.841624 0.098139
N7 -1.392152 -1.230766 0.068801
O8 -1.314025 2.824703 -0.222434
N9 0.979777 -1.205171 -0.424381
N10 -3.677600 -1.072688 0.488162
H11 -4.517117 -0.529523 0.621586
H12 -3.767290 -2.074466 0.557717
N13 1.215859 1.131451 -0.542371
N14 2.918273 -0.157718 0.540771
H15 3.603049 -0.882090 0.345777
C16 3.630173 1.096234 0.831714
H17 2.925078 1.838800 1.203185
H18 4.361350 0.885659 1.613407
H19 4.148745 1.516390 -0.039564
H20 2.458018 -0.201340 -1.550267
C21 1.348773 -2.589322 -0.218029
H22 0.532624 -3.255283 -0.512087
H23 2.197734 -2.824911 -0.862759
H24 1.626582 -2.771718 0.824798
H25 -3.232550 1.419214 0.200227
H26 -1.440906 -2.233817 0.187347

Zero-point correction=	0.214022 (Hartree/Particle)
Thermal correction to Energy=	0.228025
Thermal correction to Enthalpy=	0.228970
Thermal correction to Gibbs Free Energy=	0.172585
Sum of electronic and zero-point Energies=	-677.055424
Sum of electronic and thermal Energies=	-677.041421
Sum of electronic and thermal Enthalpies=	-677.040477
Sum of electronic and thermal Free Energies=	-677.096862

TS for 8-$NHCH_3$[9MG + H_{N3}]$^{\bullet+}$

C1 -0.052071 0.849652 -0.501305
C2 0.020118 -0.528278 -0.394798
C3 -2.236681 -0.765319 0.220709
C4 -1.294671 1.530318 -0.240775
C5 1.973197 0.302625 -0.912425
N6 -2.341228 0.561385 0.110276
N7 -1.041205 -1.338042 -0.026008
O8 -1.592736 2.690849 -0.260120
N9 1.270263 -0.906404 -0.722694
N10 -3.282500 -1.518967 0.573466
H11 -4.183332 -1.104864 0.759013
H12 -3.223397 -2.524067 0.626149
N13 1.148104 1.355350 -0.890532
N14 3.007239 0.407856 0.783340
H15 3.424809 1.333720 0.648188
C16 2.133805 0.481422 1.934564
H17 1.612695 -0.471701 2.075590
H18 2.753520 0.646958 2.826134
H19 1.401686 1.299704 1.895927
H20 2.882730 0.285069 -1.497586
C21 1.904284 -2.203221 -0.568150
H22 1.188237 -3.002101 -0.774087
H23 2.712373 -2.283872 -1.294830
H24 2.323254 -2.304909 0.436969
H25 -3.236020 1.005552 0.288325
H26 -0.927261 -2.337732 0.069319

Zero-point correction=	0.211388 (Hartree/Particle)
Thermal correction to Energy=	0.225257
Thermal correction to Enthalpy=	0.226201
Thermal correction to Gibbs Free Energy=	0.170722
Sum of electronic and zero-point Energies=	-677.025643
Sum of electronic and thermal Energies=	-677.011774
Sum of electronic and thermal Enthalpies=	-677.010830
Sum of electronic and thermal Free Energies=	-677.066309

Coordinates for structures in Table S3, optimized at ωB97XD/6-31+G(d,p)

2-$^{+}NH_2CH_3$[9MG]$^{\bullet}$
C1 -1.231962 0.909634 0.122096
C2 -0.810405 -0.440370 -0.101375
C3 1.423947 0.080796 -0.344809
C4 -0.229255 1.975354 0.089983
C5 -2.972642 -0.277260 0.191682
N6 0.390217 -0.907317 -0.304668
O7 -0.407968 3.161936 0.199158
N8 -1.976809 -1.174761 -0.048323
N9 2.281010 -0.021923 -1.480874
H10 1.997991 0.624825 -2.208591
H11 2.279054 -0.962353 -1.862944
N12 -2.554051 0.982236 0.300539
N13 2.351665 -0.302315 0.889007
H14 3.076700 0.417815 0.936464
C15 2.988187 -1.648339 0.842549
H16 2.206688 -2.393329 0.704562
H17 3.693138 -1.665269 0.012833
H18 3.517245 -1.816565 1.780383
C19 -2.090288 -2.616014 -0.218986
H20 -3.146725 -2.880404 -0.253995
H21 -1.611919 -2.912154 -1.153460
H22 -1.613869 -3.131652 0.616798
H23 -4.006323 -0.584552 0.281478
N24 1.061145 1.446823 -0.117882
H25 1.757692 2.153823 -0.327511
H26 1.776346 -0.207686 1.729285

Zero-point correction= 0.216713 (Hartree/Particle)
Thermal correction to Energy= 0.229947
Thermal correction to Enthalpy= 0.230891
Thermal correction to Gibbs Free Energy= 0.176204
Sum of electronic and zero-point Energies= -677.066536
Sum of electronic and thermal Energies= -677.053302
Sum of electronic and thermal Enthalpies= -677.052358
Sum of electronic and thermal Free Energies= -677.107045

TS for 2-$^{+}NH_2CH_3$[9MG]$^{\bullet}$
C1 1.108112 0.968475 -0.147912
C2 0.812539 -0.383397 0.219901
C3 -1.385818 0.018240 0.593559
C4 0.051316 1.975638 -0.028856
C5 2.908388 -0.111605 -0.334423
N6 -0.325768 -0.898799 0.598732
O7 0.120340 3.157149 -0.254191
N8 2.013933 -1.045624 0.086475
N9 -2.406315 -0.316684 1.440503
H10 -2.896746 0.406924 1.942570
H11 -2.302368 -1.196785 1.923511
N12 2.391538 1.108447 -0.487808
N13 -2.104379 -0.388802 -1.121431
H14 -2.843202 0.296047 -1.276958
C15 -2.631864 -1.758215 -1.227719
H16 -1.817661 -2.463437 -1.059715
H17 -3.392911 -1.893933 -0.457903
H18 -3.077816 -1.933679 -2.208916
C19 2.253529 -2.453499 0.375759
H20 3.299914 -2.676738 0.170376
H21 2.037523 -2.655367 1.425839
H22 1.617671 -3.075358 -0.255916
H23 3.945484 -0.358346 -0.520418
N24 -1.135128 1.387874 0.461072
H25 -1.884484 2.049483 0.626369
H26 -1.382503 -0.214712 -1.819953

Zero-point correction= 0.214362 (Hartree/Particle)
Thermal correction to Energy= 0.227536
Thermal correction to Enthalpy= 0.228480
Thermal correction to Gibbs Free Energy= 0.174459
Sum of electronic and zero-point Energies= -677.065295
Sum of electronic and thermal Energies= -677.052121
Sum of electronic and thermal Enthalpies= -677.051177
Sum of electronic and thermal Free Energies= -677.105198

2-$NHCH_3$[9MG + H_{N1}]$^{\bullet +}$
C1 1.162182 0.972838 -0.047819
C2 0.931514 -0.447511 -0.060190
C3 -1.392543 -0.415627 0.141212
C4 0.057558 1.893948 -0.120251
C5 3.066281 0.063368 -0.017487
N6 -0.166502 -1.141006 -0.047516
O7 -0.017588 3.076765 0.012816
N8 2.208408 -0.984436 -0.050700
N9 -1.754629 -0.171077 1.496617
H10 -0.973141 0.090167 2.088560
H11 -2.232588 -0.966551 1.904247
N12 2.473399 1.254070 -0.012474
N13 -2.395758 -0.991436 -0.642900
H14 -1.291599 1.020884 -1.468130
C15 -3.809940 -0.865460 -0.300061
H16 -4.102496 -1.456024 0.575718
H17 -4.067564 0.178860 -0.111032
H18 -4.394464 -1.203589 -1.155803
C19 2.526371 -2.407284 -0.047257
H20 3.608946 -2.520854 -0.004183
H21 2.072180 -2.882133 0.823296
H22 2.144621 -2.871260 -0.957585
H23 4.139152 -0.077931 0.000901
N24 -1.206929 1.109955 -0.449998
H25 -2.004226 1.661229 -0.118792
H26 -2.112580 -1.885049 -1.019807

Zero-point correction= 0.214110 (Hartree/Particle)
Thermal correction to Energy= 0.227953
Thermal correction to Enthalpy= 0.228898
Thermal correction to Gibbs Free Energy= 0.173081
Sum of electronic and zero-point Energies= -677.042656
Sum of electronic and thermal Energies= -677.028813
Sum of electronic and thermal Enthalpies= -677.027869
Sum of electronic and thermal Free Energies= -677.083685

TS for 2-$NHCH_3$[9MG + H_{N1}]$^{\bullet +}$
C1 1.152143 0.982876 -0.066230
C2 0.942629 -0.434878 0.036986
C3 -1.362825 -0.368117 0.316864
C4 0.026818 1.890596 0.000519
C5 3.058430 0.087972 -0.196101
N6 -0.152622 -1.119228 0.187908
O7 -0.006096 3.087091 0.019059
N8 2.215241 -0.966852 -0.056191
N9 -2.071171 -0.554837 1.533344
H10 -1.511013 -0.262369 2.329070
H11 -2.314105 -1.532269 1.667529
N12 2.452614 1.272402 -0.205179
N13 -2.201594 -0.622527 -0.903322
H14 -1.810896 0.698584 -0.989620
C15 -3.640608 -0.908599 -0.747658
H16 -3.812205 -1.869383 -0.256425
H17 -4.114530 -0.117358 -0.168153
H18 -4.077835 -0.933819 -1.745824
C19 2.548090 -2.384625 -0.007781
H20 3.630881 -2.490310 -0.064424
H21 2.186773 -2.815433 0.926847
H22 2.086846 -2.900894 -0.850992
H23 4.127755 -0.047245 -0.292968
N24 -1.244431 1.139122 0.040025

H25 -1.931202 1.668580 0.583600
H26 -1.746012 -1.322731 -1.486801

Zero-point correction= 0.211027 (Hartree/Particle)
Thermal correction to Energy= 0.223918
Thermal correction to Enthalpy= 0.224863
Thermal correction to Gibbs Free Energy= 0.171005
Sum of electronic and zero-point Energies= -677.017854
Sum of electronic and thermal Energies= -677.004962
Sum of electronic and thermal Enthalpies= -677.004018
Sum of electronic and thermal Free Energies= -677.057876

2-NHCH3[9MG + HN2]•+
C1 1.237799 0.916202 -0.066894
C2 0.806389 -0.445397 0.041761
C3 -1.440927 0.061440 0.127384
C4 0.225229 1.972122 -0.119212
C5 2.982508 -0.266073 -0.097015
N6 -0.402583 -0.921675 0.149925
O7 0.398148 3.156256 -0.259179
N8 1.976117 -1.176060 0.018080
N9 -2.064586 -0.086247 1.603508
H10 -1.412790 0.290151 2.294353
H11 -2.228419 -1.073410 1.814289
N12 2.568428 0.999119 -0.147449
N13 -2.544882 -0.217470 -0.727535
H14 -2.949974 0.424137 1.649691
C15 -2.988950 -1.606801 -0.869483
H16 -2.192794 -2.285771 -1.186929
H17 -3.413970 -1.975836 0.069184
H18 -3.793953 -1.619878 -1.604088
C19 2.081248 -2.627038 0.075087
H20 3.136178 -2.899554 0.065680
H21 1.619711 -2.997041 0.991716
H22 1.583803 -3.069653 -0.789350
H23 4.020623 -0.568221 -0.142942
N24 -1.071761 1.429519 -0.011821
H25 -1.816106 2.091520 -0.201778
H26 -2.382401 0.216485 -1.629127

Zero-point correction= 0.215743 (Hartree/Particle)
Thermal correction to Energy= 0.229232
Thermal correction to Enthalpy= 0.229232
Thermal correction to Gibbs Free Energy= 0.174977
Sum of electronic and zero-point Energies= -677.058656
Sum of electronic and thermal Energies= -677.045167
Sum of electronic and thermal Enthalpies= -677.044223
Sum of electronic and thermal Free Energies= -677.099422

TS for 2-NHCH3[9MG + HN2]•+
C1 1.173611 0.966877 -0.120102
C2 0.814995 -0.399154 0.115780
C3 -1.431584 0.009872 0.311149
C4 0.118417 1.981565 -0.119467
C5 2.966574 -0.139578 -0.173283
N6 -0.363250 -0.915189 0.331370
O7 0.230754 3.168516 -0.292350
N8 2.011788 -1.080008 0.072540
N9 -2.312200 -0.181128 1.538130
H10 -2.318678 0.607827 2.183180
H11 -2.066332 -1.034653 2.040628
N12 2.490788 1.099062 -0.293966
N13 -2.558809 -0.410050 -0.634672
H14 -3.137706 -0.337180 0.579419
C15 -2.488042 -1.750686 -1.248505
H16 -1.622514 -1.840411 -1.907316
H17 -2.424308 -2.510529 -0.469988
H18 -3.406263 -1.897344 -1.817651
C19 2.193029 -2.510202 0.277218
H20 3.236372 -2.758994 0.085858
H21 1.938837 -2.774924 1.305009
H22 1.555842 -3.064966 -0.412716
H23 4.013132 -0.400038 -0.261333
N24 -1.138566 1.394521 0.127472
H25 -1.902789 2.061218 0.122887
H26 -2.701888 0.299248 -1.352031

Zero-point correction= 0.211368 (Hartree/Particle)
Thermal correction to Energy= 0.224212
Thermal correction to Enthalpy= 0.225156
Thermal correction to Gibbs Free Energy= 0.171119
Sum of electronic and zero-point Energies= -677.027877
Sum of electronic and thermal Energies= -677.015033
Sum of electronic and thermal Enthalpies= -677.014089
Sum of electronic and thermal Free Energies= -677.068126

2-NHCH3-[9MG + HN3]•+
C1 0.936061 1.021885 -0.164672
C2 0.852221 -0.355193 0.243523
C3 -1.571505 -0.165917 0.355841
C4 -0.227858 1.927533 0.022218
C5 2.867334 0.190602 -0.357493
N6 -0.265997 -0.911000 0.657023
O7 -0.201243 3.119328 -0.157570
N8 2.108404 -0.855036 0.124394
N9 -2.598118 -0.554352 1.243948
H10 -2.398077 -0.455158 2.230605
H11 -3.088576 -1.406471 1.015321
N12 2.181652 1.316632 -0.527004
N13 -1.836102 -0.411683 -1.037862
H14 -2.493301 0.284270 -1.374441
C15 -2.223693 -1.758881 -1.444694
H16 -1.430461 -2.470519 -1.196466
H17 -3.168922 -2.115805 -1.012565
H18 -2.332415 -1.766545 -2.529749
C19 2.545781 -2.218300 0.407737
H20 3.604952 -2.300243 0.166990
H21 2.408047 -2.442944 1.467310
H22 1.988614 -2.924285 -0.211933
H23 3.922019 0.069059 -0.567738
N24 -1.307139 1.242855 0.563799
H25 -2.131721 1.801932 0.750915
H26 -0.319157 -1.870196 0.977459

Zero-point correction= 0.214520 (Hartree/Particle)
Thermal correction to Energy= 0.227750
Thermal correction to Enthalpy= 0.174774
Thermal correction to Gibbs Free Energy= 0.174977
Sum of electronic and zero-point Energies= -677.056827
Sum of electronic and thermal Energies= -677.043597
Sum of electronic and thermal Enthalpies= -677.042653
Sum of electronic and thermal Free Energies= -677.096572

TS for 2-NHCH3-[9MG + HN3]•+
C1 1.222772 0.933393 -0.018581
C2 0.911015 -0.458685 0.082661
C3 -1.454585 -0.023922 0.271694
C4 0.133518 1.927577 -0.103081
C5 3.049431 -0.117928 -0.124623
N6 -0.294625 -0.965335 0.207212
O7 0.278413 3.119309 -0.207326
N8 2.112591 -1.106501 -0.002218
N9 -2.183562 -0.046516 1.484320
H10 -1.752241 0.528252 2.198933
H11 -2.359383 -0.977353 1.845629
N12 2.536926 1.112337 -0.146301
N13 -2.188223 -0.781275 -0.811305
H14 -2.050895 -0.279125 -1.687810
C15 -3.605481 -1.149238 -0.638507
H16 -3.706203 -1.889282 0.155100

H17 -4.217345 -0.277030 -0.400649
H18 -3.945945 -1.598761 -1.571223
C19 2.330960 -2.547922 0.055767
H20 3.400660 -2.733156 0.145455
H21 1.821393 -2.957919 0.928962
H22 1.956141 -3.022035 -0.852809
H23 4.105873 -0.341820 -0.194480
N24 -1.130912 1.327769 -0.088374
H25 -1.894436 1.991637 -0.013882
H26 -1.076072 -1.560866 -0.549937

Zero-point correction=	0.210991 (Hartree/Particle)
Thermal correction to Energy=	0.223884
Thermal correction to Enthalpy=	0.224828
Thermal correction to Gibbs Free Energy=	0.171081
Sum of electronic and zero-point Energies=	-677.024907
Sum of electronic and thermal Energies=	-677.012014
Sum of electronic and thermal Enthalpies=	-677.011070
Sum of electronic and thermal Free Energies=	-677.064817

2-NHCH$_3$[9MG + H$_{O6}$]$^{•+}$

C1 -1.049193 0.889539 0.179829
C2 -0.748459 -0.458407 -0.162944
C3 1.541663 -0.087324 -0.332634
C4 -0.008073 1.846567 0.047441
C5 -2.882933 -0.135370 0.249318
N6 0.393281 -0.978772 -0.476022
O7 -0.169962 3.134589 0.171317
N8 -1.986355 -1.085335 -0.109342
N9 2.451399 -0.167511 -1.440431
H10 1.973995 -0.130937 -2.336486
H11 2.988875 -1.026774 -1.401844
N12 -2.356146 1.076677 0.422745
N13 2.141248 -0.370008 0.945427
H14 2.940570 0.243890 1.073318
C15 2.521709 -1.760777 1.214409
H16 1.634774 -2.393409 1.221236
H17 3.245395 -2.176717 0.499969
H18 2.970106 -1.790898 2.207963
C19 -2.227862 -2.501212 -0.360070
H20 -3.295966 -2.695318 -0.269683
H21 -1.891915 -2.754630 -1.366142
H22 -1.681465 -3.100183 0.369831
H23 -3.933204 -0.361536 0.378110
N24 1.169730 1.378491 -0.291734
H25 1.928534 2.023485 -0.491058
H26 -1.093203 3.353425 0.377652

Zero-point correction=	0.215310 (Hartree/Particle)
Thermal correction to Energy=	0.228621
Thermal correction to Enthalpy=	0.229565
Thermal correction to Gibbs Free Energy=	0.174522
Sum of electronic and zero-point Energies=	-677.061655
Sum of electronic and thermal Energies=	-677.048343
Sum of electronic and thermal Enthalpies=	-677.047399
Sum of electronic and thermal Free Energies=	-677.102443

TS for 2-NHCH$_3$[9MG + H$_{O6}$]$^{•+}$

C1 -0.781960 1.157635 -0.317961
C2 -0.891828 -0.289818 -0.304096
C3 1.383100 -0.615860 -0.496294
C4 0.597604 1.542071 -0.391685
C5 -2.792443 0.722541 0.124502
N6 0.059476 -1.173773 -0.295461
O7 1.244509 2.174475 0.508214
N8 -2.231625 -0.501988 -0.029296
N9 2.269047 -1.430286 -1.250677
H10 1.949597 -1.512374 -2.211588
H11 2.330878 -2.362247 -0.850799
N12 -1.939542 1.740519 -0.001962
N13 2.037681 -0.248303 0.810916
H14 3.019870 -0.502139 0.707238
C15 1.495308 -0.816306 2.064959
H16 0.511667 -0.392390 2.268047
H17 1.416463 -1.904705 2.020060
H18 2.167382 -0.528408 2.873879
C19 -2.864970 -1.808052 0.115370
H20 -3.924545 -1.659046 0.319771
H21 -2.745886 -2.374966 -0.808404
H22 -2.402073 -2.349791 0.941461
H23 -3.847749 0.845728 0.329517
N24 1.312516 0.707375 -1.221012
H25 2.270905 1.038159 -1.331351
H26 1.875682 1.298304 0.820129

Zero-point correction=	0.212054 (Hartree/Particle)
Thermal correction to Energy=	0.224065
Thermal correction to Enthalpy=	0.225009
Thermal correction to Gibbs Free Energy=	0.173796
Sum of electronic and zero-point Energies=	-676.996097
Sum of electronic and thermal Energies=	-676.984085
Sum of electronic and thermal Enthalpies=	-676.983141
Sum of electronic and thermal Free Energies=	-677.034355

2-NHCH$_3$[9MG + H$_{N7}$]$^{•+}$

C1 -1.038855 0.898134 0.131828
C2 -0.759181 -0.431222 -0.169374
C3 1.548777 -0.028075 -0.319758
C4 -0.016439 1.947318 0.025482
C5 -2.923480 -0.227837 0.243127
N6 0.400169 -0.964317 -0.444644
O7 -0.258712 3.128889 0.174798
N8 -1.989093 -1.109557 -0.088754
N9 2.475500 -0.192996 -1.400086
H10 2.050970 -0.006614 -2.303015
H11 2.883323 -1.120934 -1.410538
N12 -2.374072 0.990690 0.378257
N13 2.084660 -0.326221 0.985459
H14 2.643122 0.457196 1.305987
C15 2.798402 -1.592983 1.148571
H16 2.164546 -2.416989 0.815433
H17 3.755199 -1.631501 0.611072
H18 3.000196 -1.735833 2.211086
C19 -2.159742 -2.545150 -0.312009
H20 -3.210383 -2.801914 -0.184710
H21 -1.834293 -2.786300 -1.323953
H22 -1.548965 -3.088488 0.409259
H23 -3.970285 -0.452048 0.387344
N24 1.170382 1.394891 -0.355056
H25 1.932304 2.036881 -0.540907
H26 -2.865752 1.846879 0.611125

Zero-point correction=	0.215369 (Hartree/Particle)
Thermal correction to Energy=	0.228550
Thermal correction to Enthalpy=	0.229494
Thermal correction to Gibbs Free Energy=	0.175223
Sum of electronic and zero-point Energies=	-677.061333
Sum of electronic and thermal Energies=	-677.048152
Sum of electronic and thermal Enthalpies=	-677.047208
Sum of electronic and thermal Free Energies=	-677.101479

TS for 2-NHCH$_3$[9MG + H$_{N7}$]$^{•+}$

C1 -0.161487 1.317879 -0.063496
C2 -0.755475 0.253973 -0.805373
C3 1.192294 -0.808525 -0.398792
C4 1.299210 1.581744 -0.179629
C5 -2.064246 0.766600 0.846743
N6 -0.131985 -0.833790 -1.090966
O7 1.838623 2.635778 0.047014
N8 -2.128042 0.241923 -0.361668

N9 2.035558 -1.898519 -0.715260
H10 2.457311 -1.778148 -1.631311
H11 1.525457 -2.774991 -0.723724
N12 -0.843878 1.310593 1.155956
N13 0.797618 -0.835142 1.102629
H14 1.687356 -0.743824 1.596396
C15 0.149654 -2.082039 1.561776
H16 -0.776639 -2.239481 1.006769
H17 0.800133 -2.951522 1.436036
H18 -0.080511 -1.976715 2.623295
C19 -3.214287 -0.509382 -0.980685
H20 -4.024067 -0.626363 -0.261182
H21 -3.570547 0.028013 -1.860174
H22 -2.827516 -1.486746 -1.275939
H23 -2.888897 0.735734 1.549009
N24 1.912410 0.421507 -0.605161
H25 2.926020 0.396308 -0.621082
H26 -0.102831 0.364008 1.503818

Zero-point correction=	0.209940 (Hartree/Particle)
Thermal correction to Energy=	0.222325
Thermal correction to Enthalpy=	0.223270
Thermal correction to Gibbs Free Energy=	0.171667
Sum of electronic and zero-point Energies=	-676.951406
Sum of electronic and thermal Energies=	-676.939021
Sum of electronic and thermal Enthalpies=	-676.938077
Sum of electronic and thermal Free Energies=	-676.989680

8-$^{+}$NH$_2$CH$_3$[9MG]$^{•}$

C1 -0.117075 0.760317 -0.228921
C2 -0.313302 -0.631342 -0.052691
C3 -2.524387 -0.490804 0.200691
C4 -1.274745 1.628416 -0.143550
C5 1.847985 -0.162759 -0.451646
N6 -2.458792 0.863015 0.077901
N7 -1.450166 -1.280091 0.151546
O8 -1.337131 2.832372 -0.237193
N9 0.923398 -1.218931 -0.134866
N10 -3.720628 -1.063058 0.387543
H11 -4.581826 -0.542780 0.420454
H12 -3.755882 -2.067109 0.470898
N13 1.158599 1.080634 -0.467792
N14 2.919684 -0.060582 0.675035
H15 3.354543 -0.974617 0.823074
C16 3.960488 0.976265 0.401092
H17 3.450579 1.926974 0.253612
H18 4.644612 1.022971 1.247504
H19 4.499793 0.694391 -0.503287
H20 2.436799 -0.364158 -1.358467
C21 1.147526 -2.621145 -0.452574
H22 0.255672 -3.177873 -0.164667
H23 1.325505 -2.759373 -1.524271
H24 1.995237 -3.017857 0.112053
H25 -3.300364 1.425200 0.129993
H26 2.408777 0.174652 1.530672

Zero-point correction=	0.216944 (Hartree/Particle)
Thermal correction to Energy=	0.230285
Thermal correction to Enthalpy=	0.231229
Thermal correction to Gibbs Free Energy=	0.176684
Sum of electronic and zero-point Energies=	-677.084702
Sum of electronic and thermal Energies=	-677.071360
Sum of electronic and thermal Enthalpies=	-677.070416
Sum of electronic and thermal Free Energies=	-677.124961

TS for 8-$^{+}$NH$_2$CH$_3$[9MG]$^{•}$

C1 -0.120381 0.730542 -0.400464
C2 -0.330402 -0.667026 -0.201563
C3 -2.491901 -0.468693 0.297935
C4 -1.250826 1.627254 -0.208792
C5 1.761602 -0.216896 -0.725856
N6 -2.416882 0.883885 0.142618
N7 -1.446090 -1.288042 0.143518
O8 -1.296883 2.827888 -0.304533
N9 0.872623 -1.250479 -0.462465
N10 -3.665584 -1.011860 0.628770
H11 -4.509223 -0.475158 0.751687
H12 -3.709448 -2.014656 0.731114
N13 1.130700 0.995799 -0.762541
N14 2.899583 -0.002434 0.929027
H15 3.329423 -0.880474 1.211852
C16 3.900418 1.049759 0.688837
H17 3.386305 1.938920 0.321725
H18 4.452029 1.292420 1.600300
H19 4.604445 0.705444 -0.070820
H20 2.631119 -0.415124 -1.341984
C21 1.173743 -2.671898 -0.415954
H22 0.257196 -3.226089 -0.617613
H23 1.913144 -2.909831 -1.181888
H24 1.552314 -2.959829 0.568519
H25 -3.239392 1.460589 0.279731
H26 2.233181 0.274365 1.646295

Zero-point correction=	0.214257 (Hartree/Particle)
Thermal correction to Energy=	0.227795
Thermal correction to Enthalpy=	0.228739
Thermal correction to Gibbs Free Energy=	0.173300
Sum of electronic and zero-point Energies=	-677.081733
Sum of electronic and thermal Energies=	-677.068195
Sum of electronic and thermal Enthalpies=	-677.067251
Sum of electronic and thermal Free Energies=	-677.122690

8-NHCH$_3$[9MG + H$_{N3}$]$^{•+}$

C1 -0.041800 0.777772 -0.308090
C2 -0.187439 -0.616670 -0.205254
C3 -2.508866 -0.481904 0.221123
C4 -1.200469 1.631336 -0.164210
C5 1.982730 -0.089391 -0.559547
N6 -2.404404 0.841624 0.098139
N7 -1.392152 -1.230766 0.068801
O8 -1.314025 2.824703 -0.222434
N9 0.979777 -1.205171 -0.424381
N10 -3.677600 -1.072688 0.488162
H11 -4.517117 -0.529523 0.621586
H12 -3.767290 -2.074466 0.557717
N13 1.215859 1.131451 -0.542371
N14 2.918273 -0.157718 0.540771
H15 3.603049 -0.882090 0.345777
C16 3.630173 1.096234 0.831714
H17 2.925078 1.838800 1.203185
H18 4.361350 0.885659 1.613407
H19 4.148745 1.516390 -0.039564
H20 2.458018 -0.201340 -1.550267
C21 1.348773 -2.589322 -0.218029
H22 0.532624 -3.255283 -0.512087
H23 2.197734 -2.824911 -0.862759
H24 1.626582 -2.771718 0.824798
H25 -3.232550 1.419214 0.200227
H26 -1.440906 -2.233817 0.187347

Zero-point correction=	0.214022 (Hartree/Particle)
Thermal correction to Energy=	0.228025
Thermal correction to Enthalpy=	0.228970
Thermal correction to Gibbs Free Energy=	0.172585
Sum of electronic and zero-point Energies=	-677.055424
Sum of electronic and thermal Energies=	-677.041421
Sum of electronic and thermal Enthalpies=	-677.040477
Sum of electronic and thermal Free Energies=	-677.096862

TS for 8-NHCH$_3$[9MG + H$_{N3}$]$^{•+}$
C1 0.307984 -1.102032 -0.457193
C2 -0.198632 0.102486 -1.129170
C3 1.494267 1.248783 0.010367
C4 1.787745 -1.181796 -0.305079
C5 -1.779480 -0.830959 0.054619
N6 2.290332 0.154144 -0.182633
N7 0.203103 1.243485 -0.353998
O8 2.514775 -2.128630 -0.267604
N9 -1.567790 -0.131721 -1.197161
N10 2.008031 2.334694 0.583580
H11 2.919449 2.342709 1.016182
H12 1.479149 3.194366 0.559592
N13 -0.587070 -1.683817 0.247770
N14 -1.676271 0.240607 1.120607
H15 -2.546537 0.771255 1.092623
C16 -1.465400 -0.235126 2.506065
H17 -0.486170 -0.704795 2.587489
H18 -1.515061 0.623281 3.177139
H19 -2.225964 -0.965191 2.795128
H20 -2.715893 -1.388803 0.111675
C21 -2.501733 0.888171 -1.648834
H22 -2.253390 1.153099 -2.677010
H23 -3.508294 0.464926 -1.642859
H24 -2.486200 1.803930 -1.042490
H25 3.297627 0.223379 -0.083245
H26 -0.637779 1.071966 0.534804

Zero-point correction=	0.211816 (Hartree/Particle)
Thermal correction to Energy=	0.224017
Thermal correction to Enthalpy=	0.224961
Thermal correction to Gibbs Free Energy=	0.173570
Sum of electronic and zero-point Energies=	-676.989512
Sum of electronic and thermal Energies=	-676.977311
Sum of electronic and thermal Enthalpies=	-676.976367
Sum of electronic and thermal Free Energies=	-677.027758

8-NHCH$_3$[9MG + H$_{O6}$]$^{•+}$
C1 -0.097236 0.754487 -0.239302
C2 -0.232162 -0.688612 -0.143970
C3 -2.450417 -0.609156 0.182116
C4 -1.267541 1.488431 -0.098719
C5 1.936001 -0.079180 -0.472656
N6 -2.435335 0.767428 0.102870
N7 -1.366805 -1.356798 0.068341
N8 0.969059 -1.195114 -0.305388
N9 -3.630790 -1.205394 0.379442
H10 -4.496277 -0.711500 0.523921
H11 -3.636902 -2.212989 0.438483
N12 1.153414 1.141474 -0.431205
N13 2.903081 -0.107157 0.595789
H14 3.427488 -0.975719 0.541952
C15 3.842068 1.020792 0.563744
H16 3.307326 1.945331 0.780893
H17 4.588937 0.861285 1.342365
H18 4.354299 1.132015 -0.402777
H19 2.380174 -0.164193 -1.483223
C20 1.338207 -2.596539 -0.244497
H21 0.436666 -3.202016 -0.335723
H22 2.016380 -2.828731 -1.068588
H23 1.823452 -2.815666 0.710396
H24 -3.315770 1.264608 0.129591
O25 -1.292648 2.804421 -0.181696
H26 -2.106473 3.219797 0.127045

Zero-point correction=	0.214058 (Hartree/Particle)
Thermal correction to Energy=	0.228106
Thermal correction to Enthalpy=	0.229050
Thermal correction to Gibbs Free Energy=	0.173040
Sum of electronic and zero-point Energies=	-677.067551
Sum of electronic and thermal Energies=	-677.053504
Sum of electronic and thermal Enthalpies=	-677.052559
Sum of electronic and thermal Free Energies=	-677.108570

TS for 8-NHCH$_3$[9MG + H$_{O6}$]$^{•+}$
C1 -0.072619 -0.399347 1.080452
C2 -0.464974 0.824605 0.441576
C3 -2.458474 0.009156 -0.129949
C4 -0.601834 -1.419840 0.271195
C5 1.758876 0.580595 0.525300
N6 -1.923782 -1.275932 -0.117457
N7 -1.697650 1.084040 0.009306
N8 0.666631 1.510422 0.209128
N9 -3.760298 0.131364 -0.386453
H10 -4.384111 -0.659788 -0.402221
H11 -4.151828 1.060343 -0.437298
N12 1.238409 -0.400453 1.432882
N13 2.016702 -0.366290 -0.660905
H14 1.867194 0.127776 -1.541683
C15 3.389158 -0.925456 -0.630174
H16 3.527445 -1.441075 0.320772
H17 3.490777 -1.646313 -1.442046
H18 4.143203 -0.141907 -0.740687
H19 2.672861 1.093455 0.829510
C20 0.806353 2.761490 -0.516345
H21 -0.182510 3.207281 -0.625674
H22 1.448083 3.444805 0.042921
H23 1.228623 2.598379 -1.513197
H24 -2.299075 -1.972089 -0.751892
O25 0.198347 -2.197136 -0.358366
H26 1.133563 -1.463056 -0.529425

Zero-point correction=	0.211620 (Hartree/Particle)
Thermal correction to Energy=	0.223701
Thermal correction to Enthalpy=	0.224646
Thermal correction to Gibbs Free Energy=	0.173585
Sum of electronic and zero-point Energies=	-677.011024
Sum of electronic and thermal Energies=	-676.998943
Sum of electronic and thermal Enthalpies=	-676.997999
Sum of electronic and thermal Free Energies=	-677.049059

8-NHCH$_3$[9MG + H$_{N7}$]$^{•+}$
C1 0.167710 -0.781735 -0.226485
C2 0.272830 0.634800 -0.142715
C3 2.489349 0.560526 0.192220
C4 1.336778 -1.604579 -0.095398
C5 -1.949201 0.096984 -0.451161
N6 2.485065 -0.804339 0.126483
N7 1.394416 1.311917 0.062301
O8 1.398111 -2.814546 -0.156128
N9 -0.944475 1.153315 -0.334003
N10 3.651456 1.181141 0.399233
H11 4.527383 0.696625 0.512786
H12 3.644637 2.188968 0.450435
N13 -1.108881 -1.103741 -0.471089
N14 -2.834430 0.094255 0.682892
H15 -3.207855 1.027847 0.821956
C16 -3.928895 -0.873082 0.571099
H17 -3.544187 -1.888858 0.697018
H18 -4.632156 -0.696999 1.384986
H19 -4.472804 -0.810732 -0.383027
H20 -2.469358 0.185448 -1.422042
C21 -1.280304 2.565893 -0.316451
H22 -0.375738 3.135704 -0.528114
H23 -2.024445 2.770437 -1.089167
H24 -1.665062 2.865250 0.662914
H25 -1.462055 -2.047818 -0.538316
H26 3.345112 -1.330189 0.232221

Zero-point correction=	0.215662 (Hartree/Particle)

Thermal correction to Energy=	0.229180
Thermal correction to Enthalpy=	0.230124
Thermal correction to Gibbs Free Energy=	0.175125
Sum of electronic and zero-point Energies=	-677.104119
Sum of electronic and thermal Energies=	-677.090601
Sum of electronic and thermal Enthalpies=	-677.089656
Sum of electronic and thermal Free Energies=	-677.144655

TS for 8-NHCH$_3$[9MG + H$_{N7}$]$^{•+}$

C1 -0.198290 0.801129 -0.261963
C2 -0.286140 -0.613058 -0.110013
C3 -2.505194 -0.603911 0.173308
C4 -1.394556 1.594363 -0.103357
C5 1.874767 -0.034732 -0.468552
N6 -2.529018 0.754648 0.099842
N7 -1.382896 -1.324864 0.092391
O8 -1.515535 2.797169 -0.131648
N9 0.972014 -1.118627 -0.179580
N10 -3.656120 -1.258103 0.346386
H11 -4.550010 -0.798120 0.408774
H12 -3.623425 -2.264907 0.400155
N13 1.068829 1.189288 -0.493144
N14 2.762642 0.431842 0.647155
H15 2.524154 -0.033163 1.522585
C16 4.217161 0.480011 0.411471
H17 4.410375 1.077263 -0.480762
H18 4.689906 0.964015 1.265618
H19 4.632363 -0.522537 0.281034
H20 2.470077 -0.215207 -1.370685
C21 1.317901 -2.523254 -0.324341
H22 0.444663 -3.116992 -0.054856
H23 1.600331 -2.745305 -1.358046
H24 2.141922 -2.780438 0.344718
H25 1.983930 1.474094 0.412006
H26 -3.402064 1.261370 0.191970

Zero-point correction=	0.211340 (Hartree/Particle)
Thermal correction to Energy=	0.224442
Thermal correction to Enthalpy=	0.225386
Thermal correction to Gibbs Free Energy=	0.171109
Sum of electronic and zero-point Energies=	-677.055460
Sum of electronic and thermal Energies=	-677.042359
Sum of electronic and thermal Enthalpies=	-677.041414
Sum of electronic and thermal Free Energies=	-677.095691

Cartesian coordinates for structures in Table S4, optimized at ωB97XD/6-31+G(d,p)

[9MG – H]$^+$
N1 -1.978600 0.207417 -0.002507
C2 -0.073662 -1.020324 -0.000165
C3 1.403649 -1.227662 0.002245
O4 1.950093 -2.297275 0.004621
N5 2.049536 -0.000673 0.001956
C6 1.495730 1.282972 -0.001399
N7 2.269459 2.287660 -0.004989
H8 1.746362 3.163157 -0.006815
N9 0.086128 1.439768 -0.001067
C10 -0.571656 0.356291 0.000846
C11 -2.950067 1.305429 0.008385
H12 -2.859046 1.848000 0.949893
H13 -2.736388 1.970459 -0.828488
H14 -3.949420 0.884546 -0.090847
C15 -2.191604 -1.090376 -0.005744
N16 -1.048780 -1.873592 -0.004674
H17 -3.186233 -1.522437 -0.009370
H18 3.065441 -0.027559 0.002617

Zero-point correction= 0.133878 (Hartree/Particle)
Thermal correction to Energy= 0.143579
Thermal correction to Enthalpy= 0.144523
Thermal correction to Gibbs Free Energy= 0.098460
Sum of electronic and zero-point Energies= -580.623128
Sum of electronic and thermal Energies= -580.613427
Sum of electronic and thermal Enthalpies= -580.612482
Sum of electronic and thermal Free Energies= -580.658546

2-$^+NH_2CH_3$[9MG – H_{N2}]
C1 1.606436 0.612576 0.086249
C2 0.798574 -0.502982 -0.064416
C3 -1.085082 0.671876 -0.238019
C4 1.026487 1.935167 0.066909
C5 2.891874 -1.063010 0.146321
N6 -0.415568 1.829122 -0.114754
N7 -0.567408 -0.517050 -0.215884
O8 1.514885 3.028933 0.163119
N9 1.625196 -1.581188 -0.024477
N10 -2.485076 0.747552 -0.460906
H11 -2.991383 1.409960 0.119484
N12 2.915756 0.241025 0.216288
N13 -3.055330 -0.556145 -0.387461
H14 -2.183583 -1.160984 -0.441726
C15 -3.807480 -0.859168 0.864019
H16 -4.643395 -0.165531 0.958626
H17 -4.178783 -1.882051 0.795598
H18 -3.117427 -0.761338 1.702076
H19 3.755576 -1.710814 0.210101
C20 1.254763 -2.979995 -0.152351
H21 0.780706 -3.158708 -1.119640
H22 2.159289 -3.584117 -0.086584
H23 0.577701 -3.269553 0.653998
H24 -3.631862 -0.701915 -1.219522
H25 -0.882321 2.723597 -0.219274

Zero-point correction= 0.206687 (Hartree/Particle)
Thermal correction to Energy= 0.219128
Thermal correction to Enthalpy= 0.220072
Thermal correction to Gibbs Free Energy= 0.167645
Sum of electronic and zero-point Energies= -676.461599
Sum of electronic and thermal Energies= -676.449158
Sum of electronic and thermal Enthalpies= -676.448214
Sum of electronic and thermal Free Energies= -676.500640

2-$NHCH_3$[9MG – H_{N2} + H_{N1}]$^+$
C1 1.388785 0.951475 0.031030
C2 1.171248 -0.433070 0.014912
C3 -1.068583 -0.475171 -0.133449
C4 0.335602 1.880310 -0.038152
C5 3.304405 0.066342 0.081973
N6 -1.087024 0.961261 0.150260
N7 0.016773 -1.157254 -0.111727
O8 0.125013 3.035639 -0.113545
N9 2.401461 -0.985637 0.051739
N10 -2.270960 -1.002104 -0.421800
H11 -2.311273 -2.001766 -0.598888
N12 2.735394 1.234184 0.059024
N13 -3.417791 -0.206195 -0.417210
H14 -3.841109 -0.252660 -1.339923
C15 -4.380176 -0.588339 0.624583
H16 -3.936236 -0.422091 1.608291
H17 -5.252410 0.059266 0.523033
H18 -4.698956 -1.634336 0.546741
H19 4.370062 -0.113061 0.120872
C20 2.716347 -2.409530 0.067488
H21 1.984254 -2.942613 -0.538435
H22 3.710419 -2.551604 -0.355402
H23 2.692995 -2.789758 1.090315
H24 -1.864478 1.405917 -0.354824
H25 -1.304111 1.095706 1.144275

Zero-point correction= 0.203995 (Hartree/Particle)
Thermal correction to Energy= 0.217359
Thermal correction to Enthalpy= 0.218303
Thermal correction to Gibbs Free Energy= 0.162966
Sum of electronic and zero-point Energies= -676.435625
Sum of electronic and thermal Energies= -676.422261
Sum of electronic and thermal Enthalpies= -676.421317
Sum of electronic and thermal Free Energies= -676.476654

TS for 2-NH_2CH_3[9MG – H_{N2} + H_{N1}]
C1 1.226897 0.983372 -0.008165
C2 1.243513 -0.407004 0.050452
C3 -0.946779 -0.696571 0.123364
C4 -0.007713 1.709017 0.007805
C5 3.259640 0.412558 -0.073374
N6 -1.162239 0.716325 0.311576
N7 0.174419 -1.281073 0.007859
O8 -0.308021 2.857486 -0.081280
N9 2.546355 -0.766723 0.004543
N10 -2.186000 -1.304776 -0.029162
H11 -2.192216 -2.236595 -0.429327
N12 2.501524 1.475437 -0.094040
N13 -3.142785 -0.371973 -0.514772
H14 -3.310509 -0.512044 -1.512936
C15 -4.408872 -0.366958 0.245819
H16 -4.190391 -0.124896 1.286105
H17 -5.052337 0.401367 -0.185162
H18 -4.901132 -1.339590 0.192539
H19 4.340266 0.411974 -0.110886
C20 3.085036 -2.120263 0.054779
H21 2.417180 -2.790954 -0.485777
H22 4.064650 -2.124262 -0.422301
H23 3.179080 -2.452377 1.090239
H24 -2.300423 0.638198 -0.263310
H25 -1.419242 0.913877 1.284953

Zero-point correction= 0.201073 (Hartree/Particle)
Thermal correction to Energy= 0.213391
Thermal correction to Enthalpy= 0.214335
Thermal correction to Gibbs Free Energy= 0.161740
Sum of electronic and zero-point Energies= -676.424110
Sum of electronic and thermal Energies= -676.411793
Sum of electronic and thermal Enthalpies= -676.410849
Sum of electronic and thermal Free Energies= -676.463443

2-NHCH3[9MG]$^+$

C1 1.266157 0.973496 0.027814
C2 1.121670 -0.405841 -0.077022
C3 -1.037620 -0.329190 -0.459835
C4 0.117631 1.837305 -0.136880
C5 3.184478 0.145820 0.324476
N6 -1.059379 1.020126 -0.398780
N7 -0.028221 -1.103898 -0.327898
O8 -0.007910 3.031691 -0.096480
N9 2.362033 -0.931703 0.114833
N10 -2.317623 -1.019488 -0.721348
H11 -2.828510 -0.566449 -1.496893
N12 2.563303 1.300505 0.277994
N13 -3.130101 -1.205047 0.465663
H14 -2.019958 -1.952421 -1.033609
C15 -3.839573 0.013552 0.859443
H16 -4.387201 0.490419 0.033340
H17 -4.559146 -0.284696 1.622625
H18 -3.146912 0.718634 1.320450
H19 4.242307 0.014994 0.507685
C20 2.726699 -2.341041 0.113234
H21 2.486956 -2.789501 -0.851986
H22 3.799372 -2.419782 0.287042
H23 2.194548 -2.866104 0.908120
H24 -3.783151 -1.951703 0.238369
H25 -1.901761 1.565021 -0.543927

Zero-point correction= 0.206343 (Hartree/Particle)
Thermal correction to Energy= 0.218984
Thermal correction to Enthalpy= 0.219929
Thermal correction to Gibbs Free Energy= 0.166488
Sum of electronic and zero-point Energies= -676.432756
Sum of electronic and thermal Energies= -676.420115
Sum of electronic and thermal Enthalpies= -676.419170
Sum of electronic and thermal Free Energies= -676.472611

TS for 2-NHCH3[9MG]$^+$

C1 1.294544 0.904213 0.085085
C2 0.943273 -0.418459 -0.168623
C3 -1.160595 0.030790 -0.650547
C4 0.302248 1.949687 -0.043218
C5 3.043584 -0.241804 0.366768
N6 -0.961461 1.356913 -0.426563
N7 -0.278328 -0.901532 -0.540019
O8 0.365824 3.140604 0.116605
N9 2.078944 -1.149785 0.014782
N10 -2.485069 -0.387024 -0.988731
H11 -3.050998 0.246941 -1.550341
N12 2.609399 0.995930 0.420642
N13 -3.306951 -1.005577 0.179072
H14 -2.730265 -1.567621 -0.780951
C15 -2.876444 -0.669585 1.538898
H16 -2.991263 0.398008 1.741696
H17 -3.515666 -1.252227 2.204821
H18 -1.844187 -0.985760 1.682426
H19 4.059103 -0.552559 0.571989
C20 2.234931 -2.585662 -0.165860
H21 2.009493 -2.858444 -1.198041
H22 3.268034 -2.849754 0.057977
H23 1.571247 -3.125573 0.511184
H24 -4.283671 -0.792935 -0.017980
H25 -1.703396 2.037529 -0.544919

Zero-point correction= 0.200074 (Hartree/Particle)
Thermal correction to Energy= 0.212728
Thermal correction to Enthalpy= 0.213672
Thermal correction to Gibbs Free Energy= 0.160506
Sum of electronic and zero-point Energies= -676.373129
Sum of electronic and thermal Energies= -676.360475
Sum of electronic and thermal Enthalpies= -676.359531
Sum of electronic and thermal Free Energies= -676.412697

2-NHCH3[9MG – H_{N2} + H_{N3}]$^+$

C1 1.610680 0.663885 0.068459
C2 0.879485 -0.496683 -0.048373
C3 -1.167307 0.614812 -0.205500
C4 0.950782 1.947981 0.052389
C5 2.995496 -0.934294 0.134907
N6 -0.497222 1.767562 -0.095796
N7 -0.496729 -0.544757 -0.182154
O8 1.372255 3.065626 0.135122
N9 1.751981 -1.529890 -0.007434
N10 -2.501087 0.583997 -0.354969
H11 -3.041899 1.440078 -0.291231
N12 2.941678 0.366395 0.183013
N13 -3.133878 -0.660948 -0.383996
H14 -1.057676 -1.388127 -0.267606
C15 -4.053807 -0.856686 0.743071
H16 -4.832857 -0.086595 0.795483
H17 -4.528240 -1.830916 0.616755
H18 -3.486558 -0.861934 1.675770
H19 3.893803 -1.533147 0.194671
C20 1.457360 -2.952709 -0.102090
H21 0.989250 -3.182184 -1.062228
H22 2.396036 -3.501287 -0.034267
H23 0.812158 -3.265666 0.722232
H24 -3.608627 -0.756485 -1.277226
H25 -1.002766 2.646905 -0.121137

Zero-point correction= 0.205303 (Hartree/Particle)
Thermal correction to Energy= 0.218162
Thermal correction to Enthalpy= 0.219106
Thermal correction to Gibbs Free Energy= 0.165861
Sum of electronic and zero-point Energies= -676.482849
Sum of electronic and thermal Energies= -676.469990
Sum of electronic and thermal Enthalpies= -676.469046
Sum of electronic and thermal Free Energies= -676.522291

TS for 2-NHCH3[9MG – H_{N2} + H_{N3}]$^+$

C1 1.602701 0.578125 0.081770
C2 0.760154 -0.509747 -0.063679
C3 -1.097710 0.771481 -0.229856
C4 1.075401 1.924495 0.070831
C5 2.828549 -1.144243 0.135146
N6 -0.379308 1.887123 -0.105086
N7 -0.605202 -0.441579 -0.212074
O8 1.608038 2.994346 0.171469
N9 1.540582 -1.617579 -0.029820
N10 -2.480779 0.796646 -0.448477
H11 -3.033876 1.477546 0.061239
N12 2.898412 0.156972 0.204502
N13 -2.956210 -0.539672 -0.373016
H14 -1.798976 -1.018163 -0.331571
C15 -3.751558 -0.855519 0.833925
H16 -4.651016 -0.237731 0.878369
H17 -4.034360 -1.907482 0.782471
H18 -3.128938 -0.687415 1.714043
H19 3.666962 -1.824588 0.195260
C20 1.116276 -3.002508 -0.154241
H21 0.629689 -3.164165 -1.118454
H22 1.997948 -3.640098 -0.096066
H23 0.437375 -3.267709 0.659089
H24 -3.462312 -0.752664 -1.232327
H25 -0.812138 2.801817 -0.179378

Zero-point correction= 0.201841 (Hartree/Particle)
Thermal correction to Energy= 0.214016
Thermal correction to Enthalpy= 0.214961
Thermal correction to Gibbs Free Energy= 0.163348
Sum of electronic and zero-point Energies= -676.462424

Sum of electronic and thermal Energies= -676.450249
Sum of electronic and thermal Enthalpies= -676.449305
Sum of electronic and thermal Free Energies= -676.500918

2-NHCH$_3$[9MG – H$_{N2}$ + H$_{O6}$]$^+$

C1 -1.564261 -0.611678 0.062463
C2 -0.802181 0.566163 -0.062595
C3 1.150780 -0.486397 -0.212541
C4 -0.866632 -1.794801 0.037723
C5 -2.949798 0.965684 0.131082
N6 0.484663 -1.701197 -0.101720
N7 0.522608 0.666312 -0.190550
O8 -1.333910 -3.016922 0.127797
N9 -1.709209 1.570769 -0.016972
N10 2.490754 -0.574175 -0.366461
H11 2.953883 -1.457710 -0.173352
N12 -2.907987 -0.334793 0.182616
N13 3.252569 0.584601 -0.431602
C14 4.039908 0.826005 0.772334
H15 4.709749 -0.006292 1.033467
H16 4.642011 1.720260 0.603105
H17 3.364471 1.021739 1.608131
H18 -3.851643 1.560274 0.193142
C19 -1.430269 3.000645 -0.100305
H20 -0.364905 3.124118 -0.293408
H21 -2.001703 3.440434 -0.918591
H22 -1.688860 3.486576 0.841674
H23 3.812511 0.558198 -1.275598
H24 0.995862 -2.575675 -0.156239
H25 -2.299167 -3.010888 0.219145

Zero-point correction= 0.205020 (Hartree/Particle)
Thermal correction to Energy= 0.218102
Thermal correction to Enthalpy= 0.219046
Thermal correction to Gibbs Free Energy= 0.163141
Sum of electronic and zero-point Energies= -676.484656
Sum of electronic and thermal Energies= -676.471574
Sum of electronic and thermal Enthalpies= -676.470630
Sum of electronic and thermal Free Energies= -676.526535

TS for 2-NHCH$_3$[9MG – H$_{N2}$ + H$_{O6}$]$^+$

C1 -0.888826 1.100823 -0.201158
C2 -1.163330 -0.278890 -0.367182
C3 0.900214 -0.785255 -0.938168
C4 0.477101 1.391750 -0.440767
C5 -2.833139 0.791241 0.553486
N6 0.988414 0.512646 -1.409612
N7 -0.205938 -1.267065 -0.485559
O8 1.385411 1.971534 0.225454
N9 -2.412954 -0.450486 0.125484
N10 2.196268 -1.227666 -0.630548
H11 2.226184 -2.170419 -0.248658
N12 -1.931085 1.728422 0.415151
N13 2.741911 -0.251723 0.334740
C14 2.559465 -0.684048 1.742788
H15 3.048729 -1.643309 1.925966
H16 2.996725 0.078871 2.389193
H17 1.490460 -0.761250 1.947967
H18 -3.825451 0.938394 0.957249
C19 -3.139723 -1.711455 0.218459
H20 -2.439997 -2.503252 0.488632
H21 -3.902726 -1.620504 0.990718
H22 -3.608924 -1.946849 -0.738128
H23 3.736543 -0.180656 0.119200
H24 1.905594 0.770878 -1.756512
H25 2.182700 1.011940 0.268403

Zero-point correction= 0.202146 (Hartree/Particle)
Thermal correction to Energy= 0.213551
Thermal correction to Enthalpy= 0.214495
Thermal correction to Gibbs Free Energy= 0.164535
Sum of electronic and zero-point Energies= -676.385590
Sum of electronic and thermal Energies= -676.374185
Sum of electronic and thermal Enthalpies= -676.373241
Sum of electronic and thermal Free Energies= -676.423201

2-NHCH$_3$[9MG – H$_{N2}$ + H$_{N7}$]$^+$

C1 -1.530478 -0.664790 0.060794
C2 -0.807563 0.498728 -0.057946
C3 1.157481 -0.504336 -0.212558
C4 -0.863763 -1.939437 0.044405
C5 -2.948957 1.010800 0.124821
N6 0.523927 -1.727112 -0.101028
N7 0.514382 0.646397 -0.189178
O8 -1.349635 -3.045117 0.132306
N9 -1.725061 1.537853 -0.014276
N10 2.497812 -0.553541 -0.365847
H11 2.976865 -1.437845 -0.225688
N12 -2.858346 -0.314595 0.172130
N13 3.242081 0.617160 -0.418717
C14 4.063681 0.826531 0.768035
H15 4.749860 -0.006401 0.980652
H16 4.651286 1.733026 0.612479
H17 3.411990 0.983670 1.630387
H18 -3.864938 1.578090 0.187845
C19 -1.376324 2.955292 -0.107579
H20 -0.843651 3.127184 -1.042807
H21 -2.290368 3.546818 -0.081062
H22 -0.732290 3.219366 0.731240
H23 3.780747 0.621605 -1.277038
H24 1.062151 -2.585262 -0.147779
H25 -3.632589 -0.959173 0.271908

Zero-point correction= 0.205575 (Hartree/Particle)
Thermal correction to Energy= 0.218534
Thermal correction to Enthalpy= 0.219478
Thermal correction to Gibbs Free Energy= 0.165244
Sum of electronic and zero-point Energies= -676.496083
Sum of electronic and thermal Energies= -676.483124
Sum of electronic and thermal Enthalpies= -676.482180
Sum of electronic and thermal Free Energies= -676.536414

TS for 2-NHCH$_3$[9MG – H$_{N2}$ + H$_{N7}$]$^+$

C1 0.271954 1.191558 0.297488
C2 1.103312 0.585182 -0.612295
C3 -0.715762 -0.353136 -1.467288
C4 -1.085594 1.582424 -0.097579
C5 1.795082 0.114982 1.402784
N6 -1.478472 0.767186 -1.206851
N7 0.578001 -0.303213 -1.520634
O8 -1.847600 2.347230 0.428924
N9 2.194409 0.102041 0.135915
N10 -1.189908 -1.594636 -0.967879
H11 -2.197227 -1.709395 -1.058198
N12 0.556337 0.651997 1.559444
N13 -0.904979 -1.508675 0.523197
C14 -2.149190 -1.676149 1.306362
H15 -2.687192 -2.582712 1.016992
H16 -1.878060 -1.743286 2.361889
H17 -2.778602 -0.794681 1.167579
H18 2.383984 -0.290913 2.214857
C19 3.384377 -0.520217 -0.433257
H20 4.036263 0.249745 -0.846611
H21 3.907386 -1.071817 0.347042
H22 3.063200 -1.201025 -1.224573
H23 -0.307967 -2.321802 0.673319
H24 -2.485333 0.731069 -1.333883
H25 -0.188453 -0.393790 1.210558

Zero-point correction= 0.198860 (Hartree/Particle)

Thermal correction to Energy=	0.210654
Thermal correction to Enthalpy=	0.211599
Thermal correction to Gibbs Free Energy=	0.161132
Sum of electronic and zero-point Energies=	-676.301703
Sum of electronic and thermal Energies=	-676.289908
Sum of electronic and thermal Enthalpies=	-676.288964
Sum of electronic and thermal Free Energies=	-676.339430

3-$^{+}NH_2CH_3$[9MG – H_{N2}]

C1 0.999684 0.984003 0.150431
C2 0.649346 -0.321620 -0.090978
C3 -1.499994 0.289978 -0.790546
C4 -0.035907 2.031166 0.213103
C5 2.802991 -0.113429 0.018128
N6 -1.322600 1.495693 -0.113745
N7 -0.667446 -0.806857 -0.256602
O8 0.096656 3.186091 0.507289
N9 1.811790 -1.037001 -0.180178
N10 -2.348898 0.127104 -1.702214
H11 -2.378446 -0.833795 -2.037139
N12 2.350079 1.105543 0.214853
N13 -1.222734 -1.145874 1.085479
H14 -1.255434 -0.293117 1.665390
C15 -2.563682 -1.794127 1.023219
H16 -2.489761 -2.662097 0.369742
H17 -2.838413 -2.088886 2.036510
H18 -3.283578 -1.075819 0.633283
H19 3.847326 -0.394277 -0.002010
C20 1.968593 -2.458810 -0.458104
H21 1.185449 -2.779268 -1.146533
H22 2.937262 -2.619155 -0.930632
H23 1.924420 -3.044779 0.463550
H24 -0.544319 -1.782179 1.511089
H25 -2.005281 2.211421 -0.346232

Zero-point correction=	0.205838 (Hartree/Particle)
Thermal correction to Energy=	0.218443
Thermal correction to Enthalpy=	0.219387
Thermal correction to Gibbs Free Energy=	0.166793
Sum of electronic and zero-point Energies=	-676.417250
Sum of electronic and thermal Energies=	-676.404645
Sum of electronic and thermal Enthalpies=	-676.403700
Sum of electronic and thermal Free Energies=	-676.456295

TS for 3-$^{+}NH_2CH_3$[9MG – H_{N2}]

C1 -1.131349 0.914775 -0.143662
C2 -0.653368 -0.357513 0.211590
C3 1.422868 0.434058 0.816662
C4 -0.186159 2.055947 -0.190744
C5 -2.792785 -0.386022 -0.145719
N6 1.092093 1.650239 0.224065
N7 0.599450 -0.707383 0.507258
O8 -0.432453 3.177675 -0.544473
N9 -1.764883 -1.186604 0.184569
N10 2.439907 0.334573 1.561499
H11 2.570215 -0.620775 1.889239
N12 -2.445187 0.893299 -0.349653
N13 1.487694 -1.195567 -1.188261
H14 1.320206 -0.431848 -1.842847
C15 2.901506 -1.522877 -1.021049
H16 2.993146 -2.327093 -0.290025
H17 3.337536 -1.845567 -1.971605
H18 3.439080 -0.640731 -0.667985
H19 -3.806933 -0.756542 -0.222151
C20 -1.805527 -2.610892 0.501305
H21 -1.092408 -2.815225 1.300067
H22 -2.808817 -2.868224 0.839045
H23 -1.558397 -3.204178 -0.381486
H24 0.922419 -2.000258 -1.443175
H25 1.748951 2.404283 0.400070

Zero-point correction=	0.202094 (Hartree/Particle)
Thermal correction to Energy=	0.215072
Thermal correction to Enthalpy=	0.216017
Thermal correction to Gibbs Free Energy=	0.162016
Sum of electronic and zero-point Energies=	-676.408169
Sum of electronic and thermal Energies=	-676.395191
Sum of electronic and thermal Enthalpies=	-676.394247
Sum of electronic and thermal Free Energies=	-676.448248

3-$NHCH_3$[9MG – H_{N2} + H_{N1}]$^{+}$

C1 1.337532 0.467822 -0.114024
C2 -0.020104 0.631358 0.119039
C3 -0.343183 -1.556969 0.681689
C4 1.942234 -0.817531 -0.302789
C5 1.015063 2.548239 0.060165
N6 0.779711 -1.939346 -0.198715
N7 -0.998344 -0.368779 0.235489
O8 3.026392 -1.227881 -0.522326
N9 -0.225509 1.962819 0.228369
N10 -0.562933 -2.269191 1.679578
H11 -1.337274 -1.914392 2.241173
N12 1.970189 1.681516 -0.136211
N13 -1.625499 -0.698142 -1.043327
H14 0.412301 -2.105067 -1.145533
C15 -3.077851 -0.813684 -0.895603
H16 -3.539259 0.091102 -0.485031
H17 -3.490229 -1.020885 -1.884908
H18 -3.308794 -1.662191 -0.248996
H19 1.139156 3.621761 0.100168
C20 -1.495288 2.636637 0.476995
H21 -2.140716 1.966145 1.045627
H22 -1.308741 3.537544 1.060794
H23 -1.975758 2.905387 -0.465894
H24 -1.391350 0.046346 -1.696082
H25 1.215815 -2.800068 0.148173

Zero-point correction=	0.204183 (Hartree/Particle)
Thermal correction to Energy=	0.217094
Thermal correction to Enthalpy=	0.218038
Thermal correction to Gibbs Free Energy=	0.164403
Sum of electronic and zero-point Energies=	-676.404739
Sum of electronic and thermal Energies=	-676.391827
Sum of electronic and thermal Enthalpies=	-676.390883
Sum of electronic and thermal Free Energies=	-676.444519

TS for 3-$NHCH_3$[9MG – H_{N2} + H_{N1}]$^{+}$

C1 -0.802859 1.084129 -0.176235
C2 -0.673959 -0.268977 0.068144
C3 1.399134 0.012904 0.924664
C4 0.374723 1.936724 -0.265902
C5 -2.762199 0.300561 -0.084679
N6 1.613236 1.067946 -0.050561
N7 0.551131 -0.974748 0.249966
O8 0.518717 3.093453 -0.490377
N9 -1.930217 -0.775161 0.128435
N10 1.900139 -0.002375 2.061827
H11 1.675060 -0.853007 2.578353
N12 -2.117548 1.427132 -0.267213
N13 1.211513 -1.091571 -1.068436
H14 1.656138 0.210423 -0.967784
C15 2.229217 -2.156383 -1.071836
H16 1.788501 -3.117907 -0.802980
H17 2.657643 -2.200862 -2.074110
H18 3.018782 -1.901922 -0.362474
H19 -3.837642 0.187110 -0.088215
C20 -2.324089 -2.160021 0.369046
H21 -1.492484 -2.683062 0.841121
H22 -3.183043 -2.175007 1.039716
H23 -2.584437 -2.649518 -0.571219
H24 0.487202 -1.271498 -1.762149

H25 2.426959 1.655432 0.135437

Zero-point correction= 0.200653 (Hartree/Particle)
Thermal correction to Energy= 0.212753
Thermal correction to Enthalpy= 0.213697
Thermal correction to Gibbs Free Energy= 0.162164
Sum of electronic and zero-point Energies= -676.395430
Sum of electronic and thermal Energies= -676.383330
Sum of electronic and thermal Enthalpies= -676.382385
Sum of electronic and thermal Free Energies= -676.433919

3-$NHCH_3$[9MG]$^+$
C1 -0.608904 1.338718 -0.122597
C2 0.397237 0.424687 0.101700
C3 -1.099484 -1.385030 0.282790
C4 -1.993909 0.927039 -0.135332
C5 1.186596 2.445236 -0.042610
N6 -2.094510 -0.500498 0.136537
N7 0.186835 -0.946967 0.279485
O8 -3.004715 1.547683 -0.301028
N9 1.561095 1.126944 0.167327
N10 -1.329437 -2.685775 0.415216
H11 -2.262238 -3.067054 0.403390
N12 -0.092888 2.600858 -0.216130
N13 1.171303 -1.934610 0.162396
H14 -0.530023 -3.294621 0.537039
C15 1.708227 -2.068676 -1.196280
H16 2.095207 -1.130730 -1.610928
H17 2.510420 -2.806932 -1.157674
H18 0.924399 -2.446974 -1.855800
H19 1.927634 3.232669 -0.050776
C20 2.904962 0.700059 0.540823
H21 3.302855 -0.028862 -0.166858
H22 3.550449 1.577632 0.520278
H23 2.911685 0.297355 1.556439
H24 1.875875 -1.796698 0.877040
H25 -3.053669 -0.829123 0.171296

Zero-point correction= 0.205981 (Hartree/Particle)
Thermal correction to Energy= 0.218354
Thermal correction to Enthalpy= 0.219299
Thermal correction to Gibbs Free Energy= 0.168133
Sum of electronic and zero-point Energies= -676.478574
Sum of electronic and thermal Energies= -676.466201
Sum of electronic and thermal Enthalpies= -676.465257
Sum of electronic and thermal Free Energies= -676.516422

TS for 3-$NHCH_3$[9MG]$^+$
C1 -1.457198 0.436619 0.069931
C2 -0.092410 0.596760 0.016468
C3 0.260455 -1.813972 -0.110882
C4 -2.074411 -0.877099 -0.043814
C5 -1.110127 2.520514 0.079712
N6 -1.075676 -1.903240 -0.194911
N7 0.799735 -0.472959 -0.159909
O8 -3.234594 -1.175566 -0.041566
N9 0.143003 1.943553 -0.002300
N10 1.042297 -2.796401 0.178408
H11 1.775663 -3.140802 -0.427626
N12 -2.078251 1.649833 0.112574
N13 2.039216 -0.503223 0.542419
H14 2.012633 -1.569294 0.808583
C15 3.249039 -0.244893 -0.282731
H16 3.237944 0.771410 -0.667265
H17 4.119619 -0.405220 0.354953
H18 3.246556 -0.956594 -1.107183
H19 -1.224410 3.595260 0.120649
C20 1.387217 2.679039 -0.165454
H21 1.832814 2.467470 -1.140434
H22 1.159141 3.743572 -0.120081
H23 2.092967 2.460821 0.640125
H24 1.994747 0.084873 1.381440
H25 -1.438587 -2.851719 -0.223985

Zero-point correction= 0.201881 (Hartree/Particle)
Thermal correction to Energy= 0.214124
Thermal correction to Enthalpy= 0.215068
Thermal correction to Gibbs Free Energy= 0.163540
Sum of electronic and zero-point Energies= -676.406716
Sum of electronic and thermal Energies= -676.394473
Sum of electronic and thermal Enthalpies= -676.393529
Sum of electronic and thermal Free Energies= -676.445057

3-$NHCH_3$[9MG – H_{N2} + H_{O6}]$^+$
C1 -1.014506 0.979149 -0.085529
C2 0.277677 0.485963 0.113845
C3 -0.496789 -1.781437 0.278524
C4 -2.079005 0.077811 -0.093894
C5 0.240231 2.667759 -0.061304
N6 -1.786908 -1.218168 0.099762
N7 0.561975 -0.840030 0.290728
O8 -3.302495 0.492830 -0.271185
N9 1.086735 1.567737 0.136019
N10 -0.421074 -3.031059 0.391087
H11 0.538694 -3.342778 0.523172
N12 -1.005170 2.351421 -0.194554
N13 1.832961 -1.399401 0.226094
C14 2.416561 -1.428385 -1.112660
H15 2.490105 -0.442014 -1.589532
H16 3.413948 -1.862145 -1.026725
H17 1.815462 -2.081347 -1.749423
H18 0.648061 3.669025 -0.092125
C19 2.506319 1.680366 0.463361
H20 3.115431 1.075748 -0.208102
H21 2.792360 2.723977 0.337067
H22 2.680700 1.396824 1.503270
H23 2.426722 -1.048322 0.965313
H24 -2.506412 -1.934098 0.094574
H25 -3.977665 -0.198368 -0.246006

Zero-point correction= 0.205345 (Hartree/Particle)
Thermal correction to Energy= 0.217731
Thermal correction to Enthalpy= 0.218675
Thermal correction to Gibbs Free Energy= 0.167376
Sum of electronic and zero-point Energies= -676.449361
Sum of electronic and thermal Energies= -676.436975
Sum of electronic and thermal Enthalpies= -676.436031
Sum of electronic and thermal Free Energies= -676.487331

TS for 3-$NHCH_3$[9MG – H_{N2} + H_{O6}]$^+$
C1 -0.305944 1.260778 -0.377986
C2 -0.570438 -0.088924 -0.361437
C3 1.530215 -0.655129 -1.010801
C4 1.085279 1.425028 0.017309
C5 -2.400132 1.071000 -0.410889
N6 1.967191 0.675762 -0.694094
N7 0.440711 -1.063840 -0.122293
O8 1.410164 1.727447 1.217780
N9 -1.926277 -0.213728 -0.368652
N10 2.112242 -1.355122 -1.864104
H11 1.751066 -2.308916 -1.881998
N12 -1.450067 1.985672 -0.377310
N13 0.960262 -0.835796 1.288151
C14 -0.078919 -1.208455 2.266912
H15 -0.469149 -2.208547 2.065509
H16 0.379402 -1.178181 3.256626
H17 -0.881576 -0.469969 2.238370
H18 -3.458035 1.281514 -0.484475
C19 -2.707499 -1.447311 -0.351992
H20 -3.173907 -1.584776 0.625019

H21 -3.475222 -1.399622 -1.124412
H22 -2.040162 -2.282496 -0.562459
H23 1.728446 -1.502856 1.373816
H24 2.941261 0.751103 -0.411253
H25 1.372758 0.590607 1.504428

Zero-point correction= 0.200173 (Hartree/Particle)
Thermal correction to Energy= 0.211824
Thermal correction to Enthalpy= 0.212768
Thermal correction to Gibbs Free Energy= 0.162811
Sum of electronic and zero-point Energies= -676.344441
Sum of electronic and thermal Energies= -676.332790
Sum of electronic and thermal Enthalpies= -676.331846
Sum of electronic and thermal Free Energies= -676.381803

3-NHCH$_3$[9MG – H$_{N2}$ + H$_{N7}$]$^+$
C1 -0.952345 0.976136 -0.108183
C2 0.281039 0.440057 0.123025
C3 -0.661666 -1.769059 0.308135
C4 -2.181400 0.188296 -0.115897
C5 0.485017 2.630660 -0.039313
N6 -1.903163 -1.135200 0.141138
N7 0.484161 -0.896106 0.353842
O8 -3.286348 0.651280 -0.297892
N9 1.177294 1.492349 0.174058
N10 -0.619033 -3.022310 0.403002
H11 0.331722 -3.360630 0.532749
N12 -0.794863 2.335151 -0.201124
N13 1.704254 -1.524600 0.165480
C14 2.205697 -1.553531 -1.201060
H15 2.327633 -0.559396 -1.656627
H16 3.172795 -2.058598 -1.190729
H17 1.524284 -2.139203 -1.822744
H18 0.919613 3.617615 -0.073057
C19 2.604390 1.461844 0.512231
H20 3.151607 0.858465 -0.210551
H21 2.984166 2.482213 0.481877
H22 2.730059 1.066630 1.520935
H23 2.362453 -1.323084 0.903520
H24 -2.689097 -1.776861 0.142095
H25 -1.549380 2.991204 -0.372740

Zero-point correction= 0.205890 (Hartree/Particle)
Thermal correction to Energy= 0.218300
Thermal correction to Enthalpy= 0.219244
Thermal correction to Gibbs Free Energy= 0.167660
Sum of electronic and zero-point Energies= -676.462248
Sum of electronic and thermal Energies= -676.449837
Sum of electronic and thermal Enthalpies= -676.448893
Sum of electronic and thermal Free Energies= -676.500478

TS for 3-NHCH$_3$[9MG – H$_{N2}$ + H$_{N7}$]$^+$
C1 0.262889 1.159380 -0.332054
C2 0.718003 -0.060624 -0.746504
C3 -1.477147 -0.972918 -0.644189
C4 -1.196527 1.472940 -0.370071
C5 2.309232 0.956516 0.353389
N6 -1.942108 0.344100 -0.669193
N7 -0.099165 -1.133416 -0.198639
O8 -1.660894 2.570724 -0.197674
N9 2.068382 -0.114466 -0.387277
N10 -2.227859 -1.929191 -0.963077
H11 -1.749751 -2.824458 -0.889714
N12 1.174288 1.701104 0.560419
N13 0.056966 -0.794728 1.275789
H14 0.649445 0.741161 1.286790
C15 -1.149019 -0.983724 2.109738
H16 -1.589646 -1.972974 1.966279
H17 -0.824095 -0.876599 3.146344
H18 -1.886893 -0.211765 1.896634
H19 3.271637 1.196688 0.785335
C20 2.991242 -1.196184 -0.733492
H21 2.449676 -2.141372 -0.676468
H22 3.354735 -1.046171 -1.750601
H23 3.824188 -1.196857 -0.031396
H24 0.746312 -1.475492 1.589136
H25 -2.924030 0.475901 -0.887998

Zero-point correction= 0.198763 (Hartree/Particle)
Thermal correction to Energy= 0.210688
Thermal correction to Enthalpy= 0.211632
Thermal correction to Gibbs Free Energy= 0.161184
Sum of electronic and zero-point Energies= -676.305125
Sum of electronic and thermal Energies= -676.293200
Sum of electronic and thermal Enthalpies= -676.292255
Sum of electronic and thermal Free Energies= -676.342703

5-$^+$NH$_2$CH$_3$[9MG – H$_{N2}$]
C1 -0.148839 -0.791556 -0.156303
C2 -0.423800 0.693158 0.004498
C3 1.775428 1.221287 0.203269
C4 1.219950 -0.979953 -0.851273
C5 -2.167457 -0.384318 -0.811706
N6 0.424624 1.616115 0.224440
O7 1.494068 -1.960572 -1.495523
N8 -1.750571 0.838508 -0.306976
N9 2.742320 1.884843 0.670118
H10 2.419271 2.763252 1.072163
N11 -1.318338 -1.352717 -0.766000
N12 0.040547 -1.508349 1.182770
H13 0.111991 -2.502552 0.929992
C14 -1.042664 -1.327507 2.198286
H15 -1.083809 -0.276973 2.484052
H16 -1.982668 -1.653289 1.756816
H17 -0.800886 -1.943241 3.064184
C18 -2.420639 2.126696 -0.450625
H19 -3.478808 1.951261 -0.642662
H20 -2.310053 2.692520 0.474613
H21 -1.979543 2.691671 -1.275028
H22 -3.168460 -0.486565 -1.217804
N23 2.085363 0.011795 -0.480640
H24 0.943216 -1.239447 1.589081
H25 3.057724 -0.090260 -0.754079

Zero-point correction= 0.206132 (Hartree/Particle)
Thermal correction to Energy= 0.218791
Thermal correction to Enthalpy= 0.219735
Thermal correction to Gibbs Free Energy= 0.167022
Sum of electronic and zero-point Energies= -676.460756
Sum of electronic and thermal Energies= -676.448097
Sum of electronic and thermal Enthalpies= -676.447153
Sum of electronic and thermal Free Energies= -676.499866

5-NHCH$_3$[9MG – H$_{N2}$ + H$_{N1}$]$^+$
C1 -0.247962 -0.910562 -0.003626
C2 -0.430933 0.617563 -0.043291
C3 1.783689 1.137146 0.128249
C4 0.955905 -1.107372 -0.906575
C5 -2.289404 -0.455258 -0.591808
N6 0.474416 1.526395 0.069879
O7 1.117490 -1.662657 -1.936020
N8 -1.733909 0.808913 -0.316195
N9 2.833698 1.764736 0.386509
H10 2.651378 2.739978 0.619727
N11 -1.499833 -1.452734 -0.484180
N12 0.157669 -1.368613 1.316770
H13 0.127695 -2.386579 1.297106
C14 -0.677109 -0.887727 2.428478
H15 -0.525264 0.185554 2.567141
H16 -1.743878 -1.093696 2.282600

H17 -0.345320 -1.389716 3.337306
C18 -2.371751 2.108470 -0.495320
H19 -3.442453 1.954773 -0.625079
H20 -2.196908 2.722364 0.388859
H21 -1.960392 2.609355 -1.374169
H22 -3.332464 -0.523146 -0.882035
N23 2.091901 -0.333885 -0.257533
H24 2.302263 -0.809421 0.631007
H25 2.923226 -0.335463 -0.857688

Zero-point correction= 0.204188 (Hartree/Particle)
Thermal correction to Energy= 0.216974
Thermal correction to Enthalpy= 0.217918
Thermal correction to Gibbs Free Energy= 0.164894
Sum of electronic and zero-point Energies= -676.432572
Sum of electronic and thermal Energies= -676.419787
Sum of electronic and thermal Enthalpies= -676.418843
Sum of electronic and thermal Free Energies= -676.471866

TS for 5-NHCH$_3$[9MG – H$_{N2}$ + H$_{N1}$]$^+$
C1 -0.230448 -0.907023 -0.108693
C2 -0.473809 0.609665 -0.002430
C3 1.737392 1.145678 0.142757
C4 0.998404 -0.939372 -1.028408
C5 -2.306208 -0.511443 -0.545635
N6 0.403884 1.533845 0.143324
O7 1.178056 -1.351865 -2.121933
N8 -1.804336 0.751818 -0.211128
N9 2.765160 1.834434 0.340512
H10 2.534591 2.809003 0.529421
N11 -1.470354 -1.483712 -0.527758
N12 0.311576 -1.452220 1.164880
H13 0.302871 -2.470733 1.077452
C14 -0.407918 -1.068031 2.398069
H15 -0.287348 0.002318 2.571454
H16 -1.468606 -1.322984 2.328802
H17 0.040011 -1.607774 3.231932
C18 -2.508320 2.026724 -0.302706
H19 -3.575921 1.847514 -0.177696
H20 -2.156306 2.682739 0.493411
H21 -2.319518 2.497076 -1.270218
H22 -3.355383 -0.616967 -0.800209
N23 2.004238 -0.300717 -0.152123
H24 1.561840 -0.914183 0.824674
H25 2.973586 -0.412416 -0.455224

Zero-point correction= 0.200785 (Hartree/Particle)
Thermal correction to Energy= 0.212815
Thermal correction to Enthalpy= 0.213759
Thermal correction to Gibbs Free Energy= 0.162846
Sum of electronic and zero-point Energies= -676.426642
Sum of electronic and thermal Energies= -676.414612
Sum of electronic and thermal Enthalpies= -676.413668
Sum of electronic and thermal Free Energies= -676.464581

5-NHCH$_3$[9MG]$^+$
C1 -0.085711 -0.793920 0.014785
C2 -0.739918 0.567605 0.149659
C3 1.205499 1.644891 0.087432
C4 1.130034 -0.599816 -0.901995
C5 -2.205458 -0.973521 -0.448477
N6 -0.109380 1.692332 0.363848
O7 1.576594 -1.379821 -1.689013
N8 -2.040426 0.386422 -0.040744
N9 1.950310 2.683646 0.429017
H10 1.500678 3.459495 0.894980
N11 -1.140536 -1.664445 -0.492349
N12 0.452912 -1.185525 1.304679
H13 -0.285380 -1.131270 2.000333
C14 1.077182 -2.518327 1.341688
H15 1.996088 -2.517183 0.752054
H16 1.345659 -2.720811 2.379048
H17 0.419829 -3.310663 0.971030
H18 -3.200530 -1.326297 -0.697485
C19 -3.086506 1.402361 -0.011329
H20 -2.646627 2.335480 0.338604
H21 -3.495675 1.543110 -1.013673
H22 -3.876885 1.090267 0.672846
N23 1.776692 0.627041 -0.621870
H24 2.947775 2.718333 0.282353
H25 2.684577 0.746174 -1.056649

Zero-point correction= 0.204303 (Hartree/Particle)
Thermal correction to Energy= 0.217274
Thermal correction to Enthalpy= 0.218219
Thermal correction to Gibbs Free Energy= 0.165152
Sum of electronic and zero-point Energies= -676.494266
Sum of electronic and thermal Energies= -676.481295
Sum of electronic and thermal Enthalpies= -676.480351
Sum of electronic and thermal Free Energies= -676.533418

TS for 5-NHCH$_3$[9MG]$^+$
C1 -0.114145 -0.623123 -0.072106
C2 0.927523 0.503284 -0.054679
C3 -0.752329 1.769030 0.144526
C4 -0.975567 -0.343328 1.191215
C5 1.868968 -1.491539 -0.125657
N6 0.628131 1.746215 0.110038
O7 -1.378063 -1.136071 1.984280
N8 2.127461 -0.110457 -0.121852
N9 -1.499891 2.093325 -0.880033
H10 -0.998402 2.657629 -1.564654
N11 0.638693 -1.846167 -0.105012
N12 -1.074356 -0.470909 -1.223327
H13 -0.522945 -0.581068 -2.075782
C14 -2.144459 -1.505234 -1.234582
H15 -2.846962 -1.313766 -0.422805
H16 -2.674082 -1.422172 -2.183451
H17 -1.718214 -2.503615 -1.124043
H18 2.702869 -2.185083 -0.150284
C19 3.425893 0.546268 -0.017563
H20 3.414673 1.453665 -0.622503
H21 3.638829 0.803462 1.022304
H22 4.192189 -0.129440 -0.396893
N23 -1.349666 1.023638 1.143261
H24 -1.567847 0.880582 -1.214090
H25 -2.303509 1.246714 1.409499

Zero-point correction= 0.200363 (Hartree/Particle)
Thermal correction to Energy= 0.212244
Thermal correction to Enthalpy= 0.213189
Thermal correction to Gibbs Free Energy= 0.162804
Sum of electronic and zero-point Energies= -676.419802
Sum of electronic and thermal Energies= -676.407920
Sum of electronic and thermal Enthalpies= -676.406976
Sum of electronic and thermal Free Energies= -676.457361

5-NHCH$_3$[9MG – H$_{N2}$ + H$_{N3}$]$^+$
C1 -0.019633 -0.784788 -0.060495
C2 0.837510 0.455454 -0.155249
C3 -1.122062 1.812201 -0.043234
C4 -1.147764 -0.471089 0.954300
C5 2.068856 -1.301971 0.338558
N6 0.286773 1.640183 -0.320311
O7 -1.580998 -1.252706 1.752893
N8 2.095269 0.108793 0.006090
N9 -1.804483 2.815837 -0.360854
H10 -1.315637 3.524546 -0.900502
N11 0.917409 -1.821464 0.359642
N12 -0.642773 -1.013712 -1.343519

H13 0.077365 -1.220430 -2.029743
C14 -1.669853 -2.070006 -1.365719
H15 -2.571011 -1.720791 -0.858177
H16 -1.925040 -2.249595 -2.410213
H17 -1.334849 -3.004202 -0.904409
H18 3.011059 -1.796750 0.546741
C19 3.268420 0.978224 0.011586
H20 3.400490 1.437961 -0.970077
H21 3.164599 1.744297 0.784446
H22 4.146505 0.373072 0.234158
N23 -1.651557 0.802073 0.754645
H24 -2.540326 1.023265 1.190713
H25 0.837523 2.480159 -0.454412

Zero-point correction= 0.203936 (Hartree/Particle)
Thermal correction to Energy= 0.216694
Thermal correction to Enthalpy= 0.217638
Thermal correction to Gibbs Free Energy= 0.165264
Sum of electronic and zero-point Energies= -676.459065
Sum of electronic and thermal Energies= -676.446307
Sum of electronic and thermal Enthalpies= -676.445363
Sum of electronic and thermal Free Energies= -676.497737

TS for 5-NHCH$_3$[9MG – H$_{N2}$ + H$_{N3}$]$^+$
C1 -0.056109 -0.856880 0.082377
C2 -0.797164 0.431195 0.233892
C3 1.075611 1.724216 -0.067067
C4 0.982424 -0.577193 -1.054151
C5 -2.150010 -1.220255 -0.276893
N6 -0.072846 1.425378 0.764114
O7 1.324156 -1.406665 -1.850921
N8 -2.058406 0.200703 -0.014994
N9 1.750246 2.781307 -0.021454
H10 1.369990 3.447742 0.646703
N11 -1.049119 -1.857358 -0.209486
N12 0.660474 -0.919406 1.376226
H13 0.034867 -1.340579 2.066498
C14 1.965871 -1.623597 1.422745
H15 2.692419 -1.080292 0.819017
H16 2.300831 -1.625364 2.459715
H17 1.868331 -2.647473 1.057470
H18 -3.120040 -1.648209 -0.504424
C19 -3.172131 1.149008 -0.001662
H20 -2.788726 2.112661 0.332502
H21 -3.585408 1.237141 -1.007460
H22 -3.937372 0.793192 0.689397
N23 1.450034 0.713190 -0.983905
H24 0.520298 0.473334 1.461253
H25 2.197935 0.965495 -1.622255

Zero-point correction= 0.199788 (Hartree/Particle)
Thermal correction to Energy= 0.211937
Thermal correction to Enthalpy= 0.212881
Thermal correction to Gibbs Free Energy= 0.161688
Sum of electronic and zero-point Energies= -676.403937
Sum of electronic and thermal Energies= -676.391788
Sum of electronic and thermal Enthalpies= -676.390843
Sum of electronic and thermal Free Energies= -676.442036

5-NHCH$_3$[9MG – H$_{N2}$ + H$_{O6}$]$^+$
C1 -0.066845 -0.713564 -0.005761
C2 0.820695 0.521167 -0.134198
C3 -0.916804 1.953024 -0.188108
C4 -1.295522 -0.271393 0.762143
C5 1.955980 -1.229694 0.584486
N6 0.441239 1.729484 -0.329071
N7 2.080421 0.069985 0.071417
N8 -1.607901 2.947912 -0.525050
H9 -1.048021 3.635615 -1.027026
N10 0.772822 -1.714550 0.634729
N11 -0.587108 -1.079354 -1.310944
H12 0.098901 -0.880626 -2.031387
C13 -1.111727 -2.445951 -1.440172
H14 -1.928951 -2.601364 -0.732980
H15 -1.518348 -2.545705 -2.447145
H16 -0.354310 -3.215709 -1.267513
H17 2.846927 -1.754090 0.914067
C18 3.273927 0.906822 0.077603
H19 3.383057 1.392226 -0.892781
H20 3.197092 1.668428 0.856970
H21 4.141602 0.274282 0.263215
N22 -1.670481 0.965962 0.592210
H23 -2.564757 1.325138 0.918766
O24 -1.933175 -1.152402 1.461868
H25 -2.748958 -0.837522 1.881874

Zero-point correction= 0.204161 (Hartree/Particle)
Thermal correction to Energy= 0.216899
Thermal correction to Enthalpy= 0.217844
Thermal correction to Gibbs Free Energy= 0.165434
Sum of electronic and zero-point Energies= -676.444373
Sum of electronic and thermal Energies= -676.431635
Sum of electronic and thermal Enthalpies= -676.430691
Sum of electronic and thermal Free Energies= -676.483101

TS for 5-NHCH$_3$[9MG – H$_{N2}$ + H$_{O6}$]$^+$
C1 0.424939 -0.563731 0.131312
C2 0.030292 0.848749 -0.257081
C3 -2.234018 0.478467 -0.202264
C4 -0.816558 -1.358680 0.514767
C5 1.824256 0.858833 0.975280
N6 -1.143437 1.308483 -0.455757
N7 1.140282 1.610898 0.021727
N8 -3.444057 0.707033 -0.456460
H9 -3.560145 1.591980 -0.947532
N10 1.437171 -0.354885 1.156992
N11 0.994751 -1.548920 -0.837437
H12 0.472846 -1.516600 -1.714551
C13 2.444625 -1.520200 -1.122736
H14 2.994379 -1.761262 -0.216263
H15 2.635742 -2.285562 -1.875454
H16 2.761026 -0.547932 -1.509493
H17 2.637992 1.313963 1.530591
C18 1.140395 3.070297 -0.040466
H19 0.895573 3.381095 -1.055649
H20 0.404293 3.485342 0.653269
H21 2.138414 3.428889 0.210701
N22 -1.991446 -0.771226 0.493885
H23 -2.830580 -1.324109 0.656613
O24 -0.596037 -2.595873 0.606836
H25 0.382326 -2.540908 -0.040458

Zero-point correction= 0.201461 (Hartree/Particle)
Thermal correction to Energy= 0.213202
Thermal correction to Enthalpy= 0.214146
Thermal correction to Gibbs Free Energy= 0.163974
Sum of electronic and zero-point Energies= -676.433240
Sum of electronic and thermal Energies= -676.421499
Sum of electronic and thermal Enthalpies= -676.420555
Sum of electronic and thermal Free Energies= -676.470727

5-NHCH$_3$[9MG – H$_{N2}$ + H$_{N7}$]$^+$
C1 0.153172 -0.704582 0.066768
C2 -0.802840 0.461698 0.165751
C3 0.893523 1.955956 0.070617
C4 1.284380 -0.287747 -0.891636
C5 -2.002120 -1.291881 -0.455234
N6 -0.492435 1.679828 0.244869
O7 1.808942 -1.070042 -1.650254
N8 -2.106339 -0.045918 0.012750

N9 1.468142 3.013720 0.439394
H10 0.817119 3.647220 0.899792
N11 -0.764996 -1.718956 -0.523593
N12 0.702323 -1.028760 1.335690
H13 -0.004302 -1.103178 2.058125
C14 1.702966 -2.096585 1.409441
H15 2.586382 -1.825092 0.829685
H16 2.003383 -2.197118 2.452308
H17 1.333926 -3.068256 1.056498
H18 -2.865549 -1.875736 -0.753830
C19 -3.314386 0.777890 0.081924
H20 -3.372988 1.236229 1.068895
H21 -3.263651 1.556783 -0.680353
H22 -4.184808 0.143730 -0.082684
N23 1.620967 1.017100 -0.699286
H24 -0.477576 -2.622986 -0.876387
H25 2.474721 1.361152 -1.124581

Zero-point correction=	0.204275 (Hartree/Particle)
Thermal correction to Energy=	0.217091
Thermal correction to Enthalpy=	0.218035
Thermal correction to Gibbs Free Energy=	0.165469
Sum of electronic and zero-point Energies=	-676.473363
Sum of electronic and thermal Energies=	-676.460547
Sum of electronic and thermal Enthalpies=	-676.459603
Sum of electronic and thermal Free Energies=	-676.512170

TS for 5-NHCH$_3$[9MG – H$_{N2}$ + H$_{N7}$]$^+$

C1 -0.058836 -0.733797 0.054394
C2 0.820979 0.474450 -0.141999
C3 -0.920872 1.920776 -0.071457
C4 -1.253625 -0.334073 0.934912
C5 2.077930 -1.282817 0.363189
N6 0.466915 1.685711 -0.225497
O7 -1.810786 -1.102566 1.677660
N8 2.133143 0.013368 -0.011193
N9 -1.527159 2.960169 -0.446982
H10 -0.888013 3.621860 -0.884105
N11 0.868346 -1.772408 0.514696
N12 -0.504422 -1.517411 -1.146403
H13 -0.013323 -1.204882 -1.983736
C14 -1.944259 -1.714432 -1.433693
H15 -2.437370 -0.763757 -1.650056
H16 -2.011404 -2.369184 -2.302256
H17 -2.417557 -2.200124 -0.581723
H18 2.978738 -1.874111 0.485686
C19 3.308931 0.878587 -0.079594
H20 3.316387 1.394437 -1.039956
H21 3.269981 1.612759 0.727013
H22 4.203911 0.264733 0.015624
N23 -1.643850 0.946978 0.659528
H24 0.273610 -2.393324 -0.429915
H25 -2.520967 1.265103 1.058586

Zero-point correction=	0.200229 (Hartree/Particle)
Thermal correction to Energy=	0.212544
Thermal correction to Enthalpy=	0.213488
Thermal correction to Gibbs Free Energy=	0.161853
Sum of electronic and zero-point Energies=	-676.422040
Sum of electronic and thermal Energies=	-676.409725
Sum of electronic and thermal Enthalpies=	-676.408781
Sum of electronic and thermal Free Energies=	-676.460416

6-$^+$NH$_2$CH$_3$[9MG – H$_{N2}$]

C1 0.153066 -0.381412 -0.140913
C2 -1.251388 -0.222446 0.027698
C3 -1.213961 2.057212 0.078776
C4 0.869332 0.757738 -0.237747
C5 -0.636045 -2.350882 -0.142881
N6 0.235505 1.936934 -0.133205
N7 -1.930505 0.866958 0.128145
O8 2.226180 0.813477 -0.460973
N9 -1.716186 -1.529578 0.027697
N10 -1.784748 3.178860 0.206164
H11 -1.162237 3.980380 0.151343
N12 0.509280 -1.724079 -0.250724
H13 -0.748604 -3.426290 -0.181630
C14 -3.118137 -1.906276 0.155473
H15 -3.676684 -1.541590 -0.708035
H16 -3.190269 -2.992113 0.213342
H17 -3.529386 -1.460337 1.061931
H18 0.769584 2.793471 -0.198517
H19 2.059010 -1.243936 -0.441470
N20 2.822406 -0.472343 -0.360375
C21 3.550989 -0.610216 0.923556
H22 4.041211 -1.585428 0.909951
H23 4.281459 0.192660 1.008367
H24 2.819144 -0.569721 1.729490
H25 3.453928 -0.514496 -1.164673

Zero-point correction=	0.203975 (Hartree/Particle)
Thermal correction to Energy=	0.216701
Thermal correction to Enthalpy=	0.217645
Thermal correction to Gibbs Free Energy=	0.164105
Sum of electronic and zero-point Energies=	-676.391031
Sum of electronic and thermal Energies=	-676.378305
Sum of electronic and thermal Enthalpies=	-676.377361
Sum of electronic and thermal Free Energies=	-676.430901

TS for 6-$^+$NH$_2$CH$_3$[9MG – H$_{N2}$]

C1 0.028710 -0.497194 -0.524423
C2 -1.240115 0.022096 -0.069237
C3 -0.536178 2.196308 0.071386
C4 1.052227 0.413411 -0.640041
C5 -1.234102 -2.164241 -0.270282
N6 0.767479 1.712958 -0.277634
N7 -1.543265 1.228699 0.228327
O8 2.266575 0.074289 -0.976866
N9 -2.032921 -1.114459 0.045101
N10 -0.827908 3.415037 0.260818
H11 -0.040900 4.049807 0.144781
N12 0.013692 -1.857898 -0.597865
H13 -1.613859 -3.178643 -0.247013
C14 -3.437173 -1.124120 0.438562
H15 -4.030128 -0.591918 -0.306442
H16 -3.774628 -2.157680 0.510772
H17 -3.545491 -0.630888 1.405323
H18 1.464662 2.409927 -0.503910
H19 2.442495 -1.916554 -0.216583
N20 2.938127 -1.089042 0.126123
C21 2.698405 -0.764897 1.517311
H22 2.863011 -1.656805 2.130496
H23 3.355016 0.045009 1.829352
H24 1.647713 -0.472537 1.629514
H25 3.902440 -1.049278 -0.195111

Zero-point correction=	0.201112 (Hartree/Particle)
Thermal correction to Energy=	0.214246
Thermal correction to Enthalpy=	0.215190
Thermal correction to Gibbs Free Energy=	0.160158
Sum of electronic and zero-point Energies=	-676.366854
Sum of electronic and thermal Energies=	-676.353720
Sum of electronic and thermal Enthalpies=	-676.352776
Sum of electronic and thermal Free Energies=	-676.407808

6-NHCH$_3$[9MG – H$_{N2}$ + H$_{N1}$]$^+$

C1 0.199778 -0.440366 -0.052884
C2 -1.253880 -0.264803 -0.034009
C3 -1.298237 1.990666 0.084363

C4 0.969843 0.658259 -0.131423
C5 -0.638048 -2.383663 -0.012744
N6 0.260653 1.941995 -0.167824
N7 -1.969681 0.824463 -0.002065
O8 2.261328 0.884564 -0.210939
N9 -1.739748 -1.526496 -0.034083
N10 -1.632119 3.177087 0.311755
N11 0.511974 -1.800724 -0.018628
H12 -0.784558 -3.456496 0.013639
C13 -3.151417 -1.887000 -0.013233
H14 -3.632670 -1.558568 -0.935752
H15 -3.232885 -2.969706 0.076830
H16 -3.637709 -1.411641 0.840025
H17 0.420874 2.380018 -1.083645
N18 3.041306 -0.301734 -0.546556
C19 3.584753 -0.864535 0.687369
H20 4.293118 -1.632681 0.366957
H21 4.102332 -0.128483 1.311285
H22 2.784590 -1.357411 1.241042
H23 3.790150 0.138256 -1.078691
H24 -2.635680 3.270812 0.455985
H25 0.658361 2.595899 0.517013

Zero-point correction= 0.204029 (Hartree/Particle)
Thermal correction to Energy= 0.217048
Thermal correction to Enthalpy= 0.217992
Thermal correction to Gibbs Free Energy= 0.163619
Sum of electronic and zero-point Energies= -676.371975
Sum of electronic and thermal Energies= -676.358955
Sum of electronic and thermal Enthalpies= -676.358011
Sum of electronic and thermal Free Energies= -676.412385

TS for 6-NHCH$_3$[9MG – H$_{N2}$ + H$_{N1}$]$^+$
C1 0.402716 -0.855458 -0.254885
C2 1.361737 0.250395 -0.083123
C3 -0.175747 1.922149 -0.057050
C4 -0.847003 -0.478653 -0.516257
C5 2.278655 -1.748616 0.094914
N6 -1.149807 0.918025 -0.672499
N7 1.136777 1.516330 0.021651
O8 -1.981494 -1.237977 -0.610867
N9 2.553687 -0.387384 0.095675
N10 -0.575713 3.063231 0.285311
H11 -1.578477 3.202940 0.190011
N12 1.038735 -2.078541 -0.089530
H13 3.079337 -2.464123 0.240633
C14 3.821482 0.269628 0.384739
H15 3.764884 0.780473 1.347934
H16 4.606604 -0.485374 0.411223
H17 4.041832 0.997411 -0.396935
H18 -1.205941 1.144465 -1.672398
H19 -2.349106 0.686719 -0.290142
N20 -3.061827 -0.425892 -0.140108
C21 -3.450185 -0.835377 1.219289
H22 -4.324583 -0.242658 1.496128
H23 -3.687134 -1.899709 1.244571
H24 -2.628459 -0.614943 1.901539
H25 -3.809901 -0.586168 -0.814892

Zero-point correction= 0.200173 (Hartree/Particle)
Thermal correction to Energy= 0.212429
Thermal correction to Enthalpy= 0.213373
Thermal correction to Gibbs Free Energy= 0.161136
Sum of electronic and zero-point Energies= -676.352820
Sum of electronic and thermal Energies= -676.340565
Sum of electronic and thermal Enthalpies= -676.339620
Sum of electronic and thermal Free Energies= -676.391857

6-NHCH$_3$[9MG]$^+$
C1 0.221987 -0.471277 -0.102756
C2 -1.186318 -0.331464 -0.022676
C3 -1.182264 1.892746 0.052294
C4 0.931186 0.713921 -0.111934
C5 -0.565193 -2.428355 -0.061757
N6 0.194437 1.866816 -0.026355
N7 -1.903871 0.793044 0.048420
O8 2.219548 0.957146 -0.158871
N9 -1.671188 -1.592264 -0.008162
N10 -1.779953 3.091303 0.131844
H11 -1.283343 3.966487 0.142249
N12 0.574306 -1.808017 -0.115676
H13 -0.685957 -3.503655 -0.052302
C14 -3.076204 -1.974565 0.052164
H15 -3.580514 -1.693055 -0.873471
H16 -3.136331 -3.054057 0.186616
H17 -3.554667 -1.476859 0.896400
H18 0.722828 2.731715 -0.045533
N19 3.012031 -0.199785 -0.547380
C20 3.612845 -0.770403 0.654292
H21 4.323705 -1.519979 0.297593
H22 4.136944 -0.033861 1.272550
H23 2.843100 -1.286840 1.229456
H24 3.728263 0.263833 -1.103108
H25 -2.786981 3.107819 0.183931

Zero-point correction= 0.199177 (Hartree/Particle)
Thermal correction to Energy= 0.211147
Thermal correction to Enthalpy= 0.212091
Thermal correction to Gibbs Free Energy= 0.160509
Sum of electronic and zero-point Energies= -676.293193
Sum of electronic and thermal Energies= -676.281223
Sum of electronic and thermal Enthalpies= -676.280279
Sum of electronic and thermal Free Energies= -676.331861

TS for 6-NHCH$_3$[9MG]$^+$
C1 0.454440 -0.684251 0.658269
C2 1.361144 0.412046 0.178847
C3 -0.401021 1.665807 0.151580
C4 -0.766698 -0.251504 1.029940
C5 2.219753 -1.596434 -0.068691
N6 -0.787419 1.159564 1.409017
N7 0.931050 1.589761 -0.193484
O8 -1.958063 -0.828104 0.730874
N9 2.498953 -0.223450 -0.171077
N10 -1.365906 1.790703 -0.738799
H11 -2.218454 2.024352 -0.224849
N12 1.041848 -1.909188 0.363690
H13 2.978733 -2.317574 -0.349175
C14 3.643920 0.394535 -0.831524
H15 4.086291 1.141432 -0.172040
H16 4.381658 -0.377301 -1.048407
H17 3.319553 0.871616 -1.759155
H18 -0.108189 1.336796 2.146494
H19 -1.670868 0.589083 -1.020384
N20 -2.168056 -0.667757 -0.709811
C21 -3.612358 -0.772493 -0.945242
H22 -3.762057 -0.725127 -2.026302
H23 -4.002047 -1.711700 -0.549822
H24 -4.108565 0.070853 -0.465237
H25 -1.679912 -1.461272 -1.133938

Zero-point correction= 0.200173 (Hartree/Particle)
Thermal correction to Energy= 0.212429
Thermal correction to Enthalpy= 0.213373
Thermal correction to Gibbs Free Energy= 0.161136
Sum of electronic and zero-point Energies= -676.352820
Sum of electronic and thermal Energies= -676.340565
Sum of electronic and thermal Enthalpies= -676.339620
Sum of electronic and thermal Free Energies= -676.391857

6-NHCH$_3$[9MG – H$_{N2}$ + H$_{N7}$]$^+$

C1 0.130666 -0.430438 -0.130647
C2 -1.254180 -0.157142 0.012537
C3 -1.075011 2.104366 0.054349
C4 0.956162 0.668074 -0.186631
C5 -0.934608 -2.358337 -0.088313
N6 0.355191 1.867664 -0.095691
N7 -1.869899 0.965186 0.102797
O8 2.274675 0.712640 -0.318024
N9 -1.874395 -1.419864 0.035989
N10 -1.575697 3.262112 0.135860
H11 -0.898460 4.018702 0.087070
N12 0.268880 -1.815855 -0.189404
H13 -1.129602 -3.421061 -0.104840
C14 -3.318062 -1.604782 0.163591
H15 -3.811608 -1.143869 -0.692635
H16 -3.537894 -2.671027 0.201042
H17 -3.655779 -1.115697 1.077611
H18 0.956559 2.681767 -0.133002
N19 2.865422 -0.603967 -0.373558
C20 3.824551 -0.686225 0.730883
H21 4.363043 -1.628663 0.602772
H22 4.537705 0.143077 0.734929
H23 3.284300 -0.716585 1.678342
H24 3.376346 -0.556253 -1.254386
H25 1.154356 -2.291547 -0.299281

Zero-point correction=	0.204677 (Hartree/Particle)
Thermal correction to Energy=	0.217435
Thermal correction to Enthalpy=	0.218379
Thermal correction to Gibbs Free Energy=	0.165090
Sum of electronic and zero-point Energies=	-676.419842
Sum of electronic and thermal Energies=	-676.407084
Sum of electronic and thermal Enthalpies=	-676.406139
Sum of electronic and thermal Free Energies=	-676.459428

TS for 6-NHCH$_3$[9MG – H$_{N2}$ + H$_{N7}$]$^+$

C1 0.154675 -0.326684 -0.089090
C2 -1.249736 -0.265221 0.030989
C3 -1.335682 2.010516 0.071418
C4 0.832113 0.836848 -0.188237
C5 -0.491088 -2.359029 -0.110030
N6 0.129015 1.975192 -0.115347
N7 -1.994045 0.783301 0.102794
O8 2.179422 0.911586 -0.371091
N9 -1.621839 -1.609913 0.023329
N10 -1.966259 3.099697 0.188682
H11 -1.387159 3.933943 0.151635
N12 0.604004 -1.636029 -0.183098
H13 -0.519939 -3.439287 -0.148196
C14 -2.998669 -2.083208 0.115270
H15 -3.553626 -1.767025 -0.769282
H16 -2.997097 -3.170550 0.186265
H17 -3.463914 -1.654589 1.003594
H18 0.610035 2.862569 -0.186170
H19 1.891381 -1.273295 -0.343044
N20 2.771637 -0.397333 -0.373857
C21 3.681675 -0.513146 0.781735
H22 4.187361 -1.476858 0.691128
H23 4.406729 0.300353 0.774825
H24 3.084600 -0.494439 1.693428
H25 3.288932 -0.418381 -1.255304

Zero-point correction=	0.199896 (Hartree/Particle)
Thermal correction to Energy=	0.212298
Thermal correction to Enthalpy=	0.213242
Thermal correction to Gibbs Free Energy=	0.160180
Sum of electronic and zero-point Energies=	-676.393413
Sum of electronic and thermal Energies=	-676.381011
Sum of electronic and thermal Enthalpies=	-676.380067
Sum of electronic and thermal Free Energies=	-676.433130

7-NHCH$_3$[9MG – H$_{N2}$ + H$_{O6}$]$^+$

C1 0.207765 -0.309239 -0.119993
C2 -0.601987 0.834731 -0.014683
C3 -2.547188 -0.314507 0.136892
C4 -0.416341 -1.545140 -0.139164
C5 1.539203 1.438257 -0.218414
N6 -1.740358 -1.509398 -0.011872
N7 -1.885047 0.912250 0.104480
O8 0.170406 -2.714353 -0.268473
N9 0.295062 1.915249 -0.081380
N10 -3.785118 -0.530414 0.275172
H11 -4.297247 0.342671 0.368525
N12 1.523882 0.108300 -0.254610
N13 2.577694 -0.810066 -0.353104
H14 3.069967 -0.643551 -1.226795
C15 3.476930 -0.787050 0.811432
H16 2.903311 -1.034269 1.706159
H17 4.227916 -1.562198 0.653355
H18 3.980628 0.175648 0.950662
H19 2.429777 2.046308 -0.289583
C20 -0.119478 3.315486 -0.018794
H21 -0.798481 3.521094 -0.847007
H22 -0.638612 3.488011 0.924497
H23 0.761528 3.952541 -0.086163
H24 -2.268606 -2.376593 -0.007724
H25 1.130335 -2.591509 -0.372612

Zero-point correction=	0.204982 (Hartree/Particle)
Thermal correction to Energy=	0.217776
Thermal correction to Enthalpy=	0.218720
Thermal correction to Gibbs Free Energy=	0.165572
Sum of electronic and zero-point Energies=	-676.448931
Sum of electronic and thermal Energies=	-676.436137
Sum of electronic and thermal Enthalpies=	-676.435193
Sum of electronic and thermal Free Energies=	-676.488341

TS for 7-NHCH$_3$[9MG – H$_{N2}$ + H$_{O6}$]$^+$

C1 0.224618 -0.335369 -0.325411
C2 -0.478347 0.835072 -0.007471
C3 -2.466386 -0.208036 0.227363
C4 -0.475904 -1.590172 -0.420204
C5 1.617915 1.332081 -0.523840
N6 -1.803675 -1.420168 -0.117106
N7 -1.729369 1.001786 0.189136
O8 0.083789 -2.654835 -0.660658
N9 0.492013 1.891180 -0.171316
N10 -3.691831 -0.288022 0.535318
H11 -4.071372 0.632179 0.744938
N12 1.460972 -0.020894 -0.767678
N13 2.703713 -0.981063 0.022520
H14 3.609426 -0.728290 -0.370276
C15 2.602944 -0.964441 1.480952
H16 1.538704 -0.865302 1.732744
H17 2.962552 -1.910608 1.891089
H18 3.173060 -0.129250 1.885754
H19 2.558601 1.857694 -0.629729
C20 0.183970 3.309946 -0.004419
H21 -0.552425 3.594147 -0.757466
H22 -0.245944 3.452692 0.987125
H23 1.096614 3.893126 -0.118578
H24 -2.395508 -2.243021 -0.097751
H25 2.380369 -1.868907 -0.380521

Zero-point correction=	0.202512 (Hartree/Particle)
Thermal correction to Energy=	0.215253
Thermal correction to Enthalpy=	0.216197
Thermal correction to Gibbs Free Energy=	0.163096
Sum of electronic and zero-point Energies=	-676.382102

Sum of electronic and thermal Energies= -676.369361
Sum of electronic and thermal Enthalpies= -676.368416
Sum of electronic and thermal Free Energies= -676.421517

8-$^+NH_2CH_3$[9MG – H_{N2}]
C1 -0.373094 -0.855932 0.206120
C2 -0.236213 0.605164 0.055676
C3 -2.476011 0.932121 -0.203525
C4 -1.737678 -1.455804 0.057725
C5 1.737281 -0.395125 0.502821
N6 -2.670050 -0.467567 -0.123453
N7 -1.166763 1.453394 -0.127659
O8 -1.962704 -2.641316 0.097826
N9 1.115371 0.849829 0.184204
N10 -3.508074 1.646381 -0.355195
H11 -3.257817 2.631886 -0.407597
N12 0.733860 -1.442954 0.451998
N13 2.772434 -0.811477 -0.542743
H14 2.322546 -0.737968 -1.461325
C15 4.056843 -0.049303 -0.533289
H16 4.517168 -0.152876 0.449132
H17 4.713975 -0.463249 -1.297358
H18 3.848447 0.996978 -0.748997
H19 2.269979 -0.391570 1.461492
C20 1.649650 2.169431 0.514369
H21 0.828353 2.795367 0.868255
H22 2.396409 2.081045 1.307681
H23 2.093028 2.648937 -0.360959
H24 2.944563 -1.811999 -0.389998
H25 -3.637127 -0.762579 -0.212372

Zero-point correction= 0.206378 (Hartree/Particle)
Thermal correction to Energy= 0.219060
Thermal correction to Enthalpy= 0.220004
Thermal correction to Gibbs Free Energy= 0.167013
Sum of electronic and zero-point Energies= -676.455929
Sum of electronic and thermal Energies= -676.443247
Sum of electronic and thermal Enthalpies= -676.442303
Sum of electronic and thermal Free Energies= -676.495294

8-$NHCH_3$[9MG – H_{N2} + H_{N1}]$^+$
C1 -0.169605 0.781449 -0.153934
C2 -0.271947 -0.698321 -0.055703
C3 -2.550338 -0.785789 0.100313
C4 -1.391926 1.603249 -0.042844
C5 1.890186 0.018738 -0.368161
N6 -2.535162 0.733477 0.468943
N7 -1.353456 -1.418184 0.059890
O8 -1.591825 2.744668 -0.279651
N9 0.981653 -1.119531 -0.134612
N10 -3.733624 -1.170251 -0.049191
H11 -3.789717 -2.164920 -0.262733
N12 1.014188 1.210769 -0.330323
N13 2.966966 0.031379 0.546380
H14 -2.550536 0.796725 1.495309
C15 4.117324 0.854967 0.169020
H16 3.874046 1.916675 0.046939
H17 4.879451 0.752012 0.941850
H18 4.537090 0.475101 -0.766268
H19 2.289488 -0.025771 -1.391598
C20 1.424125 -2.504288 -0.143427
H21 0.698180 -3.107255 0.401831
H22 1.503715 -2.872445 -1.169917
H23 2.398253 -2.552325 0.344266
H24 2.654976 0.212499 1.494827
H25 -3.431218 1.118723 0.143517

Zero-point correction= 0.203587 (Hartree/Particle)
Thermal correction to Energy= 0.216660
Thermal correction to Enthalpy= 0.217604
Thermal correction to Gibbs Free Energy= 0.163668
Sum of electronic and zero-point Energies= -676.424536
Sum of electronic and thermal Energies= -676.411464
Sum of electronic and thermal Enthalpies= -676.410519
Sum of electronic and thermal Free Energies= -676.464455

TS for 8-$NHCH_3$[9MG – H_{N2} + H_{N1}]$^+$
C1 0.416170 -1.008148 -0.836462
C2 -0.211700 0.367224 -0.957537
C3 1.489466 1.348798 0.098506
C4 1.770781 -1.031895 -0.214184
C5 -1.595839 -1.073090 -0.158626
N6 1.502395 0.023973 0.819828
N7 0.352833 1.489540 -0.691302
O8 2.747377 -1.689386 -0.289599
N9 -1.541070 0.094933 -1.016705
N10 2.395490 2.153907 0.411630
H11 2.312183 3.039686 -0.085932
N12 -0.431660 -1.900834 -0.506391
N13 -1.126770 -0.694905 1.262331
H14 0.262888 -0.265819 1.209865
C15 -1.988870 0.268381 1.984930
H16 -3.043110 -0.015760 1.932389
H17 -1.679516 0.288523 3.030554
H18 -1.852606 1.263198 1.562287
H19 -2.560189 -1.579488 -0.155920
C20 -2.567838 1.128869 -1.093412
H21 -2.686173 1.425685 -2.136188
H22 -3.513608 0.717380 -0.740703
H23 -2.293465 2.007881 -0.503296
H24 -1.104981 -1.572446 1.786707
H25 2.248013 0.039110 1.521989

Zero-point correction= 0.200178 (Hartree/Particle)
Thermal correction to Energy= 0.211940
Thermal correction to Enthalpy= 0.212884
Thermal correction to Gibbs Free Energy= 0.162650
Sum of electronic and zero-point Energies= -676.349422
Sum of electronic and thermal Energies= -676.337661
Sum of electronic and thermal Enthalpies= -676.336717
Sum of electronic and thermal Free Energies= -676.386950

8-$NHCH_3$[9MG]$^+$
C1 -0.283925 0.900585 -0.130945
C2 -0.199136 -0.576352 -0.100888
C3 -2.421212 -0.824584 0.095289
C4 -1.631603 1.524496 -0.027305
C5 1.864085 0.432052 -0.344062
N6 -2.632124 0.533292 0.079792
N7 -1.224434 -1.408920 0.010096
O8 -1.894512 2.692589 -0.028603
N9 1.072622 -0.861997 -0.200912
N10 -3.485411 -1.611082 0.202390
H11 -3.334512 -2.609921 0.210924
N12 0.842799 1.477215 -0.240843
N13 2.929963 0.609806 0.529391
H14 2.698575 0.705033 1.507200
C15 4.268759 0.141884 0.204645
H16 4.491486 0.380468 -0.838965
H17 4.986637 0.682442 0.822862
H18 4.417959 -0.933945 0.359142
H19 2.230493 0.447831 -1.380210
C20 1.679437 -2.180365 -0.191213
H21 0.893391 -2.929278 -0.097354
H22 2.229205 -2.340125 -1.121425
H23 2.364769 -2.255701 0.655540
H24 -3.574637 0.902630 0.144146
H25 -4.429610 -1.264641 0.274439

Zero-point correction= 0.203719 (Hartree/Particle)

Thermal correction to Energy= 0.217069
Thermal correction to Enthalpy= 0.218013
Thermal correction to Gibbs Free Energy= 0.163933
Sum of electronic and zero-point Energies= -676.499022
Sum of electronic and thermal Energies= -676.485673
Sum of electronic and thermal Enthalpies= -676.484728
Sum of electronic and thermal Free Energies= -676.538808

TS for 8-$NHCH_3$[9MG]$^+$
C1 -0.910060 -0.998638 -0.054940
C2 -0.035330 -0.427275 1.018555
C3 -0.949949 1.397652 0.579396
C4 -2.310569 -0.468156 -0.140604
C5 1.152057 -1.126386 -0.622410
N6 -2.149610 0.933084 0.118600
N7 -0.235153 0.730024 1.568074
O8 -3.319295 -0.982253 -0.501971
N9 1.163995 -1.052444 0.836965
N10 -0.262340 2.264261 -0.156513
H11 0.470657 2.684697 0.419071
N12 -0.219691 -1.478832 -1.010293
N13 1.269962 0.278988 -1.256338
H14 0.913070 0.154781 -2.207819
C15 2.656270 0.798355 -1.365195
H16 3.320954 0.067334 -1.831907
H17 2.634353 1.699427 -1.979864
H18 3.034887 1.058221 -0.377939
H19 1.919429 -1.789430 -1.022830
C20 2.338397 -0.659782 1.606582
H21 2.257990 -1.096418 2.602428
H22 3.229304 -1.064400 1.125920
H23 2.411376 0.428640 1.706534
H24 -2.689244 1.560338 -0.473136
H25 0.436549 1.334647 -0.776448

Zero-point correction= 0.199925 (Hartree/Particle)
Thermal correction to Energy= 0.211452
Thermal correction to Enthalpy= 0.212396
Thermal correction to Gibbs Free Energy= 0.163008
Sum of electronic and zero-point Energies= -676.352488
Sum of electronic and thermal Energies= -676.340961
Sum of electronic and thermal Enthalpies= -676.340017
Sum of electronic and thermal Free Energies= -676.389405

8-$NHCH_3$[9MG – H_{N2} + H_{N3}]$^+$
C1 -0.112089 0.759568 -0.249112
C2 -0.230908 -0.704222 -0.141255
C3 -2.616043 -0.539007 0.225614
C4 -1.338851 1.608348 -0.129763
C5 1.910327 -0.063782 -0.489651
N6 -2.471816 0.839569 0.093012
N7 -1.401103 -1.290179 0.090261
O8 -1.347499 2.805958 -0.216273
N9 0.958655 -1.207627 -0.317583
N10 -3.750291 -1.033018 0.446423
H11 -3.789668 -2.044399 0.528434
N12 1.089750 1.124477 -0.438527
N13 2.872892 -0.069902 0.571143
H14 3.465214 -0.890250 0.480844
C15 3.721727 1.133699 0.624514
H16 3.117527 1.994887 0.908064
H17 4.474502 0.970503 1.395755
H18 4.224746 1.349060 -0.326874
H19 2.320014 -0.132835 -1.514311
C20 1.374410 -2.600918 -0.255754
H21 0.561684 -3.252671 -0.581741
H22 2.212175 -2.747239 -0.938962
H23 1.676116 -2.856635 0.763188
H24 -3.344744 1.349683 0.188981
H25 -1.475629 -2.293133 0.206134

Zero-point correction= 0.203975 (Hartree/Particle)
Thermal correction to Energy= 0.217022
Thermal correction to Enthalpy= 0.217966
Thermal correction to Gibbs Free Energy= 0.163901
Sum of electronic and zero-point Energies= -676.463494
Sum of electronic and thermal Energies= -676.450447
Sum of electronic and thermal Enthalpies= -676.449503
Sum of electronic and thermal Free Energies= -676.503568

TS for 8-$NHCH_3$[9MG – H_{N2} + H_{N3}]$^+$
C1 0.402266 -1.000097 -0.580594
C2 -0.220614 0.297538 -0.924286
C3 1.505155 1.374632 0.242587
C4 1.890974 -1.021301 -0.409933
C5 -1.734282 -0.921731 -0.115806
N6 2.304282 0.269985 -0.099260
N7 0.134046 1.341295 -0.200836
O8 2.613828 -1.972559 -0.513820
N9 -1.516358 0.058072 -1.207135
N10 2.005639 2.333308 0.886242
H11 1.355219 3.107985 0.999421
N12 -0.496833 -1.727338 -0.045037
N13 -1.730485 -0.039314 1.095626
H14 -2.652760 0.386803 1.172322
C15 -1.400454 -0.676254 2.396913
H16 -0.369866 -1.026601 2.387918
H17 -1.516459 0.080480 3.173050
H18 -2.062324 -1.520100 2.603859
H19 -2.637466 -1.519212 -0.235468
C20 -2.488318 1.122597 -1.459519
H21 -2.177479 1.654963 -2.358458
H22 -3.454341 0.654493 -1.651834
H23 -2.575585 1.843107 -0.639944
H24 -0.741629 1.029635 0.645441
H25 3.291678 0.384555 0.110882

Zero-point correction= 0.200216 (Hartree/Particle)
Thermal correction to Energy= 0.212072
Thermal correction to Enthalpy= 0.213016
Thermal correction to Gibbs Free Energy= 0.162521
Sum of electronic and zero-point Energies= -676.365654
Sum of electronic and thermal Energies= -676.353797
Sum of electronic and thermal Enthalpies= -676.352853
Sum of electronic and thermal Free Energies= -676.403349

8-$NHCH_3$[9MG – H_{N2} + H_{O6}]$^+$
C1 -0.164037 0.704167 -0.208598
C2 -0.269060 -0.764156 -0.108088
C3 -2.519398 -0.745831 0.187062
C4 -1.411301 1.460459 -0.080145
C5 1.878350 -0.038768 -0.429485
N6 -2.477927 0.732853 0.102317
N7 -1.353231 -1.452563 0.077322
N8 0.982518 -1.188353 -0.267538
N9 -3.702484 -1.146914 0.362890
H10 -3.742751 -2.162812 0.423936
N11 1.011171 1.148499 -0.385859
N12 2.831906 0.040877 0.637406
H13 3.251696 -0.869903 0.789680
C14 3.866830 1.058532 0.431621
H15 3.412594 2.050058 0.458215
H16 4.581846 0.990892 1.251685
H17 4.404156 0.939949 -0.520155
H18 2.319985 -0.060181 -1.444527
C19 1.415757 -2.574421 -0.254744
H20 0.552682 -3.207357 -0.463552
H21 2.170550 -2.726188 -1.029140
H22 1.823790 -2.843892 0.723531
H23 -3.409091 1.134265 0.206286

O24 -1.370928 2.751659 -0.155956
H25 -2.224550 3.201216 -0.059816

Zero-point correction= 0.203961 (Hartree/Particle)
Thermal correction to Energy= 0.216792
Thermal correction to Enthalpy= 0.217736
Thermal correction to Gibbs Free Energy= 0.164723
Sum of electronic and zero-point Energies= -676.442260
Sum of electronic and thermal Energies= -676.429429
Sum of electronic and thermal Enthalpies= -676.428485
Sum of electronic and thermal Free Energies= -676.481498

TS for 8-NHCH$_3$[9MG – H$_{N2}$ + H$_{O6}$]$^+$
C1 -0.148801 -0.397456 0.908716
C2 -0.501745 0.869990 0.236545
C3 -2.607563 0.058326 -0.076257
C4 -0.761468 -1.467777 0.123883
C5 1.708115 0.567267 0.564763
N6 -2.036680 -1.296693 -0.176654
N7 -1.691340 1.128877 -0.167514
N8 0.689025 1.505352 0.130684
N9 -3.860968 0.122513 -0.089394
H10 -4.176809 1.090633 -0.138732
N11 1.037388 -0.467480 1.364950
N12 2.089589 -0.401624 -0.551745
H13 2.067313 0.095240 -1.443104
C14 3.422399 -1.021724 -0.353812
H15 3.419925 -1.557388 0.595941
H16 3.595898 -1.733438 -1.161329
H17 4.214264 -0.268888 -0.354730
H18 2.579334 1.044140 1.014253
C19 0.940563 2.792047 -0.500996
H20 -0.018139 3.294487 -0.630777
H21 1.581151 3.402716 0.138069
H22 1.407437 2.668832 -1.483105
O23 0.103807 -2.149295 -0.516116
H24 1.090785 -1.473625 -0.547306
H25 -2.489722 -1.909008 -0.849585

Zero-point correction= 0.200468 (Hartree/Particle)
Thermal correction to Energy= 0.212059
Thermal correction to Enthalpy= 0.213003
Thermal correction to Gibbs Free Energy= 0.163317
Sum of electronic and zero-point Energies= -676.375444
Sum of electronic and thermal Energies= -676.363853
Sum of electronic and thermal Enthalpies= -676.362909
Sum of electronic and thermal Free Energies= -676.412595

8-NHCH$_3$[9MG – H$_{N2}$ + H$_{N7}$]$^+$
C1 -0.391665 0.803727 -0.136139
C2 -0.273901 -0.654899 -0.090982
C3 -2.546963 -0.877370 0.114545
C4 -1.718264 1.477793 -0.018620
C5 1.858687 0.290042 -0.349113
N6 -2.693507 0.542516 0.089532
N7 -1.275172 -1.451286 0.023352
O8 -1.822152 2.684313 -0.033244
N9 1.046894 -0.922768 -0.177771
N10 -3.624848 -1.529217 0.225144
H11 -3.442498 -2.531584 0.235258
N12 0.764554 1.328977 -0.276772
N13 2.868117 0.549023 0.577759
H14 2.631675 0.307114 1.532079
C15 4.264420 0.304679 0.209011
H16 4.489277 0.817944 -0.728607
H17 4.903300 0.735553 0.979763
H18 4.505400 -0.759037 0.103105
H19 2.274732 0.336930 -1.364162
C20 1.638366 -2.250146 -0.215768
H21 0.848500 -2.977147 -0.027197
H22 2.080948 -2.444999 -1.196501
H23 2.402442 -2.341472 0.559390
H24 0.950860 2.331521 -0.297654
H25 -3.653765 0.866992 0.164174

Zero-point correction= 0.204029 (Hartree/Particle)
Thermal correction to Energy= 0.217144
Thermal correction to Enthalpy= 0.218088
Thermal correction to Gibbs Free Energy= 0.164161
Sum of electronic and zero-point Energies= -676.458147
Sum of electronic and thermal Energies= -676.445032
Sum of electronic and thermal Enthalpies= -676.444088
Sum of electronic and thermal Free Energies= -676.498015

TS for 8-NHCH$_3$[9MG – H$_{N2}$ + H$_{N7}$]$^+$
C1 -0.264016 -0.749708 0.254329
C2 -0.333250 0.711739 0.075087
C3 -2.607398 0.666568 -0.129350
C4 -1.492149 -1.574196 0.037374
C5 1.823180 0.065787 0.451499
N6 -2.575118 -0.752936 -0.084617
N7 -1.399916 1.385519 -0.113813
O8 -1.482613 -2.781836 0.006770
N9 0.970316 1.148256 0.069095
N10 -3.750904 1.198969 -0.210009
H11 -3.675724 2.213766 -0.254288
N12 0.936723 -1.112929 0.521288
N13 2.727296 -0.569522 -0.538672
H14 2.492602 -0.275260 -1.486673
C15 4.180350 -0.544160 -0.285403
H16 4.380491 -0.982836 0.692984
H17 4.670311 -1.152601 -1.044837
H18 4.573020 0.475340 -0.323085
H19 2.361742 0.239012 1.389788
C20 1.357523 2.551353 0.146522
H21 0.568163 3.142105 -0.318523
H22 1.480494 2.869745 1.186241
H23 2.287278 2.707733 -0.402616
H24 1.863360 -1.632361 -0.124335
H25 -3.485071 -1.195754 -0.172077

Zero-point correction= 0.199670 (Hartree/Particle)
Thermal correction to Energy= 0.212370
Thermal correction to Enthalpy= 0.213314
Thermal correction to Gibbs Free Energy= 0.160135
Sum of electronic and zero-point Energies= -676.409480
Sum of electronic and thermal Energies= -676.396780
Sum of electronic and thermal Enthalpies= -676.395836
Sum of electronic and thermal Free Energies= -676.449015

Cartesian coordinates for structures in Table S5, optimized at ωB97XD/6-31+G(d,p)

9MG···$^{+}CH_2NH_2$
C1 -0.224932 0.195163 -0.001165
C2 1.105110 0.605584 0.001426
C3 1.957131 -1.444073 -0.000562
C4 -0.493278 -1.193913 -0.000849
C5 -0.262522 2.314790 0.005196
N6 2.214096 -0.157152 -0.002902
N7 1.066955 1.968029 0.006753
N8 2.972703 -2.330968 0.021649
H9 2.841032 -3.320037 -0.104525
H10 3.908828 -1.961700 -0.031882
N11 -1.071505 1.286245 0.001664
N12 -3.944090 -0.693878 -0.001992
H13 -4.741131 -1.324214 -0.003333
C14 -4.117120 0.567601 -0.002379
H15 -3.216993 1.190596 -0.000844
H16 -5.120945 0.984266 -0.004253
C17 2.220484 2.855757 -0.008024
H18 1.876906 3.878245 0.147217
H19 2.905915 2.576487 0.792835
H20 2.735859 2.783582 -0.967223
H21 -0.569149 3.351940 0.008881
N22 0.675264 -1.953849 -0.000043
H23 0.524827 -2.955266 0.020442
O24 -1.587216 -1.789890 0.000662
H25 -2.950620 -1.119218 -0.000371

Zero-point correction= 0.202498 (Hartree/Particle)
Thermal correction to Energy= 0.216449
Thermal correction to Enthalpy= 0.217393
Thermal correction to Gibbs Free Energy= 0.161507
Sum of electronic and zero-point Energies= -676.541457
Sum of electronic and thermal Energies= -676.527506
Sum of electronic and thermal Enthalpies= -676.526562
Sum of electronic and thermal Free Energies= -676.582447

TS for 9MG···$^{+}CH_2NH_2$
C1 1.116841 0.984937 0.111213
C2 0.875555 -0.367934 -0.259512
C3 -1.240211 -0.002021 -0.911706
C4 0.020762 1.962031 -0.016186
C5 2.911684 -0.074779 0.433076
N6 -1.111392 1.327156 -0.552003
N7 -0.241579 -0.902293 -0.686861
O8 0.041519 3.130312 0.272313
N9 2.077603 -1.019815 -0.024779
N10 -2.374928 -0.370660 -1.456532
H11 -2.306861 -1.348697 -1.745207
N12 2.361611 1.150097 0.534796
N13 -2.041563 -1.074350 1.462383
H14 -1.749501 -0.356330 2.110184
C15 -3.326538 -1.007377 0.924677
H16 -3.242486 -0.652956 -0.215122
H17 -3.835241 -1.970711 0.872292
H18 -3.946543 -0.225380 1.366847
H19 3.940225 -0.292766 0.691487
C20 2.363297 -2.428173 -0.282176
H21 2.227688 -2.639242 -1.343421
H22 3.394518 -2.630785 0.004083
H23 1.690659 -3.051906 0.307153
H24 -1.635966 -1.981884 1.634308
H25 -1.885234 1.937106 -0.796452

Zero-point correction= 0.198283 (Hartree/Particle)
Thermal correction to Energy= 0.211307
Thermal correction to Enthalpy= 0.212251
Thermal correction to Gibbs Free Energy= 0.158220
Sum of electronic and zero-point Energies= -676.409820
Sum of electronic and thermal Energies= -676.396797
Sum of electronic and thermal Enthalpies= -676.395853
Sum of electronic and thermal Free Energies= -676.449884

9MG
N1 1.925385 -0.404816 -0.008124
C2 0.252461 1.014390 -0.009946
C3 -1.132501 1.402457 0.000726
O4 -1.656530 2.499300 0.012475
N5 -1.958940 0.237145 0.007479
H6 -2.946246 0.443596 0.085896
C7 -1.531930 -1.063025 0.000528
N8 -2.503066 -2.031267 0.060083
H9 -3.394781 -1.839190 -0.369916
H10 -2.151002 -2.963855 -0.097079
N11 -0.273502 -1.410691 -0.013098
C12 0.563018 -0.338508 -0.005505
C13 2.721295 -1.614596 0.019539
H14 2.616062 -2.119852 0.982531
H15 2.396306 -2.291177 -0.773002
H16 3.767687 -1.350260 -0.138383
C17 2.365674 0.902242 -0.009137
N18 1.397783 1.777511 -0.011571
H19 3.422490 1.133410 -0.010459

Zero-point correction= 0.146855 (Hartree/Particle)
Thermal correction to Energy= 0.156717
Thermal correction to Enthalpy= 0.157661
Thermal correction to Gibbs Free Energy= 0.111719
Sum of electronic and zero-point Energies= -581.568634
Sum of electronic and thermal Energies= -581.558773
Sum of electronic and thermal Enthalpies= -581.557829
Sum of electronic and thermal Free Energies= -581.603770

$^{+}CH_2NH_2$
N1 -0.600479 -0.000003 0.000000
H2 -1.136509 -0.866579 0.000000
C3 0.675301 0.000000 0.000000
H4 1.212262 0.944830 0.000000
H5 1.212306 -0.944804 0.000001
H6 -1.136514 0.866576 0.000001

Zero-point correction= 0.054899 (Hartree/Particle)
Thermal correction to Energy= 0.057879
Thermal correction to Enthalpy= 0.058823
Thermal correction to Gibbs Free Energy= 0.032692
Sum of electronic and zero-point Energies= -94.896747
Sum of electronic and thermal Energies= -94.893767
Sum of electronic and thermal Enthalpies= -94.892823
Sum of electronic and thermal Free Energies= -94.918954

2-CH_2NH_2[9MG]$^{+}$
C1 1.162714 0.958047 -0.123995
C2 0.788295 -0.428019 0.135795
C3 -1.482706 0.004224 0.333306
C4 0.097975 1.999327 -0.046636
C5 2.923122 -0.214812 -0.234881
N6 -0.356554 -0.923038 0.345410
O7 0.287545 3.182018 -0.189841
N8 2.019915 -1.129338 0.043890
N9 -2.358952 -0.185565 1.438482
H10 -1.895783 -0.125220 2.338178
H11 -2.855919 -1.066997 1.334454
N12 2.437184 1.070822 -0.352814
H13 -3.027311 0.436357 -1.076856
H14 -2.408726 -2.401087 -1.268287
H15 -3.875469 -1.631187 -1.217473
C16 2.196711 -2.566403 0.249654

H17 3.237584 -2.821071 0.055181
H18 1.936051 -2.812959 1.279406
H19 1.543702 -3.106177 -0.436139
H20 3.973666 -0.449178 -0.364305
N21 -1.093977 1.411614 0.298736
H22 -1.857766 2.054902 0.479225
C23 -2.278134 -0.353641 -0.990080
H24 -1.578757 -0.254848 -1.830597
N25 -2.924686 -1.626070 -0.875567

Zero-point correction=	0.203556 (Hartree/Particle)
Thermal correction to Energy=	0.216478
Thermal correction to Enthalpy=	0.217422
Thermal correction to Gibbs Free Energy=	0.164497
Sum of electronic and zero-point Energies=	-676.439936
Sum of electronic and thermal Energies=	-676.427013
Sum of electronic and thermal Enthalpies=	-676.426069
Sum of electronic and thermal Free Energies=	-676.478995

TS for 2-CH_2NH_2[9MG]$^+$

C1 1.128559 0.962996 -0.116232
C2 0.794498 -0.397780 0.204806
C3 -1.429154 -0.002129 0.462251
C4 0.066238 1.990210 -0.038446
C5 2.900155 -0.172352 -0.284635
N6 -0.351952 -0.886307 0.538381
O7 0.195704 3.172449 -0.233533
N8 2.003588 -1.090833 0.069318
N9 -2.510537 -0.192557 1.365155
H10 -2.235373 -0.011379 2.325540
H11 -2.871670 -1.138174 1.292421
N12 2.403880 1.083067 -0.418605
H13 -2.754579 0.389435 -1.261956
H14 -2.346342 -2.528797 -1.276085
H15 -3.800793 -1.649544 -1.021194
C16 2.214204 -2.513016 0.325616
H17 3.256283 -2.752439 0.117900
H18 1.986370 -2.729969 1.369722
H19 1.564539 -3.099299 -0.324451
H20 3.943376 -0.414991 -0.446462
N21 -1.121472 1.387940 0.358569
H22 -1.908168 2.017653 0.481209
C23 -2.100840 -0.468119 -1.108551
H24 -1.227534 -0.443718 -1.764487
N25 -2.796893 -1.646631 -1.097494

Zero-point correction=	0.202189 (Hartree/Particle)
Thermal correction to Energy=	0.214918
Thermal correction to Enthalpy=	0.215862
Thermal correction to Gibbs Free Energy=	0.163677
Sum of electronic and zero-point Energies=	-676.438556
Sum of electronic and thermal Energies=	-676.425826
Sum of electronic and thermal Enthalpies=	-676.424882
Sum of electronic and thermal Free Energies=	-676.477067

3-CH_2NH_2[9MG]$^+$

C1 -1.544014 -0.189426 0.079385
C2 -0.369881 0.533759 0.063995
C3 0.970021 -1.384460 -0.057792
C4 -1.526451 -1.631990 0.016841
C5 -2.113860 1.837345 -0.032082
N6 0.909378 -0.032996 0.085015
N7 -0.728900 1.847360 -0.023246
N8 2.164287 -1.972499 -0.088020
H9 2.244333 -2.976862 -0.132666
H10 2.964741 -1.381883 -0.325376
N11 -2.625503 0.643426 0.023612
N12 3.194004 0.597476 -0.306783
H13 3.064192 1.191941 -1.119361
C14 2.093635 0.672483 0.628057
H15 2.389917 0.170467 1.554333
H16 1.766065 1.678636 0.887632
C17 0.075096 3.051673 -0.193237
H18 -0.576715 3.834831 -0.579792
H19 0.497131 3.391829 0.755119
H20 0.864812 2.882629 -0.928011
H21 -2.669943 2.763284 -0.087181
N22 -0.159054 -2.102187 -0.126032
H23 -0.096220 -3.101859 -0.287664
O24 -2.413097 -2.438105 0.017597
H25 4.069698 0.851457 0.139362

Zero-point correction=	0.206856 (Hartree/Particle)
Thermal correction to Energy=	0.219105
Thermal correction to Enthalpy=	0.220049
Thermal correction to Gibbs Free Energy=	0.168759
Sum of electronic and zero-point Energies=	-676.514073
Sum of electronic and thermal Energies=	-676.501823
Sum of electronic and thermal Enthalpies=	-676.500879
Sum of electronic and thermal Free Energies=	-676.552169

TS for 3-CH_2NH_2[9MG]$^+$

C1 -1.611714 0.091399 0.054038
C2 -0.315568 0.557893 0.038447
C3 0.622328 -1.520546 -0.037434
C4 -1.858113 -1.333110 0.012068
C5 -1.766114 2.192996 -0.024216
N6 0.808290 -0.219772 0.064690
N7 -0.403196 1.924460 -0.008900
N8 1.691727 -2.365406 -0.026466
H9 1.545833 -3.359660 -0.126024
H10 2.548454 -2.019269 -0.460544
N11 -2.512534 1.120743 0.010585
N12 3.455222 0.151276 -0.323266
H13 3.552724 0.753005 -1.160186
C14 2.583073 0.397897 0.639339
H15 2.576653 -0.235677 1.528378
H16 2.156327 1.416717 0.694916
C17 0.630584 2.938038 -0.150680
H18 0.151252 3.885133 -0.429082
H19 1.158358 3.100141 0.789215
H20 1.315503 2.673734 -0.957272
H21 -2.134175 3.209320 -0.075922
N22 -0.611765 -2.054734 -0.096213
H23 -0.741683 -3.052049 -0.218002
O24 -2.886034 -1.956169 0.009949
H25 4.257970 -0.565416 -0.197446

Zero-point correction=	0.203776 (Hartree/Particle)
Thermal correction to Energy=	0.215665
Thermal correction to Enthalpy=	0.216610
Thermal correction to Gibbs Free Energy=	0.165473
Sum of electronic and zero-point Energies=	-676.477726
Sum of electronic and thermal Energies=	-676.465837
Sum of electronic and thermal Enthalpies=	-676.464892
Sum of electronic and thermal Free Energies=	-676.516029

4-CH_2NH_2[9MG]$^+$

C1 0.100182 1.106205 -0.191344
C2 0.625069 -0.234152 0.180766
C3 -1.368696 -1.092404 -0.617795
C4 -1.381097 1.317192 -0.129077
C5 2.125900 1.135167 -0.785126
N6 -0.104277 -1.302042 -0.419274
N7 1.990732 -0.086599 -0.368034
N8 -2.171862 -2.107288 -1.001869
H9 -3.049515 -1.931626 -1.464877
H10 -1.728941 -2.996497 -1.176558
N11 0.969415 1.898112 -0.727694
N12 -0.572362 -0.422323 2.325913

H13 -0.882090 -1.334029 2.630992
C14 0.739604 -0.419211 1.746127
H15 1.363549 0.385469 2.149350
H16 1.265479 -1.370059 1.885995
C17 2.979135 -1.156043 -0.362990
H18 3.312232 -1.348005 0.659261
H19 2.503946 -2.049580 -0.771603
H20 3.829578 -0.863073 -0.977527
H21 3.057064 1.529989 -1.176379
N22 -2.024919 0.133428 -0.447628
H23 -3.036004 0.158388 -0.388313
O24 -1.933646 2.354377 0.123293
H25 -0.693804 0.250466 3.070058

Zero-point correction= 0.203848 (Hartree/Particle)
Thermal correction to Energy= 0.216977
Thermal correction to Enthalpy= 0.217921
Thermal correction to Gibbs Free Energy= 0.165141
Sum of electronic and zero-point Energies= -676.475369
Sum of electronic and thermal Energies= -676.462240
Sum of electronic and thermal Enthalpies= -676.461295
Sum of electronic and thermal Free Energies= -676.514076

TS for 4-CH_2NH_2[9MG]$^+$
C1 0.189459 1.106338 -0.227131
C2 0.587651 -0.270120 -0.004366
C3 -1.460361 -1.033372 -0.589099
C4 -1.264124 1.414240 -0.187071
C5 2.263041 1.064462 -0.579793
N6 -0.204582 -1.329182 -0.468910
N7 1.970492 -0.206686 -0.400732
N8 -2.371398 -1.993907 -0.876460
H9 -3.244655 -1.756915 -1.321414
H10 -1.997723 -2.907962 -1.085065
N11 1.189276 1.906232 -0.494668
N12 -0.554933 -0.510535 2.343730
H13 -0.967107 -1.410027 2.523559
C14 0.676268 -0.411926 1.742306
H15 1.269200 0.446231 2.066823
H16 1.237775 -1.344967 1.807748
C17 2.860021 -1.356593 -0.471056
H18 3.098521 -1.711746 0.534771
H19 2.352439 -2.146167 -1.026659
H20 3.778023 -1.065939 -0.980232
H21 3.268762 1.412817 -0.782063
N22 -1.994041 0.248555 -0.407266
H23 -2.999962 0.372309 -0.400284
O24 -1.768947 2.492690 -0.008650
H25 -0.999123 0.291338 2.759428

Zero-point correction= 0.202313 (Hartree/Particle)
Thermal correction to Energy= 0.215269
Thermal correction to Enthalpy= 0.216214
Thermal correction to Gibbs Free Energy= 0.163631
Sum of electronic and zero-point Energies= -676.474540
Sum of electronic and thermal Energies= -676.461584
Sum of electronic and thermal Enthalpies= -676.460640
Sum of electronic and thermal Free Energies= -676.513222

5-CH_2NH_2[9MG]$^+$
C1 -0.275724 -0.906025 -0.013193
C2 -0.668919 0.529534 0.020337
C3 1.406618 1.269528 -0.243365
C4 0.974062 -1.072843 -0.850207
C5 -2.406496 -0.766884 -0.359069
N6 0.137879 1.559677 0.090529
N7 -2.000059 0.560148 -0.072736
N8 2.316909 2.221672 -0.106301
H9 3.294185 2.084571 -0.312810
H10 2.017016 3.115050 0.257415
N11 -1.465485 -1.626589 -0.404001
N12 1.197130 -0.677708 2.041186
H13 0.950334 -0.141771 2.861414
C14 0.094950 -1.397263 1.470094
H15 0.295285 -2.464390 1.349544
H16 -0.827126 -1.287717 2.047326
C17 -2.834299 1.756543 -0.072175
H18 -3.870257 1.459040 -0.230286
H19 -2.744796 2.266618 0.887765
H20 -2.518694 2.427703 -0.872896
H21 -3.456195 -0.975453 -0.531711
N22 1.772299 0.089905 -0.828266
H23 2.682639 -0.009999 -1.262510
O24 1.341477 -2.071868 -1.400022
H25 1.993932 -1.263992 2.249505

Zero-point correction= 0.205145 (Hartree/Particle)
Thermal correction to Energy= 0.217998
Thermal correction to Enthalpy= 0.218942
Thermal correction to Gibbs Free Energy= 0.166532
Sum of electronic and zero-point Energies= -676.513708
Sum of electronic and thermal Energies= -676.500856
Sum of electronic and thermal Enthalpies= -676.499911
Sum of electronic and thermal Free Energies= -676.552322

TS for 5-CH_2NH_2[9MG]$^+$
C1 -0.244741 -0.719866 -0.514480
C2 -0.731366 0.532095 -0.064480
C3 1.247283 1.545406 -0.077354
C4 1.141087 -0.790087 -0.935093
C5 -2.364040 -0.855710 -0.538426
N6 -0.032688 1.631144 0.249210
N7 -2.077972 0.410188 -0.030431
N8 2.045223 2.598053 0.159139
H9 3.000077 2.642192 -0.157710
H10 1.607971 3.449763 0.477390
N11 -1.313437 -1.541825 -0.862755
N12 1.262380 -1.501711 2.027098
H13 1.289668 -0.773218 2.727219
C14 0.152221 -1.823532 1.406794
H15 0.153670 -2.703584 0.772717
H16 -0.786711 -1.465227 1.814223
C17 -3.028403 1.455723 0.327017
H18 -4.008638 1.001864 0.470369
H19 -2.710138 1.935461 1.252757
H20 -3.081813 2.202764 -0.467039
H21 -3.387438 -1.194406 -0.635591
N22 1.811819 0.427026 -0.637018
H23 2.782700 0.437570 -0.927918
O24 1.762505 -1.728365 -1.381579
H25 2.141102 -1.930569 1.765645

Zero-point correction= 0.203132 (Hartree/Particle)
Thermal correction to Energy= 0.216159
Thermal correction to Enthalpy= 0.217103
Thermal correction to Gibbs Free Energy= 0.163849
Sum of electronic and zero-point Energies= -676.502356
Sum of electronic and thermal Energies= -676.489329
Sum of electronic and thermal Enthalpies= -676.488385
Sum of electronic and thermal Free Energies= -676.541639

8-CH_2NH_2[9MG]$^+$
C1 -0.099750 0.850401 -0.406644
C2 -0.036777 -0.609911 -0.281424
C3 -2.210210 -0.805597 0.211795
C4 -1.408584 1.512998 -0.185195
C5 1.976453 0.302032 -0.738513
N6 -1.041039 -1.419952 0.022257
N7 1.210490 -0.933135 -0.555339
N8 -3.263845 -1.556791 0.513184

H9 -4.187869 -1.183977 0.666717
H10 -3.128676 -2.555706 0.576502
N11 1.025626 1.370685 -0.720035
N12 2.479878 0.525430 1.747225
H13 2.159831 -0.340657 2.150388
C14 3.018319 0.505961 0.436350
H15 3.527379 1.447116 0.218805
H16 3.753858 -0.298414 0.341913
C17 1.784635 -2.267490 -0.532337
H18 2.489852 -2.370818 -1.358059
H19 2.303508 -2.440776 0.414841
H20 0.981504 -2.996071 -0.640339
H21 2.519000 0.287933 -1.689652
N22 -2.400633 0.552154 0.119948
H23 -3.320877 0.947481 0.280102
O24 -1.657425 2.683883 -0.237293
H25 1.944017 1.333723 2.022256

Zero-point correction=	0.204668 (Hartree/Particle)
Thermal correction to Energy=	0.217672
Thermal correction to Enthalpy=	0.218616
Thermal correction to Gibbs Free Energy=	0.165554
Sum of electronic and zero-point Energies=	-676.511656
Sum of electronic and thermal Energies=	-676.498652
Sum of electronic and thermal Enthalpies=	-676.497708
Sum of electronic and thermal Free Energies=	-676.550770

TS for 8-CH_2NH_2[9MG]$^+$

C1 0.016525 -0.644537 -0.498760
C2 0.288366 0.715248 -0.221488
C3 2.419314 0.384813 0.318928
C4 1.075976 -1.621871 -0.297464
C5 -1.813459 0.399114 -0.799106
N6 1.439661 1.264828 0.186251
N7 -0.874534 1.367712 -0.443990
N8 3.627696 0.820579 0.701605
H9 4.432082 0.220658 0.778965
H10 3.747260 1.811769 0.841615
N11 -1.250433 -0.817728 -0.886688
N12 -3.635671 -1.175926 0.904996
H13 -3.280851 -2.119779 0.817897
C14 -2.806748 -0.165943 1.055924
H15 -3.245513 0.816366 1.197799
H16 -1.830337 -0.362327 1.487799
C17 -1.095507 2.799229 -0.305010
H18 -1.376548 3.049814 0.721040
H19 -0.170529 3.319550 -0.553518
H20 -1.882444 3.109011 -0.993297
H21 -2.710856 0.679808 -1.336370
N22 2.269435 -0.962614 0.100417
H23 3.051992 -1.589973 0.244896
O24 1.041791 -2.822228 -0.417165
H25 -4.608468 -1.031349 0.674206

Zero-point correction=	0.202445 (Hartree/Particle)
Thermal correction to Energy=	0.215719
Thermal correction to Enthalpy=	0.216663
Thermal correction to Gibbs Free Energy=	0.161807
Sum of electronic and zero-point Energies=	-676.494079
Sum of electronic and thermal Energies=	-676.480805
Sum of electronic and thermal Enthalpies=	-676.479861
Sum of electronic and thermal Free Energies=	-676.534717

[9MG – H_{N2} + H_{N3}]···$^+CH_2NH_2$

C1 0.175442 0.180426 0.001848
C2 -1.138752 0.583854 -0.000005
C3 -1.992179 -1.653295 -0.000926
C4 0.481585 -1.227897 0.002374
C5 0.194703 2.311533 0.004072
N6 -2.205575 -0.265582 -0.001721
N7 -1.135482 1.941824 0.002100
N8 -2.863300 -2.570869 -0.002240
H9 -3.822631 -2.238549 -0.003429
N10 1.003977 1.287071 0.004396
N11 3.538305 0.027822 -0.000491
H12 3.000097 0.907742 0.002299
C13 4.804772 -0.036565 -0.006448
H14 5.290001 -1.008827 -0.009672
H15 5.403257 0.870531 -0.008418
C16 -2.295678 2.821512 -0.004489
H17 -1.949864 3.853614 0.041317
H18 -2.870201 2.684511 -0.923487
H19 -2.922399 2.626086 0.868715
H20 0.485374 3.352425 0.005637
N21 -0.637582 -2.020915 0.001572
H22 -0.487876 -3.023619 0.002113
O23 1.608960 -1.744126 0.003536
H24 2.878038 -0.811891 0.001354
H25 -3.157237 0.068115 -0.008590

Zero-point correction=	0.201953 (Hartree/Particle)
Thermal correction to Energy=	0.215923
Thermal correction to Enthalpy=	0.216867
Thermal correction to Gibbs Free Energy=	0.159709
Sum of electronic and zero-point Energies=	-676.513346
Sum of electronic and thermal Energies=	-676.499376
Sum of electronic and thermal Enthalpies=	-676.498432
Sum of electronic and thermal Free Energies=	-676.555590

TS for [9MG – H_{N2} + H_{N3}]···$^+CH_2NH_2$

C1 -0.196955 1.109398 -0.246955
C2 -0.607636 -0.233345 -0.409825
C3 1.541455 -1.025488 -0.735851
C4 1.254871 1.420407 -0.251989
C5 -2.301932 1.122271 -0.315362
N6 0.155866 -1.315877 -0.433048
N7 -1.990203 -0.187621 -0.407438
N8 2.340243 -1.902967 -1.161841
H9 1.885420 -2.806560 -1.277316
N10 -1.245363 1.934056 -0.212147
N11 0.050606 -0.015920 2.229266
H12 -0.867997 0.228127 2.574001
C13 0.420228 -1.325814 2.112429
H14 1.475031 -1.532304 2.289691
H15 -0.277604 -2.046730 2.540119
C16 -2.895777 -1.316145 -0.609447
H17 -3.920759 -0.951841 -0.551680
H18 -2.716660 -1.759051 -1.589951
H19 -2.733171 -2.066081 0.165339
H20 -3.328540 1.466694 -0.333438
N21 2.003981 0.269664 -0.478516
H22 2.997653 0.407430 -0.634146
O23 1.744143 2.503726 -0.054343
H24 0.754767 0.697737 2.368420
H25 0.287279 -1.644289 0.871765

Zero-point correction=	0.198072 (Hartree/Particle)
Thermal correction to Energy=	0.210811
Thermal correction to Enthalpy=	0.211755
Thermal correction to Gibbs Free Energy=	0.158886
Sum of electronic and zero-point Energies=	-676.408681
Sum of electronic and thermal Energies=	-676.395942
Sum of electronic and thermal Enthalpies=	-676.394997
Sum of electronic and thermal Free Energies=	-676.447867

[9MG – H_{N2} + H_{N3}]

C1 -0.197673 1.003888 0.002844
C2 -0.576153 -0.316147 -0.001375
C3 1.664247 -1.159249 -0.001730

C4 1.216725 1.340393 -0.001140
C5 -2.320434 0.992328 0.004863
N6 0.284458 -1.385214 -0.019648
O7 1.729717 2.439996 0.001598
N8 -1.934731 -0.341911 -0.003010
N9 2.577657 -2.051865 0.016151
H10 2.201585 -2.995625 0.012508
N11 -1.311190 1.812825 0.007017
C12 -2.790070 -1.510334 -0.000968
H13 -2.570518 -2.149051 -0.861385
H14 -2.669387 -2.079814 0.925240
H15 -3.827165 -1.181929 -0.073353
H16 -3.366637 1.265226 0.008912
N17 2.018176 0.181366 -0.012018
H18 3.016661 0.344395 -0.008553
H19 -0.042721 -2.334845 0.049441

Zero-point correction=	0.145483 (Hartree/Particle)
Thermal correction to Energy=	0.155880
Thermal correction to Enthalpy=	0.156825
Thermal correction to Gibbs Free Energy=	0.108653
Sum of electronic and zero-point Energies=	-581.544828
Sum of electronic and thermal Energies=	-581.534430
Sum of electronic and thermal Enthalpies=	-581.533486
Sum of electronic and thermal Free Energies=	-581.581658

2-CH_2NH_2[9MG – H_{N2} + H_{N3}]$^+$

C1 1.602293 0.591153 0.022014
C2 0.754177 -0.492641 -0.025058
C3 -1.185715 0.800019 -0.037425
C4 1.064516 1.930671 0.041739
C5 2.826725 -1.133546 0.002694
N6 -0.625994 -0.419856 -0.060111
O7 1.592979 3.005515 0.080402
N8 1.524853 -1.605145 -0.040085
N9 -2.524991 0.907499 -0.021226
H10 -2.920584 1.831913 0.064894
N11 2.903514 0.166726 0.038527
H12 -4.448376 0.210861 -0.215633
H13 -3.583372 -2.187846 0.042735
H14 -3.462046 -1.267002 1.396251
C15 1.086645 -2.991946 -0.089934
H16 1.969321 -3.630416 -0.108635
H17 0.497512 -3.240142 0.796177
H18 0.505722 -3.174153 -0.996928
H19 3.663692 -1.818105 0.005316
N20 -0.397596 1.884760 -0.016087
H21 -0.815065 2.808186 -0.059094
C22 -3.440367 -0.187863 -0.359976
H23 -3.321033 -0.451035 -1.416152
N24 -3.137205 -1.366202 0.438376
H25 -1.277295 -1.195939 0.147769

Zero-point correction=	0.206709 (Hartree/Particle)
Thermal correction to Energy=	0.219251
Thermal correction to Enthalpy=	0.220196
Thermal correction to Gibbs Free Energy=	0.167814
Sum of electronic and zero-point Energies=	-676.522633
Sum of electronic and thermal Energies=	-676.510090
Sum of electronic and thermal Enthalpies=	-676.509146
Sum of electronic and thermal Free Energies=	-676.561527

TS for 2-CH_2NH_2[9MG – H_{N2} + H_{N3}]$^+$

C1 1.749131 -0.392791 -0.012724
C2 0.739142 0.542835 0.012822
C3 -0.971051 -1.041281 0.001895
C4 1.431077 -1.800756 -0.032158
C5 2.683947 1.504455 0.015047
N6 -0.612407 0.252054 0.025476
O7 2.123943 -2.778246 -0.054660
N8 1.323203 1.763612 0.032983
N9 -2.275830 -1.360039 -0.035778
H10 -2.518211 -2.335874 -0.122092
N11 2.966638 0.232717 -0.010568
H12 -4.732067 -0.592792 0.251293
H13 -4.254139 1.911708 -0.009174
H14 -3.964652 1.016179 -1.354130
C15 0.669755 3.063475 0.066021
H16 1.439512 3.834024 0.094972
H17 0.064280 3.211331 -0.831425
H18 0.051530 3.154933 0.961980
H19 3.401642 2.313165 0.021603
N20 -0.020589 -1.987258 0.001090
H21 -0.286978 -2.965053 0.043513
C22 -3.802747 -0.038532 0.409592
H23 -3.745139 0.244631 1.465768
N24 -3.676443 1.169661 -0.391703
H25 -1.374838 0.914052 -0.198509

Zero-point correction=	0.201150 (Hartree/Particle)
Thermal correction to Energy=	0.214113
Thermal correction to Enthalpy=	0.215057
Thermal correction to Gibbs Free Energy=	0.162024
Sum of electronic and zero-point Energies=	-676.448654
Sum of electronic and thermal Energies=	-676.435691
Sum of electronic and thermal Enthalpies=	-676.434747
Sum of electronic and thermal Free Energies=	-676.487780

4-CH_2NH_2[9MG – H_{N2} + H_{N3}]$^+$

C1 0.082273 1.097422 -0.142514
C2 0.617273 -0.260036 0.113200
C3 -1.541965 -1.100586 -0.717853
C4 -1.394539 1.328063 -0.103240
C5 2.137881 1.217522 -0.609748
N6 -0.143828 -1.247556 -0.587192
N7 1.988411 -0.058494 -0.379222
N8 -2.366442 -1.998624 -1.048454
H9 -1.949810 -2.903594 -1.247933
N10 0.983236 1.958524 -0.494616
N11 -0.524581 -0.694966 2.356069
H12 -1.053354 -1.543005 2.233634
C13 0.710193 -0.539846 1.712145
H14 1.291562 0.274517 2.155379
H15 1.293992 -1.464211 1.792672
C16 3.005041 -1.099618 -0.469907
H17 3.927728 -0.663874 -0.851550
H18 3.190208 -1.530027 0.516518
H19 2.658797 -1.871748 -1.159753
H20 3.087853 1.661517 -0.886839
N21 -2.058373 0.180400 -0.457358
H22 -3.065992 0.243936 -0.566409
O23 -1.910207 2.381930 0.173152
H24 -1.063686 0.116556 2.612344
H25 0.118464 -2.208012 -0.400358

Zero-point correction=	0.203370 (Hartree/Particle)
Thermal correction to Energy=	0.216484
Thermal correction to Enthalpy=	0.217429
Thermal correction to Gibbs Free Energy=	0.164361
Sum of electronic and zero-point Energies=	-676.445956
Sum of electronic and thermal Energies=	-676.432842
Sum of electronic and thermal Enthalpies=	-676.431898
Sum of electronic and thermal Free Energies=	-676.484965

TS for 4-CH_2NH_2[9MG – H_{N2} + H_{N3}]$^+$

C1 0.171603 1.061756 -0.267478
C2 0.599733 -0.287647 -0.082520
C3 -1.610168 -1.111566 -0.627330
C4 -1.282594 1.369789 -0.266373
C5 2.265797 1.087768 -0.495815

N6 -0.220378 -1.330880 -0.535018
N7 1.980505 -0.210533 -0.386827
N8 -2.504689 -1.984508 -0.817880
H9 -2.151445 -2.928684 -0.943593
N10 1.185604 1.891061 -0.435733
N11 -0.628337 -0.424055 2.359636
H12 -1.064562 -1.323122 2.489618
C13 0.603935 -0.303590 1.823217
H14 1.138182 0.617483 2.053703
H15 1.209330 -1.204294 1.921800
C16 2.902397 -1.335254 -0.453918
H17 3.890007 -0.965744 -0.727391
H18 2.968632 -1.835945 0.515839
H19 2.559323 -2.037732 -1.216255
H20 3.279809 1.450925 -0.610827
N21 -2.022043 0.224058 -0.485683
H22 -3.025313 0.346682 -0.582470
O23 -1.760001 2.466129 -0.106669
H24 -1.192444 0.386074 2.569646
H25 0.029633 -2.278232 -0.284890

Zero-point correction=	0.202765 (Hartree/Particle)
Thermal correction to Energy=	0.215388
Thermal correction to Enthalpy=	0.216332
Thermal correction to Gibbs Free Energy=	0.164264
Sum of electronic and zero-point Energies=	-676.443560
Sum of electronic and thermal Energies=	-676.430937
Sum of electronic and thermal Enthalpies=	-676.429993
Sum of electronic and thermal Free Energies=	-676.482061

5-CH$_2$NH$_2$[9MG – H$_{N2}$ + H$_{N3}$]$^+$

C1 -0.222898 -0.900158 0.017980
C2 -0.697311 0.502280 0.030143
C3 1.514171 1.351715 -0.273091
C4 1.026305 -1.034899 -0.854288
C5 -2.367898 -0.875818 -0.324657
N6 0.131211 1.529758 0.079828
N7 -2.017469 0.490632 -0.073949
N8 2.408444 2.228655 -0.180165
H9 2.114431 3.109205 0.231970
N10 -1.383317 -1.676328 -0.346337
N11 1.244366 -0.541835 2.038969
H12 1.063746 -0.221348 2.979880
C13 0.198696 -1.355727 1.499082
H14 0.454191 -2.411954 1.372343
H15 -0.709387 -1.295438 2.104129
C16 -2.914122 1.641823 -0.111272
H17 -3.925358 1.285853 -0.304042
H18 -2.903212 2.161019 0.849224
H19 -2.621525 2.319396 -0.917804
H20 -3.408907 -1.129938 -0.487250
N21 1.781572 0.124348 -0.872644
H22 2.703524 0.056335 -1.290607
O23 1.362421 -2.056067 -1.389985
H24 2.160580 -0.969255 1.999556
H25 -0.202754 2.482763 0.159183

Zero-point correction=	0.204545 (Hartree/Particle)
Thermal correction to Energy=	0.217315
Thermal correction to Enthalpy=	0.218259
Thermal correction to Gibbs Free Energy=	0.165999
Sum of electronic and zero-point Energies=	-676.479335
Sum of electronic and thermal Energies=	-676.466565
Sum of electronic and thermal Enthalpies=	-676.465621
Sum of electronic and thermal Free Energies=	-676.517881

TS for 5-CH$_2$NH$_2$[9MG – H$_{N2}$ + H$_{N3}$]$^+$

C1 -0.193188 -0.757675 -0.402810
C2 -0.776738 0.472154 -0.031728
C3 1.301077 1.663711 -0.121544
C4 1.186355 -0.733208 -0.919078
C5 -2.311739 -1.013738 -0.494067
N6 -0.071982 1.590032 0.237896
N7 -2.107832 0.299346 -0.029562
N8 2.056889 2.664342 0.024693
H9 1.610311 3.485548 0.421672
N10 -1.217720 -1.637776 -0.763350
N11 1.446213 -1.295208 1.979884
H12 1.456399 -0.542890 2.653350
C13 0.328302 -1.731596 1.430540
H14 0.377703 -2.652807 0.859077
H15 -0.607745 -1.483208 1.919846
C16 -3.130089 1.297428 0.262390
H17 -4.106452 0.817622 0.207327
H18 -2.996305 1.697937 1.269734
H19 -3.093925 2.102744 -0.475430
H20 -3.314159 -1.408896 -0.595505
N21 1.810534 0.488190 -0.670251
H22 2.771068 0.567488 -0.986596
O23 1.781750 -1.668205 -1.402164
H24 2.342958 -1.639220 1.661791
H25 -0.530443 2.456387 0.484670

Zero-point correction=	0.203149 (Hartree/Particle)
Thermal correction to Energy=	0.215963
Thermal correction to Enthalpy=	0.216908
Thermal correction to Gibbs Free Energy=	0.163932
Sum of electronic and zero-point Energies=	-676.471111
Sum of electronic and thermal Energies=	-676.458296
Sum of electronic and thermal Enthalpies=	-676.457352
Sum of electronic and thermal Free Energies=	-676.510328

7-CH$_2$NH$_2$[9MG – H$_{N2}$ + H$_{N3}$]$^+$

C1 -0.321127 -0.183387 -0.125153
C2 0.599134 0.819388 -0.007465
C3 2.441602 -0.716443 0.063712
C4 0.082302 -1.584875 -0.155631
C5 -1.397047 1.716098 -0.138699
N6 1.936787 0.605715 0.089202
N7 -0.078817 2.010197 -0.016587
N8 3.651389 -1.066338 0.140131
H9 4.315877 -0.303817 0.226766
N10 -1.564563 0.408336 -0.207577
N11 -3.207570 -1.203917 0.707574
H12 -3.423471 -0.800369 1.606063
C13 -2.903976 -0.340285 -0.341497
H14 -2.813277 -0.885759 -1.280872
H15 -3.641445 0.458145 -0.445685
C16 0.508217 3.344746 0.090722
H17 -0.286219 4.085545 0.013224
H18 1.003522 3.455021 1.057264
H19 1.216813 3.501475 -0.724784
H20 -2.183016 2.455435 -0.174742
N21 1.455881 -1.705736 -0.060181
H22 1.826563 -2.650017 -0.077419
O23 -0.668717 -2.535802 -0.251232
H24 -2.669923 -2.059841 0.741408
H25 2.597925 1.361346 0.184759

Zero-point correction=	0.205666 (Hartree/Particle)
Thermal correction to Energy=	0.218615
Thermal correction to Enthalpy=	0.219559
Thermal correction to Gibbs Free Energy=	0.166687
Sum of electronic and zero-point Energies=	-676.512939
Sum of electronic and thermal Energies=	-676.499990
Sum of electronic and thermal Enthalpies=	-676.499046
Sum of electronic and thermal Free Energies=	-676.551918

TS for 7-CH$_2$NH$_2$[9MG – H$_{N2}$ + H$_{N3}$]$^+$

C1 -0.317932 0.007326 -0.082029

C2 0.850093 0.720585 0.008368
C3 2.219695 -1.245562 0.036123
C4 -0.286682 -1.442171 -0.119084
C5 -0.853199 2.087654 -0.084554
N6 2.087961 0.155514 0.076546
N7 0.516296 2.041531 0.008829
N8 3.291202 -1.916011 0.075901
H9 4.136611 -1.357740 0.141448
N10 -1.377330 0.888594 -0.139657
N11 -3.540178 -0.801225 0.587816
H12 -3.956178 -0.526959 1.466691
C13 -3.327457 0.058309 -0.364534
H14 -2.979989 -0.322488 -1.315352
H15 -3.815911 1.023592 -0.324637
C16 1.430583 3.173416 0.093905
H17 0.853443 4.095420 0.034790
H18 1.966005 3.154707 1.045704
H19 2.136044 3.149869 -0.739567
H20 -1.392375 3.023489 -0.109873
N21 1.000731 -1.932671 -0.054907
H22 1.102827 -2.941030 -0.083522
O23 -1.257066 -2.189157 -0.198624
H24 -3.064250 -1.701508 0.524212
H25 2.928926 0.708438 0.138232

Zero-point correction=	0.203927 (Hartree/Particle)
Thermal correction to Energy=	0.216733
Thermal correction to Enthalpy=	0.217677
Thermal correction to Gibbs Free Energy=	0.164623
Sum of electronic and zero-point Energies=	-676.503545
Sum of electronic and thermal Energies=	-676.490739
Sum of electronic and thermal Enthalpies=	-676.489795
Sum of electronic and thermal Free Energies=	-676.542850

8-CH_2NH_2[9MG – H_{N2} + H_{N3}]$^+$

C1 0.138137 -0.843419 -0.415091
C2 -0.010898 0.609976 -0.316774
C3 2.321222 0.878322 0.242627
C4 1.478066 -1.462504 -0.185769
C5 -1.977334 -0.390169 -0.727017
N6 1.011568 1.401473 -0.003771
N7 -1.252806 0.894430 -0.605664
N8 3.329122 1.562621 0.555218
H9 3.178359 2.564029 0.630588
N10 -0.969664 -1.408930 -0.695785
N11 -2.180661 -0.477387 1.717160
H12 -2.717970 -0.045204 2.457751
C13 -2.931165 -0.588903 0.487754
H14 -3.426369 -1.552876 0.333335
H15 -3.697423 0.189255 0.439380
C16 -1.899275 2.194052 -0.527929
H17 -2.829393 2.158658 -1.094877
H18 -2.113535 2.443541 0.515615
H19 -1.259123 2.956286 -0.977305
H20 -2.535567 -0.424098 -1.667753
N21 2.436821 -0.503692 0.108737
H22 3.374983 -0.850807 0.282735
O23 1.706538 -2.640853 -0.244289
H24 -1.851870 -1.376058 2.051343
H25 0.892434 2.400362 0.105442

Zero-point correction=	0.205402 (Hartree/Particle)
Thermal correction to Energy=	0.218062
Thermal correction to Enthalpy=	0.219006
Thermal correction to Gibbs Free Energy=	0.166357
Sum of electronic and zero-point Energies=	-676.484575
Sum of electronic and thermal Energies=	-676.471916
Sum of electronic and thermal Enthalpies=	-676.470971
Sum of electronic and thermal Free Energies=	-676.523620

TS for 8-CH_2NH_2[9MG – H_{N2} + H_{N3}]$^+$

C1 -0.002342 -0.684124 -0.607543
C2 -0.054438 0.684113 -0.287498
C3 2.279254 0.780845 0.272108
C4 1.250173 -1.431328 -0.405812
C5 -2.014211 -0.065219 -0.907469
N6 1.008050 1.400564 0.146019
N7 -1.315271 1.086712 -0.508556
N8 3.356196 1.351155 0.603968
H9 3.276117 2.347943 0.780203
N10 -1.186557 -1.103728 -1.052164
N11 -1.617553 -0.994913 1.805261
H12 -1.355742 -0.260526 2.446990
C13 -2.682871 -0.906408 1.038166
H14 -3.052635 -1.807193 0.566100
H15 -3.368746 -0.086315 1.217125
C16 -1.853696 2.431757 -0.363756
H17 -2.921988 2.404895 -0.577632
H18 -1.706298 2.799666 0.655209
H19 -1.376163 3.108268 -1.076433
H20 -2.973709 0.026730 -1.400502
N21 2.277365 -0.591641 0.023077
H22 3.184391 -1.028266 0.147421
O23 1.398378 -2.621620 -0.546984
H24 -1.044086 -1.829425 1.800896
H25 0.965018 2.402331 0.264071

Zero-point correction=	0.202689 (Hartree/Particle)
Thermal correction to Energy=	0.215556
Thermal correction to Enthalpy=	0.216500
Thermal correction to Gibbs Free Energy=	0.163402
Sum of electronic and zero-point Energies=	-676.465124
Sum of electronic and thermal Energies=	-676.452257
Sum of electronic and thermal Enthalpies=	-676.451313
Sum of electronic and thermal Free Energies=	-676.504410

[9MG – H_{N2} + H_{C5}]···$^+CH_2NH_2$

C1 -0.136738 0.069484 0.587094
C2 1.225955 0.581745 0.204735
C3 2.116287 -1.482561 0.010694
C4 -0.361140 -1.294050 -0.046976
C5 -0.296271 2.173489 0.031936
N6 0.789005 -1.978101 -0.191785
N7 2.288542 -0.090144 -0.001306
O8 -1.466770 -1.758535 -0.321130
N9 1.060447 1.934716 0.028294
N10 3.030064 -2.349031 0.089770
H11 3.940915 -1.900549 0.169122
N12 -1.056890 1.164469 0.289463
N13 -3.530829 -0.084925 -0.037884
H14 -2.919961 -0.922559 -0.196714
C15 -4.798879 -0.084132 -0.076730
H16 -0.147646 -0.111280 1.674471
H17 -5.349037 0.835716 0.101287
H18 -5.331083 -1.007392 -0.288728
H19 -0.666862 3.170308 -0.181619
C20 2.118171 2.841159 -0.398415
H21 2.547182 2.493669 -1.340752
H22 1.696415 3.837805 -0.527206
H23 2.899198 2.872236 0.362005
H24 -2.936135 0.743376 0.155410
H25 0.734501 -2.952752 -0.468135

Zero-point correction=	0.202654 (Hartree/Particle)
Thermal correction to Energy=	0.216075
Thermal correction to Enthalpy=	0.217019
Thermal correction to Gibbs Free Energy=	0.161593
Sum of electronic and zero-point Energies=	-676.483032
Sum of electronic and thermal Energies=	-676.469612
Sum of electronic and thermal Enthalpies=	-676.468667

Sum of electronic and thermal Free Energies= -676.524094

TS for [9MG – H_{N2} + H_{C5}]···$^{+}CH_2NH_2$

C1 -0.199456 -0.487343 -0.492194
C2 0.098145 0.898037 -0.064361
C3 2.287338 0.481722 0.363082
C4 0.980947 -1.394493 -0.681390
C5 -1.896213 0.696541 -0.933351
N6 2.120880 -0.808787 -0.181407
N7 1.199762 1.389152 0.325688
O8 0.914710 -2.506102 -1.144736
N9 -1.102785 1.576725 -0.296071
N10 3.418382 0.780736 0.843410
H11 3.429345 1.742743 1.177906
N12 -1.378899 -0.518360 -1.138370
N13 -2.660164 -1.420510 1.090140
H14 -3.059739 -2.050047 0.409910
C15 -1.401431 -1.635510 1.573269
H16 -0.601723 -1.035498 0.846873
H17 -1.207692 -1.206597 2.555984
H18 -1.019374 -2.648813 1.437019
H19 -2.880960 0.983490 -1.286019
C20 -1.320377 3.002805 -0.066120
H21 -0.652446 3.583424 -0.704332
H22 -2.357663 3.240114 -0.300415
H23 -1.116578 3.232412 0.979882
H24 -3.294105 -0.790484 1.556779
H25 2.969305 -1.365179 -0.213034

Zero-point correction= 0.198145 (Hartree/Particle)
Thermal correction to Energy= 0.211204
Thermal correction to Enthalpy= 0.212148
Thermal correction to Gibbs Free Energy= 0.158371
Sum of electronic and zero-point Energies= -676.412249
Sum of electronic and thermal Energies= -676.399190
Sum of electronic and thermal Enthalpies= -676.398246
Sum of electronic and thermal Free Energies= -676.452023

[9MG – H_{N2} + H_{C5}]

C1 -0.197673 1.003888 0.002844
C2 -0.576153 -0.316147 -0.001375
C3 1.664247 -1.159249 -0.001730
C4 1.216725 1.340393 -0.001140
C5 -2.320434 0.992328 0.004863
N6 0.284458 -1.385214 -0.019648
O7 1.729717 2.439996 0.001598
N8 -1.934731 -0.341911 -0.003010
N9 2.577657 -2.051865 0.016151
H10 2.201585 -2.995625 0.012508
N11 -1.311190 1.812825 0.007017
C12 -2.790070 -1.510334 -0.000968
H13 -2.570518 -2.149051 -0.861385
H14 -2.669387 -2.079814 0.925240
H15 -3.827165 -1.181929 -0.073353
H16 -3.366637 1.265226 0.008912
N17 2.018176 0.181366 -0.012018
H18 3.016661 0.344395 -0.008553
H19 -0.042721 -2.334845 0.049441

Zero-point correction= 0.145483 (Hartree/Particle)
Thermal correction to Energy= 0.155880
Thermal correction to Enthalpy= 0.156825
Thermal correction to Gibbs Free Energy= 0.108653
Sum of electronic and zero-point Energies= -581.544828
Sum of electronic and thermal Energies= -581.534430
Sum of electronic and thermal Enthalpies= -581.533486
Sum of electronic and thermal Free Energies= -581.581658

[9MG + H_{C5}]$^{+}$···CH_2NH

C1 1.870837 -0.335297 -0.521731
C2 0.732865 0.597891 -0.229820
C3 -0.783700 -1.034187 -0.076526
C4 1.602085 -1.677724 0.132928
C5 2.695490 1.584844 0.019628
N6 -0.534493 0.291418 -0.154452
O7 2.404081 -2.465238 0.537869
N8 1.291526 1.788601 -0.000188
N9 -2.030222 -1.443564 -0.120165
H10 -2.254260 -2.427396 -0.081235
N11 3.080593 0.393046 -0.214455
H12 -5.916533 1.233087 0.285811
H13 -4.543124 0.528682 -1.369359
C14 0.599300 3.022233 0.353235
H15 0.950124 3.832628 -0.286974
H16 -0.467847 2.867942 0.197905
H17 0.784749 3.266048 1.401259
H18 3.350243 2.420347 0.240199
N19 0.217412 -1.960542 0.116813
H20 -0.020509 -2.897538 0.423321
C21 -5.023490 0.633350 0.474738
H22 -4.804935 0.374522 1.510726
N23 -4.241530 0.228699 -0.441786
H24 1.868671 -0.551719 -1.604115
H25 -2.822558 -0.754963 -0.235575

Zero-point correction= 0.201082 (Hartree/Particle)
Thermal correction to Energy= 0.215191
Thermal correction to Enthalpy= 0.216135
Thermal correction to Gibbs Free Energy= 0.157930
Sum of electronic and zero-point Energies= -676.489536
Sum of electronic and thermal Energies= -676.475427
Sum of electronic and thermal Enthalpies= -676.474483
Sum of electronic and thermal Free Energies= -676.532688

CH_2NH

N1 -0.668480 -0.154105 0.000063
C2 0.587037 0.028696 0.000111
H3 1.242805 -0.843547 -0.000555
H4 1.077032 1.009505 -0.000024
H5 -1.162700 0.740597 -0.000533

Zero-point correction= 0.040426 (Hartree/Particle)
Thermal correction to Energy= 0.043343
Thermal correction to Enthalpy= 0.044287
Thermal correction to Gibbs Free Energy= 0.018513
Sum of electronic and zero-point Energies= -94.562478
Sum of electronic and thermal Energies= -94.559561
Sum of electronic and thermal Enthalpies= -94.558617
Sum of electronic and thermal Free Energies= -94.584391

[9MG – H_{N2} + H_{C5} + H_{N7}]$^{+}$···CH_2NH

C1 -0.176662 -0.080784 0.570340
C2 1.079521 0.660420 0.219673
C3 2.301589 -1.234502 0.066001
C4 -0.170277 -1.440077 -0.128324
C5 -0.650379 2.008339 -0.058539
N6 2.234101 0.179269 0.033272
N7 0.695095 1.984985 -0.007926
N8 3.355135 -1.909729 0.215232
H9 4.165090 -1.301804 0.322424
N10 -1.216987 0.867009 0.212385
N11 -3.788379 0.279307 0.254787
C12 -4.191600 -0.854279 -0.164074
H13 -5.244017 -1.141330 -0.158163
H14 -3.453517 -1.564272 -0.535028
C15 1.619987 3.042999 -0.408120
H16 1.066049 3.974231 -0.521838
H17 2.379293 3.160115 0.365080
H18 2.099722 2.772381 -1.350372
H19 -1.200527 2.906685 -0.313384

N20 1.094692 -1.935838 -0.203448
H21 1.210115 -2.900976 -0.493388
O22 -1.180309 -2.003251 -0.489199
H23 -4.538280 0.881136 0.594034
H24 -0.212936 -0.281183 1.651404
H25 -2.317191 0.653320 0.240979

Zero-point correction= 0.200498 (Hartree/Particle)
Thermal correction to Energy= 0.214084
Thermal correction to Enthalpy= 0.215028
Thermal correction to Gibbs Free Energy= 0.159675
Sum of electronic and zero-point Energies= -676.479394
Sum of electronic and thermal Energies= -676.465807
Sum of electronic and thermal Enthalpies= -676.464863
Sum of electronic and thermal Free Energies= -676.520216

[9MG – H_{N2} + H_{C5} + H_{N7}]$^+$
C1 0.096390 0.916230 0.612415
C2 0.480829 -0.481895 0.235767
C3 -1.674689 -1.139655 0.031461
C4 -1.196381 1.297147 -0.117326
C5 2.266641 0.803576 -0.021880
N6 -2.018277 0.213994 -0.220570
N7 -0.288172 -1.454238 0.010069
O8 -1.427798 2.427646 -0.468989
N9 1.874362 -0.467512 0.011490
N10 -2.602443 -1.980927 0.157717
H11 -2.235994 -2.926669 0.249639
N12 1.300013 1.648822 0.261826
H13 -0.096345 0.996835 1.691552
H14 3.277625 1.104635 -0.270246
C15 2.646438 -1.650359 -0.375578
H16 2.223903 -2.067366 -1.290904
H17 3.684258 -1.360531 -0.535014
H18 2.585638 -2.387695 0.424599
H19 -2.973759 0.370557 -0.524520
H20 1.383310 2.657831 0.273924

Zero-point correction= 0.159458 (Hartree/Particle)
Thermal correction to Energy= 0.169265
Thermal correction to Enthalpy= 0.170210
Thermal correction to Gibbs Free Energy= 0.124201
Sum of electronic and zero-point Energies= -581.876385
Sum of electronic and thermal Energies= -581.866578
Sum of electronic and thermal Enthalpies= -581.865633
Sum of electronic and thermal Free Energies= -581.911642

[9MG – H_{N2} + H_{O6} + H_{N7}]$^+$···CH_2NH
C1 0.142242 0.165584 0.064819
C2 -1.229709 0.479422 -0.007894
C3 -1.949308 -1.664724 -0.053664
C4 0.482833 -1.183762 0.085607
C5 -0.037210 2.360333 0.064628
N6 -2.252901 -0.306593 -0.066407
N7 -1.283059 1.883219 -0.003360
N8 -2.750478 -2.643802 -0.101588
H9 -3.711838 -2.317830 -0.154008
N10 0.842126 1.369239 0.107298
N11 3.706847 0.059078 0.362389
C12 4.577329 0.147113 -0.565180
H13 5.620990 0.409570 -0.383280
H14 4.279277 -0.055708 -1.593513
C15 -2.527097 2.643910 -0.072100
H16 -2.305200 3.704471 0.040580
H17 -3.008211 2.457670 -1.032968
H18 -3.186357 2.310321 0.729676
H19 0.216404 3.410026 0.083192
N20 -0.554921 -2.022704 0.025029
H21 -0.377148 -3.021735 0.039366
O22 1.676185 -1.705274 0.154492
H23 4.104661 0.241339 1.284957
H24 1.850131 1.461239 0.160249
H25 2.400037 -1.013475 0.228992

Zero-point correction= 0.202516 (Hartree/Particle)
Thermal correction to Energy= 0.216376
Thermal correction to Enthalpy= 0.217320
Thermal correction to Gibbs Free Energy= 0.160810
Sum of electronic and zero-point Energies= -676.480205
Sum of electronic and thermal Energies= -676.466346
Sum of electronic and thermal Enthalpies= -676.465402
Sum of electronic and thermal Free Energies= -676.521912

TS for [9MG – H_{N2} + H_{O6} + H_{N7}]$^+$···CH_2NH
C1 -0.211923 0.225803 -0.368793
C2 1.180245 0.442892 -0.040021
C3 1.676706 -1.772191 0.136514
C4 -0.645176 -1.120235 -0.468680
C5 0.086118 2.320678 -0.273674
N6 2.083925 -0.428258 0.212820
N7 1.308158 1.830757 -0.007183
N8 2.446127 -2.761402 0.337291
H9 3.393997 -2.449006 0.536957
N10 -0.862556 1.397369 -0.481085
C11 2.540349 2.558944 0.282850
H12 2.863833 2.335539 1.300230
H13 3.312175 2.252055 -0.423398
H14 2.348569 3.626175 0.178819
H15 -0.101146 3.386559 -0.318422
N16 0.327149 -2.038899 -0.181035
H17 0.095001 -3.020919 -0.281156
O18 -1.821573 -1.464363 -0.773507
H19 -2.744625 -1.110123 0.151097
H20 -2.415404 -0.181409 1.595486
C21 -3.302643 -0.326051 0.959125
N22 -3.655717 0.786957 0.250987
H23 -2.920413 1.333575 -0.192756
H24 -4.600965 0.901472 -0.086635
H25 -4.130095 -0.843726 1.441341

Zero-point correction= 0.195881 (Hartree/Particle)
Thermal correction to Energy= 0.208965
Thermal correction to Enthalpy= 0.209909
Thermal correction to Gibbs Free Energy= 0.156002
Sum of electronic and zero-point Energies= -676.383465
Sum of electronic and thermal Energies= -676.370380
Sum of electronic and thermal Enthalpies= -676.369436
Sum of electronic and thermal Free Energies= -676.423344

[9MG – H_{N2} + H_{O6} + H_{N7}]$^+$
C1 -0.088263 0.950776 -0.001374
C2 -0.550773 -0.388951 -0.000702
C3 1.499733 -1.349300 -0.001050
C4 1.287773 1.121582 0.001815
C5 -2.297815 0.988516 -0.004466
N6 0.113559 -1.491240 0.001018
O7 1.992306 2.239050 0.003893
N8 -1.954199 -0.296541 -0.003181
N9 2.380752 -2.254362 -0.005167
H10 1.956138 -3.177951 -0.006938
N11 -1.223471 1.769165 -0.003440
H12 -1.272133 2.777436 -0.007936
C13 -2.838259 -1.460952 0.007293
H14 -3.870533 -1.125583 -0.083957
H15 -2.574511 -2.106030 -0.831026
H16 -2.696049 -2.005118 0.941490
H17 -3.314940 1.353960 -0.006670
N18 2.008265 0.000015 0.002665
H19 3.021645 0.068262 0.003941
H20 1.463220 3.043336 0.007596

Zero-point correction=	0.158896 (Hartree/Particle)
Thermal correction to Energy=	0.169264
Thermal correction to Enthalpy=	0.170208
Thermal correction to Gibbs Free Energy=	0.122804
Sum of electronic and zero-point Energies=	-581.877324
Sum of electronic and thermal Energies=	-581.866956
Sum of electronic and thermal Enthalpies=	-581.866012
Sum of electronic and thermal Free Energies=	-581.913415

Cartesian coordinates for structures in Table S6, optimized at ωB97XD/6-31+G(d,p)

[9MG – H_{N2} + H_{O6}]$^{•+}$···$^{•}CH_2NH_2$

C1 -0.181761 0.164160 0.031746
C2 1.182210 0.531548 0.009821
C3 1.953079 -1.580739 -0.055195
C4 -0.480792 -1.233903 0.027386
C5 -0.128542 2.275130 0.063057
N6 2.242278 -0.253667 -0.032234
N7 1.177410 1.903975 0.033929
N8 2.832812 -2.533423 -0.102807
H9 3.772362 -2.134139 -0.119851
N10 -0.979994 1.259805 0.060770
C11 2.358104 2.762749 0.027162
H12 2.966362 2.553912 0.907806
H13 2.941058 2.573972 -0.874856
H14 2.031457 3.801759 0.043644
H15 -0.420688 3.316637 0.085491
N16 0.610695 -2.017787 -0.024117
H17 0.478696 -3.025790 -0.030843
O18 -1.621606 -1.796880 0.063553
H19 -2.513131 -1.130152 0.233404
H20 -3.389653 0.197356 1.407488
C21 -3.749751 -0.306097 0.510303
N22 -3.931459 0.514899 -0.568297
H23 -3.284156 1.279005 -0.706242
H24 -4.465801 0.208375 -1.367168
H25 -4.521129 -1.059597 0.656308

Zero-point correction=	0.196165 (Hartree/Particle)
Thermal correction to Energy=	0.209842
Thermal correction to Enthalpy=	0.210786
Thermal correction to Gibbs Free Energy=	0.155191
Sum of electronic and zero-point Energies=	-676.431202
Sum of electronic and thermal Energies=	-676.417524
Sum of electronic and thermal Enthalpies=	-676.416580
Sum of electronic and thermal Free Energies=	-676.472175

TS for [9MG – H_{N2} + H_{O6}]$^{•+}$···$^{•}CH_2NH_2$

C1 -0.190109 0.149864 0.004742
C2 1.164402 0.539267 0.008130
C3 1.972849 -1.559702 -0.041801
C4 -0.478660 -1.259676 -0.003907
C5 -0.169607 2.266300 0.032112
N6 2.240487 -0.226995 -0.013292
N7 1.139465 1.913628 0.028029
N8 2.875440 -2.491762 -0.073508
H9 3.804226 -2.067981 -0.072734
N10 -1.005031 1.236321 0.015861
C11 2.308357 2.787203 0.057561
H12 2.822044 2.681765 1.013965
H13 2.983927 2.512283 -0.752646
H14 1.977702 3.816860 -0.073484
H15 -0.477208 3.303453 0.045592
N16 0.640390 -2.019774 -0.035223
H17 0.526992 -3.029348 -0.044103
O18 -1.604541 -1.821506 0.012044
H19 -2.662487 -1.085555 0.236004
H20 -3.379764 0.080800 1.450924
C21 -3.726647 -0.371161 0.518739
N22 -3.875465 0.527762 -0.502705
H23 -3.171147 1.254281 -0.593031
H24 -4.439042 0.307463 -1.312401
H25 -4.539444 -1.088804 0.617965

Zero-point correction=	0.195333 (Hartree/Particle)
Thermal correction to Energy=	0.208180
Thermal correction to Enthalpy=	0.209124
Thermal correction to Gibbs Free Energy=	0.155947
Sum of electronic and zero-point Energies=	-676.431681
Sum of electronic and thermal Energies=	-676.418834
Sum of electronic and thermal Enthalpies=	-676.417889
Sum of electronic and thermal Free Energies=	-676.471066

[9MG – H_{N2} + H_{O6}]$^{•+}$

C1 -0.110547 0.940424 -0.000058
C2 -0.596607 -0.387486 0.000014
C3 1.456629 -1.316060 0.000128
C4 1.286673 1.113583 -0.000032
C5 -2.203024 1.094163 -0.000114
N6 0.110481 -1.499823 0.000104
O7 1.925054 2.247680 -0.000089
N8 -1.959857 -0.247996 -0.000019
N9 2.346234 -2.259947 0.000214
H10 1.888911 -3.173186 0.000261
N11 -1.111931 1.844853 -0.000136
C12 -2.935279 -1.336641 -0.000055
H13 -2.798540 -1.947766 -0.892716
H14 -2.797701 -1.948528 0.891951
H15 -3.935177 -0.904996 0.000611
H16 -3.211701 1.485966 -0.000159
N17 2.005100 -0.006676 0.000057
H18 3.021907 0.043557 0.000078
H19 1.314614 3.002718 -0.000152

Zero-point correction=	0.145907 (Hartree/Particle)
Thermal correction to Energy=	0.155614
Thermal correction to Enthalpy=	0.156559
Thermal correction to Gibbs Free Energy=	0.109434
Sum of electronic and zero-point Energies=	-581.269005
Sum of electronic and thermal Energies=	-581.259298
Sum of electronic and thermal Enthalpies=	-581.258353
Sum of electronic and thermal Free Energies=	-581.305478

2-CH_2NH_2[9MG – H_{N2} + H_{O6}]$^{+}$

C1 -1.563321 0.541418 0.056942
C2 -0.701971 -0.565271 -0.070506
C3 1.161748 0.663262 -0.189681
C4 -0.971754 1.781344 0.055729
C5 -2.810574 -1.148499 0.088071
N6 0.628029 -0.548042 -0.181880
N7 -1.519999 -1.644373 -0.048499
N8 2.481608 0.823241 -0.325883
N9 -2.880178 0.148960 0.155696
N10 3.638803 -1.060349 0.798370
H11 2.827744 -1.550732 1.148716
C12 3.412114 -0.342364 -0.404598
H13 4.349981 0.069592 -0.779393
H14 3.005322 -1.015469 -1.160075
C15 -1.113395 -3.039773 -0.162343
H16 -2.004511 -3.664968 -0.118108
H17 -0.604616 -3.199538 -1.113959
H18 -0.445971 -3.300828 0.660005
H19 -3.657962 -1.820033 0.129498
N20 0.383928 1.810250 -0.068694
H21 0.817500 2.726621 -0.094032
O22 -1.546601 2.955294 0.156809
H23 4.108364 -0.543760 1.529076
H24 -2.508600 2.863228 0.236185
H25 2.883126 1.745022 -0.237830

Zero-point correction=	0.206347 (Hartree/Particle)
Thermal correction to Energy=	0.219147
Thermal correction to Enthalpy=	0.220091
Thermal correction to Gibbs Free Energy=	0.167052
Sum of electronic and zero-point Energies=	-676.526708
Sum of electronic and thermal Energies=	-676.513908
Sum of electronic and thermal Enthalpies=	-676.512964

Sum of electronic and thermal Free Energies= -676.566003

TS for 2-CH_2NH_2[9MG – H_{N2} + H_{O6}]$^+$

C1 1.692180 -0.342298 -0.184897
C2 0.684278 0.593634 -0.068837
C3 -1.025286 -0.980445 -0.034692
C4 1.377138 -1.747737 -0.188765
C5 2.619955 1.557809 0.029721
N6 -0.663990 0.300498 0.020496
O7 2.084680 -2.724612 0.245517
N8 1.262278 1.814428 0.055021
N9 -2.340894 -1.290427 0.006510
H10 -2.633761 -2.245833 -0.162125
N11 2.908992 0.288919 -0.085094
H12 -4.697634 -0.639454 0.292855
H13 -3.984285 2.039442 0.135370
H14 -4.214567 1.035880 -1.261517
C15 0.597180 3.108309 0.134464
H16 1.359729 3.872844 0.297055
H17 0.070910 3.326923 -0.803796
H18 -0.092440 3.118628 0.987508
H19 3.340056 2.362619 0.098835
N20 -0.087455 -1.941738 -0.133992
H21 -0.349344 -2.911772 0.005941
C22 -3.720692 -0.118750 0.403708
H23 -3.625316 0.150907 1.464140
N24 -3.684474 1.096922 -0.374983
H25 3.039494 -2.616636 0.021676

Zero-point correction= 0.200165 (Hartree/Particle)
Thermal correction to Energy= 0.212023
Thermal correction to Enthalpy= 0.212967
Thermal correction to Gibbs Free Energy= 0.162686
Sum of electronic and zero-point Energies= -676.437702
Sum of electronic and thermal Energies= -676.425844
Sum of electronic and thermal Enthalpies= -676.424899
Sum of electronic and thermal Free Energies= -676.475180

3-CH_2NH_2[9MG – H_{N2} + H_{O6}]$^+$

C1 1.326878 0.531112 0.036621
C2 -0.057752 0.570497 -0.124800
C3 -0.199058 -1.813737 -0.190453
C4 1.971477 -0.700642 0.065215
C5 0.838918 2.570918 0.069714
N6 -0.824923 -0.548532 -0.274373
N7 -0.372901 1.893827 -0.099816
N8 -0.718540 -2.964989 -0.225591
H9 -1.724251 -3.004552 -0.331763
N10 1.866241 1.788935 0.154639
N11 -3.021710 -0.673722 0.869206
H12 -2.876904 0.056182 1.552804
C13 -2.321385 -0.527359 -0.353074
H14 -2.600618 -1.320909 -1.047037
H15 -2.591027 0.405942 -0.839882
C16 -1.652282 2.589352 -0.235663
H17 -1.476111 3.645312 -0.033234
H18 -2.042986 2.492124 -1.249836
H19 -2.376940 2.222756 0.492152
H20 0.858361 3.651005 0.120367
N21 1.210483 -1.788550 -0.058807
H22 1.635228 -2.710895 -0.029674
O23 3.254710 -0.885438 0.202547
H24 -2.973853 -1.586102 1.301304
H25 3.720092 -0.036984 0.282275

Zero-point correction= 0.206253 (Hartree/Particle)
Thermal correction to Energy= 0.218659
Thermal correction to Enthalpy= 0.219603
Thermal correction to Gibbs Free Energy= 0.167612
Sum of electronic and zero-point Energies= -676.494079
Sum of electronic and thermal Energies= -676.481673
Sum of electronic and thermal Enthalpies= -676.480729
Sum of electronic and thermal Free Energies= -676.532720

TS for 3-CH_2NH_2[9MG – H_{N2} + H_{O6}]$^+$

C1 1.430413 0.450915 0.043883
C2 0.050572 0.567359 -0.121308
C3 -0.223461 -1.805341 -0.184963
C4 2.005155 -0.814872 0.075444
C5 1.057019 2.514819 0.073626
N6 -0.777485 -0.507264 -0.271832
N7 -0.190264 1.906248 -0.098494
N8 -0.806317 -2.925833 -0.220352
H9 -1.812385 -2.909307 -0.329195
N10 2.038851 1.676798 0.162100
N11 -3.441626 -0.476010 0.840401
H12 -3.258095 0.245454 1.523658
C13 -2.731003 -0.370377 -0.380133
H14 -3.052277 -1.147895 -1.074054
H15 -2.946830 0.575964 -0.868597
C16 -1.428452 2.671961 -0.238483
H17 -1.194132 3.716669 -0.036634
H18 -1.821301 2.595535 -1.253608
H19 -2.174364 2.347230 0.487742
H20 1.136603 3.592194 0.123253
N21 1.184936 -1.858738 -0.049543
H22 1.557442 -2.803324 -0.018345
O23 3.275702 -1.070867 0.216418
H24 -3.445924 -1.389143 1.273536
H25 3.787519 -0.249628 0.296554

Zero-point correction= 0.203422 (Hartree/Particle)
Thermal correction to Energy= 0.215527
Thermal correction to Enthalpy= 0.216472
Thermal correction to Gibbs Free Energy= 0.165697
Sum of electronic and zero-point Energies= -676.458599
Sum of electronic and thermal Energies= -676.446493
Sum of electronic and thermal Enthalpies= -676.445549
Sum of electronic and thermal Free Energies= -676.496324

5-CH_2NH_2[9MG – H_{N2} + H_{O6}]$^+$

C1 -0.178779 -0.842833 0.097845
C2 -0.675091 0.569029 0.012006
C3 1.388752 1.429895 -0.309723
C4 1.028517 -0.940923 -0.750102
C5 -2.300354 -0.896469 -0.292419
N6 0.032262 1.633036 -0.071604
N7 -2.034371 0.447330 -0.040193
N8 2.329947 2.262680 -0.279125
H9 2.001627 3.175757 0.030619
N10 -1.284997 -1.687477 -0.293665
N11 1.256968 -0.330860 2.100764
H12 0.949107 0.368787 2.761043
C13 0.228446 -1.188927 1.610956
H14 0.526637 -2.240533 1.608733
H15 -0.713740 -1.115372 2.163233
C16 -2.963747 1.564197 -0.166991
H17 -3.982035 1.177026 -0.143639
H18 -2.819530 2.249944 0.668230
H19 -2.788756 2.095287 -1.105340
H20 -3.319544 -1.215069 -0.482427
N21 1.775903 0.119440 -0.832707
H22 2.673376 0.098182 -1.312437
O23 1.378685 -2.044041 -1.335071
H24 2.076283 -0.806100 2.450730
H25 0.670650 -2.708425 -1.251901

Zero-point correction= 0.205051 (Hartree/Particle)
Thermal correction to Energy= 0.217687
Thermal correction to Enthalpy= 0.218631

Thermal correction to Gibbs Free Energy= 0.166563
Sum of electronic and zero-point Energies= -676.474866
Sum of electronic and thermal Energies= -676.462230
Sum of electronic and thermal Enthalpies= -676.461286
Sum of electronic and thermal Free Energies= -676.513354

TS for 5-$CH_2NH_2[9MG – H_{N2} + H_{O6}]^+$
C1 0.004622 -0.721890 -0.248479
C2 -0.918977 0.411273 0.060860
C3 0.696078 1.979041 0.065955
C4 1.165899 -0.205213 -0.970669
C5 -2.019159 -1.413494 -0.541019
N6 -0.623964 1.643099 0.292554
N7 -2.159709 -0.135234 0.022151
N8 1.333226 3.019685 0.383007
H9 0.737504 3.670205 0.892615
N10 -0.809195 -1.775748 -0.795942
N11 2.113139 -1.694208 1.684177
H12 2.782340 -1.011899 2.002532
C13 0.778702 -1.393567 1.591899
H14 0.126424 -2.300188 1.711682
H15 0.490716 -0.578732 2.270412
C16 -3.402867 0.567992 0.325410
H17 -4.223415 -0.148346 0.317607
H18 -3.323440 1.024534 1.312908
H19 -3.587571 1.346640 -0.418055
H20 -2.898476 -2.019039 -0.736614
N21 1.504151 1.035762 -0.714458
H22 2.379968 1.445122 -1.034284
O23 1.878563 -1.024143 -1.683383
H24 2.465719 -2.591266 1.391108
H25 2.692399 -0.642233 -2.047016

Zero-point correction= 0.201392 (Hartree/Particle)
Thermal correction to Energy= 0.213965
Thermal correction to Enthalpy= 0.214909
Thermal correction to Gibbs Free Energy= 0.163104
Sum of electronic and zero-point Energies= -676.439260
Sum of electronic and thermal Energies= -676.426687
Sum of electronic and thermal Enthalpies= -676.425743
Sum of electronic and thermal Free Energies= -676.477549

7-$CH_2NH_2[9MG – H_{N2} + H_{O6}]^+$
C1 0.134684 -0.418534 0.000002
C2 -0.539638 0.831688 0.000001
C3 -2.637107 -0.024999 -0.000005
C4 -0.676757 -1.540668 0.000004
C5 1.657333 1.167375 0.000006
N6 -1.802635 1.088133 -0.000001
N7 0.475628 1.793850 0.000005
N8 -3.901353 -0.057588 -0.000012
H9 -4.287399 0.882761 -0.000016
N10 1.510325 -0.146871 0.000005
N11 3.881652 -0.457955 -0.000019
H12 4.437370 -0.590118 0.832703
C13 2.633324 -1.132240 0.000005
H14 2.496742 -1.747909 -0.895352
H15 2.496769 -1.747883 0.895385
C16 0.231977 3.233459 0.000005
H17 1.189102 3.753455 0.000018
H18 -0.340559 3.498320 -0.889545
H19 -0.340580 3.498315 0.889542
H20 2.624305 1.648804 0.000007
N21 -1.991382 -1.311626 0.000001
H22 -2.627145 -2.103024 0.000001
O23 -0.340501 -2.822523 0.000009
H24 4.437332 -0.590108 -0.832769
H25 0.609525 -2.974520 0.000005

Zero-point correction= 0.204833 (Hartree/Particle)
Thermal correction to Energy= 0.218247
Thermal correction to Enthalpy= 0.219191
Thermal correction to Gibbs Free Energy= 0.163673
Sum of electronic and zero-point Energies= -676.485869
Sum of electronic and thermal Energies= -676.472455
Sum of electronic and thermal Enthalpies= -676.471510
Sum of electronic and thermal Free Energies= -676.527029

TS for 7-$CH_2NH_2[9MG – H_{N2} + H_{O6}]^+$
C1 -0.115107 -0.360690 -0.000002
C2 0.677385 0.818175 -0.000001
C3 2.681794 -0.238060 0.000005
C4 0.583581 -1.556288 -0.000004
C5 -1.476628 1.365528 -0.000006
N6 1.959310 0.950815 0.000001
N7 -0.239694 1.874341 -0.000005
N8 3.936907 -0.393211 0.000012
H9 4.412406 0.505226 0.000016
N10 -1.457883 0.043219 -0.000005
N11 -4.209110 -0.295506 0.000019
H12 -4.775032 -0.373103 -0.832703
C13 -3.032127 -1.087777 -0.000005
H14 -2.955950 -1.713797 0.895352
H15 -2.955975 -1.713768 -0.895385
C16 0.142544 3.283502 -0.000005
H17 -0.759587 3.893947 -0.000018
H18 0.738086 3.491539 0.889545
H19 0.738106 3.491532 -0.889542
H20 -2.392304 1.938544 -0.000007
N21 1.914230 -1.455934 -0.000001
H22 2.470173 -2.305306 -0.000001
O23 0.124488 -2.799451 -0.000009
H24 -4.774993 -0.373097 0.832769
H25 -0.835806 -2.858515 -0.000005

Zero-point correction= 0.201835 (Hartree/Particle)
Thermal correction to Energy= 0.214754
Thermal correction to Enthalpy= 0.215698
Thermal correction to Gibbs Free Energy= 0.162158
Sum of electronic and zero-point Energies= -676.447981
Sum of electronic and thermal Energies= -676.435062
Sum of electronic and thermal Enthalpies= -676.434118
Sum of electronic and thermal Free Energies= -676.487658

8-$CH_2NH_2[9MG – H_{N2} + H_{O6}]^+$
C1 -0.161415 0.748194 -0.407568
C2 -0.009818 -0.704545 -0.292168
C3 -2.191584 -1.035070 0.245427
C4 -1.490901 1.296407 -0.175690
C5 1.949097 0.384745 -0.722338
N6 -0.941942 -1.554038 0.011006
N7 1.272349 -0.907105 -0.606073
N8 -3.260913 -1.625256 0.564113
H9 -3.112705 -2.629864 0.643072
N10 0.898132 1.383110 -0.715204
N11 2.115654 0.544869 1.734596
H12 2.409063 -0.218667 2.328456
C13 2.873682 0.647762 0.510798
H14 3.321763 1.634696 0.372898
H15 3.682421 -0.087988 0.457781
C16 1.952769 -2.186839 -0.557284
H17 2.728194 -2.216728 -1.324399
H18 2.399913 -2.355103 0.427677
H19 1.222223 -2.972855 -0.750893
H20 2.529647 0.452839 -1.647982
N21 -2.402616 0.421026 0.128419
H22 -3.362629 0.709170 0.315432
O23 -1.779155 2.557418 -0.254136
H24 2.117645 1.397628 2.277351
H25 -0.997926 3.085366 -0.491367

Zero-point correction= 0.204958 (Hartree/Particle)
Thermal correction to Energy= 0.217729
Thermal correction to Enthalpy= 0.218673
Thermal correction to Gibbs Free Energy= 0.165819
Sum of electronic and zero-point Energies= -676.467894
Sum of electronic and thermal Energies= -676.455123
Sum of electronic and thermal Enthalpies= -676.454179
Sum of electronic and thermal Free Energies= -676.507034

TS for 8-CH$_2$NH$_2$[9MG – H$_{N2}$ + H$_{O6}$]$^+$
C1 0.082361 -0.735712 -0.485524
C2 -0.071311 0.684048 -0.343474
C3 2.080941 1.063039 0.253958
C4 1.387436 -1.246485 -0.298531
C5 -1.959997 -0.366244 -0.871069
N6 0.817963 1.565690 0.011472
N7 -1.382415 0.889129 -0.666841
N8 3.121867 1.671428 0.640580
H9 2.934989 2.664470 0.757244
N10 -1.043207 -1.346275 -0.866985
N11 -1.568644 -0.606003 1.942808
H12 -1.392553 0.318531 2.311232
C13 -2.592990 -0.855838 1.146476
H14 -2.858592 -1.888712 0.961858
H15 -3.370458 -0.102747 1.084563
C16 -2.060280 2.173681 -0.661975
H17 -2.816658 2.191452 -1.447344
H18 -2.529868 2.371103 0.307226
H19 -1.317805 2.948845 -0.854001
H20 -2.916511 -0.461234 -1.370250
N21 2.302960 -0.365857 0.050253
H22 3.267383 -0.657470 0.187416
O23 1.747703 -2.496251 -0.450026
H24 -0.929011 -1.335874 2.221020
H25 1.010839 -3.034068 -0.776923

Zero-point correction= 0.203100 (Hartree/Particle)
Thermal correction to Energy= 0.215670
Thermal correction to Enthalpy= 0.216614
Thermal correction to Gibbs Free Energy= 0.164446
Sum of electronic and zero-point Energies= -676.449864
Sum of electronic and thermal Energies= -676.437294
Sum of electronic and thermal Enthalpies= -676.436350
Sum of electronic and thermal Free Energies= -676.488518

9MG$^{•+}$···$^•$NHCH$_3$
C1 -1.067314 1.028551 -0.039378
C2 -1.482327 -0.345625 0.009063
C3 0.593572 -1.142320 -0.133333
C4 0.365056 1.312331 -0.148921
C5 -3.167181 1.028963 0.114871
N6 -0.734040 -1.420419 -0.030664
O7 0.909859 2.385041 -0.200589
N8 -2.849433 -0.292768 0.108827
N9 1.431914 -2.166803 -0.178142
H10 1.058824 -3.104093 -0.139562
N11 -2.112197 1.848478 0.026998
C12 -3.750676 -1.438458 0.189394
H13 -4.773018 -1.069922 0.263662
H14 -3.645087 -2.050152 -0.707231
H15 -3.509183 -2.031531 1.072095
H16 -4.192622 1.367968 0.185449
N17 1.108261 0.106879 -0.190971
H18 2.139585 0.180400 -0.280314
H19 2.431228 -2.009242 -0.252270
N20 3.968216 -0.210108 -0.374268
H21 4.502211 -0.163957 -1.246994
C22 4.816408 0.190787 0.709918
H23 4.297449 0.107015 1.665799
H24 5.740928 -0.404716 0.734389
H25 5.136528 1.235714 0.567541

Zero-point correction= 0.197896 (Hartree/Particle)
Thermal correction to Energy= 0.212687
Thermal correction to Enthalpy= 0.213632
Thermal correction to Gibbs Free Energy= 0.153065
Sum of electronic and zero-point Energies= -676.442218
Sum of electronic and thermal Energies= -676.427427
Sum of electronic and thermal Enthalpies= -676.426483
Sum of electronic and thermal Free Energies= -676.487049

TS for 9MG$^{•+}$···$^•$NHCH$_3$
C1 -1.607991 0.846867 -0.133385
C2 -1.211078 -0.508850 0.102617
C3 0.924029 0.000852 0.605196
C4 -0.604157 1.901314 0.033092
C5 -3.314532 -0.348612 -0.442827
N6 -0.031773 -0.969111 0.427397
O7 -0.743914 3.090673 -0.101892
N8 -2.356074 -1.246229 -0.111736
N9 2.136564 -0.309322 0.983893
H10 2.191870 -1.319507 1.108091
N11 -2.893764 0.922582 -0.466269
C12 -2.479326 -2.693816 0.012616
H13 -3.507584 -2.975203 -0.211575
H14 -1.803740 -3.180784 -0.691562
H15 -2.229757 -2.995854 1.030417
H16 -4.329077 -0.652878 -0.665356
N17 0.632749 1.335754 0.407309
H18 1.350747 2.007733 0.651314
H19 3.735116 0.401659 0.391922
N20 4.115471 0.086290 -0.507588
H21 3.752940 0.527651 -1.346049
C22 4.959318 -1.066063 -0.614128
H23 5.145019 -1.482119 0.374655
H24 4.510368 -1.819109 -1.271122
H25 5.915635 -0.766873 -1.065724

Zero-point correction= 0.197666 (Hartree/Particle)
Thermal correction to Energy= 0.211948
Thermal correction to Enthalpy= 0.212892
Thermal correction to Gibbs Free Energy= 0.154221
Sum of electronic and zero-point Energies= -676.415700
Sum of electronic and thermal Energies= -676.401419
Sum of electronic and thermal Enthalpies= -676.400475
Sum of electronic and thermal Free Energies= -676.459146

9MG$^{•+}$
N1 1.951089 -0.354606 -0.005142
C2 0.216432 1.034959 -0.000539
C3 -1.205061 1.373599 0.001928
O4 -1.725500 2.455145 0.004341
N5 -1.999347 0.182774 0.001639
H6 -2.998658 0.360703 0.003298
C7 -1.529623 -1.090371 -0.000790
N8 -2.379565 -2.107275 -0.002563
H9 -3.382593 -1.994376 -0.003061
H10 -1.998213 -3.043644 -0.004027
N11 -0.209722 -1.400170 -0.001375
C12 0.580659 -0.354446 -0.001519
C13 2.813092 -1.534798 0.008441
H14 2.706697 -2.055012 0.961059
H15 2.530406 -2.196941 -0.810123
H16 3.844856 -1.211354 -0.122566
C17 2.319038 0.954047 -0.004590
N18 1.291151 1.814582 -0.002565
H19 3.359041 1.254389 -0.006842

Zero-point correction= 0.146470 (Hartree/Particle)

Thermal correction to Energy= 0.156362
Thermal correction to Enthalpy= 0.157306
Thermal correction to Gibbs Free Energy= 0.110575
Sum of electronic and zero-point Energies= -581.292005
Sum of electronic and thermal Energies= -581.282114
Sum of electronic and thermal Enthalpies= -581.281170
Sum of electronic and thermal Free Energies= -581.327900

3-NHCH$_3$[9MG]$^+$
C1 -0.608904 1.338718 -0.122597
C2 0.397237 0.424687 0.101700
C3 -1.099484 -1.385030 0.282790
C4 -1.993909 0.927039 -0.135332
C5 1.186596 2.445236 -0.042610
N6 -2.094510 -0.500498 0.136537
N7 0.186835 -0.946967 0.279485
O8 -3.004715 1.547683 -0.301028
N9 1.561095 1.126944 0.167327
N10 -1.329437 -2.685775 0.415216
H11 -2.262238 -3.067054 0.403390
N12 -0.092888 2.600858 -0.216130
N13 1.171303 -1.934610 0.162396
H14 -0.530023 -3.294621 0.537039
C15 1.708227 -2.068676 -1.196280
H16 2.095207 -1.130730 -1.610928
H17 2.510420 -2.806932 -1.157674
H18 0.924399 -2.446974 -1.855800
H19 1.927634 3.232669 -0.050776
C20 2.904962 0.700059 0.540823
H21 3.302855 -0.028862 -0.166858
H22 3.550449 1.577632 0.520278
H23 2.911685 0.297355 1.556439
H24 1.875875 -1.796698 0.877040
H25 -3.053669 -0.829123 0.171296

Zero-point correction= 0.205981 (Hartree/Particle)
Thermal correction to Energy= 0.218354
Thermal correction to Enthalpy= 0.219299
Thermal correction to Gibbs Free Energy= 0.168133
Sum of electronic and zero-point Energies= -676.478574
Sum of electronic and thermal Energies= -676.466201
Sum of electronic and thermal Enthalpies= -676.465257
Sum of electronic and thermal Free Energies= -676.516422

TS for 3-NHCH$_3$[9MG]$^+$
C1 -1.538973 0.459090 0.223979
C2 -0.197509 0.596352 -0.048352
C3 0.160138 -1.726785 -0.244457
C4 -2.158983 -0.850529 0.164457
C5 -1.155762 2.536551 0.049411
N6 0.615965 -0.486910 -0.479273
O7 -3.304581 -1.196654 0.258971
N8 0.070071 1.928026 -0.181142
N9 0.963017 -2.803316 -0.337789
H10 0.613476 -3.735955 -0.126707
N11 -2.129326 1.697950 0.225848
N12 2.841172 -0.034794 -0.302436
H13 3.258478 -0.809528 -1.025531
C14 3.429025 -0.358780 0.974484
H15 2.966688 0.263802 1.748265
H16 3.419312 -1.412673 1.283643
H17 4.487302 -0.050059 0.870282
C18 1.329382 2.643078 -0.355743
H19 2.019845 2.421739 0.464280
H20 1.792786 2.383570 -1.304600
H21 1.098334 3.706369 -0.339387
H22 -1.236277 3.613896 0.079190
N23 -1.163561 -1.882427 -0.055576
H24 -1.559151 -2.818797 -0.085504
H25 1.990583 -2.712700 -0.305783

Zero-point correction= 0.196378 (Hartree/Particle)
Thermal correction to Energy= 0.209584
Thermal correction to Enthalpy= 0.210528
Thermal correction to Gibbs Free Energy= 0.156076
Sum of electronic and zero-point Energies= -676.374054
Sum of electronic and thermal Energies= -676.360848
Sum of electronic and thermal Enthalpies= -676.359903
Sum of electronic and thermal Free Energies= -676.414356

4-NHCH$_3$[9MG]$^+$
C1 0.058090 0.923896 -0.481269
C2 -0.519998 -0.285407 0.239090
C3 1.605862 -1.178806 -0.031269
C4 1.489828 1.258009 -0.199412
C5 -1.800149 0.580215 -1.448410
N6 0.323449 -1.403318 -0.043175
O7 1.964556 2.360998 -0.174744
N8 -1.783833 -0.388534 -0.601199
N9 2.469096 -2.206930 -0.071108
H10 3.437419 -2.083603 -0.320792
N11 -0.652184 1.381915 -1.446673
N12 -0.872311 -0.185497 1.600302
H13 -0.259094 -0.719585 2.199421
C14 -1.347171 1.091652 2.120233
H15 -2.244929 1.406503 1.578360
H16 -1.631596 0.956655 3.163700
H17 -0.602368 1.896407 2.061890
C18 -2.780142 -1.415801 -0.353590
H19 -2.311629 -2.387210 -0.524767
H20 -3.092105 -1.344773 0.689655
H21 -3.628184 -1.273468 -1.022252
H22 -2.613087 0.759836 -2.143340
N23 2.192064 0.088607 0.060063
H24 3.175346 0.192114 0.282354
H25 2.081891 -3.137114 -0.125971

Zero-point correction= 0.202666 (Hartree/Particle)
Thermal correction to Energy= 0.216018
Thermal correction to Enthalpy= 0.216962
Thermal correction to Gibbs Free Energy= 0.163569
Sum of electronic and zero-point Energies= -676.467425
Sum of electronic and thermal Energies= -676.454073
Sum of electronic and thermal Enthalpies= -676.453129
Sum of electronic and thermal Free Energies= -676.506522

TS for 4-NHCH$_3$[9MG]$^+$
C1 -0.005314 0.836677 -0.671320
C2 -0.343148 -0.462752 -0.147237
C3 1.812785 -1.025992 0.100121
C4 1.336696 1.356035 -0.357107
C5 -1.883727 0.347617 -1.478658
N6 0.557406 -1.442291 -0.014474
O7 1.707802 2.499631 -0.357740
N8 -1.599212 -0.702030 -0.710028
N9 2.771279 -1.930747 0.313770
H10 3.749477 -1.727946 0.175396
N11 -0.918874 1.294101 -1.504228
N12 -1.070558 0.002012 1.941891
H13 -0.204607 0.009776 2.496242
C14 -1.770042 1.242091 2.156261
H15 -2.722126 1.240462 1.620204
H16 -1.972574 1.357669 3.231709
H17 -1.182446 2.124038 1.859966
C18 -2.431511 -1.851913 -0.385750
H19 -1.928575 -2.764218 -0.709498
H20 -2.566087 -1.870391 0.697821
H21 -3.391385 -1.751294 -0.890274
H22 -2.821338 0.441878 -2.011170
N23 2.183906 0.295507 0.035256

H24 3.124409 0.576150 0.290459
H25 2.490779 -2.899612 0.367902

Zero-point correction=	0.198125 (Hartree/Particle)
Thermal correction to Energy=	0.211852
Thermal correction to Enthalpy=	0.212796
Thermal correction to Gibbs Free Energy=	0.157232
Sum of electronic and zero-point Energies=	-676.408977
Sum of electronic and thermal Energies=	-676.395250
Sum of electronic and thermal Enthalpies=	-676.394306
Sum of electronic and thermal Free Energies=	-676.449870

5-NHCH$_3$[9MG]$^+$

C1 -0.085711 -0.793920 0.014785
C2 -0.739918 0.567605 0.149659
C3 1.205499 1.644891 0.087432
C4 1.130034 -0.599816 -0.901995
C5 -2.205458 -0.973521 -0.448477
N6 -0.109380 1.692332 0.363848
O7 1.576594 -1.379821 -1.689013
N8 -2.040426 0.386422 -0.040744
N9 1.950310 2.683646 0.429017
H10 1.500678 3.459495 0.894980
N11 -1.140536 -1.664445 -0.492349
N12 0.452912 -1.185525 1.304679
H13 -0.285380 -1.131270 2.000333
C14 1.077182 -2.518327 1.341688
H15 1.996088 -2.517183 0.752054
H16 1.345659 -2.720811 2.379048
H17 0.419829 -3.310663 0.971030
H18 -3.200530 -1.326297 -0.697485
C19 -3.086506 1.402361 -0.011329
H20 -2.646627 2.335480 0.338604
H21 -3.495675 1.543110 -1.013673
H22 -3.876885 1.090267 0.672846
N23 1.776692 0.627041 -0.621870
H24 2.947775 2.718333 0.282353
H25 2.684577 0.746174 -1.056649

Zero-point correction=	0.204303 (Hartree/Particle)
Thermal correction to Energy=	0.217274
Thermal correction to Enthalpy=	0.218219
Thermal correction to Gibbs Free Energy=	0.165152
Sum of electronic and zero-point Energies=	-676.494266
Sum of electronic and thermal Energies=	-676.481295
Sum of electronic and thermal Enthalpies=	-676.480351
Sum of electronic and thermal Free Energies=	-676.533418

TS for 5-NHCH$_3$[9MG]$^+$

C1 0.292338 -0.803782 -0.496596
C2 0.762290 0.496476 -0.057875
C3 -1.229302 1.475419 -0.199123
C4 -1.059633 -0.853703 -1.054180
C5 2.399714 -0.890854 -0.477917
N6 0.051742 1.586572 0.168948
O7 -1.616261 -1.768698 -1.602083
N8 2.101900 0.381496 -0.004611
N9 -2.032087 2.519406 0.017672
H10 -3.003769 2.531744 -0.241038
N11 1.364792 -1.605133 -0.809508
N12 -0.190032 -1.494728 1.824896
H13 -0.124231 -2.490886 1.538425
C14 -1.554999 -1.204372 2.167426
H15 -1.689494 -0.136576 2.398208
H16 -2.311949 -1.589712 1.455182
H17 -1.741565 -1.775160 3.099857
C18 3.062349 1.413808 0.387972
H19 2.496338 2.288275 0.712388
H20 3.678937 1.053856 1.213771
H21 3.690695 1.681223 -0.464467
H22 3.429295 -1.223897 -0.549432
N23 -1.753751 0.368052 -0.801676
H24 -2.705918 0.375146 -1.150681
H25 -1.622730 3.347962 0.416160

Zero-point correction=	0.198054 (Hartree/Particle)
Thermal correction to Energy=	0.211370
Thermal correction to Enthalpy=	0.212314
Thermal correction to Gibbs Free Energy=	0.158445
Sum of electronic and zero-point Energies=	-676.423708
Sum of electronic and thermal Energies=	-676.410392
Sum of electronic and thermal Enthalpies=	-676.409448
Sum of electronic and thermal Free Energies=	-676.463317

7-NHCH$_3$[9MG]$^+$

C1 -0.329760 -0.261046 -0.138025
C2 0.523188 0.812438 -0.010554
C3 2.364272 -0.424430 0.093464
C4 0.191342 -1.598910 -0.190790
C5 -1.538453 1.574463 -0.200729
N6 1.856109 0.795463 0.113155
O7 -0.409356 -2.642934 -0.336143
N8 -0.259114 1.952475 -0.046097
N9 3.690093 -0.582011 0.212324
H10 4.143222 -1.480656 0.211715
N11 -1.613382 0.251434 -0.262158
N12 -2.834798 -0.420608 -0.376383
H13 -2.748863 -1.058520 -1.163622
C14 -3.177850 -1.150793 0.853283
H15 -3.301982 -0.433476 1.666545
H16 -4.138495 -1.634200 0.672254
H17 -2.436613 -1.911025 1.120604
C18 0.249564 3.319485 0.059403
H19 0.723038 3.452505 1.032316
H20 0.981622 3.489263 -0.730165
H21 -0.582151 4.014238 -0.048522
H22 -2.393969 2.229977 -0.265069
N23 1.595858 -1.557727 -0.046896
H24 2.035300 -2.470925 -0.074498
H25 4.256552 0.245871 0.313663

Zero-point correction=	0.205489 (Hartree/Particle)
Thermal correction to Energy=	0.218438
Thermal correction to Enthalpy=	0.219382
Thermal correction to Gibbs Free Energy=	0.166775
Sum of electronic and zero-point Energies=	-676.508701
Sum of electronic and thermal Energies=	-676.495752
Sum of electronic and thermal Enthalpies=	-676.494807
Sum of electronic and thermal Free Energies=	-676.547415

TS for 7-NHCH$_3$[9MG]$^+$

C1 -0.287514 0.007185 -0.119668
C2 0.893376 0.716288 0.001818
C3 2.186153 -1.087827 0.058769
C4 -0.258419 -1.419457 -0.174843
C5 -0.813320 2.102037 -0.131268
N6 2.141383 0.227109 0.102528
O7 -1.198460 -2.195543 -0.292625
N8 0.541445 2.045149 -0.001816
N9 3.375498 -1.710948 0.147313
H10 3.479621 -2.710488 0.124333
N11 -1.329059 0.895869 -0.201907
N12 -3.592180 0.127943 -0.350405
H13 -3.423247 -0.418731 -1.216501
C14 -3.736713 -0.750539 0.725863
H15 -3.762328 -0.224462 1.690677
H16 -4.793286 -1.077965 0.494009
H17 -3.103586 -1.659561 0.692774
C18 1.466882 3.166627 0.108329
H19 1.979767 3.126366 1.067919

H20 2.199008 3.112665 -0.699864
H21 0.901821 4.096656 0.031290
H22 -1.370384 3.026012 -0.173183
N23 1.065872 -1.882811 -0.069559
H24 1.157831 -2.890542 -0.105187
H25 4.199082 -1.137683 0.237658

Zero-point correction=	0.197724 (Hartree/Particle)
Thermal correction to Energy=	0.211285
Thermal correction to Enthalpy=	0.212229
Thermal correction to Gibbs Free Energy=	0.157628
Sum of electronic and zero-point Energies=	-676.411893
Sum of electronic and thermal Energies=	-676.398332
Sum of electronic and thermal Enthalpies=	-676.397388
Sum of electronic and thermal Free Energies=	-676.451989

8-NHCH3[9MG]+
C1 -0.283925 0.900585 -0.130945
C2 -0.199136 -0.576352 -0.100888
C3 -2.421212 -0.824584 0.095289
C4 -1.631603 1.524496 -0.027305
C5 1.864085 0.432052 -0.344062
N6 -2.632124 0.533292 0.079792
N7 -1.224434 -1.408920 0.010096
O8 -1.894512 2.692589 -0.028603
N9 1.072622 -0.861997 -0.200912
N10 -3.485411 -1.611082 0.202390
H11 -3.334512 -2.609921 0.210924
N12 0.842799 1.477215 -0.240843
N13 2.929963 0.609806 0.529391
H14 2.698575 0.705033 1.507200
C15 4.268759 0.141884 0.204645
H16 4.491486 0.380468 -0.838965
H17 4.986637 0.682442 0.822862
H18 4.417959 -0.933945 0.359142
H19 2.230493 0.447831 -1.380210
C20 1.679437 -2.180365 -0.191213
H21 0.893391 -2.929278 -0.097354
H22 2.229205 -2.340125 -1.121425
H23 2.364769 -2.255701 0.655540
H24 -3.574637 0.902630 0.144146
H25 -4.429610 -1.264641 0.274439

Zero-point correction=	0.203719 (Hartree/Particle)
Thermal correction to Energy=	0.217069
Thermal correction to Enthalpy=	0.218013
Thermal correction to Gibbs Free Energy=	0.163933
Sum of electronic and zero-point Energies=	-676.499022
Sum of electronic and thermal Energies=	-676.485673
Sum of electronic and thermal Enthalpies=	-676.484728
Sum of electronic and thermal Free Energies=	-676.538808

TS for 8-NHCH3[9MG]+
C1 -0.322036 0.899119 -0.284912
C2 -0.227946 -0.558255 -0.232880
C3 -2.400000 -0.833211 0.218629
C4 -1.645847 1.519880 -0.054215
C5 1.747098 0.411271 -0.618365
N6 -1.207186 -1.408010 0.018536
O7 -1.929961 2.683355 -0.054888
N8 1.050528 -0.832301 -0.461861
N9 -3.432378 -1.630232 0.464828
H10 -4.369351 -1.294612 0.626081
N11 0.811326 1.455422 -0.519576
N12 3.071803 0.807893 0.893792
H13 2.578480 0.218420 1.590777
C14 4.288771 0.079628 0.553174
H15 4.760804 0.532015 -0.321817
H16 4.959679 0.242648 1.411514
H17 4.190373 -1.005474 0.416394
C18 1.660052 -2.153724 -0.466605
H19 2.282211 -2.283775 0.420874
H20 0.865647 -2.899458 -0.460250
H21 2.268147 -2.274360 -1.365579
H22 2.417605 0.471887 -1.474244
N23 -2.621848 0.518486 0.186684
H24 -3.553304 0.885209 0.346829
H25 -3.266872 -2.626392 0.482738

Zero-point correction=	0.200575 (Hartree/Particle)
Thermal correction to Energy=	0.213402
Thermal correction to Enthalpy=	0.214346
Thermal correction to Gibbs Free Energy=	0.161589
Sum of electronic and zero-point Energies=	-676.424729
Sum of electronic and thermal Energies=	-676.411902
Sum of electronic and thermal Enthalpies=	-676.410958
Sum of electronic and thermal Free Energies=	-676.463714

[9MG – HN2 + HN3]•+···•NHCH3
C1 1.741845 0.344205 0.065101
C2 0.339680 0.574488 -0.074144
C3 -0.157078 -1.735364 -0.155210
C4 2.253104 -1.040425 0.101052
C5 1.450213 2.432888 0.065635
N6 -0.569882 -0.381237 -0.177967
O7 3.404288 -1.368928 0.213947
N8 0.180717 1.924733 -0.069116
N9 -0.953812 -2.720031 -0.252505
H10 -1.927649 -2.446999 -0.357882
N11 2.395607 1.502114 0.148219
N12 -3.307678 -0.098009 -0.381650
H13 -3.825509 0.039481 -1.254208
C14 -4.225803 -0.307885 0.698101
H15 -3.699579 -0.422181 1.646738
H16 -4.947945 0.519176 0.768599
H17 -4.830776 -1.209614 0.510327
C18 -1.068594 2.669865 -0.192316
H19 -1.502395 2.512222 -1.181477
H20 -1.767364 2.348965 0.582214
H21 -0.853475 3.729331 -0.059038
H22 1.629173 3.499758 0.100589
N23 1.203661 -1.957479 -0.015376
H24 1.468176 -2.937512 -0.003437
H25 -1.617455 -0.198483 -0.274552

Zero-point correction=	0.196978 (Hartree/Particle)
Thermal correction to Energy=	0.211451
Thermal correction to Enthalpy=	0.212395
Thermal correction to Gibbs Free Energy=	0.153633
Sum of electronic and zero-point Energies=	-676.406566
Sum of electronic and thermal Energies=	-676.392093
Sum of electronic and thermal Enthalpies=	-676.391149
Sum of electronic and thermal Free Energies=	-676.449911

TS for [9MG – HN2 + HN3]•+···•NHCH3
C1 1.711404 0.116251 0.112010
C2 0.387301 0.575103 -0.158741
C3 -0.468295 -1.562155 -0.391952
C4 1.968813 -1.331579 0.179437
C5 1.785708 2.222590 0.144860
N6 -0.679656 -0.170020 -0.376832
O7 3.022643 -1.865064 0.415862
N8 0.472337 1.942846 -0.125090
N9 -1.382935 -2.412686 -0.671610
H10 -2.254952 -1.973710 -0.954226
N11 2.544138 1.143671 0.296482
C12 -0.602472 2.892191 -0.364091
H13 -1.055671 2.702781 -1.339695
H14 -1.352543 2.822836 0.427728
H15 -0.186623 3.899305 -0.362414

H16 2.144213 3.241104 0.219229
N17 0.794426 -2.043237 -0.076266
H18 0.882952 -3.053607 -0.101542
H19 -2.037086 0.201464 -0.292851
N20 -3.193712 0.352305 -0.089438
H21 -3.789407 0.914754 -0.698845
C22 -3.835286 -0.278704 1.012269
H23 -3.112139 -0.824127 1.617554
H24 -4.375126 0.466874 1.614928
H25 -4.609994 -0.969498 0.639761

Zero-point correction=	0.193387 (Hartree/Particle)
Thermal correction to Energy=	0.207188
Thermal correction to Enthalpy=	0.208133
Thermal correction to Gibbs Free Energy=	0.151638
Sum of electronic and zero-point Energies=	-676.403400
Sum of electronic and thermal Energies=	-676.389599
Sum of electronic and thermal Enthalpies=	-676.388655
Sum of electronic and thermal Free Energies=	-676.445150

[9MG – H_{N2} + H_{N3}]$^{•+}$

C1 -0.153405 1.015130 -0.000034
C2 -0.603634 -0.342754 0.000003
C3 1.635988 -1.194748 0.000060
C4 1.295628 1.309989 -0.000024
C5 -2.262493 1.048845 -0.000063
N6 0.219785 -1.385655 0.000047
O7 1.789922 2.404285 -0.000052
N8 -1.956944 -0.295171 -0.000017
N9 2.486577 -2.129033 0.000101
H10 2.113252 -3.074168 0.000126
N11 -1.194266 1.841524 -0.000074
C12 -2.891135 -1.421322 0.000006
H13 -2.749656 -2.023567 -0.899695
H14 -2.749685 -2.023507 0.899751
H15 -3.905606 -1.024945 -0.000024
H16 -3.288968 1.392891 -0.000088
N17 2.045275 0.128614 0.000023
H18 3.054251 0.246700 0.000033
H19 -0.121653 -2.340480 0.000072

Zero-point correction=	0.145327 (Hartree/Particle)
Thermal correction to Energy=	0.155307
Thermal correction to Enthalpy=	0.156251
Thermal correction to Gibbs Free Energy=	0.109067
Sum of electronic and zero-point Energies=	-581.251276
Sum of electronic and thermal Energies=	-581.241297
Sum of electronic and thermal Enthalpies=	-581.240353
Sum of electronic and thermal Free Energies=	-581.287536

2-$NHCH_3$[9MG – H_{N2} + H_{N3}]$^+$

C1 1.610680 0.663885 0.068459
C2 0.879485 -0.496683 -0.048373
C3 -1.167307 0.614812 -0.205500
C4 0.950782 1.947981 0.052389
C5 2.995496 -0.934294 0.134907
N6 -0.497222 1.767562 -0.095796
N7 -0.496729 -0.544757 -0.182154
O8 1.372255 3.065626 0.135122
N9 1.751981 -1.529890 -0.007434
N10 -2.501087 0.583997 -0.354969
H11 -3.041899 1.440078 -0.291231
N12 2.941678 0.366395 0.183013
N13 -3.133878 -0.660948 -0.383996
H14 -1.057676 -1.388127 -0.267606
C15 -4.053807 -0.856686 0.743071
H16 -4.832857 -0.086595 0.795483
H17 -4.528240 -1.830916 0.616755
H18 -3.486558 -0.861934 1.675770
H19 3.893803 -1.533147 0.194671
C20 1.457360 -2.952709 -0.102090
H21 0.989250 -3.182184 -1.062228
H22 2.396036 -3.501287 -0.034267
H23 0.812158 -3.265666 0.722232
H24 -3.608627 -0.756485 -1.277226
H25 -1.002766 2.646905 -0.121137

Zero-point correction=	0.205303 (Hartree/Particle)
Thermal correction to Energy=	0.218162
Thermal correction to Enthalpy=	0.219106
Thermal correction to Gibbs Free Energy=	0.165861
Sum of electronic and zero-point Energies=	-676.482849
Sum of electronic and thermal Energies=	-676.469990
Sum of electronic and thermal Enthalpies=	-676.469046
Sum of electronic and thermal Free Energies=	-676.522291

TS for 2-$NHCH_3$[9MG – H_{N2} + H_{N3}]$^+$

C1 1.196568 1.003833 -0.000130
C2 1.314462 -0.394596 -0.015791
C3 -0.971446 -0.699265 -0.505019
C4 -0.113667 1.634519 -0.190096
C5 3.234403 0.547960 0.318519
N6 0.268732 -1.234272 -0.192414
O7 -0.385614 2.802394 -0.135788
N8 2.626536 -0.680493 0.185001
N9 -1.989227 -1.425331 -0.885431
H10 -1.878706 -2.421736 -0.694138
N11 2.402262 1.564140 0.211987
N12 -3.787634 -0.001767 -0.064296
H13 -4.337547 -0.309084 -0.871349
C14 -4.385818 -0.495502 1.141402
H15 -3.696264 -0.397332 1.982786
H16 -5.259710 0.143022 1.358321
H17 -4.761389 -1.522955 1.064506
C18 3.254398 -1.995748 0.256320
H19 4.320384 -1.857091 0.431992
H20 2.836227 -2.569924 1.086038
H21 3.124196 -2.527542 -0.688815
H22 4.298635 0.629719 0.495321
N23 -1.097655 0.650067 -0.453844
H24 -2.087988 0.938647 -0.473013
H25 0.392568 -2.238499 -0.243591

Zero-point correction=	0.195932 (Hartree/Particle)
Thermal correction to Energy=	0.209789
Thermal correction to Enthalpy=	0.210733
Thermal correction to Gibbs Free Energy=	0.154312
Sum of electronic and zero-point Energies=	-676.381177
Sum of electronic and thermal Energies=	-676.367319
Sum of electronic and thermal Enthalpies=	-676.366375
Sum of electronic and thermal Free Energies=	-676.422797

4-$NHCH_3$[9MG – H_{N2} + H_{N3}]$^+$

C1 -0.230665 1.211684 0.125251
C2 0.551714 -0.094334 0.160369
C3 -1.489946 -1.097142 -0.740695
C4 -1.712159 1.129294 0.345154
C5 1.706188 1.743941 -0.558935
N6 -0.098218 -0.867115 -0.862393
O7 -2.358343 2.012519 0.848112
N8 1.854240 0.466584 -0.412127
N9 -2.138477 -2.082408 -1.188838
H10 -1.573928 -2.788055 -1.653456
N11 0.434913 2.230679 -0.282199
N12 0.670817 -0.711283 1.408888
H13 0.962591 -0.119250 2.173925
C14 0.925970 -2.141980 1.546009
H15 0.107686 -2.716157 1.104743
H16 1.877257 -2.471919 1.110510
H17 0.944255 -2.372949 2.610735

C18 3.078872 -0.304554 -0.582082
H19 3.873649 0.352285 -0.935025
H20 3.362777 -0.738956 0.378101
H21 2.914158 -1.095797 -1.316266
H22 2.501486 2.400884 -0.896259
N23 -2.194854 -0.082955 -0.079126
H24 -3.194347 -0.241102 -0.002600
H25 0.412368 -1.665098 -1.219148

Zero-point correction= 0.202759 (Hartree/Particle)
Thermal correction to Energy= 0.215980
Thermal correction to Enthalpy= 0.216925
Thermal correction to Gibbs Free Energy= 0.163446
Sum of electronic and zero-point Energies= -676.437613
Sum of electronic and thermal Energies= -676.424391
Sum of electronic and thermal Enthalpies= -676.423447
Sum of electronic and thermal Free Energies= -676.476926

TS for 4-NHCH$_3$[9MG – H$_{N2}$ + H$_{N3}$]$^+$
C1 -0.021154 -0.806379 -0.661926
C2 0.289665 0.540300 -0.285541
C3 -2.005272 1.047205 0.135581
C4 -1.371256 -1.338176 -0.392118
C5 1.955049 -0.422020 -1.294672
N6 -0.655093 1.441937 -0.038116
O7 -1.692331 -2.496434 -0.442172
N8 1.570549 0.750614 -0.714964
N9 -2.962400 1.819526 0.428048
N10 1.015038 -1.365471 -1.288535
N11 0.971273 -0.095649 1.916144
H12 0.108297 -0.316461 2.445609
C13 1.893868 -1.207193 2.071652
H14 2.887981 -0.951434 1.642678
H15 1.999566 -1.448052 3.147817
H16 1.503302 -2.138472 1.583063
C17 2.385779 1.930217 -0.449683
H18 1.970311 2.800428 -0.961083
H19 2.418291 2.095290 0.630743
H20 3.390919 1.756618 -0.823847
H21 2.950527 -0.556787 -1.699674
N22 -2.240664 -0.315642 -0.015810
H23 -3.194997 -0.597377 0.199523
H24 -2.711329 2.795991 0.545100
H25 -0.435220 2.420801 0.080305

Zero-point correction= 0.197379 (Hartree/Particle)
Thermal correction to Energy= 0.210687
Thermal correction to Enthalpy= 0.211631
Thermal correction to Gibbs Free Energy= 0.157238
Sum of electronic and zero-point Energies= -676.387105
Sum of electronic and thermal Energies= -676.373797
Sum of electronic and thermal Enthalpies= -676.372853
Sum of electronic and thermal Free Energies= -676.427246

5-NHCH$_3$[9MG – H$_{N2}$ + H$_{N3}$]$^+$
C1 0.019690 -0.784791 0.060547
C2 -0.837536 0.455379 0.155347
C3 1.121920 1.812280 0.043254
C4 1.147733 -0.470990 -0.954318
C5 -2.068775 -1.302121 -0.338443
N6 -0.286893 1.640155 0.320382
O7 1.580959 -1.252568 -1.752953
N8 -2.095278 0.108644 -0.005982
N9 1.804287 2.815954 0.360869
H10 1.315407 3.524606 0.900563
N11 -0.917294 -1.821535 -0.359542
N12 0.642964 -1.013692 1.343515
H13 -0.077118 -1.220529 2.029766
C14 1.670057 -2.069980 1.365529
H15 2.571285 -1.720615 0.858221
H16 1.925053 -2.249861 2.410018
H17 1.335100 -3.004039 0.903921
H18 -3.010948 -1.796968 -0.546601
C19 -3.268408 0.978098 -0.011705
H20 -3.400639 1.437949 0.969891
H21 -3.164439 1.744103 -0.784628
H22 -4.146471 0.372957 -0.234414
N23 1.651440 0.802206 -0.754680
H24 2.540165 1.023475 -1.190800
H25 -0.837717 2.480082 0.454494

Zero-point correction= 0.203937 (Hartree/Particle)
Thermal correction to Energy= 0.216694
Thermal correction to Enthalpy= 0.217638
Thermal correction to Gibbs Free Energy= 0.165267
Sum of electronic and zero-point Energies= -676.459064
Sum of electronic and thermal Energies= -676.446307
Sum of electronic and thermal Enthalpies= -676.445362
Sum of electronic and thermal Free Energies= -676.497734

TS for 5-NHCH$_3$[9MG – H$_{N2}$ + H$_{N3}$]$^+$
C1 0.023470 -0.670253 0.438590
C2 0.999160 0.253061 -0.049966
C3 -0.570162 2.060430 -0.043856
C4 -1.176522 -0.101369 1.079760
C5 1.895203 -1.669594 0.455081
N6 0.722337 1.525698 -0.336903
O7 -1.940129 -0.706148 1.793391
N8 2.174277 -0.393809 -0.065450
N9 -0.992989 3.190135 -0.415902
H10 -0.338781 3.734457 -0.968968
N11 0.654768 -1.847096 0.772316
N12 -1.529541 -0.925014 -1.701822
H13 -0.876711 -1.239649 -2.435908
C14 -2.375254 -1.987853 -1.337584
H15 -2.976511 -1.748827 -0.450163
H16 -3.077819 -2.014693 -2.218902
H17 -1.911720 -2.990211 -1.292765
H18 2.685884 -2.404111 0.546819
C19 3.480926 0.141744 -0.434905
H20 3.411238 0.659809 -1.396865
H21 3.848448 0.822118 0.340741
H22 4.179330 -0.690835 -0.543688
N23 -1.357193 1.239237 0.749001
H24 -2.220519 1.666660 1.065941
H25 1.435667 2.173414 -0.644773

Zero-point correction= 0.197402 (Hartree/Particle)
Thermal correction to Energy= 0.210670
Thermal correction to Enthalpy= 0.211615
Thermal correction to Gibbs Free Energy= 0.157340
Sum of electronic and zero-point Energies= -676.363162
Sum of electronic and thermal Energies= -676.349894
Sum of electronic and thermal Enthalpies= -676.348949
Sum of electronic and thermal Free Energies= -676.403224

7-NHCH$_3$[9MG – H$_{N2}$ + H$_{N3}$]$^+$
C1 0.312793 -0.239998 -0.122505
C2 -0.542852 0.819554 0.006170
C3 -2.482787 -0.594332 0.059274
C4 -0.179269 -1.619260 -0.143010
C5 1.497569 1.616301 -0.130638
N6 -1.563843 -1.641342 -0.068059
N7 -1.890225 0.690838 0.106334
O8 0.498460 -2.619805 -0.205149
N9 0.194430 1.971779 -0.013771
N10 -3.714462 -0.860672 0.125078
H11 -4.325046 -0.054655 0.214844
N12 1.584558 0.295584 -0.212719
N13 2.732202 -0.449644 -0.462476

H14 3.429448 0.172824 -0.856072
C15 3.214636 -1.194993 0.707056
H16 2.489807 -1.971988 0.951459
H17 4.140291 -1.683100 0.401131
H18 3.405354 -0.550455 1.573459
H19 2.326206 2.306277 -0.170389
C20 -0.324891 3.335201 0.082281
H21 -0.814802 3.476865 1.047495
H22 0.505232 4.035291 0.001134
H23 -1.023805 3.520305 -0.735683
H24 -1.992535 -2.560651 -0.084178
H25 -2.497653 1.487082 0.225513

Zero-point correction= 0.204779 (Hartree/Particle)
Thermal correction to Energy= 0.217719
Thermal correction to Enthalpy= 0.218663
Thermal correction to Gibbs Free Energy= 0.165908
Sum of electronic and zero-point Energies= -676.462317
Sum of electronic and thermal Energies= -676.449377
Sum of electronic and thermal Enthalpies= -676.448433
Sum of electronic and thermal Free Energies= -676.501188

TS for 7-NHCH$_3$[9MG – H$_{N2}$ + H$_{N3}$]$^+$
C1 -0.292211 -0.038353 -0.082247
C2 0.857962 0.705471 0.009716
C3 2.247381 -1.183258 0.065308
C4 -0.231287 -1.478190 -0.154848
C5 -0.880377 2.045681 -0.120936
N6 2.109971 0.189777 0.138061
O7 -1.186593 -2.226835 -0.294832
N8 0.483008 2.022156 -0.007166
N9 3.358186 -1.815284 0.118640
N10 -1.360011 0.821143 -0.160604
N11 -3.393614 0.184087 -0.355123
H12 -3.015187 -0.347189 -1.158658
C13 -3.670538 -0.737434 0.678351
H14 -3.888629 -0.240900 1.623235
H15 -4.641534 -1.108234 0.261464
H16 -3.003867 -1.605288 0.742458
C17 1.383413 3.170084 0.078066
H18 1.917650 3.144385 1.029559
H19 2.093826 3.147622 -0.751670
H20 0.796038 4.084890 0.018018
H21 -1.456523 2.957014 -0.160961
N22 1.072561 -1.931346 -0.046998
H23 1.197104 -2.936807 -0.090345
H24 4.185084 -1.240447 0.231979
H25 2.932010 0.761895 -0.034561

Zero-point correction= 0.199856 (Hartree/Particle)
Thermal correction to Energy= 0.212675
Thermal correction to Enthalpy= 0.213619
Thermal correction to Gibbs Free Energy= 0.160882
Sum of electronic and zero-point Energies= -676.394541
Sum of electronic and thermal Energies= -676.381722
Sum of electronic and thermal Enthalpies= -676.380778
Sum of electronic and thermal Free Energies= -676.433515

8-NHCH$_3$[9MG – H$_{N2}$ + H$_{N3}$]$^+$
C1 0.112086 -0.759602 -0.249102
C2 0.230910 0.704183 -0.141269
C3 2.616038 0.539001 0.225602
C4 1.338855 -1.608372 -0.129745
C5 -1.910333 0.063767 -0.489596
N6 2.471817 -0.839578 0.093013
N7 1.401094 1.290165 0.090223
O8 1.347515 -2.805983 -0.216233
N9 -0.958652 1.207599 -0.317586
N10 3.750280 1.033022 0.446420
H11 3.789651 2.044403 0.528420
N12 -1.089760 -1.124504 -0.438490
N13 -2.872824 0.069873 0.571266
H14 -3.464969 0.890373 0.481198
C15 -3.721911 -1.133562 0.624375
H16 -3.117902 -1.994936 0.907773
H17 -4.474682 -0.970369 1.395619
H18 -4.224940 -1.348623 -0.327078
H19 -2.320088 0.132804 -1.514232
C20 -1.374263 2.600938 -0.255792
H21 -0.561934 3.252499 -0.583172
H22 -2.212932 2.747006 -0.937934
H23 -1.674632 2.857119 0.763430
H24 3.344746 -1.349686 0.188999
H25 1.475596 2.293123 0.206082

Zero-point correction= 0.203973 (Hartree/Particle)
Thermal correction to Energy= 0.217021
Thermal correction to Enthalpy= 0.217965
Thermal correction to Gibbs Free Energy= 0.163894
Sum of electronic and zero-point Energies= -676.463496
Sum of electronic and thermal Energies= -676.450448
Sum of electronic and thermal Enthalpies= -676.449503
Sum of electronic and thermal Free Energies= -676.503575

TS for 8-NHCH$_3$[9MG – H$_{N2}$ + H$_{N3}$]$^+$
C1 -0.347092 0.901867 -0.213266
C2 -0.250446 -0.549593 -0.098654
C3 -2.464489 -0.824264 0.159225
C4 -1.680352 1.527694 -0.019785
C5 1.700396 0.398606 -0.640355
N6 -1.211626 -1.387994 0.266611
O7 -1.956151 2.690729 -0.019206
N8 1.016779 -0.839152 -0.393191
N9 -3.524374 -1.631002 0.274298
N10 0.768797 1.452262 -0.545128
N11 3.074214 0.835321 0.824510
H12 2.621080 0.226354 1.535000
C13 4.295130 0.145470 0.427625
H14 4.721058 0.620748 -0.459491
H15 4.996172 0.316930 1.260152
H16 4.227422 -0.941156 0.276290
C17 1.628174 -2.154811 -0.409852
H18 2.486237 -2.177276 0.319274
H19 0.850230 -2.900517 -0.076703
H20 1.985298 -2.387366 -1.445980
H21 2.335152 0.427479 -1.525832
N22 -2.663382 0.532009 0.191388
H23 -3.619531 0.893384 0.229186
H24 -3.342111 -2.616351 0.395110
H25 -1.122572 -2.387977 0.087609

Zero-point correction= 0.199347 (Hartree/Particle)
Thermal correction to Energy= 0.211303
Thermal correction to Enthalpy= 0.212248
Thermal correction to Gibbs Free Energy= 0.161511
Sum of electronic and zero-point Energies= -676.361475
Sum of electronic and thermal Energies= -676.349518
Sum of electronic and thermal Enthalpies= -676.348574
Sum of electronic and thermal Free Energies= -676.399311

[9MG – H$_{N2}$ + H$_{O6}$]$^{•+}$···$^•$NHCH$_3$
C1 -0.634427 -0.839051 -0.073422
C2 -1.622566 0.174950 0.021648
C3 -0.154710 1.881883 -0.047373
C4 0.727189 -0.420552 -0.161961
C5 -2.485423 -1.829751 0.034968
N6 -1.455089 1.479218 0.038806
N7 -2.810883 -0.509312 0.089128
N8 0.255638 3.103868 -0.050616
H9 -0.539870 3.738527 0.020828

N10 -1.184473 -2.067771 -0.063698
N11 4.091448 -0.426696 -0.416242
H12 4.568130 -0.266770 -1.306819
C13 5.007950 -0.437546 0.679887
H14 4.486736 -0.592444 1.624357
H15 5.750498 -1.238374 0.536028
H16 5.583035 0.499747 0.712477
C17 -4.136363 0.087463 0.210113
H18 -4.882300 -0.700659 0.114749
H19 -4.236184 0.573523 1.181571
H20 -4.276317 0.822384 -0.583105
H21 -3.243445 -2.601014 0.069503
N22 0.885252 0.916079 -0.144071
H23 1.812678 1.323005 -0.202769
O24 1.699257 -1.236766 -0.249373
H25 2.699825 -0.835866 -0.328133

Zero-point correction=	0.196206 (Hartree/Particle)
Thermal correction to Energy=	0.210215
Thermal correction to Enthalpy=	0.211159
Thermal correction to Gibbs Free Energy=	0.153823
Sum of electronic and zero-point Energies=	-676.422326
Sum of electronic and thermal Energies=	-676.408317
Sum of electronic and thermal Enthalpies=	-676.407373
Sum of electronic and thermal Free Energies=	-676.464709

TS for [9MG – H_{N2} + H_{O6}]$^{•+}$···$^{•}$NHCH$_3$

C1 0.587752 -0.813784 -0.075089
C2 1.637500 0.135640 0.022292
C3 0.279121 1.931956 -0.015275
C4 -0.749826 -0.312327 -0.149484
C5 2.375095 -1.918469 0.006503
N6 1.553126 1.447495 0.054651
N7 2.782081 -0.621666 0.072003
N8 -0.051474 3.177347 -0.001705
H9 0.784291 3.757773 0.069220
N10 1.060829 -2.073997 -0.083844
N11 -4.131073 -0.298705 -0.406131
H12 -4.574879 0.067836 -1.250788
C13 -5.070751 -0.602414 0.624317
H14 -4.564520 -0.979826 1.512092
H15 -5.669359 0.286518 0.872832
H16 -5.788548 -1.354327 0.260116
C17 4.141944 -0.107952 0.188182
H18 4.838090 -0.940845 0.096463
H19 4.326707 0.613449 -0.608318
H20 4.272462 0.375729 1.157202
H21 3.084842 -2.734923 0.026348
N22 -0.819598 1.034161 -0.112241
H23 -1.718855 1.500746 -0.152627
O24 -1.767954 -1.060671 -0.240455
H25 -2.808864 -0.635106 -0.326705

Zero-point correction=	0.194219 (Hartree/Particle)
Thermal correction to Energy=	0.207774
Thermal correction to Enthalpy=	0.208718
Thermal correction to Gibbs Free Energy=	0.152411
Sum of electronic and zero-point Energies=	-676.424254
Sum of electronic and thermal Energies=	-676.410698
Sum of electronic and thermal Enthalpies=	-676.409754
Sum of electronic and thermal Free Energies=	-676.466062

2-NHCH$_3$[9MG – H_{N2} + H_{O6}]$^{+}$

C1 -1.564261 -0.611678 0.062463
C2 -0.802181 0.566163 -0.062595
C3 1.150780 -0.486397 -0.212541
C4 -0.866632 -1.794801 0.037723
C5 -2.949798 0.965684 0.131082
N6 0.484663 -1.701197 -0.101720
N7 0.522608 0.666312 -0.190550
O8 -1.333910 -3.016922 0.127797
N9 -1.709209 1.570769 -0.016972
N10 2.490754 -0.574175 -0.366461
H11 2.953883 -1.457710 -0.173352
N12 -2.907987 -0.334793 0.182616
N13 3.252569 0.584601 -0.431602
C14 4.039908 0.826005 0.772334
H15 4.709749 -0.006292 1.033467
H16 4.642011 1.720260 0.603105
H17 3.364471 1.021739 1.608131
H18 -3.851643 1.560274 0.193142
C19 -1.430269 3.000645 -0.100305
H20 -0.364905 3.124118 -0.293408
H21 -2.001703 3.440434 -0.918591
H22 -1.688860 3.486576 0.841674
H23 3.812511 0.558198 -1.275598
H24 0.995862 -2.575675 -0.156239
H25 -2.299167 -3.010888 0.219145

Zero-point correction=	0.205020 (Hartree/Particle)
Thermal correction to Energy=	0.218102
Thermal correction to Enthalpy=	0.219046
Thermal correction to Gibbs Free Energy=	0.163141
Sum of electronic and zero-point Energies=	-676.484656
Sum of electronic and thermal Energies=	-676.471574
Sum of electronic and thermal Enthalpies=	-676.470630
Sum of electronic and thermal Free Energies=	-676.526535

TS for 2-NHCH$_3$[9MG – H_{N2} + H_{O6}]$^{+}$

C1 -1.706287 -0.504601 0.123450
C2 -0.822630 0.588518 -0.021577
C3 1.015137 -0.655314 -0.166979
C4 -1.129771 -1.748569 0.077538
C5 -2.931115 1.202660 0.133590
N6 0.228020 -1.790067 -0.021954
N7 0.499456 0.556322 -0.164365
O8 -1.690180 -2.951110 0.110139
N9 -1.630742 1.681452 -0.003320
N10 2.327474 -0.865456 -0.370928
H11 2.718569 -1.788237 -0.186798
N12 -3.018416 -0.095341 0.208851
N13 3.523175 0.673044 -0.385991
C14 4.574593 0.627218 0.662892
H15 5.374481 -0.235342 0.567368
H16 5.044753 1.653482 0.558730
H17 4.076140 0.557826 1.718705
H18 -3.766304 1.887235 0.172897
C19 -1.211243 3.074929 -0.127779
H20 -0.130174 3.082730 -0.308931
H21 -1.720442 3.537862 -0.976625
H22 -1.431267 3.623661 0.786681
H23 3.834614 0.837020 -1.404464
H24 0.640914 -2.722690 0.024012
H25 -1.354705 -3.453390 -0.755542

Zero-point correction=	0.196951 (Hartree/Particle)
Thermal correction to Energy=	0.208686
Thermal correction to Enthalpy=	0.209630
Thermal correction to Gibbs Free Energy=	0.159501
Sum of electronic and zero-point Energies=	-676.368154
Sum of electronic and thermal Energies=	-676.356419
Sum of electronic and thermal Enthalpies=	-676.355475
Sum of electronic and thermal Free Energies=	-676.405605

3-NHCH$_3$[9MG – H_{N2} + H_{O6}]$^{+}$

C1 1.014254 -0.979299 0.085558
C2 -0.277779 -0.485867 -0.113903
C3 0.497105 1.781359 -0.278605
C4 2.078988 -0.078168 0.093872
C5 -0.240817 -2.667649 0.061392

N6 -0.561825 0.840188 -0.290933
N7 -1.087071 -1.567485 -0.136044
N8 0.421692 3.031015 -0.391009
H9 -0.538000 3.342991 -0.523033
N10 1.004652 -2.351549 0.194680
N11 -1.832663 1.399848 -0.225939
H12 -2.426635 1.049091 -0.965144
C13 -2.416028 1.428638 1.112917
H14 -1.814365 2.080924 1.749842
H15 -3.413122 1.863137 1.027324
H16 -2.490177 0.442117 1.589395
C17 -2.506619 -1.679901 -0.463608
H18 -2.792885 -2.723418 -0.337057
H19 -3.115750 -1.074998 0.207581
H20 -2.680756 -1.396625 -1.503632
H21 -0.648847 -3.668830 0.092280
N22 1.787152 1.217836 -0.099943
H23 2.506697 1.933725 -0.094813
O24 3.302241 -0.493628 0.271211
H25 3.977742 0.197261 0.246150

Zero-point correction= 0.205359 (Hartree/Particle)
Thermal correction to Energy= 0.217740
Thermal correction to Enthalpy= 0.218684
Thermal correction to Gibbs Free Energy= 0.167395
Sum of electronic and zero-point Energies= -676.449348
Sum of electronic and thermal Energies= -676.436967
Sum of electronic and thermal Enthalpies= -676.436023
Sum of electronic and thermal Free Energies= -676.487312

TS for 3-NHCH$_3$[9MG – H$_{N2}$ + H$_{O6}$]$^+$
C1 -1.307460 0.708701 0.105634
C2 0.059419 0.548664 -0.121684
C3 -0.119492 -1.835136 -0.293351
C4 -2.100019 -0.437551 0.119669
C5 -0.500117 2.653823 0.074150
N6 0.617410 -0.650308 -0.341194
N7 0.588724 1.794103 -0.140345
N8 0.292829 -3.020555 -0.405126
H9 1.294017 -3.064267 -0.570038
N10 -1.628254 2.042942 0.219161
N11 2.605147 -0.987815 -0.268397
H12 3.020275 -0.205002 -0.783463
C13 3.019701 -0.832849 1.108035
H14 2.560493 -1.616831 1.716964
H15 4.113331 -0.981654 1.113371
H16 2.808535 0.144839 1.555316
C17 1.934465 2.257640 -0.487964
H18 1.949477 3.339133 -0.372425
H19 2.680598 1.843833 0.192166
H20 2.166088 2.021403 -1.526786
H21 -0.347880 3.725166 0.112415
N22 -1.503055 -1.626688 -0.086372
H23 -2.023202 -2.496009 -0.063152
O24 -3.386202 -0.345324 0.308541
H25 -3.860705 -1.189526 0.286286

Zero-point correction= 0.200043 (Hartree/Particle)
Thermal correction to Energy= 0.212964
Thermal correction to Enthalpy= 0.213908
Thermal correction to Gibbs Free Energy= 0.160705
Sum of electronic and zero-point Energies= -676.374859
Sum of electronic and thermal Energies= -676.361938
Sum of electronic and thermal Enthalpies= -676.360994
Sum of electronic and thermal Free Energies= -676.414197

5-NHCH$_3$[9MG – H$_{N2}$ + H$_{O6}$]$^+$
C1 0.066895 -0.713555 0.005772
C2 -0.820751 0.521097 0.134211
C3 0.916629 1.953099 0.188088
C4 1.295516 -0.271289 -0.762160
C5 -1.955894 -1.229869 -0.584444
N6 -0.441395 1.729449 0.329065
N7 -2.080441 0.069807 -0.071395
N8 1.607650 2.948040 0.525028
H9 1.047720 3.635696 1.027012
N10 -0.772696 -1.714626 -0.634689
N11 0.587228 -1.079293 1.310937
H12 -0.098774 -0.880638 2.031403
C13 1.112072 -2.445811 1.440156
H14 1.929277 -2.601106 0.732916
H15 1.518761 -2.545517 2.447107
H16 0.354736 -3.215654 1.267538
H17 -2.846798 -1.754348 -0.914007
C18 -3.274018 0.906542 -0.077585
H19 -3.383244 1.391874 0.892824
H20 -3.197206 1.668205 -0.856898
H21 -4.141627 0.273938 -0.263286
N22 1.670378 0.966098 -0.592241
H23 2.564622 1.325345 -0.918808
O24 1.933223 -1.152260 -1.461883
H25 2.748981 -0.837330 -1.881898

Zero-point correction= 0.204161 (Hartree/Particle)
Thermal correction to Energy= 0.216899
Thermal correction to Enthalpy= 0.217843
Thermal correction to Gibbs Free Energy= 0.165433
Sum of electronic and zero-point Energies= -676.444374
Sum of electronic and thermal Energies= -676.431636
Sum of electronic and thermal Enthalpies= -676.430691
Sum of electronic and thermal Free Energies= -676.483102

TS for 5-NHCH$_3$[9MG – H$_{N2}$ + H$_{O6}$]$^+$
C1 -0.114520 -0.517970 -0.548308
C2 -0.646867 0.733968 -0.074917
C3 1.375453 1.698775 0.213257
C4 1.300732 -0.563706 -0.742755
C5 -2.202195 -0.660193 -0.734603
N6 0.003326 1.788895 0.311909
O7 1.846994 -1.610151 -1.281692
N8 -2.009273 0.566993 -0.164190
N9 2.229690 2.565742 0.582110
H10 1.768202 3.379240 0.986934
N11 -1.097530 -1.331135 -1.002717
N12 0.584826 -1.448452 1.456268
H13 0.133051 -0.792203 2.104349
C14 -0.099336 -2.710337 1.535958
H15 0.243227 -3.383377 0.748466
H16 0.153443 -3.158034 2.510637
H17 -1.193964 -2.626369 1.502412
H18 -3.198151 -1.029955 -0.944298
C19 -3.012211 1.576636 0.158506
H20 -2.828042 1.962101 1.161471
H21 -2.961375 2.394510 -0.562114
H22 -3.996953 1.112196 0.121019
N23 1.961736 0.527755 -0.391574
H24 2.975731 0.577830 -0.432497
H25 2.813103 -1.586349 -1.328314

Zero-point correction= 0.198314 (Hartree/Particle)
Thermal correction to Energy= 0.211725
Thermal correction to Enthalpy= 0.212669
Thermal correction to Gibbs Free Energy= 0.157944
Sum of electronic and zero-point Energies= -676.390565
Sum of electronic and thermal Energies= -676.377155
Sum of electronic and thermal Enthalpies= -676.376210
Sum of electronic and thermal Free Energies= -676.430936

7-NHCH$_3$[9MG – H$_{N2}$ + H$_{O6}$]$^+$
C1 0.239326 -0.307714 -0.150398

C2 -0.586863 0.830657 -0.017846
C3 -2.533773 -0.311736 0.119182
C4 -0.387234 -1.542080 -0.141706
C5 1.533985 1.472139 -0.198935
N6 -1.718674 -1.503270 -0.010258
N7 -1.870816 0.906009 0.111785
O8 0.282443 -2.671109 -0.243543
N9 0.282630 1.927190 -0.061426
N10 -3.773206 -0.540718 0.227589
H11 -4.297192 0.325601 0.316726
N12 1.553783 0.146803 -0.268982
N13 2.660294 -0.684335 -0.426147
H14 3.230347 -0.332607 -1.187386
C15 3.401054 -0.903792 0.818076
H16 2.748143 -1.404264 1.535268
H17 4.227713 -1.575681 0.584227
H18 3.796730 0.018461 1.261654
H19 2.415648 2.094955 -0.257846
C20 -0.158504 3.316943 0.030878
H21 -0.866880 3.518738 -0.773144
H22 -0.650600 3.470305 0.991851
H23 0.707384 3.972059 -0.056860
H24 -2.275814 -2.351189 0.010996
H25 -0.281057 -3.455762 -0.240574

Zero-point correction=	0.204060 (Hartree/Particle)
Thermal correction to Energy=	0.217136
Thermal correction to Enthalpy=	0.218081
Thermal correction to Gibbs Free Energy=	0.164301
Sum of electronic and zero-point Energies=	-676.442363
Sum of electronic and thermal Energies=	-676.429287
Sum of electronic and thermal Enthalpies=	-676.428342
Sum of electronic and thermal Free Energies=	-676.482122

TS for 7-NHCH$_3$[9MG – H$_{N2}$ + H$_{O6}$]$^+$

C1 0.194278 -0.229748 -0.133411
C2 -0.778180 0.787424 -0.007971
C3 -2.553519 -0.606511 0.122268
C4 -0.265104 -1.537337 -0.129152
C5 1.242125 1.703188 -0.189384
N6 -1.589887 -1.677821 0.000210
N7 -2.060843 0.689511 0.114451
O8 0.550918 -2.571244 -0.229114
N9 -0.059430 1.991002 -0.051055
N10 -3.750958 -0.995074 0.225083
H11 -4.387189 -0.206917 0.308957
N12 1.435628 0.392254 -0.262475
N13 3.122783 -0.589734 -0.438330
H14 3.601143 -0.288643 -1.299702
C15 3.949093 -0.591585 0.767204
H16 3.339128 -0.980922 1.624847
H17 4.743584 -1.346442 0.554599
H18 4.400607 0.400994 1.041008
H19 2.031850 2.438935 -0.250300
C20 -0.684173 3.308994 0.033370
H21 -1.406581 3.414368 -0.772737
H22 -1.194478 3.399363 0.992833
H23 0.090635 4.074489 -0.051572
H24 -2.027624 -2.593058 0.020103
H25 0.093403 -3.419725 -0.227869

Zero-point correction=	0.198837 (Hartree/Particle)
Thermal correction to Energy=	0.211483
Thermal correction to Enthalpy=	0.212427
Thermal correction to Gibbs Free Energy=	0.160250
Sum of electronic and zero-point Energies=	-676.366131
Sum of electronic and thermal Energies=	-676.353486
Sum of electronic and thermal Enthalpies=	-676.352541
Sum of electronic and thermal Free Energies=	-676.404718

8-NHCH$_3$[9MG – H$_{N2}$ + H$_{O6}$]$^+$

C1 -0.164041 0.704183 -0.208576
C2 -0.269064 -0.764141 -0.108089
C3 -2.519398 -0.745845 0.187066
C4 -1.411312 1.460469 -0.080149
C5 1.878347 -0.038755 -0.429468
N6 -2.477936 0.732855 0.102287
N7 -1.353226 -1.452564 0.077313
N8 0.982510 -1.188343 -0.267554
N9 -3.702478 -1.146925 0.362930
H10 -3.742739 -2.162822 0.424007
N11 1.011172 1.148515 -0.385817
N12 2.831929 0.040866 0.637390
H13 3.251623 -0.869928 0.789814
C14 3.866876 1.058498 0.431622
H15 3.412708 2.050049 0.458480
H16 4.582047 0.990669 1.251536
H17 4.404031 0.940060 -0.520271
H18 2.319950 -0.060129 -1.444526
C19 1.415729 -2.574418 -0.254777
H20 0.552897 -3.207314 -0.464733
H21 2.171321 -2.725894 -1.028443
H22 1.822738 -2.844240 0.723832
H23 -3.409105 1.134259 0.206242
O24 -1.370940 2.751663 -0.155963
H25 -2.224569 3.201216 -0.059856

Zero-point correction=	0.203960 (Hartree/Particle)
Thermal correction to Energy=	0.216791
Thermal correction to Enthalpy=	0.217736
Thermal correction to Gibbs Free Energy=	0.164725
Sum of electronic and zero-point Energies=	-676.442261
Sum of electronic and thermal Energies=	-676.429430
Sum of electronic and thermal Enthalpies=	-676.428485
Sum of electronic and thermal Free Energies=	-676.481496

TS for 8-NHCH$_3$[9MG – H$_{N2}$ + H$_{O6}$]$^+$

C1 0.150895 -0.709669 -0.565505
C2 0.011292 0.704566 -0.357958
C3 2.135566 1.002475 0.342378
C4 1.423177 -1.266657 -0.290327
C5 -1.843969 -0.235271 -1.053529
N6 2.342781 -0.407320 0.138584
N7 0.906949 1.545748 0.072240
O8 1.640204 -2.541216 -0.458413
N9 -1.285448 0.963614 -0.719257
N10 3.174968 1.607811 0.773113
H11 2.985999 2.601392 0.900767
N12 -0.978884 -1.265554 -1.009183
N13 -2.919445 -0.767742 0.903149
H14 -3.243028 -1.734108 0.785301
C15 -2.012180 -0.721782 2.014227
H16 -1.558076 0.269548 2.102218
H17 -2.573201 -0.915844 2.941516
H18 -1.229892 -1.494130 1.965047
H19 -2.820855 -0.294983 -1.508322
C20 -1.988043 2.235072 -0.595338
H21 -1.258758 3.001307 -0.335515
H22 -2.460758 2.487835 -1.544851
H23 -2.743060 2.151746 0.189353
H24 3.291208 -0.697802 0.362117
H25 2.541920 -2.833534 -0.264539

Zero-point correction=	0.198107 (Hartree/Particle)
Thermal correction to Energy=	0.211462
Thermal correction to Enthalpy=	0.212406
Thermal correction to Gibbs Free Energy=	0.158071
Sum of electronic and zero-point Energies=	-676.389172
Sum of electronic and thermal Energies=	-676.375817
Sum of electronic and thermal Enthalpies=	-676.374873

Sum of electronic and thermal Free Energies= -676.429208

[9MG – H_{N2} + H_{N7}]$^{•+}$···$^{•}$NHCH$_3$

C1 -0.139854 -0.004282 0.012636
C2 1.092593 0.629683 -0.001669
C3 2.292003 -1.281061 -0.006021
C4 -0.213640 -1.457499 0.017817
C5 -0.501114 2.136932 0.004235
N6 1.072557 -1.985625 0.009001
N7 2.297959 0.077906 -0.011298
O8 -1.221364 -2.133727 0.027866
N9 0.827531 1.992662 -0.007503
N10 3.457013 -1.871589 -0.015894
H11 3.380872 -2.890842 -0.011302
N12 -1.112854 0.951305 0.016069
H13 -1.007858 3.090912 0.003398
C14 1.844384 3.045184 -0.007478
H15 2.542092 2.861025 -0.824114
H16 1.353657 4.007734 -0.144134
H17 2.380663 3.026790 0.941606
H18 1.106630 -2.998930 0.012503
H19 -2.212193 0.783433 0.024983
N20 -3.732970 0.514121 0.031512
C21 -4.300154 -0.798413 -0.052451
H22 -5.030716 -0.959631 0.753242
H23 -4.866011 -0.892693 -0.993654
H24 -3.518185 -1.556594 -0.019427
H25 -4.467999 1.223893 0.078357

Zero-point correction= 0.196140 (Hartree/Particle)
Thermal correction to Energy= 0.210251
Thermal correction to Enthalpy= 0.211196
Thermal correction to Gibbs Free Energy= 0.154163
Sum of electronic and zero-point Energies= -676.421852
Sum of electronic and thermal Energies= -676.407741
Sum of electronic and thermal Enthalpies= -676.406796
Sum of electronic and thermal Free Energies= -676.463829

TS for [9MG – H_{N2} + H_{N7}]$^{•+}$···$^{•}$NHCH$_3$

C1 -0.154179 0.007077 -0.017157
C2 1.087835 0.629255 -0.000291
C3 2.272732 -1.292438 0.004958
C4 -0.233621 -1.444593 -0.021150
C5 -0.502668 2.134873 -0.015326
N6 1.044261 -1.985611 -0.009908
N7 2.287580 0.069198 0.011015
O8 -1.247873 -2.116438 -0.031477
N9 0.829802 1.991470 -0.000229
N10 3.428720 -1.893071 0.013713
H11 3.342347 -2.911190 0.007755
N12 -1.130533 0.958359 -0.026487
H13 -0.996185 3.096203 -0.018668
C14 1.847755 3.040409 0.032133
H15 2.560092 2.868521 -0.774665
H16 1.361404 4.006091 -0.099023
H17 2.368482 3.008947 0.989610
H18 1.069353 -2.998974 -0.012263
H19 -2.360235 0.756825 -0.043347
N20 -3.659884 0.509916 -0.057173
C21 -4.221715 -0.794490 0.087255
H22 -4.768366 -0.843074 1.044300
H23 -4.971539 -0.979662 -0.694156
H24 -3.435548 -1.548663 0.071765
H25 -4.363273 1.244098 -0.158535

Zero-point correction= 0.193253 (Hartree/Particle)
Thermal correction to Energy= 0.206956
Thermal correction to Enthalpy= 0.207901
Thermal correction to Gibbs Free Energy= 0.152196
Sum of electronic and zero-point Energies= -676.424104
Sum of electronic and thermal Energies= -676.410401
Sum of electronic and thermal Enthalpies= -676.409457
Sum of electronic and thermal Free Energies= -676.465162

[9MG – H_{N2} + H_{N7}]$^{•+}$

C1 -0.101894 0.935621 -0.000020
C2 -0.529128 -0.374740 0.000000
C3 1.553762 -1.204386 0.000045
C4 1.314840 1.275451 -0.000009
C5 -2.297999 0.934828 -0.000051
N6 0.232418 -1.470708 0.000033
O7 1.781716 2.390512 -0.000026
N8 -1.919695 -0.342640 -0.000020
N9 2.467665 -2.160317 0.000077
H10 1.999639 -3.069486 0.000089
N11 -1.218566 1.725003 -0.000052
C12 -2.786482 -1.525162 -0.000002
H13 -2.577847 -2.115699 -0.891993
H14 -2.577973 -2.115584 0.892096
H15 -3.825434 -1.199298 -0.000097
H16 -3.321389 1.280433 -0.000072
N17 2.050874 0.094655 0.000025
H18 3.062984 0.184576 0.000037
H19 -1.221177 2.739342 -0.000074

Zero-point correction= 0.146106 (Hartree/Particle)
Thermal correction to Energy= 0.155753
Thermal correction to Enthalpy= 0.156697
Thermal correction to Gibbs Free Energy= 0.110215
Sum of electronic and zero-point Energies= -581.272348
Sum of electronic and thermal Energies= -581.262702
Sum of electronic and thermal Enthalpies= -581.261758
Sum of electronic and thermal Free Energies= -581.308239

2-NHCH$_3$[9MG – H_{N2} + H_{N7}]$^{+}$

C1 1.526726 0.665387 0.062732
C2 0.805370 -0.498560 -0.059472
C3 -1.159267 0.504221 -0.219483
C4 0.860126 1.940020 0.044052
C5 2.946643 -1.008751 0.130584
N6 -0.526615 1.727364 -0.106979
N7 -0.516426 -0.646648 -0.194651
O8 1.346211 3.045326 0.134321
N9 1.723788 -1.537000 -0.013025
N10 -2.499776 0.554583 -0.374319
H11 -2.977249 1.438534 -0.226144
N12 2.854450 0.316519 0.178699
N13 -3.244373 -0.616197 -0.418090
C14 -4.042061 -0.833767 0.783331
H15 -4.734879 -0.009800 1.008572
H16 -4.619619 -1.748565 0.639580
H17 -3.371746 -0.981886 1.632948
H18 3.863655 -1.574133 0.196343
C19 1.378134 -2.954476 -0.117824
H20 0.896987 -3.131579 -1.079736
H21 2.288612 -3.546587 -0.037329
H22 0.688650 -3.212164 0.685947
H23 -3.797700 -0.618550 -1.266872
H24 -1.065040 2.585267 -0.156009
H25 3.627289 0.962073 0.283160

Zero-point correction= 0.205705 (Hartree/Particle)
Thermal correction to Energy= 0.218606
Thermal correction to Enthalpy= 0.219550
Thermal correction to Gibbs Free Energy= 0.165645
Sum of electronic and zero-point Energies= -676.495958
Sum of electronic and thermal Energies= -676.483057
Sum of electronic and thermal Enthalpies= -676.482113
Sum of electronic and thermal Free Energies= -676.536018

TS for 2-NHCH$_3$[9MG – H$_{N2}$ + H$_{N7}$]$^+$
C1 -1.687925 -0.481716 0.063904
C2 -0.784253 0.544980 -0.061906
C3 0.947195 -0.800442 -0.190837
C4 -1.260261 -1.861273 0.058881
C5 -2.810017 1.403947 0.125716
N6 0.121939 -1.884753 -0.074365
N7 0.548318 0.452848 -0.198868
O8 -1.927297 -2.863215 0.157231
N9 -1.518744 1.717791 -0.017284
N10 2.208610 -1.194884 -0.475979
H11 2.469739 -2.052295 0.258396
N12 -2.941937 0.081801 0.183139
N13 3.834281 0.659522 -0.475701
C14 4.519398 0.608628 0.783975
H15 5.010078 -0.357944 0.984725
H16 5.320379 1.370384 0.798341
H17 3.822267 0.830205 1.597960
H18 -3.623952 2.114806 0.187990
C19 -0.949941 3.060525 -0.132271
H20 -0.448028 3.156373 -1.095682
H21 -1.753280 3.791819 -0.055448
H22 -0.226652 3.209560 0.669279
H23 4.538150 0.412051 -1.219720
H24 0.505355 -2.817996 -0.147630
H25 -3.808133 -0.425410 0.292575

Zero-point correction=	0.197240 (Hartree/Particle)
Thermal correction to Energy=	0.210411
Thermal correction to Enthalpy=	0.211355
Thermal correction to Gibbs Free Energy=	0.156520
Sum of electronic and zero-point Energies=	-676.368957
Sum of electronic and thermal Energies=	-676.355786
Sum of electronic and thermal Enthalpies=	-676.354842
Sum of electronic and thermal Free Energies=	-676.409677

3-NHCH$_3$[9MG – H$_{N2}$ + H$_{N7}$]$^+$
C1 -0.952365 0.976104 0.108258
C2 0.281030 0.440047 -0.122964
C3 -0.661639 -1.769093 -0.308098
C4 -2.181414 0.188242 0.115869
C5 0.484949 2.630657 0.039384
N6 0.484163 -0.896111 -0.353786
N7 1.177263 1.492373 -0.174001
N8 -0.618940 -3.022345 -0.402916
H9 0.331858 -3.360581 -0.532595
N10 -0.794919 2.335127 0.201196
N11 1.704264 -1.524614 -0.165486
H12 2.362418 -1.323094 -0.903573
C13 2.205836 -1.553450 1.201012
H14 1.524369 -2.138912 1.822833
H15 3.172811 -2.058749 1.190633
H16 2.328071 -0.559300 1.656470
C17 2.604309 1.461989 -0.512390
H18 2.984064 2.482359 -0.481786
H19 3.151662 0.858407 0.210112
H20 2.729820 1.067062 -1.521228
H21 0.919552 3.617609 0.073150
N22 -1.903158 -1.135244 -0.141217
H23 -2.689080 -1.776923 -0.142216
O24 -3.286378 0.651211 0.297803
H25 -1.549456 2.991157 0.372824

Zero-point correction=	0.205888 (Hartree/Particle)
Thermal correction to Energy=	0.218299
Thermal correction to Enthalpy=	0.219243
Thermal correction to Gibbs Free Energy=	0.167659
Sum of electronic and zero-point Energies=	-676.462250
Sum of electronic and thermal Energies=	-676.449839
Sum of electronic and thermal Enthalpies=	-676.448895
Sum of electronic and thermal Free Energies=	-676.500479

TS for 3-NHCH$_3$[9MG – H$_{N2}$ + H$_{N7}$]$^+$
C1 -1.340431 0.534275 0.137277
C2 -0.002662 0.515635 -0.131260
C3 -0.033580 -1.886546 -0.318243
C4 -2.173903 -0.662203 0.156767
C5 -0.651306 2.615410 0.057697
N6 0.689298 -0.638991 -0.392924
N7 0.420579 1.827245 -0.193126
N8 0.487277 -3.026208 -0.413997
H9 1.490809 -2.976134 -0.556835
N10 -1.712635 1.848826 0.246582
N11 2.600586 -0.798223 -0.154485
H12 3.115679 -0.476162 -0.977575
C13 3.139484 -0.542645 1.167068
H14 2.604063 -1.188949 1.911547
H15 4.203774 -0.894630 1.128673
H16 3.088702 0.531491 1.515633
C17 1.733540 2.349513 -0.585028
H18 1.700413 3.437413 -0.526679
H19 2.500721 1.981115 0.092894
H20 1.954242 2.051011 -1.610058
H21 -0.625906 3.693675 0.097478
N22 -1.418298 -1.776164 -0.128074
H23 -1.900042 -2.671522 -0.112937
O24 -3.371274 -0.656415 0.366975
H25 -2.656773 2.167977 0.448560

Zero-point correction=	0.198995 (Hartree/Particle)
Thermal correction to Energy=	0.211653
Thermal correction to Enthalpy=	0.212598
Thermal correction to Gibbs Free Energy=	0.159997
Sum of electronic and zero-point Energies=	-676.381473
Sum of electronic and thermal Energies=	-676.368814
Sum of electronic and thermal Enthalpies=	-676.367870
Sum of electronic and thermal Free Energies=	-676.420470

5-NHCH$_3$[9MG – H$_{N2}$ + H$_{N7}$]$^+$
C1 0.153169 -0.704582 0.066764
C2 -0.802850 0.461693 0.165741
C3 0.893502 1.955962 0.070603
C4 1.284400 -0.287751 -0.891611
C5 -2.002116 -1.291893 -0.455249
N6 -0.492452 1.679826 0.244849
O7 1.808999 -1.070060 -1.650190
N8 -2.106345 -0.045935 0.012747
N9 1.468109 3.013737 0.439367
H10 0.817077 3.647241 0.899748
N11 -0.764988 -1.718955 -0.523618
N12 0.702287 -1.028767 1.335697
H13 -0.004373 -1.103282 2.058087
C14 1.703011 -2.096513 1.409441
H15 2.586576 -1.824769 0.830023
H16 2.003117 -2.197313 2.452368
H17 1.334177 -3.068128 1.056108
H18 -2.865540 -1.875755 -0.753845
C19 -3.314403 0.777856 0.081937
H20 -3.372973 1.236229 1.068894
H21 -3.263717 1.556721 -0.680371
H22 -4.184816 0.143666 -0.082607
N23 1.620967 1.017102 -0.699278
H24 2.474726 1.361157 -1.124561
H25 -0.477564 -2.622982 -0.876416

Zero-point correction=	0.204276 (Hartree/Particle)
Thermal correction to Energy=	0.217092
Thermal correction to Enthalpy=	0.218036
Thermal correction to Gibbs Free Energy=	0.165471
Sum of electronic and zero-point Energies=	-676.473363

Sum of electronic and thermal Energies= -676.460547
Sum of electronic and thermal Enthalpies= -676.459603
Sum of electronic and thermal Free Energies= -676.512167

TS for 5-NHCH$_3$[9MG – H$_{N2}$ + H$_{N7}$]$^+$
C1 0.030507 -0.513083 -0.509928
C2 -0.829110 0.498618 -0.055823
C3 0.842542 2.004656 0.141619
C4 1.412640 -0.190471 -0.887236
C5 -2.041453 -1.258924 -0.623259
N6 -0.499249 1.700489 0.322510
O7 2.165381 -0.965026 -1.438890
N8 -2.122475 -0.043194 -0.092447
N9 1.363925 3.114318 0.522624
H10 0.655244 3.702284 0.960373
N11 -0.766483 -1.555615 -0.910827
N12 0.884465 -1.175094 1.565631
H13 0.066925 -1.186259 2.188020
C14 1.402588 -2.514894 1.476872
H15 2.134211 -2.586426 0.668372
H16 1.930772 -2.724566 2.421598
H17 0.633494 -3.294268 1.378172
H18 -2.881574 -1.915300 -0.799854
C19 -3.329837 0.668464 0.331760
H20 -3.261193 0.877645 1.399438
H21 -3.396140 1.605979 -0.220605
H22 -4.200032 0.047590 0.123762
N23 1.691562 1.102810 -0.527016
H24 2.640806 1.430844 -0.670625
H25 -0.435033 -2.409508 -1.344890

Zero-point correction= 0.197868 (Hartree/Particle)
Thermal correction to Energy= 0.211277
Thermal correction to Enthalpy= 0.212221
Thermal correction to Gibbs Free Energy= 0.157714
Sum of electronic and zero-point Energies= -676.398938
Sum of electronic and thermal Energies= -676.385528
Sum of electronic and thermal Enthalpies= -676.384584
Sum of electronic and thermal Free Energies= -676.439091

8-NHCH$_3$[9MG – H$_{N2}$ + H$_{N7}$]$^+$
C1 -0.391643 0.803712 -0.136148
C2 -0.273927 -0.654917 -0.090974
C3 -2.546996 -0.877311 0.114548
C4 -1.718216 1.477828 -0.018627
C5 1.858702 0.289934 -0.349089
N6 -2.693493 0.542588 0.089522
N7 -1.275223 -1.451270 0.023365
O8 -1.822056 2.684353 -0.033255
N9 1.046860 -0.922837 -0.177757
N10 -3.624905 -1.529119 0.225150
H11 -3.442592 -2.531493 0.235275
N12 0.764593 1.328922 -0.276786
N13 2.868110 0.548840 0.577817
H14 2.631717 0.306758 1.532103
C15 4.264451 0.304860 0.208980
H16 4.489264 0.818587 -0.728395
H17 4.903248 0.735470 0.979947
H18 4.505578 -0.758775 0.102598
H19 2.274768 0.336817 -1.364130
C20 1.638295 -2.250229 -0.215784
H21 0.848399 -2.977229 -0.027320
H22 2.080926 -2.445025 -1.196511
H23 2.402368 -2.341619 0.559374
H24 0.950925 2.331461 -0.297665
H25 -3.653741 0.867095 0.164157

Zero-point correction= 0.204029 (Hartree/Particle)
Thermal correction to Energy= 0.217144
Thermal correction to Enthalpy= 0.218088
Thermal correction to Gibbs Free Energy= 0.164161
Sum of electronic and zero-point Energies= -676.458147
Sum of electronic and thermal Energies= -676.445032
Sum of electronic and thermal Enthalpies= -676.444088
Sum of electronic and thermal Free Energies= -676.498015

TS for 8-NHCH$_3$[9MG – H$_{N2}$ + H$_{N7}$]$^+$
C1 -0.218213 0.763347 -0.499607
C2 -0.053843 -0.617508 -0.381181
C3 -2.195654 -0.935449 0.267731
C4 -1.492652 1.411298 -0.205995
C5 1.898493 0.290381 -0.937906
N6 -2.409447 0.448275 0.160739
N7 -0.969257 -1.484910 -0.010823
O8 -1.696127 2.604802 -0.273357
N9 1.266200 -0.885054 -0.725490
N10 -3.215657 -1.637776 0.644454
H11 -2.957999 -2.624274 0.687993
N12 0.965426 1.286830 -0.895351
N13 2.891119 0.614999 0.871399
H14 3.255592 1.574118 0.895102
C15 2.021288 0.409784 1.995573
H16 1.650065 -0.617341 2.015817
H17 2.575773 0.601539 2.924690
H18 1.167897 1.109252 2.004813
H19 2.828098 0.376358 -1.478993
C20 1.927916 -2.184388 -0.660610
H21 1.171859 -2.931088 -0.421932
H22 2.382373 -2.417244 -1.624073
H23 2.692311 -2.158538 0.119828
H24 1.128232 2.264418 -1.106766
H25 -3.347891 0.763055 0.387856

Zero-point correction= 0.198141 (Hartree/Particle)
Thermal correction to Energy= 0.211277
Thermal correction to Enthalpy= 0.212221
Thermal correction to Gibbs Free Energy= 0.158431
Sum of electronic and zero-point Energies= -676.400397
Sum of electronic and thermal Energies= -676.387260
Sum of electronic and thermal Enthalpies= -676.386316
Sum of electronic and thermal Free Energies= -676.440106
